# Atom and step economical synthesis of acyclic quaternary centers *via* iridium-catalyzed hydroarylative cross-coupling of 1,1-disubstituted alkenes†

**DOI:** 10.1039/d2sc02790a

**Published:** 2022-09-13

**Authors:** Phillippa Cooper, Andrew G. Dalling, Elliot H. E. Farrar, Timothy P. Aldhous, Simon Grélaud, Eleanor Lester, Lyman J. Feron, Paul D. Kemmitt, Matthew N. Grayson, John F. Bower

**Affiliations:** School of Chemistry, University of Bristol Bristol BS8 1TS UK; Department of Chemistry, University of Bath Bath BA2 7AY UK M.N.Grayson@bath.ac.uk; Medicinal Chemistry, Oncology, IMED Biotech Unit, AstraZeneca Cambridge UK; Department of Chemistry, University of Liverpool Crown Street Liverpool L69 7ZD UK john.bower@liverpool.ac.uk

## Abstract

Quaternary benzylic centers are accessed with high atom and step economy by Ir-catalyzed alkene hydroarylation. These studies provide unique examples of the use of non-polarized 1,1-disubstituted alkenes in branch selective Murai-type hydro(hetero)arylations. Detailed mechanistic studies have been undertaken, and these indicate that the first irreversible step is the demanding alkene carbometallation process. Structure-reactivity studies show that the efficiency of this is critically dependent on key structural features of the ligand. Computational studies have been undertaken to rationalize this experimental data, showing how more sterically demanding ligands reduce the reaction barrier *via* predistortion of the reacting intermediate. The key insight disclosed here will underpin the ongoing development of increasingly sophisticated branch selective Murai hydroarylations.

## Abstract 2

Quaternary benzylic centers are accessed with high atom and step economy by intermolecular Ir-catalyzed hydroarylation of non-polarized 1,1-disubstituted alkenes.

## Graphical abstract

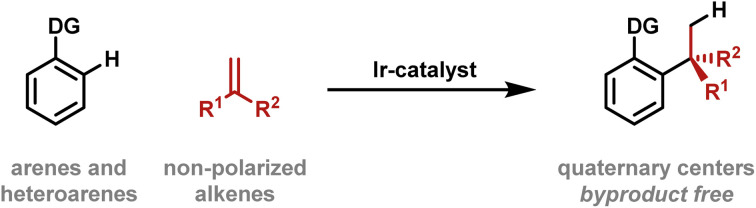

## Introduction

**Scheme 1:**
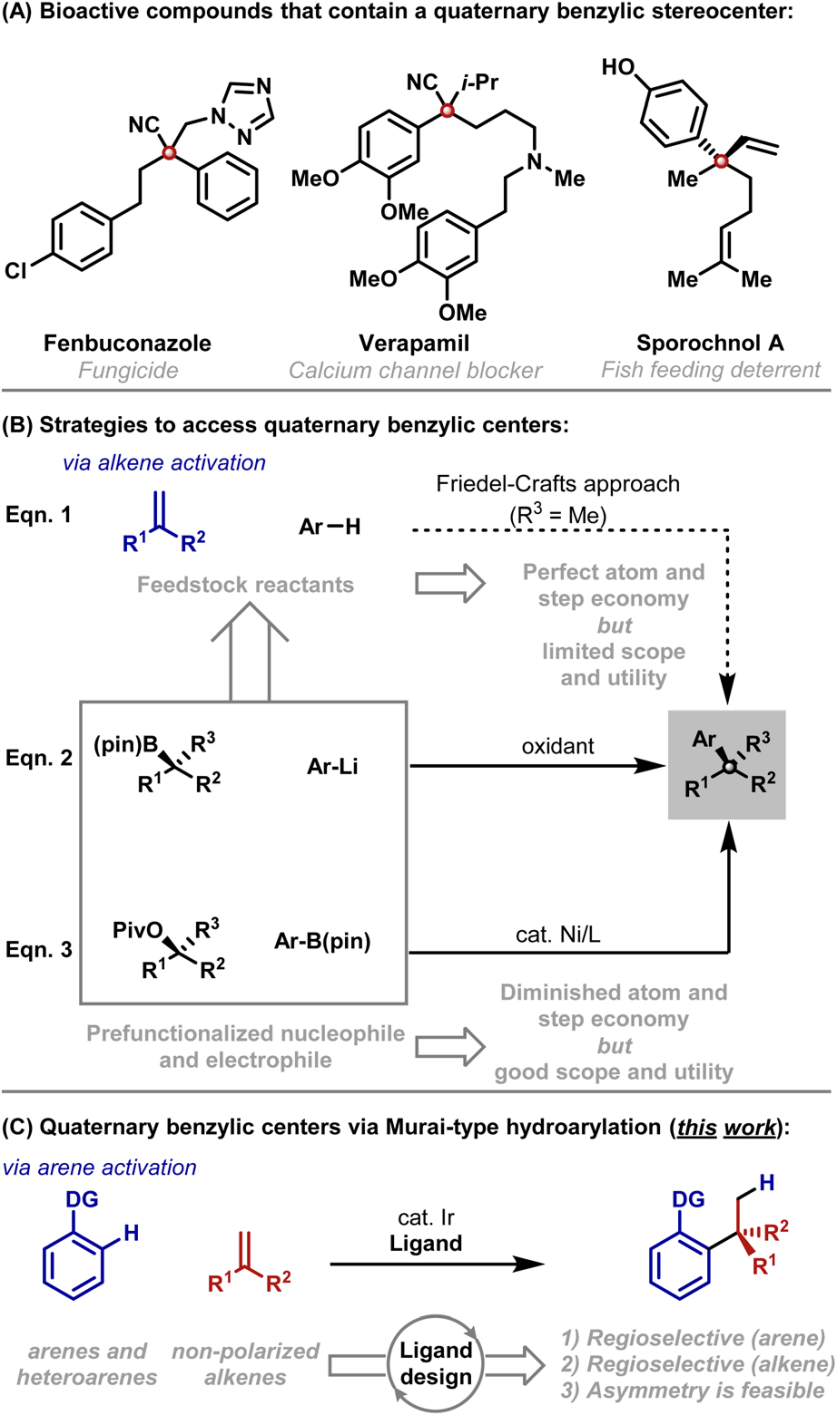
Introduction.

It is well appreciated that the synthesis of acyclic quaternary centers is challenging. The development of step and atom economical methods, especially those that can be adapted to enantioselective settings, represents an enduring challenge.^1^ Aryl substituted quaternary centers are especially important as they are key motifs in a variety of bioactive compounds (Scheme 1A).^2^ A conceptually ideal framework for accessing these subunits involves Friedel–Crafts-type addition of an aryl C–H bond across a 1,1-disubstituted alkene; however, this approach is notoriously problematic, offers limited scope and is not well suited to enantioselective protocols (Scheme 1B, eqn (1)).^3^ These limitations have stimulated the development of other methods that require prefunctionalized reactants. For example, “formal” (non-enantioselective) alkene hydroarylations can be achieved through the use of a reductant and an aryl halide.^4^ Outside of allylic substitution^5^ and conjugate addition reactions,^6^ the most prominent asymmetric arylative protocols for accessing acyclic quaternary centers rely on the oxidative coupling of α-tertiary boronic esters with aryl lithium reagents (eqn (2)).^7^ Ni-catalyzed cross-couplings of α-tertiary benzylic acetates with aryl boronic esters offer a powerful alternative (eqn (3)).^8^

As outlined above, state of the art cross-coupling methods require substantial levels of prefunctionalization, and the desired stereochemistry is established in an earlier step, thereby detracting from atom and step economy. Consequently, we considered whether the Friedel–Crafts approach might be re-engineered to offer broader scope (*e.g.* a wider electronic range) and greater control (*e.g.* predictable mono-alkylation and regiocontrol). This would then provide a viable method where feedstock reactants are used directly, and C–C bond formation and stereocenter creation are united.^9,10^ To achieve this, we envisaged initiating the reaction by metal-catalyzed C–H activation of the arene rather than by Friedel–Crafts-type activation of the alkene (Scheme 1C). Murai hydroarylations of this type are extremely rare,^11^ with Ellman's Rh-catalyzed method for the branch selective hydroheteroarylation of methyl methacrylate with specific classes of *N*-heteroarene being the most significant development so far.^12^ The paucity of methods reflects wider difficulties in developing intermolecular branch selective Murai-hydroarylations.^11*b*–*d*^ Indeed, broad scope protocols involving non-polarized mono-substituted alkenes have only been developed recently,^13^ whereas processes involving non-polarized 1,1-disubstituted alkenes have remained elusive. The key impediment lies in designing catalysts that can tolerate sterically demanding alkenes, and, at the same time, exert threefold control of (1) C–H bond activation regioselectivity, (2) branched : linear regioselectivity^11*b*^ and (3) enantioselectivity.^11*c*^ In this report, we outline efforts to address these objectives, which have resulted in an efficient C–H activation protocol for the hydro(hetero)arylation of non-polarized 1,1-disubstituted alkenes. Our results make a significant contribution to the area of Murai-type hydroarylations,^11*c*^ provide an enabling cross-coupling methodology and set the stage for the development of an enantioselective method.

## Results and discussion

### Reaction development and scope

We have previously developed an Ir-system modified with bisphosphite ligand L-1 for the enantioselective hydroarylative cross-coupling of anilide *ortho*-C–H bonds with monosubstituted alkenes.^13,14^ These processes are highly efficient and this prompted us to explore hydroarylations of much more demanding 1,1-disubstituted alkenes. Initially, we focused on generating 2a by benzamide directed cross-coupling of 1a with *p*-phenyl-α-methylstyrene (Table 1). Remarkably, use of [Ir(cod)_2_]BARF/L-1 provided 2a in 68% yield and, importantly, with >20 : 1 branched : linear (B : L) selectivity (entry 1). As part of extensive optimization efforts, we varied the electronics of the flanking biaryl units of the ligand. Replacement of the *t*-Bu groups of L-1 with chloride, methoxy or trifluoromethyl groups (L2–4) resulted in lower yields (entries 2–4). Pleasingly, L-5 (Y = H) was effective, and this allowed 2a to be isolated in 73% yield (entry 5). Higher catalyst loadings led to diminished yields and 1,4-dioxane was found to be the optimal solvent.^15^ Ir-precatalysts possessing more strongly coordinating counterions (*e.g.* triflate) were less effective (entry 6). Replacement of the *N*,*N*-diethyl groups of 1a with other substituents gave targets 2b–e in lower yields (entries 7–10). The attempted use of weaker 6-membered anilide-based chelates or stronger 5-membered 2-pyridyl-based chelates was not successful. As discussed later, reaction efficiency is specifically dependent on the structural features of the central biaryl unit of L-5, such that we have been unable to identify commercially available ligands that offer comparable efficiencies. Additionally, d^F^ppb, a ligand developed previously for branch selective hydroarylations of monosubstituted alkenes,^16^ is ineffective.

**Table 1:** Optimization studies

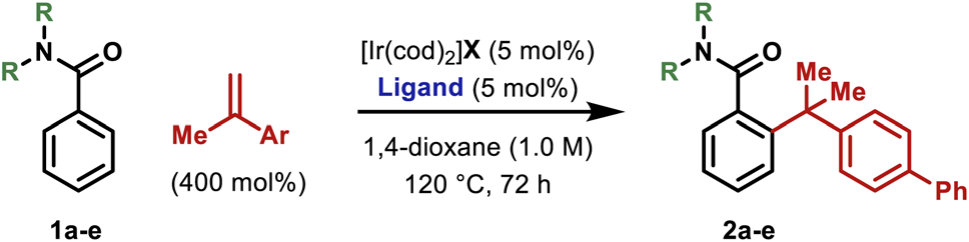					
Entry	R	X	Ligand	B : L selectivity	Yield (%)^a^
1	Et (1a)	BARF	L-1	>20 : 1	68
2	Et	BARF	L-2	>20 : 1	55
3	Et	BARF	L-3	>20 : 1	64
4	Et	BARF	L-4	>20 : 1	37
**5**	**Et**	**BARF**	L-5	**>20** **:** **1**	**75 (73)** b
6	Et	OTf	L-5	>20 : 1	44
7	Me (1b)	BARF	L-5	>20 : 1	48
8	–C_4_H_8_– (1c)	BARF	L-5	>20 : 1	51
9	–C_5_H_10_– (1d)	BARF	L-5	>20 : 1	31
10	i-Pr (1e)	BARF	L-5	>20 : 1	<10
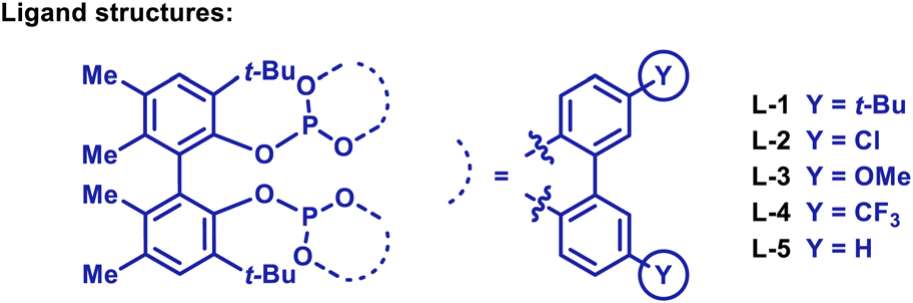					

a NMR yield.

b Isolated yield.

As outlined in Table 2, the optimized conditions offer broad scope. Hydroarylation of electronically diverse α-substituted styrenes with benzamide 1a delivered 2f–i in high yields (Table 2A). In each case, complete branch selectivity was observed and only mono-*ortho*-alkylation of the arene occurred. The latter is consistent with initial monoalkylation causing the directing group to twist from the plane of the arene, such that alkylation of the remaining *ortho*-position cannot occur. Significantly, the process extends to aliphatic alkenes; for example, 2j was accessed in 57% yield. Substitution at the *meta*- and *para*-positions of the benzamide is tolerated (2k–m). For 2l, C–C bond formation was highly selective for the less hindered *ortho*-position, and this presumably reflects the steric demands of L-5. Very electron poor arenes (*e.g.* R^1^ = *p*-NO_2_) are not suitable, perhaps because these systems are less effective at stabilizing the cyclometallated Ir(iii)-intermediate (*vide infra*).

**Table 2:** Scope of the alkene hydroarylation protocol

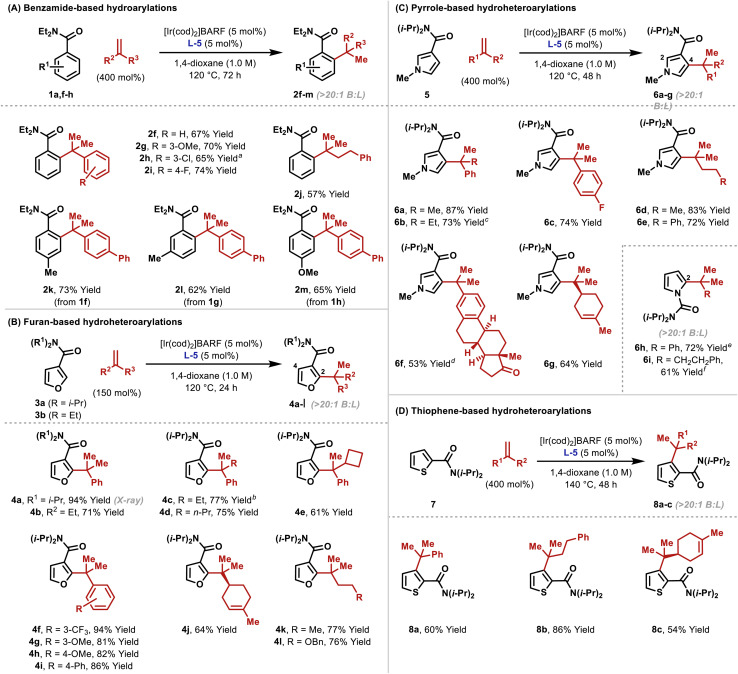

a [Ir(cod)_2_]BARF/L-5 (7.5 mol%).

b 4c was formed in 60 : 40 e.r. using (*S*)-L-5 (see the ESI).

c The reaction time was 16 h.

d Using alkene (150 mol%) over 72 h.

e An 11 : 1 ratio of mono- (6h) to 2,5-dialkylated products formed.

f Using alkene (150 mol%).

To highlight complementarity to Friedel–Crafts alkylations, we evaluated hydroheteroarylations using electron rich 5-membered heteroaromatics. Cross-coupling of furans 3a–b with α-methylstyrene revealed that an *N*,*N*-diisopropyl directing group is more efficient than an *N*,*N*-diethyl variant (Table 2B). In both cases, alkylation was observed at the furan C2 position only, which likely reflects an electronic preference. Quaternary benzylic stereocenters could be installed (4c–e), and electronically diverse styrenes underwent efficient hydroheteroarylation to afford furans 4f–i. The cross-coupling extended to alkenes where the R^2^ and R^3^ groups are both aliphatic, to give, for example, 4j–l. For the former, the generation of a quaternary center adjacent to a tertiary center is notable, especially as complete branch selectivity was maintained and competing hydroheteroarylation of the trisubstituted alkene was not observed. To validate the prospects of an enantioselective protocol, we prepared L-5 as a single enantiomer, and this provided 4c in 60 : 40 e.r. Further improvements are clearly required, and this aspect will be facilitated by the mechanistic studies outlined later.

To the best of our knowledge, examples of pyrroles participating in intermolecular branch selective Murai-type processes have not been reported. Consequently, we were pleased to find that the protocol extended to pyrroles possessing directing groups at C3. Hydroheteroarylation of α-methyl and α-ethyl styrene with pyrrole 5 afforded products 6a and 6b in 87% and 73% yield, respectively, and with high selectivity for the C4 position (Table 2C). Here, steric effects of the *N*-Me group likely disfavor bond formation at C2 (*cf.*Table 2B). As with furan-based processes, electronically distinct styrenes and non-activated aliphatic alkenes are viable reaction partners (6c–g). The directing group can also be placed on the pyrrole nitrogen, and this allowed the generation of C2-alkylated pyrroles 6h–i. These results validate the use of a distinct pyrrole unit and demonstrate that the method offers a true alternative to problematic Friedel–Crafts reactions of (acid sensitive) pyrroles.

To explore scope further, we examined hydroheteroarylations with thiophene 7, which possesses a C2 directing group (Table 2D). Although this substrate required a higher reaction temperature (140 °C *vs.* 120 °C for other processes), both styrenic and “all-aliphatic” 1,1-disubstituted alkenes participated to provide 8a–c in 54–86% yields. As with 6g, the trisubstituted alkene associated with 8c did not undergo hydroheteroarylation.

### Mechanistic studies

**Scheme 2:**
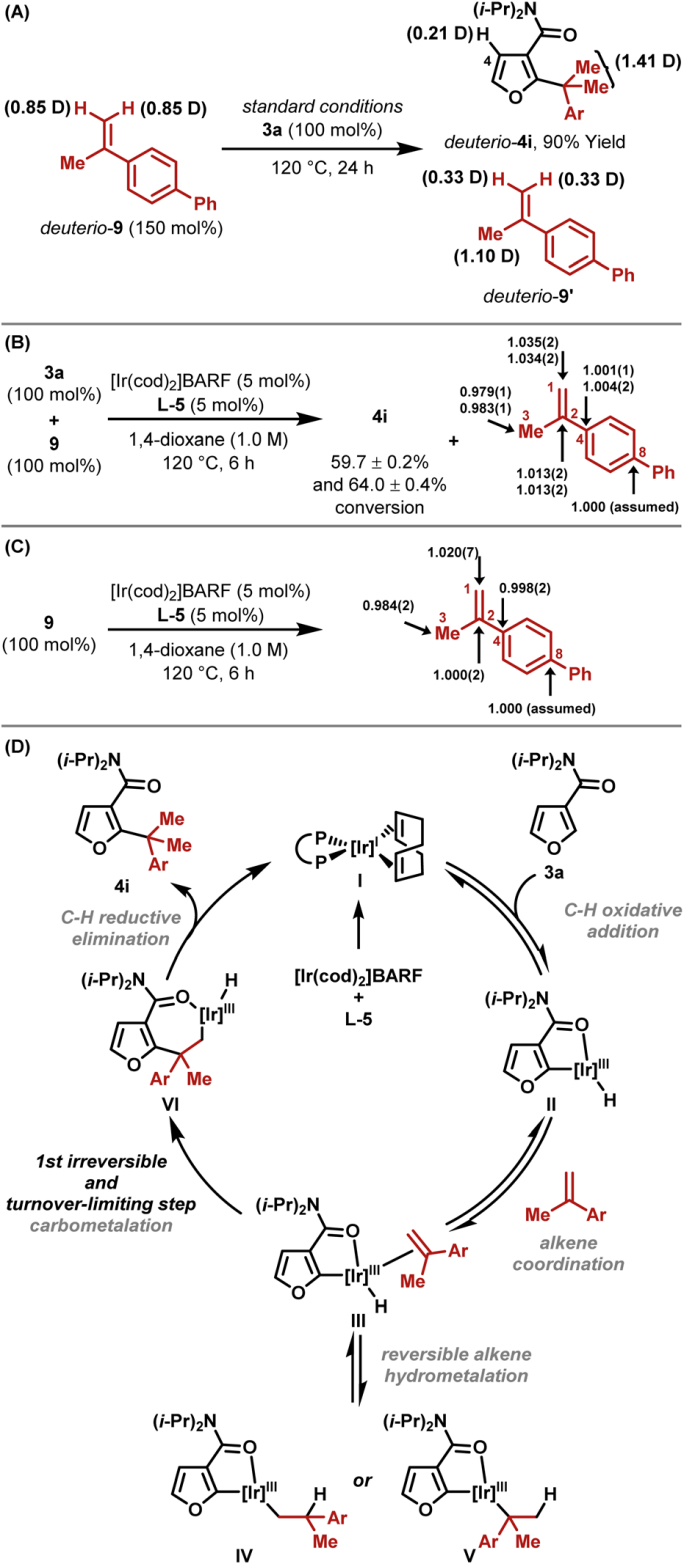
Mechanistic studies.

A series of experiments support the working mechanistic hypothesis in Scheme 2D. Hydroheteroarylation of *deuterio*-9 with furan 3a resulted in deuterium transfer to C4–H of *deuterio*-4i, and scrambling in recovered *deuterio*-9′ (Scheme 2A). These results indicate (a) that *ortho*-C–H activation is reversible and non-selective for C4–H *vs.* C2–H, (b) that reversible alkene hydrometallation is operative (III to IV and V) and (c) that the alkene isomerizes under the reaction conditions. Based on these data, C–C bond formation could occur either *via* C–C reductive elimination from V or *via* carbometallation from III. To distinguish these options, natural abundance ^13^C KIE experiments were undertaken on alkene 9 using the Singleton method, which establishes which carbon centers are involved in the first irreversible step.^17^ This revealed significant KIEs at both C2 (1.013) and C1 (1.035), as well as a large inverse KIE at C3 (0.981) (Scheme 2B). The KIEs were determined by integration of ^13^C NMR data for recovered *versus* starting alkene. In this case, this provides an enhanced KIE at C1 and a diminished KIE at C3, because the starting alkene is depleted in carbon-13 at C1 relative to C3 owing to KIEs that are inherent to the method used for its synthesis.^18^ Under the hydroheteroarylation conditions, alkene isomerization between C1–C2 and C2–C3 occurs, such that a (more) even carbon-13 distribution is established. To confirm this, alkene 9 was exposed to the reaction conditions in the absence of arene 3a; integration of the ^13^C NMR spectra of recovered 9 revealed a relative enhancement at C1 (1.020) and a depletion at C3 (0.984), with the average approximating unity as expected (Scheme 2C). In Scheme 2B, the sum of the KIE enhancements at C1 and C3 is 0.016; if this is derived solely from the terminal alkene carbon during C–C bond formation, then a hypothetical “isomerization-free” KIE value for this center can be approximated as 1.016, which is similar to the value obtained for C2.^19^ Accordingly, C–C bond formation likely occurs *via* irreversible alkene carbometallation from III (both C-centers involved), and the large inverse KIE at C3 in Scheme 2B is an artefact of both the method used for alkene synthesis and alkene isomerization under the reaction conditions. The assertion that carbometallation is irreversible contrasts earlier work with monosubstituted alkenes,^13^ and likely reflects the increased steric demands of 1,1-disubstituted variants. Branched selectivity is presumably favored during carbometallation because the bulky Ir-center ends up at the less hindered position. The processes described here require 5-membered amide-based chelates, which are presumably sufficiently tractable to be intercepted by the hindered alkene. Weaker 6-membered anilide-based chelates are not suitable.^13^

### Ligand effects and computational studies

As mentioned earlier, the structural features of the central biaryl unit of L-5 are critical for efficient reactivity. To probe this, structurally varied ligands L-6–8 were synthesized and compared for the hydroheteroarylation of 9 with 3a (Table 3). L-5 (R^1^ = Me, R^2^ = *t*-Bu) affords 4i in 86% yield (entry 1). Reducing the size of the R^2^ substituent (L-6, R^1^ = R^2^ = Me) decreased the yield slightly (76%), whereas removal of this unit (L-7) significantly lowered the yield (19%) (entry 2–3). Removal of the R^1^ substituent (L-8, R^1^ = H, R^2^ = H) had a less significant effect (entry 4). NMR profiling indicated that the yields are reflected in the reaction rates (see the ESI†).

**Table 3:** Studies on key ligand structural features

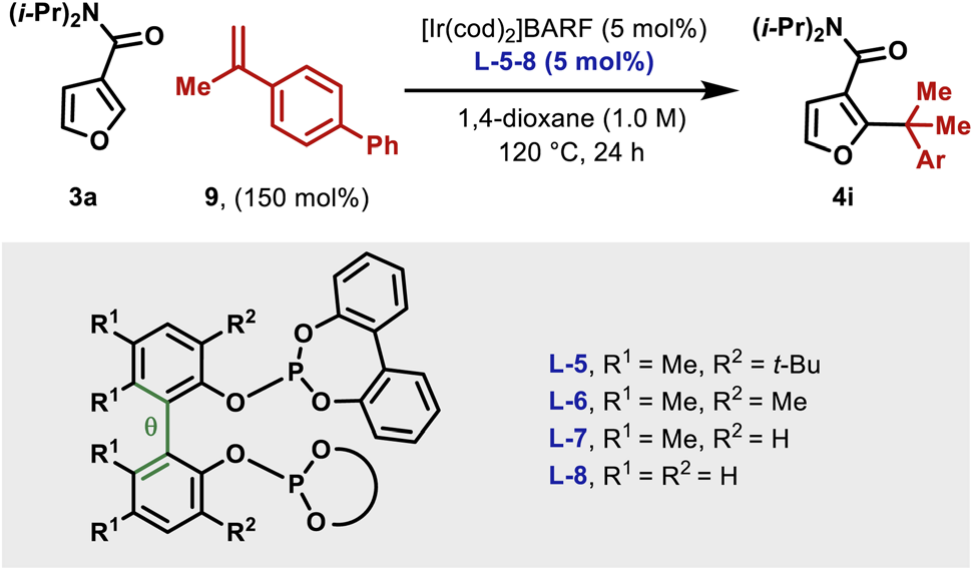				
Entry	Ligand	Yield of 4i	Rel. barrier to TS-III (ΔΔ*G*; kcal mol^−1^)	*δ θ*III to TS-III (°)
1	L-5	86%	0	−9.2
2	L-6	76%	2.5	−7.8
3	L-7	19%	5.7	13.9
4	L-8	12%	6.5	30.5
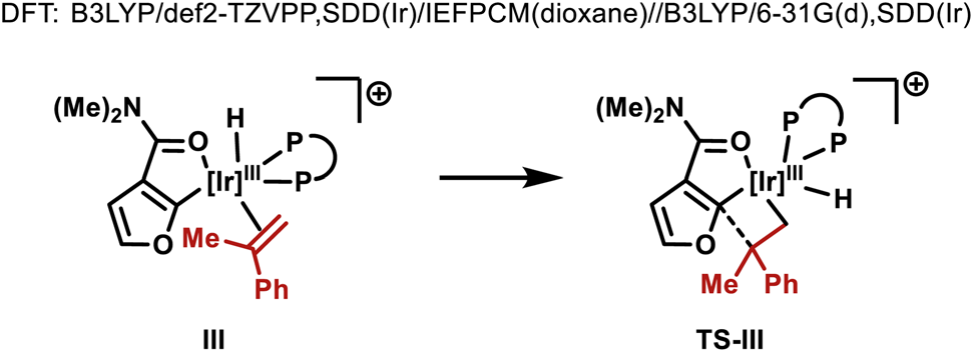				

**Scheme 3:**
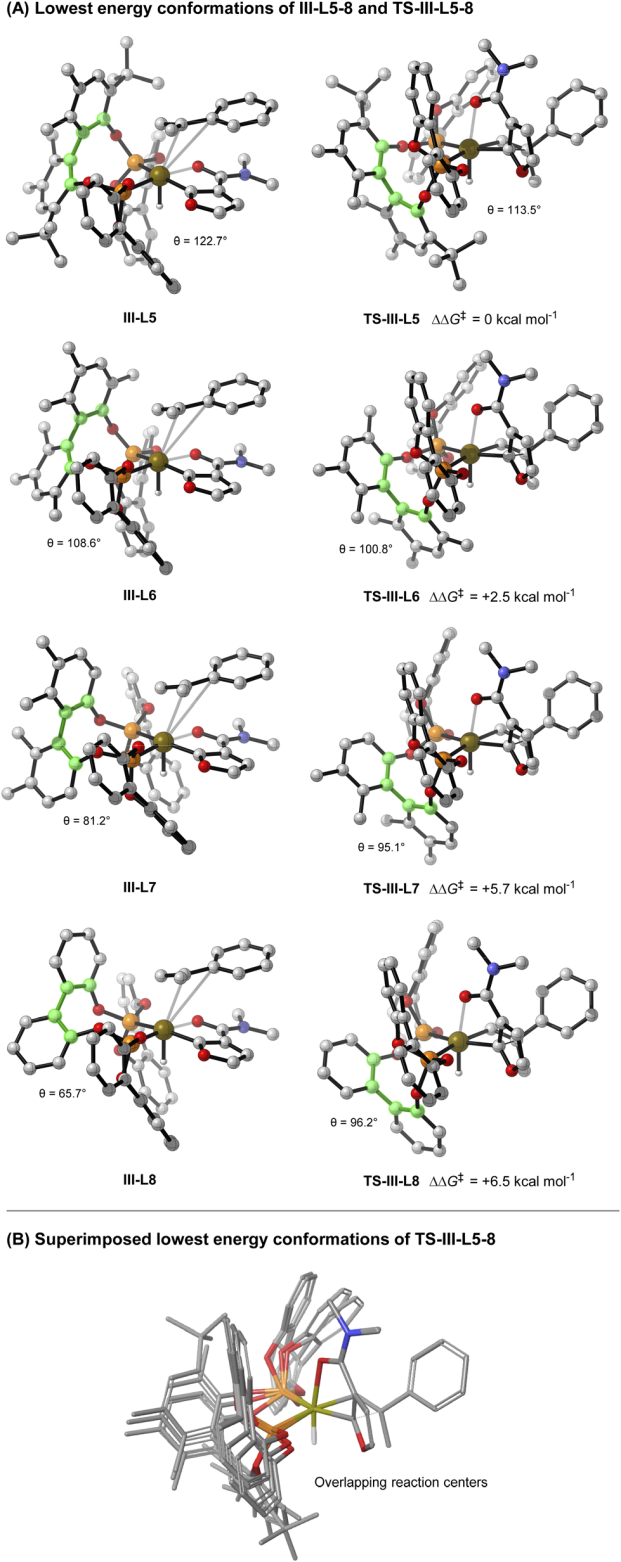
Computational studies (B3LYP/def2-TZVPP,SDD(Ir)/IEFPCM(dioxane)//B3LYP/6-31G(d),SDD(Ir)). Green highlighted atoms indicate measured dihedral angle *θ*.

Given the observed significance of the R^2^ substituent, the turnover-limiting carbometallation event (III to TS-III) was modelled with DFT using Gaussian 16 (full details are given in the ESI†).^20^ A thorough conformational analysis was performed to identify the lowest energy conformations of III-L-5–8 and TS-III-L-5–8; as expected, their energies were found to be significantly lower with the amide oxygen coordinated to the Ir(iii)-center. The lowest energy conformations of each species and the relative barriers to transition state TS-III from intermediate III are provided in Scheme 3A. The relative barriers show an increase upon progression from L-5 to L-8, which correlates with the trend in yield for 4i. As shown by superimposing the transition states of type TS-III for ligands L-5-8, the Ir(iii) reaction center is nearly identical for each ligand (Scheme 3B). However, there are differences in the dihedral angle (labelled *θ* in Scheme 3A and Table 3) between the conformation of ligands L-5–8 in each of the transition states. The difference in the dihedral angle between intermediates of type III and transition states TS-III has an approximately linear relationship with the relative reaction barrier for carbometallation, and hence the yield of furan 4i. When ligands with more sterically demanding substituents are used, this difference is smaller. As a result, the energy required to distort the intermediate into its transition state geometry, a principal contributor to the reaction barrier, decreases.^21^ Thus, the barrier to carbometallation is determined primarily by the extent to which the conformation of the ligand is predistorted in intermediate III.

## Conclusions

In summary, we report unique examples of branch selective Murai-type hydro(hetero)arylations of non-polarized 1,1-disubstituted alkenes. The chemistry is enabled by key structural features of L-5, a ligand that is accessed in just one step. Experimental trends in yield over a variety of ligands were examined by DFT calculations, which revealed how more sterically demanding ligands reduce the reaction barrier *via* predistortion of the structure of the reacting intermediate. The protocol provides a broad range of challenging quaternary benzylic centers in an atom and step economical manner, and addresses key problems associated with Friedel–Crafts alkylation (*i.e.* tolerance of electron poor arenes, mild conditions, mono-alkylation only, regioselective, high yields). Consequently, the method can be considered “enabling”, and so we anticipate that it will be of broad interest. Ongoing efforts towards (a) an enantioselective variant and (b) catalysts that can accommodate internal alkenes will be guided by the intriguing ligand design insights outlined here.

## Data availability

Compound characterisation data and Gaussian 16 output files for all computed structures are openly available in the ESI.†

## Author contributions

P. C., A. G. D, T. P. A., S. G. and E. L. performed the experimental work, which was directed by L. J. F., P. D. K. and J. F. B. The computational studies were conducted by E. H. E. F. and M. N. G. The manuscript was written by J. F. B. and M. N. G. with contributions from all authors.

## Conflicts of interest

There are no conflicts to declare.

## Supplementary Material

SC-013-D2SC02790A-s001

SC-013-D2SC02790A-s002
