# Supplementary material for: Atom and step economical synthesis of acyclic quaternary centers via iridium-catalyzed hydroarylative cross-coupling of 1,1-disubstituted alkenes
Source: Chem Sci. 2022 Sep 13;13(37):11183–9. doi: 10.1039/d2sc02790a (PMC9516889; doi:10.1039/d2sc02790a)
Supplement: SC-013-D2SC02790A-s001 [file SC-013-D2SC02790A-s001.pdf]

Electronic Supplementary Material (ESI) for Chemical Science.
This journal is © The Royal Society of Chemistry 2022

Atom and Step Economical Synthesis of Acyclic Quaternary Centers via Iridium-Catalyzed Hydroarylative Cross-Coupling of 1,1-Disubstituted Alkenes

Phillippa Cooper,† Andrew G. Dalling,† Elliot H. E. Farrar,§ Timothy P. Aldhous,†,¥ Simon Grélaud,† Eleanor Lester,† Lyman. J. Feron,‡ Paul D. Kemmitt,‡ Matthew N. Grayson,*,§ and John F. Bower*,¥

† School of Chemistry, University of Bristol, Bristol, BS8 1TS, United Kingdom
‡ Medicinal Chemistry, Oncology, IMED Biotech Unit, AstraZeneca, Cambridge, United Kingdom
§ Department of Chemistry, University of Bath, Bath, BA2 7AY, United Kingdom
¥ Department of Chemistry, University of Liverpool, Crown Street, Liverpool, L69 7ZD, United Kingdom

Supporting Information

Table of Contents

General Experimental Details **S2**

Experimental Procedures and Data **S3**

General Procedures S3

Synthesis of Bisphosphite Ligands S5

Synthesis of Substrates S10

Heteroaromatic Substrates S10

Synthesis of Alkene Substrates S13

Reaction Scope S17

Deuterium Labelling Experiments S36

^{13}C KIE Determination Experiments S38

NMR profiling Experiments S45

Computational Details **S46**

Copies of ^{1}H and ^{13}C NMR Spectra for Novel Compounds **S82**

References **S132**

General Experimental Details

All materials for which a synthetic route is not described or referenced were purchased from commercial sources (Acros, Sigma-Aldrich, Alfa Aesar, Fluorochem, Strem and TCI). Catalytic reactions were carried out in Young-type re-sealable tubes. Liquid styrene derivatives were distilled using a Hickman distilling head before use. All other commercially available alkenes were used as received without any further purification. Iridium pre-catalysts were synthesized according to previously reported procedures.[1] Anhydrous solvents were obtained by distillation using standard procedures or by passage through drying columns supplied by Anhydrous Engineering Ltd. Anhydrous 1,4-dioxane was distilled from sodium benzophenone ketyl and sparged with argon for 10 minutes prior to use. All reactions were performed using dry solvents unless stated otherwise. Et_3N was distilled over CaH_2 and stored over activated 4Å molecular sieves under nitrogen. The removal of the solvents *in vacuo* was achieved by employing rotary evaporators connected with diaphragm pumps (15 mmHg) or, for high-boiling solvents, oil pumps (0.1 mmHg). Materials were then dried on a high-vacuum line prior to analysis. Reactions requiring anhydrous conditions were performed under a nitrogen atmosphere, using Schlenk techniques and flame/oven-dried equipment. In particular, catalytic reactions were carried out in oven dried (minimum 2 hours) or flame dried Young-type re-sealable tubes. Flash column chromatography (FCC) was performed using silica gel (Aldrich 40−63 µm, 230−400 mesh). Ligands were purified by chromatography on deactivated silica gel (stirred overnight with 10% w/w of Et_3N). Thin layer chromatography was performed using aluminium backed 60 F_{254} silica plates. Visualization was achieved by UV fluorescence or a basic $KMnO_4$ solution and heat. Proton nuclear magnetic resonance spectra (NMR) were recorded at 400 MHz or 500 MHz as stated. ^{13}C NMR spectra were recorded at 100 MHz or 125 MHz as stated. Chemical shifts (δ) are given in parts per million (ppm). Peaks are described as singlets (s), doublets (d), triplets (t), quartets (q), septets (sept), multiplets (m) and broad (br.). Coupling constants (*J*) are quoted to the nearest 0.5 Hz. All assignments of NMR spectra were based on 2D NMR data ($DEPT^{135}$, COSY, HSQC and HMBC). Where compounds were isolated as a mixture of isomers (*e.g.* rotamers), they are referred as *A* and *B*. NMR yields were determined against 1,3,5-trimethoxybenzene as an internal standard. Mass spectra were recorded using a Bruker MicroTof (ESI+ mode) and a Bruker Ultraflex II (MALDI). Infrared spectra were recorded on a Perkin Elmer Spectrum Two FTIR spectrometer as thin films or solids compressed on a diamond plate. Melting points were determined using Reichert melting point apparatus and are uncorrected. Optical rotations were measured using a $ADP440^+$ polarimeter at the concentration and temperature stated. Enantiomeric excess was determined using an Agilent 1290 Infinity chiral SFC as stated for each compound.

Experimental Procedures and Data

General Procedures

General Procedure A: for the carbamoyl protection of heteroaromatics from acids

The title compounds were prepared following a modified literature procedure.[2] An oven-dried flask was charged with the corresponding acid (100 mol%) and DMF (2 drops) in CH_2Cl_2 (0.2 M) under nitrogen and the solution was cooled to 0 °C. Oxalyl chloride (120 mol%) was added dropwise over 5 minutes and the resulting solution was stirred for 2 h at 0 °C. The solvent was removed *in vacuo*, before the residue was dissolved in CH_2Cl_2 (0.2 M), purged with nitrogen and cooled to 0 °C. Diisopropylamine (200 mol%) was added dropwise over 5 minutes, before the solution was warmed to ambient temperature and stirred overnight. The reaction mixture was quenched with aq. 1M HCl (5 mL/mmol) and extracted with CH_2Cl_2 (3 × 5 mL/mmol). The organic extracts were combined, washed with saturated aq. NaOH (5 mL/mmol) and brine (5 mL/mmol), dried over Na_2SO_4 and concentrated *in vacuo*. Purification of the residue by FCC afforded the title compounds.

General Procedure B: for the preparation of amide substrates from liquid acid chlorides

To an ice-cooled (0 °C) solution of amine (100 mol%) and Et_3N (120 mol%) in CH_2Cl_2 (0.2 M) was added dropwise the acid chloride (120 mol%). The reaction was warmed to ambient temperature and stirred overnight. Water (5 mL/mmol) was added, and the mixture was extracted with CH_2Cl_2 (3 × 5 mL/mmol). The organic extracts were combined, washed with brine (5 mL/mmol), dried over Na_2SO_4 and concentrated *in vacuo* to provide the crude product. Purification by flash column chromatography (hexane EtOAc 20–50%) afforded the pure amides.

General Procedure C: for the preparation of α-methyl styrene substrates

To a flame-dried flask was added methyltriphenylphosphonium bromide (120 mol%) in anhydrous THF (0.5 M) under nitrogen and the solution was cooled to 0 °C. Potassium *tert*-butoxide (120 mol%) was added portion-wise and the resulting solution was stirred at 0 °C for 1–2 h. The relative ketone was added dropwise and after stirring for 20 min at 0 °C the solution was warmed to ambient temperature and stirred overnight. The solution was filtered with hexane and concentrated *in vacuo* to provide the crude product. Purification by FCC afforded the pure styrene.

General Procedure D: for the formation of quaternary centres on 5-membered heteroaromatics

A flame-dried tube, fitted with a magnetic stirrer, was charged with substrate (0.10 mmol), $[Ir(cod)_2]$BARF (5.0 mol%) and **L-5** (5.0 mol%). The tube was fitted with a rubber septum and purged with nitrogen. Styrene derivative (150–400 mol%) in anhydrous 1,4-dioxane (1.0 M concentration with respect to heteroaromatic substrate) was added. The tube was fitted with a Young's tap and the reaction

mixture was heated to 120 °C for 16–48 h before being cooled to ambient temperature and concentrated *in vacuo*. Purification of the residues by FCC afforded the pure products.

General Procedure E: for the formation of quaternary centres on benzamide substrates

A flame-dried tube, fitted with a magnetic stirrer, was charged with substrate (0.10 mmol), [Ir(cod)$_2$]BARF (5.0 mol%) and **L-5** (5.0 mol%). The tube was taken into a glove box where styrene (400 mol%) and anhydrous 1,4-dioxane (1.0 M concentration with respect to benzamide substrate) were added. The tube was fitted with a Young's tap and removed from the glove box. The reaction mixture was then heated to 120 °C for 72 h before being cooled to ambient temperature and concentrated *in vacuo*. Purification of the residues by FCC afforded the pure products.

Synthesis of Bisphosphite Ligands

<u>General Procedure F:</u> for the synthesis of bisphosphite ligands[3]

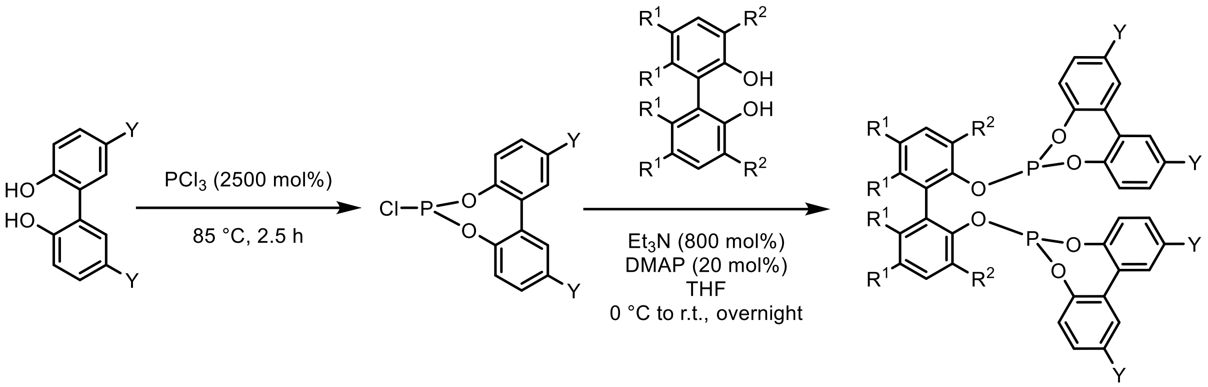

A flame-dried resealable tube under an atmosphere of nitrogen was charged with 2,2'-biphenol derivative (300 mol%). PCl_3 (2500–3000 mol% as specified) was added and the mixture was heated at reflux (85 °C) for 2 h. Following formation of the chlorophosphite, the reaction mixture was cooled to ambient temperature and concentrated under high vacuum for 2 h (a second trap, cooled with liquid nitrogen was placed between the reaction tube and the Schlenk line). The tube was refilled four times with nitrogen during this 2 h period. To the oily or solid chlorophosphite under nitrogen, was added the appropriate biphenol (100 mol%), DMAP (20 mol%), and freshly collected anhydrous THF (~ 0.1 M). The suspension was cooled to 0 °C and anhydrous Et_3N (800 mol%) was added dropwise. The tube was sealed, and the reaction mixture was warmed to ambient temperature and stirred overnight. The reaction mixture was filtered through a pad of Celite® and washed with Et_2O. The solution was concentrated *in vacuo* and purified by flash column chromatography under the conditions specified. The colorless solid that was obtained was azeotropically dried with PhMe (twice) and pentane (twice) to remove any traces of Et_3N to afford the pure ligand.

L-5:

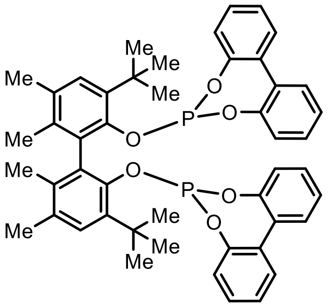

General Procedure F: 2,2'-biphenol (2.10 g, 11.3 mmol, 300 mol%) and PCl_3 (4.93 mL, 56.5 mmol, 2500 mol%) were employed at 85 °C for 2 h. Following formation of the chlorophosphite, 3,3'-di-*tert*-butyl-5,5',6,6'-tetramethyl-[1,1'-biphenyl]-2,2'-diol (600 mg, 2.26 mmol, 100 mol%), DMAP (55.2 mg, 0.45 mmol, 20 mol%), Et_3N (2.52 mL, 18.7 mmol, 800 mol%) and THF (20 mL) were added.

Purification of the crude material by FCC (hexane/EtOAc 5–8%, SiO_2 deactivated with 10% of Et_3N) afforded the title compound **L-5** (1.65 g, 93%) as a colorless solid. ν_{max}/cm^{-1}: 3064 (m), 2958 (m), 2914 (m), 1434 (s); ^1H NMR (400 MHz, C_6D_6): δ 7.40 (2H, s, C**3**-H), 7.23 – 7.12 (8H, m, Ar-CH), 7.05 (2H, td, *J* = 7.7, 1.7 Hz, Ar-CH), 6.98 (2H, td, *J* = 7.7, 1.8 Hz, Ar-CH), 6.92 (4H, tdd, *J* = 7.3, 5.8, 1.3 Hz, Ar-CH), 2.19 (6H, s, C**9**-H_3), 2.02 (6H, s, C**10**-H_3), 1.52 (18H, s, C**8**-H_3); ^{13}C NMR (101 MHz, C_6D_6): δ 150.39 (t, *J* = 2.5 Hz, C**1**), 150.2 (Ar-C), 150.0 (Ar-C), 138.4 (C**2**), 136.6 (C**5**), 132.36 – 131.63 (m, C**11**), 130.0 (Ar-C), 129.9 (Ar-CH), 129.9 (Ar-CH), 129.0 (Ar-CH), 125.2 (Ar-CH), 125.1 (Ar-CH), 123.6 (Ar-CH), 122.9 (Ar-CH), 35.2 (C**7**), 30.9 (C**8**), 20.6 (C**9**), 17.7 (t, *J* = 3.6 Hz, C**10**); ^{31}P NMR (162 MHz, C_6D_6): δ 142.5; HRMS ($MALDI^+$) calculated for $C_{48}H_{48}O_6P_2Na$ 805.2818 Found $[M+Na]^+$ 805.2811; m.p. 152–154 °C (pentane).

5,5',6,6'-Tetramethyl-[1,1'-biphenyl]-2,2'-diol

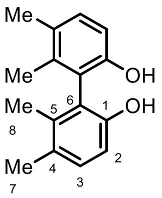

To a solution of 3,3'-di-*tert*-butyl-5,5',6,6'-tetramethyl-[1,1'-biphenyl]-2,2'-diol (5.53 g, 15.6 mmol) in benzene (80 mL) at 0 °C was added a solution of $AlCl_3$ (3.33 g, 25.0 mmol) in nitromethane (15 mL) and benzene (30 mL) dropwise *via* syringe over 30 min. The solution was stirred at 0 °C for 1 h before being carefully quenched with water (50 mL). Et_2O (50 mL) was added, and the layers were separated. The aqueous layer was extracted with Et_2O (2 × 30 mL), and the combined organic layers were washed with brine (100 mL). Purification of the crude material by FCC (hexane/EtOAc 5–25%) afforded the title compound (3.04 g, 80%) as a colorless powder. ^1H NMR (400 MHz, $CDCl_3$): δ 7.14 (2H, d, *J* = 8.0 Hz, 2 × C**3**-H), 6.82 (2H, d, *J* = 8.0 Hz, 2 × C**2**-H), 4.50 (2H, s, 2 × OH), 2.26 (6H, s, 2 × C**7**-H_3), 1.90 (6H, s, 2 × C**8**-H_3); ^{13}C NMR (101 MHz, $CDCl_3$): δ 151.9 (C**1**), 137.1 (C**5**), 131.5 (C**3**), 129.4 (C**6**), 120.4 (C**4**), 112.8 (C**2**), 20.0 (C**7**), 16.5 (C**8**); m.p. 193–195 °C (hexane/CH_2Cl_2) *The spectroscopic properties of this compound were consistent with the data available in the literature*.[4]

6,6'-Dimethoxy-2,2',3,3'-tetramethyl-1,1'-biphenyl

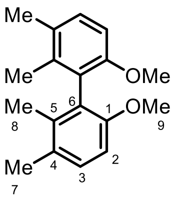

A Schlenk tube was charged with 5,5',6,6'-tetramethyl-[1,1'-biphenyl]-2,2'-diol (2.00 g, 8.25 mmol) and K_2CO_3 (4.56 g, 33.0 mmol) and placed under N_2. DMF (25 mL) and iodomethane (2.05 mL, 33.0 mmol) were added *via* syringe, the tube was sealed and heated to 80 °C for 16 h. The reaction mixture was cooled to ambient temperature and filtered through Celite®, rinsing with EtOAc (50 mL). The filtrate

was transferred to a separating funnel and water (50 mL) was added. The layers were separated, and the organic layer was washed with water (100 mL), brine (100 mL), dried over Na_2SO_4 and concentrated *in vacuo*. Purification of the crude material by FCC (hexane/EtOAc 5–10%) afforded the title compound (1.45 g, 65%) as a colorless solid; m.p. 110–112 °C (hexane/CH_2Cl_2); ^{1}H NMR (400 MHz, $CDCl_3$): δ 7.11 (2H, d, *J* = 8.5 Hz, 2 × C**3**-H), 6.74 (2H, d, *J* = 8.5 Hz, 2 × C**2**-H), 3.66 (6H, s, 2 × C**9**-H_3), 2.27 (6H, s, 2 × C**7**-H_3), 1.83 (6H, s, 2 × C**8**-H_3); ^{13}C NMR (101 MHz, $CDCl_3$): δ 155.5 (C**1**), 136.7 (C**5**), 129.1 (C**3**), 128.7 (C**6**), 127.0 (C**4**), 108.3 (C**2**), 56.1 (C**9**), 20.1 (C**7**), 16.4 (C**8**). *The spectroscopic properties of this compound were consistent with the data available in the literature*.[4]

2,2'-Dimethoxy-3,3',5,5',6,6'-hexamethyl-1,1'-biphenyl

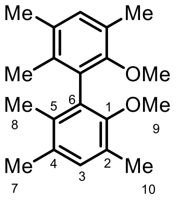

To a suspension of paraformaldehyde (2.44 g) in phosphoric acid (9.20 mL, 85 wt.% in H_2O), conc. aq. HCl (9.20 mL, 37% w/w) and acetic acid (9.20 mL) at ambient temperature was added 6,6'-dimethoxy-2,2',3,3'-tetramethyl-1,1'-biphenyl (1.00 g, 3.70 mmol) in one portion. The suspension was heated to 90 °C for 48 h before being cooled to ambient temperature and diluted with water (50 mL) and PhMe (50 mL). The layers were separated, and the aqueous portion was extracted with PhMe (2 × 30 mL). The combined organic portions were washed with water (100 mL), sat. aq. Na_2CO_3 (50 mL), brine (50 mL) and dried over Na_2SO_4. The solvent was removed *in vacuo* to leave the intermediate benzyl chloride (1.52 g, quant.) as an orange oil, which was used without purification; *only ^{1}H NMR data is listed for this compound*. ^{1}H NMR (400 MHz, $CDCl_3$): δ 7.25 (2H, s, 2 × Ar-CH), 4.81 (2H, d, *J* = 11.0 Hz, 2 × CH_2), 4.57 (2H, d, *J* = 11.0, 2 × CH_2), 3.37 (6H, s, 2 × CH_3), 2.30 (6H, s, 2 × CH_3), 1.94 (6H, s, 2 × CH_3). To a solution of the intermediate benzyl chloride (1.36 g, 3.70 mmol) in anhydrous THF (9 mL) at 0 °C was added $LiAlH_4$ (9.25 mL, 9.25 mmol, 1 M in THF) dropwise over 10 min. The solution was heated to reflux and stirred for 4 h, before being cooled to 0 °C and diluted with Et_2O (10 mL). Water (0.40 mL) was added dropwise, taking care to control the exotherm and evolution of gas, followed by 4 M aq. NaOH (0.40 mL). Water (1.50 mL) was added, and the solution was warmed to ambient temperature and stirred for 15 min. $MgSO_4$ was added and the suspension was stirred for a further 15 min before being filtered through Celite®, rinsing with Et_2O (20 mL). The filtrate was concentrated *in vacuo* and purified by FCC (hexane/EtOAc, 5%) to afford the title compound (794 mg, 72%) as a colorless solid. m.p. 67–70 °C (CH_2Cl_2); ^{1}H NMR (400 MHz, $CDCl_3$): δ 7.00 (2H, s, 2 × C**3**-H), 3.33 (6H, s, 2 × C**9**-H_3), 2.28 (6H, s, 2 × C**10**-H), 2.25 (6H, s, 2 × C**7**-H_3), 1.87 (6H, s, 2 × C**8**-H_3); ^{13}C NMR (101 MHz, $CDCl_3$): δ 154.3 (C**1**), 133.7 (C**4**), 131.9, 131.7 (C**5**, C**6**), 131.5 (C**3**), 127.7 (C**2**), 59.6 (C**9**), 20.2 (C**7**), 16.6 (C**8**), 16.2 (C**10**). *The spectroscopic properties of this compound were consistent with the data available in the literature*.[4]

3,3',5,5',6,6'-Hexamethyl-[1,1'-biphenyl]-2,2'-diol

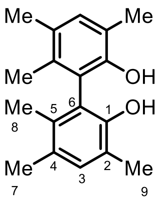

To a solution of 2,2'-dimethoxy-3,3',5,5',6,6'-hexamethyl-1,1'-biphenyl (750 mg, 2.51 mmol) in anhydrous CH_2Cl_2 (15 mL) at 0 °C was added BBr_3 (7.00 mL, 7.00 mmol, 1 M in CH_2Cl_2) dropwise *via* syringe over 10 min. The solution was maintained at 0 °C for 1 h before being diluted with CH_2Cl_2 (20 mL) and quenched with water (20 mL), taking care to control the exotherm. The layers were separated, and the aqueous layer was extracted with CH_2Cl_2 (2 × 20 mL). The combined organic layers were washed with water (50 mL), brine (50 mL), dried over Na_2SO_4 and concentrated *in vacuo*. Purification of the rude material by FCC (hexane/EtOAc 1%) afforded the title compound (446 mg, 66%) as a colorless solid. m.p. 135 – 137 °C (hexane/CH_2Cl_2); ^{1}H NMR (400 MHz, $CDCl_3$): δ 7.00 (2H, s, 2 × C**3**-H), 4.54 (2H, s, 2 × OH), 2.23 (12H, s, 2 × C**7**-H_3, 2 × C**9**-H_3), 1.85 (6H, s, 2 × C**8**-H_3); ^{13}C NMR (101 MHz, $CDCl_3$): δ 149.9 (C**1**), 134.0 (C**6**), 132.8 (C**3**), 128.6 (C**4**), 121.5 (C**2**), 120.0 (C**5**), 19.7 (C**7**), 16.2 (C**8**), 15.8 (C**9**); *The spectroscopic properties of this compound were consistent with the data available in the literature*.[4]

(L-6): 6,6'-((3,3',5,5',6,6'-Hexamethyl-[1,1'-biphenyl]-2,2'-diyl)bis(oxy))didibenzo[*d,f*][1,3,2]dioxaphosphepine

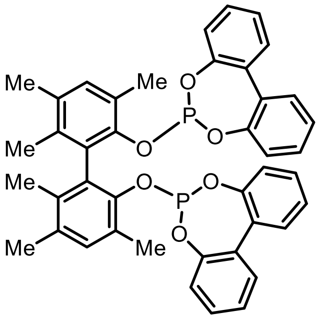

General Procedure F: 2,2'-Biphenol (600 mg, 3.22 mmol, 3 equiv.) and PCl_3 (2.80 mL, 32.2 mmol, 30 equiv.) were employed at 85 °C for 2 h. Following formation of the chlorophosphite, 3,3',5,5',6,6'-hexamethyl-[1,1'-biphenyl]-2,2'-diol (289 mg, 1.07 mmol, 1 equiv.), DMAP (25.6 mg, 0.21 mmol, 0.20 equiv.), Et_3N (0.61 mL, 4.49 mmol, 4.20 equiv.) and THF (10 mL) were employed. Purification of the crude material by FCC (hexane/EtOAc 5%) on neutral alumina afforded the title compound (406 mg, 54%) as a colorless powder. m.p. 88–90 °C (pentane); ν_{max}/cm^{-1}: 2919 (m), 1498 (s), 1474 (s), 1433 (s), 1183 (s); ^{1}H NMR (500 MHz, C_6D_6): δ 7.15 – 7.12 (4H, m, 4 × Ar-CH), 7.02 – 6.88 (14H, m, 14 × Ar-CH), 2.47 (6H, s, 2 × CH_3), 2.13 (6H, s, 2 × CH_3), 2.02 (6H, s, 2 × CH_3); ^{13}C NMR (126 MHz, C_6D_6): δ 149.6 (d, J = 12.5 Hz, Ar-C), 147.5 (Ar-C), 135.2 (Ar-C), 132.7 (Ar-C), 132.2 (Ar-CH), 131.7 (Ar-C), 131.3 (Ar-C), 130.3 (Ar-C), 129.6 (d, J = 17.0 Hz, Ar-CH), 128.7 (d, J = 9.0 Hz, Ar-CH), 124.8 (d, J

= 15.0 Hz, Ar-CH), 122.4 (d, *J* = 49.0 Hz, Ar-CH), 19.7 (CH_3), 17.4 (CH_3), 17.1 (CH_3); ^{31}P NMR (162 MHz, $CDCl_3$): δ 145.6; HRMS (MALDI$^+$): Calculated for $C_{42}H_{36}O_6P_2$: 699.2060. Found [M+H]$^+$: 699.2066.

(L-7): 6,6'-((5,5',6,6'-Tetramethyl-[1,1'-biphenyl]-2,2'-diyl)bis(oxy))didibenzo[*d,f*][1,3,2]dioxa-phosphepine

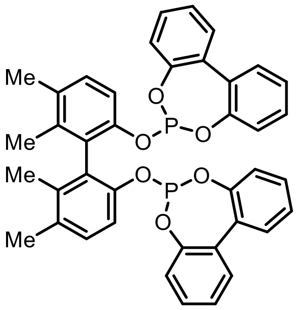

General Procedure F: 2,2'-Biphenol (600 mg, 3.22 mmol, 3 equiv.) and PCl_3 (2.80 mL, 32.2 mmol, 30 equiv.) were employed at 85 °C for 2 h. Following formation of the chlorophosphite, 5,5',6,6'-tetramethyl-[1,1'-biphenyl]-2,2'-diol (259 mg, 1.07 mmol, 1 equiv.), DMAP (25.6 mg, 0.21 mmol, 0.20 equiv.), Et_3N (0.61 mL, 4.49 mmol, 4.20 equiv.) and THF (10 mL) were employed. Purification of the crude material by FCC (hexane/EtOAc 5%) on neutral alumina afforded the title compound (550 mg, 77%) as a colorless powder. m.p. 95 – 98 °C (pentane); ν_{max}/cm^{-1}: 3063 (m), 2923 (m), 1475 (s), 1434 (s), 1202 (s); ^{1}H NMR (500 MHz, C_6D_6): δ 7.26 (2H, d, *J* = 8.0 Hz, 2 × Ar-CH), 7.14 – 7.10 (4H, m, 4 × Ar-CH), 7.03 (2H, d, *J* = 8.0 Hz, 2 × Ar-CH), 7.01 – 6.95 (6H, m, 6 × Ar-CH), 6.92 – 6.87 (4H, m, 4 × Ar-CH), 6.80 (2H, d, *J* = 8.0 Hz,2 × Ar-CH), 2.05 (6H, s, 2 × CH_3), 1.89 (6H, s, 2 × CH_3); ^{13}C NMR (126 MHz, C_6D_6): δ 149.9 (Ar-C), 148.6 (Ar-C), 138.2 (Ar-C), 133.0 (Ar-C), 131.8 (Ar-C), 130.1 (Ar-CH), 129.9 (Ar-CH), 129.1 (Ar-CH), 128.6 (Ar-C), 125.3 (Ar-CH), 122.7 (Ar-CH), 117.8 (Ar-CH), 20.1 (CH_3), 17.1 (CH_3); ^{31}P NMR (162 MHz, $CDCl_3$): δ 144.9; HRMS (MALDI$^+$): Calculated for $C_{40}H_{32}O_6P_2$: 617.1747. Found [M+H]$^+$: 671.1759.

(L-8): 2,2'-Bis(dibenzo[*d,f*][1,3,2]dioxaphosphepin-6-yloxy)-1,1'-biphenyl

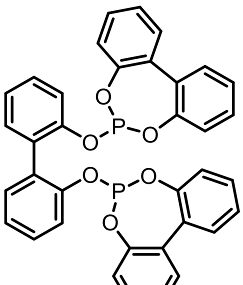

General Procedure F: 2,2'-Biphenol (600 mg, 3.22 mmol, 3 equiv.) and PCl_3 (2.80 mL, 32.2 mmol, 30 equiv.) were employed at 85 °C for 2 h. Following formation of the chlorophosphite, 2,2'-biphenol (200 mg, 1.07 mmol, 1 equiv.), DMAP (25.6 mg, 0.21 mmol, 0.20 equiv.), Et_3N (0.61 mL, 4.49 mmol, 4.20 equiv.) and THF (10 mL) were employed. Purification of the residue by FCC (hexane/EtOAc 10%)

on neutral alumina afforded the title compound (413 mg, 63%) as a colorless powder. m.p. 150–151°C (pentane); ν_{max}/cm^{-1}: 3062 (m), 1498 (s), 1474 (s), 1434 (s), 1194 (s); ^{1}H NMR (500 MHz, C_6D_6): δ 7.34 (2H, dd, *J* = 8.0, 1.0 Hz, 2 × Ar-CH), 7.22 (2H, dd, *J* = 7.5, 2.0 Hz, 2 × Ar-CH), 7.13 – 7.10 (4H, m, 4 × Ar-CH), 7.05 (2H, td, *J* = 8.0, 2.0 Hz, 2 × Ar-CH), 6.96 – 6.91 (8H, m, 8 × Ar-CH), 6.91 – 6.85 (6H, m, 6 × Ar-CH); ^{13}C NMR (126 MHz, C_6D_6): δ 150.3 (Ar-C), 149.8 (Ar-C), 132.7 (Ar-CH), 131.7 (Ar-C), 130.9 (Ar-C), 130.1 (Ar-CH), 129.3 (*two signals,* 2 × Ar-CH), 125.3 (Ar-CH), 124.4 (Ar-CH), 122.6 (Ar-CH), 121.0 (Ar-CH); ^{31}P NMR (162 MHz, $CDCl_3$): δ 144.4; HRMS (MALDI^{+}): Calculated for $C_{36}H_{24}NaO_6P_2$: 637.0940. Found $[M+Na]^{+}$: 637.0948.

Synthesis of Substrates

Heteroaromatic Substrates

Benzamides **1a-b** and **1e** were purchased from commercial sources. **1c-d** and **1f-g** were prepared according to General Procedure B. *The spectroscopic properties of these compounds were consistent with the data available in the literature.*[5-8]

(3a): *N,N*-Diisopropylfuran-3-carboxamide

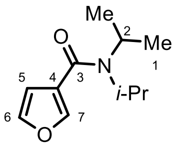

General Procedure A: The reaction was carried out with 3-furoic acid. Purification of the residue by FCC (hexane/EtOAc 30–50%) afforded the title compound (1.10 g, 72%) as an off-white solid. ν_{max}/cm^{-1}: 2971 (m), 2932 (m), 1618 (s), 1437 (s); ^{1}H NMR (400 MHz, $CDCl_3$): δ 7.64 – 7.59 (1H, m, C**7**), 7.42 – 7.33 (1H, m, C**6**), 6.54 – 6.46 (1H, m, C**5**), 4.35 – 3.33 (2H, m, C**2**), 1.34 (12H, s, C**1**); ^{13}C NMR (101 MHz, $CDCl_3$): δ 164.4 (C**3**), 142.7 (C**6**), 142.1 (C**7**), 123.5 (C**4**), 109.9 (C**5**), 47.5 (C**2**), 21.0 (C**1**); HRMS: (ESI^{+}) calculated for $C_{11}H_{18}NO_2$ 196.1332. Found $[M+H]^{+}$ 196.1338; m.p. 43–45 °C (hexane/EtOAc) (Lit.[3] 44–45 °C, hexane).

(3b): *N,N*-Diethylfuran-3-carboxamide

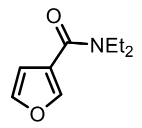

General Procedure A: The reaction was carried out with 3-furoic acid. Purification of the residue by FCC (petroleum ether/EtOAc 40%) afforded the title compound (913 mg, 70%) as a light brown oil. ^{1}H NMR (400 MHz, $CDCl_3$): δ 7.71 – 7.69 (1H, m), 7.41 (1H, t, *J* = 1.0 Hz), 6.58 (1H, dd, *J* = 2.0, 1.0 Hz), 3.48 (4H, q, *J* = 7.0 Hz), 1.21 (6H, t, *J* = 7.0 Hz); ^{13}C NMR (101 MHz, $CDCl_3$): δ 164.1, 142.7, 142.6, 121.8, 110.14, 42.8, 40.1, 14.5, 12.9. *All spectroscopic data agrees with that reported in literature.*[9]

***N,N*-Diisopropyl-1*H*-pyrrole-3-carboxamide**

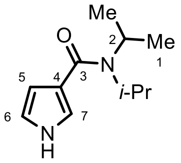

General Procedure A: Purification of the residue by FCC (hexane/EtOAc 80%) afforded the title compound (1.23 g, 71%) as colorless needles. ν_{max}/cm^{-1}: 3178 (m), 2957 (m), 2940 (m), 1582 (s); ^{1}H NMR (400 MHz, $CDCl_3$): δ 10.02 (1H, br. s, NH), 7.02 – 6.79 (1H, m, C**6**-H), 6.69 – 6.48 (1H, m, C**7**-H), 6.32 – 6.08 (1H, m, C**5**-H), 4.74 – 3.09 (2H, m, C**2**-H), 1.35 (12H, s, C**1**-H_3); ^{13}C NMR (101 MHz, $CDCl_3$): δ 168.2 (C**3**), 120.8 (C**4**), 120.4 (C**6**), 117.9 (C**7**), 107.7 (C**5**), 47.6 (C**2**), 21.2 (C**1**); HRMS: (ESI$^+$) calculated for $C_{11}H_{19}N_2O$ 195.1492. Found $[M+H]^+$ 195.1489; m.p. 78–80 °C ($CDCl_3$).

(5): *N,N*-Diisopropyl-1-methyl-1*H*-pyrrole-3-carboxamide

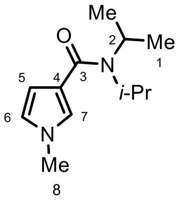

To a flame-dried flask was added NaH (125 mg, 3.12 mmol, 60% in oil) and dry THF (4.40 mL) under nitrogen. To the stirred solution was added *N,N*-diisopropyl-1*H*-pyrrole-3-carboxamide (500 mg, 2.60 mmol) portion-wise and the resulting solution was stirred for 1 h. Methyl iodide (0.453 mL, 7.28 mmol) was added dropwise and the solution was stirred for 3 h, before being quenched with H_2O (10 mL) and extracted with Et_2O (3 ×10 mL). The organic extracts were combined, dried over Na_2SO_4 and concentrated *in vacuo*. Purification of the residue by FCC (CH_2Cl_2/EtOAc 20–30%) afforded the title compound (453 mg, 84%) as white needles. ν_{max}/cm^{-1}: 2954 (m), 2931 (m), 1603 (s), 1286 (s); ^{1}H NMR (400 MHz, $CDCl_3$): δ 6.92 (1H, t, J = 2.0 Hz, C**7**-H), 6.50 (1H, t, J = 2.5 Hz, C**6**-H), 6.24 (1H, dd, J = 2.5, 2.0 Hz, C**5**-H), 4.24 – 3.73 (2H, m, C**2**-H), 3.63 (3H, s, C**8**-H_3), 1.34 (12H, d, J = 6.5 Hz, C**1**-H_3); ^{13}C NMR (101 MHz, $CDCl_3$): δ 166.8 (C**3**), 123.9 (C**7**), 121.4 (C**4**), 121.2 (C**6**), 108.4 (C**5**), 48.1 (C**2**), 36.2 (C**8**), 21.1 (C**1**); HRMS: (ESI$^+$) calculated for $C_{12}H_{21}N_2O$ 209.1648. Found $[M+H]^+$ 209.1649; m.p. 84–86 °C ($CDCl_3$).

***N,N*-Diisopropyl-1*H*-pyrrole-1-carboxamide**

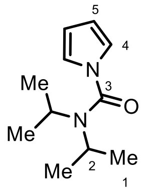

The title compound was prepared following a modified literature procedure.[10] A flame-dried round-bottom flask was charged with NaH (60% in mineral oil, 715 mg, 17.9 mmol), suspended in THF (15 mL) under nitrogen. The suspension was cooled to 0 °C and a solution of pyrrole (1.03 mL, 14.9 mmol) in THF (18 mL) was added dropwise over 10 minutes. The solution was stirred at 0 °C for 1 h, followed by dropwise addition of *N*,*N*-diisopropylcarbamoyl chloride (2.68 g, 16.4 mmol) in THF (20 mL) over 10 minutes. The solution was then warmed to ambient temperature and stirred overnight. The reaction was quenched by the addition of saturated aqueous ammonium chloride solution (50 mL) and extracted with CH_2Cl_2 (3 × 50 mL). The organic extracts were combined, washed with brine (50 mL), dried over Na_2SO_4, filtered, and concentrated in *vacuo*. The crude material was purified by FCC (hexane/EtOAc 20%) to afford the title compound (2.46 g, quantitative) as colorless needles. ν_{max}/cm^{-1}: 2971 (m), 2934 (m), 1679 (s), 1430 (s), 1332 (s), 1318 (s); ^{1}H NMR (400 MHz, $CDCl_3$): δ 7.00 – 6.95 (2H, m, C**4**-H), 6.24 – 6.19 (2H, m, C**5**-H), 3.83 (2H, hept, *J* = 6.5 Hz, C**2**-H), 1.37 (12H, d, *J* = 6.5 Hz, C**1**-H); ^{13}C NMR (101 MHz, $CDCl_3$): δ 152.8 (C**3**), 120.2 (C**4**), 110.1 (C**5**), 48.7 (C**2**), 21.2 (C**1**); HRMS: (ESI$^+$) calculated for $C_{11}H_{19}N_2O$ 195.1492. Found $[M+H]^+$ 195.1492; m.p. 73–75 °C ($CDCl_3$).

(7): *N,N*-Diisopropylthiophene-2-carboxamide

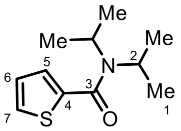

To a solution of thiophene-2-carboxylic acid (1.92 g, 15.0 mmol) in CH_2Cl_2 (15 mL) at 0 °C was added 1 drop of DMF. Oxalyl chloride (1.42 mL, 16.8 mmol) was added dropwise *via* syringe over 10 min. The solution was stirred at 0 °C for 2 h before being concentrated *in vacuo*. CH_2Cl_2 (15 mL) was added to the oily residue and the solution was cooled to 0 °C, before diisopropylamine (4.21 mL, 30.0 mmol) was added dropwise *via* syringe over 10 min. The solution was warmed to ambient temperature and stirred for 16 h. CH_2Cl_2 (20 mL) and water (20 mL) were added, and the layers were separated. The aqueous layer was extracted with CH_2Cl_2 (2 × 20 mL) and the combined organic layers were washed with brine (50 mL), dried over Na_2SO_4 and concentrated *in vacuo.* Purification of the residue by FCC (hexane/EtOAc 10%) afforded the title compound (3.12 g, 98%) as a colorless crystalline solid. m.p. 60–62 °C (hexane/CH_2Cl_2); ^{1}H NMR (400 MHz, $CDCl_3$): δ 7.36 (1H, dd, *J* = 5.0, 1.0 Hz, C**5**-H), 7.19 (1H, dd, *J* = 3.5, 1.0 Hz, C**7**-H), 7.00 (1H, dd, *J* = 5.0, 3.5 Hz, C**6**-H), 4.08 – 3.85 (2H, m, 2 × C**2**-H), 1.37 (12H, d, *J* = 6.5 Hz, 4 × C**1**-H_3); ^{13}C NMR (101 MHz, $CDCl_3$): δ 164.1 (C**3**), 140.1 (C**4**), 127.3 (C**5**), 126.8 (C**7**), 126.5 (C**6**), 49.0 (C**2**), 21.0 (C**1**); *The spectroscopic properties of this compound were consistent with the data available in the literature*.[11]

Synthesis of Alkene Substrates

a-methylstyrene, (S)-(-)-limonene, 2-methyl-1-pentene, 4-fluoro-a-methyl-styrene were purchased from commercial sources (Sigma Aldrich, Alfa Aesar, Acros). But-1-en-2-ylbenzene and pent-1-en-2-ylbenzene were prepared according to literature procedures.[12,13]

1-(Prop-1-en-2-yl)-3-(trifluoromethyl)benzene

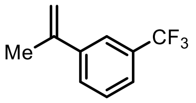

General Procedure C: Purification of the residue by FCC (petroleum ether) afforded the title compound (405 mg, 41 %) as a colorless oil; ^{1}H NMR (400 MHz, $CDCl_3$): δ 7.70 (1H, s) 7.63 (1H, d, J = 4.0 Hz), 7.52 (1H, d, J = 4.0 Hz), 7.43 (1H, t, J = 8.0), 5.43 (1H, s), 5.19 (1H, s), 2.18 (3H, s); ^{13}C NMR (126 MHz, $CDCl_3$): δ 142.1, 142.0, 130.6 (q, J = 32.0 Hz), 128.7 – 128.8 (m), 128.7, 124.2 (q, J = 273.5 Hz), 124.0 (q, J = 4.0 Hz), 122.3 (q, J = 4.0 Hz), 114.0, 21.7; ^{19}F NMR (377 MHz, $CDCl_3$): δ -62.56. *The spectroscopic properties for this compound were consistent with the data available in the literature.*[14]

1-Methoxy-3-(prop-1-en-2-yl)benzene

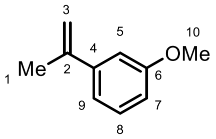

General Procedure C: Purification of the residue by FCC (hexane/EtOAc 5%) afforded the title compound (1.25 g, 85%) as a colorless oil. ν_{max}/cm^{-1}: 2943 (m), 2834 (m), 1576 (s), 1231 (s); ^{1}H NMR (400 MHz, $CDCl_3$): δ 7.30 – 7.22 (1H, m, C**8**-H), 7.07 (1H, ddd J = 7.5, 2.0, 1.0 Hz, C**7**-H), 7.02 – 6.99 (1H, m, C**5**-H), 6.83 (1H, ddd, J = 8.5, 2.0, 1.0 Hz, C**9**-H), 5.38- 5.36 (1H, m, C**3**-H_2), 5.10 – 5.08 (1H, m, C**3**-H_2), 3.83 (3H, s, C**10**-H_3), 2.16 – 2.14 (1H, m, C**1**-H_3); ^{13}C NMR (101 MHz, $CDCl_3$): δ 159.7 (C**6**), 143.4 (C**2**), 143.0 (C**4**), 129.3 (C**8**), 118.3 (C**7**), 112.8 (C**3**), 112.8 (C**9**), 111.7 (C**5**), 55.4 (C**10**), 22.0 (C**1**); HRMS: (APCI) calculated for $C_{10}H_{12}O$ 149.0961. Found [M] 149.0959.

1-Isopropenyl-4-methoxybenzene

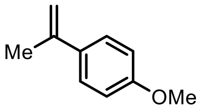

General Procedure C: Purification of the residue by FCC (petroleum ether/EtOAc 5%) afforded the title compound (481 mg, 48%) as white needles. ^{1}H NMR (400 MHz, $CDCl_3$): δ 7.42 (2H, d, J = 8.0 Hz), 6.87 (2H, d, J = 8.0 Hz), 5.29 (1H, s), 4.99 (1H, s), 3.82 (3H, s), 2.13 (3H, s); ^{13}C NMR (101 MHz, $CDCl_3$): δ 159.0, 142.6, 133.8, 126.6, 113.5, 110.6, 55.33, 21.9; m.p. = 33–34 °C ($CDCl_3$). *The spectroscopic properties for this compound were consistent with the data available in the literature.*[15]

1-Chloro-3-(prop-1-en-2-yl)benzene

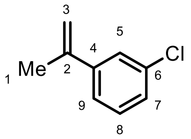

General Procedure C: Purification of the residue by FCC (hexane/EtOAc 10%) afforded the title compound (1.20 g, 79%) as a colorless oil. ν_{max}/cm^{-1}: 3088 (m), 2947 (m), 1593 (m), 1562 (s); ^{1}H NMR (400 MHz, $CDCl_3$): δ 7.44 – 7.40 (1H, m, C**5**-H), 7.35 – 7.29 (1H, m, C**9**-H), 7.24 – 7.22 (2H, m, C**7**-H, C**8**-H), 5.38 – 5.34 (1H, m, C**3**-H_2), 5.14 – 5.08 (1H, m, C**3**-H_2), 2.15 – 2.08 (3H, m, C**1**-H_3); ^{13}C NMR (101 MHz, $CDCl_3$): δ 143.3 (C**4**), 142.3 (C**2**), 134.4 (C**6**), 129.6 (C**8**), 127.5 (C**7**), 125.9 (C**5**), 123.8 (C**9**), 113.8 (C**3**), 21.8 (C**1**); HRMS: (EI) calculated for C_9H_9Cl 215.0387. Found [M] 215.0388.

(3-Methylbut-3-en-1-yl)benzene

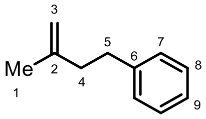

To a suspension of methyltriphenylphosphonium bromide (3.93 g, 11.0 mmol) in anhydrous THF (40 mL) at 0 °C under N_2 was added *n*-BuLi (6.88 mL, 11.0 mmol, 1.6 M in hexanes) dropwise over 10 min. The resulting orange suspension was stirred at 0 °C for 1 h before 4-phenylbutan-2-one (1.50 mL, 10.0 mmol) in THF (5 mL) was added dropwise over 10 min. The suspension was maintained at 0 °C for 1 h before being warmed to ambient temperature over 1 h and quenched with sat. aq. NH_4Cl (20 mL). Et_2O (20 mL) was added, and the layers were separated. The aqueous layer was extracted with Et_2O (2 × 30 mL), and the combined organic portions were washed with brine (50 mL), dried over Na_2SO_4 and concentrated *in vacuo*. Purification of the residue by FCC (hexane/Et_2O 1%) afforded the title compound (1.07 g, 73%) as a colorless oil. ^{1}H NMR (400 MHz, $CDCl_3$): δ 7.36 – 7.25 (2H, m, 2 × C**8**-H), 7.23 – 7.14 (3H, m, 2 × C**7**-H, C**9**-H), 4.75 (2H, d, *J* = 11.0 Hz, C**3**-H_2), 2.87 – 2.69 (2H, m, C**5**-H_2), 2.33 (2H, dd, *J* = 10.0, 6.5 Hz, C**4**-H_2), 1.78 (3H, s, C**1**-H_3); ^{13}C NMR (101 MHz, $CDCl_3$): δ 145.6 (C**2**), 142.4 (C**6**), 128.5, 128.4 (C**7**, C**8**), 125.9 (C**9**), 110.3 (C**3**), 39.7 (C**4**), 34.4 (C**5**), 22.8 (C**1**). *The spectroscopic properties of this compound were consistent with the data available in the literature*.[16]

(1-Cyclobutylvinyl)benzene

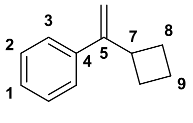

General Procedure C: Purification of the residue by FCC (hexane) afforded the title compound (473 mg, 60%) as a colourless oil; R_f = 0.75 (hexane); ^{1}H NMR (500 MHz, $CDCl_3$) δ 7.44 – 7.40 (m, 2H, C**3**-H), 7.38 – 7.33 (m, 2H, C**2**-H), 7.32 – 7.27 (m, 1H, C**1**-H), 5.39 (d, *J* = 1.5 Hz, 1H, C**6**-H), 5.08 (d, *J* =

1.5 Hz, 1H, C**6**-H’), 3.57 – 3.46 (m, 1H, C**7**-H), 2.33 – 2.19 (m, 2H, C**8**-H), 2.07 – 1.96 (m, 3H, C**8**-H’, C**9**-H_2), 1.89 – 1.77 (m, 1H, C**9**-H); ^{13}C NMR (126 MHz, $CDCl_3$) δ 152.2 (C**5**), 140.9 (C**4**), 128.3 (C**2**), 127.4 (C**1**), 126.2 (C**3**), 109.9 (C**6**), 39.7 (C**7**), 28.6 (C**8**), 17.9 (C**9**). *The spectroscopic properties for this compound were consistent with the data available in the literature*.[17]

(((3-Methylbut-3-en-1-yl)oxy)methyl)benzene

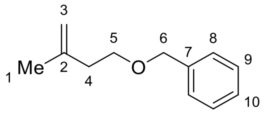

(((3-Methylbut-3-en-1-yl)oxy)methyl)benzene was synthesised from 2-methyl-1-buten-4-ol (5 mmol) using a known literature procedure[18] to afford the title compound as a colourless liquid (500 mg, 57%); R_f = 0.69 (hexane/EtOAc 10%); ^{1}H NMR (500 MHz, $CDCl_3$) δ 7.37 – 7.31 (m, 4H, C**8**-H_2, C**9**-H_2), 7.30 – 7.27 (m, 1H, C**10**-H_1), 4.79 (br, 1H, C**3**-H_1), 4.75 (br, 1H, C**3**-H_1), 4.53 (s, 2H, C**6**-H_2), 3.59 (t, *J* = 7.0 Hz, 2H, C**5**-H_2), 2.35 (t, *J* = 7.0 Hz, 2H, C**4**-H_2), 1.75 (s, 3H, C**1**-H_3); ^{13}C NMR (126 MHz, $CDCl_3$) δ 143.0 (C**11**), 138.6 (C**7**), 128.5 (C**8**), 127.8 (C**9**), 127.7 (C**10**), 111.6 (C**3**), 73.1 (C**6**), 68.9 (C**5**), 38.0 (C**4**), 22.8 (C**1**). *The spectroscopic properties for this compound were consistent with the data available in the literature*.[18]

(8*R*,9*S*,13*S*,14*S*)-13-Methyl-3-(prop-1-en-2-yl)-6,7,8,9,11,12,13,14,15,16-decahydro-17*H*-cyclopenta[*a*]phenanthren-17-one

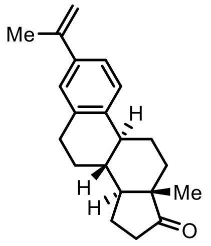

Prepared according to the literature procedure.[19] *The spectroscopic properties of this compound were consistent with the data available in the literature.*

(9): 4-(Prop-1-en-2-yl)-1,1'-biphenyl

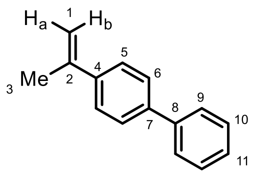

General Procedure C: Purification of the residue by FCC (hexane/EtOAc 5%) afforded the title compound (3.48 g, 88%) as a colorless solid. m.p. 118–120 °C (hexane/EtOAc); v_{max}/cm^{-1}: 2974 (m),

2939 (m), 1627 (m), 1423 (s); ^{1}H NMR (400 MHz, $CDCl_3$): δ 7.63 – 7.60 (2H, m, C**9**-H), 7.60 – 7.54 (4H, m, C**5**-H, C**6**-H), 7.49 – 7.42 (2H, m, C**10**-H), 7.39 – 7.31 (1H, m, C**11**-H), 5.44 (1H, dq, *J* = 1.5, 1.0 Hz, C**1**-(H_a)$_2$), 5.12 (1H, p, *J* = 1.5 Hz, C**1**-(H_b)$_2$), 2.20 (3H, dd, *J* = 1.5, 1.0 Hz, C**3**-H_3); ^{13}C NMR (101 MHz, $CDCl_3$): δ 142.9 (C**2**), 140.9 (C**8**), 140.3 (C**7**), 140.3 (C**4**), 128.9 (C**10**), 127.4 (C**11**), 127.1 (C**9**), 127.1 (C**6**), 126.0 (C**5**), 112.6 (C**1**), 22.0 (C**3**); HRMS: (EI^+) calculated for $C_{15}H_{14}$ 194.1090. Found $[M]^+$ 194.1088.

Alternative synthesis of alkene 9:

2-([1,1'-Biphenyl]-4-yl)propan-2-ol

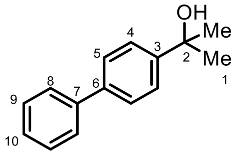

To a solution of methyl 4-phenylbenzoate (1.50 g, 7.06 mmol) in anhydrous THF (20 mL) under N_2 at 0 °C was added MeMgBr (7.06 mL, 21.2 mmol, 3.0 M in Et_2O) dropwise *via* syringe over 10 min. The solution was stirred at 0 °C for 1 h before being warmed to ambient temperature and stirred for 16 h. Upon completion of the reaction, sat. aq. NH_4Cl (20 mL) and Et_2O (20 mL) were added, and the layers separated. The aqueous layer was extracted with Et_2O (20 mL), and the combined organic portions were washed with brine (50 mL), dried over Na_2SO_4 and concentrated *in vacuo*. Purification of the residue by FCC (hexane/EtOAc 15%) afforded the title compound (1.34 g, 89%) as a colorless solid. m.p. 87–90 °C (hexane/CH_2Cl_2); ν_{max}/cm^{-1}: 3333 (m), 2968 (s), 1486 (s), 1402 (s); ^{1}H NMR (400 MHz, $CDCl_3$): δ 7.62 – 7.57 (6H, m, 2 × C**4**-H, 2 × C**5**-H, 2 × C**8**-H), 7.44 (2H, dd, *J* = 8.0, 7.0 Hz, 2 × C**9**-H), 7.35 (1H, m, C**10**-H), 1.68 (1H, *br.* s, OH), 1.64 (6H, s, 2 × C**1**-H_3); ^{13}C NMR (101 MHz, $CDCl_3$): δ 148.3 (C**3**), 141.0 (C**7**), 139.8 (C**6**), 128.9 (C**9**), 127.4 (C**10**), 127.2, 127.1 (C**5**, C**8**), 125.0 (C**4**), 72.6 (C**2**), 31.9 (C**1**); HRMS (ESI^+): Calculated for $C_{15}H_{16}NaO$: 235.1093. Found $[M+Na]^+$: 235.1086.

(9): 4-(Prop-1-en-2-yl)-1,1'-biphenyl

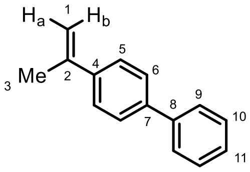

To a solution of 2-([1,1'-biphenyl]-4-yl)propan-2-ol (1.00 g, 4.71 mmol) in CH_2Cl_2 (20 mL) at 0 °C was added DMAP (57.4 mg, 0.47 mmol) and Et_3N (0.72 mL, 5.18 mmol). Methanesulfonyl chloride (0.55 mL, 7.07 mmol) was added dropwise *via* syringe over 5 min. The solution was maintained at 0 °C for 1 h before being warmed to ambient temperature and stirred for 16 h. Upon completion of the reaction, water (20 mL) was added, and the layers were separated. The aqueous layer was extracted with CH_2Cl_2

(2 × 20 mL) and the combined organic portions were washed with water (50 mL), brine (50 mL), dried over Na_2SO_4 and concentrated *in vacuo*. Purification of the residue by FCC (hexane/Et_2O 1%) afforded the title compound (454 mg, 50%) as a colorless solid. *The analytical data for this compound was the same as described earlier.*

Reaction Scope

(2a): 2-(2-([1,1'-Biphenyl]-4-yl)propan-2-yl)-*N,N*-diethylbenzamide

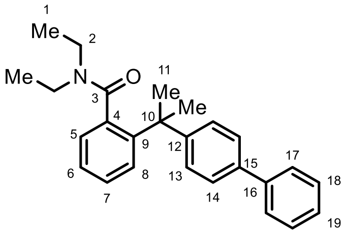

General Procedure E: The reaction was carried out with styrene derivative **9** (400 mol%) and was run for 72 h. Purification of the residue by FCC (hexane/EtOAc 0–20%) afforded the title compound (27.7 mg, 73%) as a yellow wax. ν_{max}/cm^{-1}: 2970 (m), 2930 (m), 1628 (s), 1487 (s), 1423 (s); ^{1}H NMR (400 MHz, $CDCl_3$): δ 7.57 (2H, dd, *J* = 8.0, 1.5 Hz, C**17**-H), 7.52 (1H, d, *J* = 8.5 Hz, C**8**-H), 7.49 (2H, d, *J* = 8.5 Hz, C**18**-H), 7.43 (2H, dd, *J* = 8.5, 7.0 Hz, C**14**-H), 7.36 – 7.30 (4H, m, C**7**-H, C**13**-H, C**19**-H), 7.21 (1H, ddd, *J* = 7.5, 7.5, 1.5 Hz, C**6**-H), 7.05 (1H, dd, *J* = 7.5, 1.5 Hz, C**5**-H), 3.17 (1H, dq, *J* = 14.0, 7.0 Hz, C**2**-H_2), 3.06 (1H, dq, *J* = 14.0, 7.0 Hz, C**2**-H_2), 2.88 (1H, dq, *J* = 14.0, 7.0 Hz, C**2**-H_2), 2.37 (1H, dq, *J* = 14.0, 7.0 Hz, C**2**-H_2), 1.89 (3H, s, C**11**-H_3), 1.73 (3H, s, C**11**-H_3), 1.01 – 0.98 (3H, m, C**1**-H_3), 0.97 – 0.94 (3H, m, C**1**-H_3); ^{13}C NMR (101 MHz, $CDCl_3$): δ 171.6 (C**3**), 148.8 (C**12**), 146.7 (C**9**), 141.1 (C**16**), 138.5 (C**15**), 136.6 (C**4**), 128.9 (C**14**), 128.6 (C**7**), 128.2 (C**8**), 127.8 (C**13**), 127.6 (C**5**), 127.2 (C**19**), 127.0 (C**17**), 126.6 (C**18**), 125.9 (C**6**), 44.2 (C**2**), 44.1 (C**10**), 39.3 (C**2**), 33.1 (C**11**), 30.0 (C**11**), 13.7 (C**1**), 13.0 (C**1**); HRMS: (ESI$^+$) calculated for $C_{26}H_{30}NO$ 372.2322. Found $[M+H]^+$ 372.2315.

(2b): 2-(2-([1,1'-Biphenyl]-4-yl)propan-2-yl)-*N,N*-dimethylbenzamide

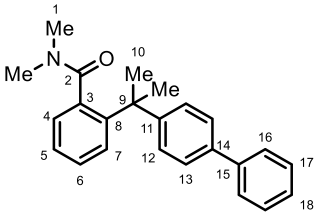

General Procedure E: The reaction was carried out with styrene derivative **9** (400 mol%) and was run for 72 h. Purification of the residue by FCC (hexane/EtOAc 10–30%) afforded the title compound (16.4 mg, 48%) as a colorless wax. ν_{max}/cm^{-1}: 2961 (m), 2925 (m), 1633 (s), 1487 (s); ^{1}H NMR (400 MHz, $CDCl_3$): δ 7.63 (1H, dd, *J* = 7.5, 1.0 Hz, C**7**-H), 7.61 – 7.54 (2H, m, C**16**-H), 7.52 – 7.48 (2H, m, C**13**-

H), 7.47 – 7.39 (2H, m, C**17**-H), 7.41 – 7.29 (4H, m, C**6**-H, C**12**-H, C**18**-H), 7.20 (1H, ddd, *J* = 7.5, 1.5, 1.5 Hz, C**5**-H), 6.98 (1H, dd, *J* = 7.5, 1.5 Hz, C**4**-H), 2.57 (3H, s, C**1**-H_3), 2.27 (3H, s, C**1**-H_3), 1.91 (3H, s, C**10**-H_3), 1.74 (3H, s, C**10**-H_3); ^{13}C NMR (101 MHz, $CDCl_3$): δ 171.4 (C**2**), 148.2 (C**11**), 147.0 (C**8**), 141.1 (C**15**), 138.5 (C**14**), 136.3 (C**3**), 129.0 (C**17**), 128.7 (C**6**), 128.1 (C**12**), 128.0 (C**4**), 127.4 (C**7**), 127.3 (C**18**), 127.0 (C**16**), 126.4 (C**13**), 126.0 (C**5**), 43.7 (C**9**), 39.4 (C**1**), 34.2 (C**1**), 33.1 (C**10**), 29.8 (C**10**); HRMS: (ESI$^+$) calculated for $C_{24}H_{25}NO$ 344.2009. Found $[M+H]^+$ 344.2011.

(2c): (2-(2-([1,1'-Biphenyl]-4-yl)propan-2-yl)phenyl)(pyrrolidin-1-yl)methanone

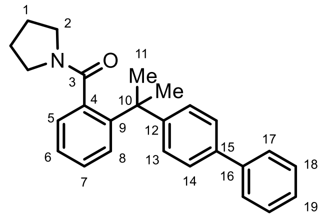

General Procedure E: The reaction was carried out with styrene derivative **9** (400 mol%) and was run for 72 h. Purification of the residue by FCC (hexane/EtOAc 0–40%) afforded the title compound (18.9 mg, 51%) as a colorless wax. v_{max}/cm^{-1}: 2969 (m), 2871 (m), 1621 (s), 1417 (s); ^{1}H NMR (400 MHz, $CDCl_3$): δ 7.66 (1H, d, *J* = 7.5 Hz, C**8**-H), 7.58 – 7.51 (2H, m, C**17**-H), 7.47 (2H, d, *J* = 8.0 Hz, C**14**-H), 7.45 – 7.41 (2H, m, C**18**-H), 7.42 – 7.28 (4H, m, C**7**-H, C**13**-H, C**19**-H), 7.27 – 7.16 (1H, m, C**6**-H), 7.06 (1H, dd, *J* = 7.5, 1.5 Hz, C**5**-H), 3.37 – 3.29 (1H, m, C**2**-H_2), 2.97 – 2.87 (1H, m, C**2**-H_2), 2.86 – 2.75 (1H, m, C**2**-H_2), 2.52 – 2.40 (1H, m, C**2**-H_2), 1.96 (3H, s, C**11**-H_3), 1.73 (3H, s, C**11**-H_3), 1.62 – 1.38 (3H, m, C**1**-H_2), 1.29 – 1.15 (1H, m, C**1**-H_2); ^{13}C NMR (101 MHz, $CDCl_3$): δ 169.9 (C**3**), 148.2 (C**12**), 146.9 (C**9**), 141.1 (C**16**), 138.5 (C**15**), 137.5 (C**4**), 129.0 (C**18**), 128.8 (C**7**), 128.2 (C**5**), 128.0 (C**13**), 127.3 (C**19**), 127.1 (C**8**), 127.0 (**17**), 126.4 (C**14**), 126.2 (C**6**), 48.2 (C**2**), 45.0 (C**2**), 43.6 (C**10**), 33.4 (C**11**), 29.9 (C**11**), 25.4 (C**1**), 24.2 (C**1**); HRMS: (ESI$^+$) calculated for $C_{26}H_{27}NO$ 370.2165. Found $[M+H]^+$ 370.2168.

(2d): (2-(2-([1,1'-Biphenyl]-4-yl)propan-2-yl)phenyl)(piperidin-1-yl)methanone

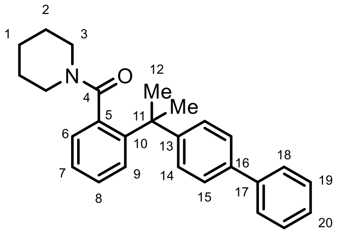

General Procedure E: The reaction was carried out with styrene derivative **9** (400 mol%) and was run for 72 h. Purification of the residue by FCC (hexane/EtOAc 0–20%) afforded the title compound (12.0 mg, 31%) as an orange oil. v_{max}/cm^{-1}: 2930 (m), 2852 (m), 1627 (s), 1430 (s); ^{1}H NMR (400 MHz, $CDCl_3$): δ 7.60 – 7.55 (2H, m, C**18**-H), 7.55 – 7.51 (1H, m, C**9**-H), 7.51 – 7.47 (2H, m, C**15**-H), 7.47 – 7.39 (2H, m, C**19**-H), 7.39 – 7.30 (4H, m, C**8**-H, C**14**-H, C**20**-H), 7.23 – 7.16 (1H, m, C**7**-H), 7.03 (1H,

dd, J = 7.5, 1.5 Hz, C**6**-H), 4.18 – 4.02 (1H, m, C**3**-H_2), 3.08 – 2.90 (1H, m, C**3**-H_2), 2.43 – 2.27 (1H, m, C**3**-H_2), 2.28 – 2.13 (1H, m, C**3**-H_2), 1.88 (3H, s, C**12**-H_3), 1.74 (3H, s, C**12**-H_3), 1.71 – 1.60 (1H, m, C**2**-H_2), 1.54 – 1.35 (2H, m, C**2**-H_2), 1.35 – 1.25 (3H, m, C**1**-H_2, C**2**-H_2); ^{13}C NMR (101 MHz, $CDCl_3$): δ 170.1 (C**4**), 148.6 (C**13**), 146.8 (C**10**), 141.2 (C**17**), 138.5 (C**16**), 136.3 (C**5**), 128.9 (C**19**), 128.6 (C**8**), 128.0 (C**9**), 128.0 (C**14**), 127.5 (C**6**), 127.2 (C**20**), 127.1 (C**18**), 126.6 (C**15**), 126.0 (C**7**), 48.5 (C**3**), 43.9 (C**11**), 41.9 (C**3**), 33.0 (C**12**), 30.0 (C**12**), 25.8 (C**1**), 25.5 (C**2**), 24.7 (C**2**); HRMS: (ESI$^+$) calculated for $C_{27}H_{29}NO$ 384.2322. Found $[M+H]^+$ 384.2316.

(2f): *N,N*-Diethyl-2-(2-phenylpropan-2-yl)benzamide

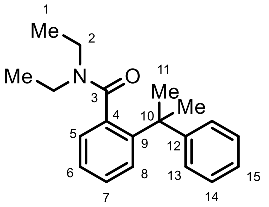

General Procedure E: The reaction was carried out with α-methylstyrene (400 mol%) and was run for 72 h. Purification of the residue by FCC (hexane/EtOAc 20%) afforded the title compound (28.5 mg, 67%) as an orange oil. ν_{max}/cm^{-1}: 2974 (m), 2939 (m), 1627 (m), 1423 (s); ^{1}H NMR (400 MHz, $CDCl_3$): δ 7.47 – 7.41 (1H, m, C**8**-H), 7.34 – 7.27 (1H, m, C**7**-H), 7.26 – 7.15 (4H, m, C**13**-H, C**14**-H), 7.18 (1H, ddd, J = 7.5, 1.0, 1.0 Hz, C**6**-H), 7.16 – 7.10 (1H, m, C**15**-H), 7.03 (1H, dd, J = 7.5, 1.0 Hz, C**5**-H), 3.10 – 2.97 (2H, m, C**2**-H_2), 2.86 (1H, dq, J = 14.5, 7.0 Hz, C**2**-H_2), 2.36 (1H, dq, J = 14.5, 7.0 Hz, C**2**-H_2), 1.83 (3H, s, C**11**-H_3), 1.69 (3H, s, C**11**-H_3), 1.02 (3H, t, J = 7.0 Hz, C**1**-H_3), 0.96 (3H, t, J = 7.0 Hz, C**1**-H_3); ^{13}C NMR (101 MHz, $CDCl_3$): δ 171.6 (C**3**), 149.7 (C**12**), 146.6 (C**9**), 136.6 (C**4**), 128.5 (C**8**), 128.5 (C**7**), 128.0 (C**14**), 127.5 (C**5**), 127.2 (C**13**), 125.8 (C**6**), 125.7 (C**15**), 44.3 (C**10**), 44.0 (C**2**), 39.3 (C**2**), 33.0 (C**11**), 30.0 (C**11**), 13.6 (C**1**), 12.9 (C**1**); HRMS: (ESI$^+$) calculated for $C_{20}H_{25}NONa$ 318.1828. Found $[M+Na]^+$ 318.1835.

(2g): *N,N*-Diethyl-2-(2-(3-methoxyphenyl)propan-2-yl)benzamide

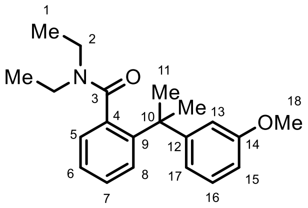

General Procedure E: The reaction was carried out with 1-methoxy-3-(prop-1-en-2-yl)benzene (400 mol%) and was run for 72 h. Purification of the residue by FCC (hexane/EtOAc 0–30%) afforded the title compound (22.9 mg, 70%) as an orange oil. ν_{max}/cm^{-1}: 2967 (m), 2933 (m), 1627 (s), 1424 (s); ^{1}H NMR (400 MHz, $CDCl_3$): δ 7.40 (1H, dd, J = 8.0, 1.5 Hz, C**8**-H), 7.31 – 7.25 (1H, m, C**7**-H), 7.21 – 7.13 (2H, m, C**6**-H, C**16**-H), 7.03 (1H, dd, J = 7.5, 1.5 Hz, C**5**-H), 6.86 – 6.77 (2H, m, C**13**-H, C**17**-H),

6.69 (1H, dd, *J* = 8.0, 3.5 Hz, C**15**-H), 3.76 (3H, s, C**18**-H_3), 3.28 – 3.10 (2H, m, C**2**-H_2), 2.91 (1H, dq, *J* = 14.5, 7.0 Hz, C**2**-H_2), 2.44 (1H, dq, *J* = 14.5, 7.0 Hz, C**2**-H_2), 1.80 (3H, s, C**11**-H_3), 1.68 (3H, s, C**11**-H_3), 1.03 (3H, t, *J* = 7.0 Hz, C**1**-H_3), 0.98 (3H, t, *J* = 7.0 Hz, C**1**-H_3);^{13}C NMR (101 MHz, $CDCl_3$): δ 171.7 (C**3**), 159.3 (C**14**), 151.6 (C**12**), 146.4 (C**9**), 136.5 (C**4**), 128.9 (C**16**), 128.6 (C**8**), 128.5 (C**7**), 127.5 (C**5**), 125.8 (C**6**), 120.0 (ArCH), 113.7 (ArCH), 110.5 (C**15**), 55.2 (C**18**), 44.4 (C**10**), 43.7 (C**2**), 39.1 (C**2**), 32.9 (C**11**), 29.9 (C**11**), 13.6 (C**1**), 12.8 (C**1**); HRMS: (ESI$^+$) calculated for $C_{21}H_{27}NO_2$ 326.2115. Found $[M+H]^+$ 326.2112.

(2h): 2-(2-(3-Chlorophenyl)propan-2-yl)-*N,N*-diethylbenzamide

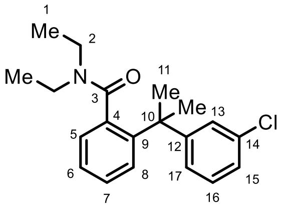

General Procedure E: The reaction was carried out with 1-chloro-3-(prop-1-en-2-yl)benzene (400 mol%), Ir(cod)$_2$]BARF (7.5 mol%) and **L-5** (7.5 mol%) and was run for 72 h. Purification of the residue by FCC (hexane/EtOAc 0–30%) afforded the title compound (21.5 mg, 65%) as a yellow oil. ν_{max}/cm^{-1}: 2959 (m), 2931 (m), 1629 (s), 1423 (s); ^{1}H NMR (400 MHz, $CDCl_3$): δ 7.46 (1H, dd, *J* = 8.0, 1.0 Hz, C**8**-H), 7.32 (1H, ddd, *J* = 8.0, 1.5, 1.0 Hz, C**7**-H), 7.22 (1H, dd, *J* = 7.5, 1.0 Hz, C**6**-H), 7.20 – 7.15 (3H, m, C**13**-H, C**16**-H, C**17**-H), 7.13 (1H, ddd, *J* = 7.5, 4.0, 2.0 Hz, C**15**-H), 7.05 (1H, dd, *J* = 7.5, 1.5 Hz, C**5**-H), 3.23 – 3.05 (2H, m, C**2**-H_2), 2.86 (1H, dq, *J* = 14.5, 7.0 Hz, C**2**-H_2), 2.41 (1H, dq, *J* = 14.5, 7.0 Hz, C**2**-H_2), 1.82 (3H, s, C**11**-H_3), 1.66 (3H, s, C**11**-H_3), 1.06 – 0.95 (6H, m, C**1**-H_3); ^{13}C NMR (101 MHz, $CDCl_3$): δ 171.4 (C**3**), 151.9 (C**12**), 145.9 (C**9**), 136.5 (C**4**), 133.8 (C**14**), 129.3 (C**16**), 128.8 (C**7**), 128.1 (C**8**), 127.7 (C**5**), 127.6 (Ar**C**H), 126.1 (C**6**), 126.0 (C**15**), 125.6 (Ar**C**H), 44.4 (C**10**), 43.9 (C**2**), 39.4 (C**2**), 33.0 (C**11**), 29.9 (C**11**), 13.8 (C**1**), 13.0 (C**1**); HRMS: (ESI$^+$) calculated for $C_{20}H_{24}NOCl$ 330.1619. Found $[M+H]^+$ 330.1612.

(2i): *N,N*-Diethyl-2-(2-(4-fluorophenyl)propan-2-yl)benzamide

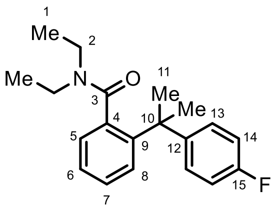

General Procedure E: The reaction was carried out with 4-fluoro-α-methyl-styrene (400 mol%) and was run for 72 h. Purification of the residue by FCC (hexane/EtOAc 0–20%) afforded the title compound (23.1 mg, 74%) as a yellow oil. ν_{max}/cm^{-1}: 2970 (m), 2934 (m), 1627 (s), 1507 (s); ^{1}H NMR (400 MHz,

$CDCl_3$): δ 7.42 (1H, dd, *J* = 8.0, 1.0 Hz, C**8**-H), 7.30 (1H, ddd, *J* = 8.0, 1.5, 1.0 Hz, C**7**-H), 7.24 – 7.16 (3H, m, C**6**-H, C**13**-H), 7.03 (1H, dd, *J* = 7.5, 1.5 Hz, C**5**-H), 6.98 – 6.88 (2H, m, C**14**-H), 3.25 – 3.09 (2H, m, C**2**-H_2), 2.87 (1H, dq, *J* = 14.5, 7.0 Hz, C**2**-H_2), 2.44 (1H, dq, *J* = 14.5, 7.0 Hz, C**2**-H_2), 1.81 (3H, s, C**11**-H_3), 1.67 (3H, s, C**11**-H_3), 1.03 (3H, t, *J* = 7.0 Hz, C**1**-H_3), 0.99 (3H, t, *J* = 7.0 Hz, C**1**-H_3); ^{13}C NMR (101 MHz, $CDCl_3$): δ 171.6 (C**3**), 161.0 (d, *J* = 244.0 Hz, C**15**), 146.5 (C**9**), 145.6 (d, *J* = 3.0 Hz, C**12**), 136.4 (C**4**), 128.77 (d, *J* = 7.5 Hz, C**13**), 128.7 (C**7**), 128.3 (C**8**), 127.6 (C**5**), 126.0 (C**6**), 114.56 (d, *J* = 20.9 Hz, C**14**), 44.1 (C**2**), 43.9 (C**10**), 39.2 (C**2**), 33.0 (C**11**), 30.3 (C**11**), 13.7 (C**1**), 12.9 (C**1**); ^{19}F NMR (377 MHz, $CDCl_3$) δ -118.07 (ddd, *J* = 8.5, 5.5 Hz); HRMS: (ESI$^+$) calculated for $C_{20}H_{24}NOF$ 314.1915. Found $[M+H]^+$ 314.1912.

(2j): *N,N*-diethyl-2-(2-methyl-4-phenylbutan-2-yl)benzamide

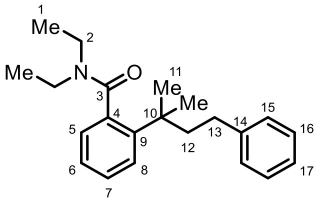

General Procedure E: The reaction was carried out with (3-methylbut-3-en-1-yl)benzene (58.5 mg, 0.40 mmol, 400 mol%) and the reaction was run for 72 h. Purification of the residue by FCC (hexane/EtOAc 10%) afforded the title compound (18.5 mg, 57%) as a colorless oil. ν_{max}/cm^{-1}: 2972 (s), 2932 (s), 1631 (s), 1423 (m); ^{1}H NMR (400 MHz, $CDCl_3$): δ 7.47 (1H, dd, *J* = 8.0, 1.5 Hz, C**8**-H), 7.33 (1H, dd, *J* = 8.0, 1.5 Hz, C**7**-H), 7.25 – 7.18 (3H, m, C**6**-H, 2 × C**15**-H), 7.17 – 7.11 (3H, m, 2 × C**16**-H, C**17**-H), 7.08 (1H, dd, *J* = 7.5, 1.5 Hz, C**5**-H), 3.76 (1H, dq, *J* = 14.0, 7.0 Hz, 1 × C**2**-H), 3.35 (1H, dq, *J* = 14.0, 7.0 Hz, 1 × C**2**-H), 3.20 (1H, dq, *J* = 14.0, 7.0 Hz, 1 × C**2**-H), 3.04 (1H, dq, *J* = 14.0, 7.0 Hz, 1 × C**2**-H), 2.49 – 2.34 (2H, m, C**13**-H_2), 2.22 (1H, m, 1 × C**12**-H), 1.87 (1H, ddd, *J* = 13.5, 10.0, 7.5 Hz, 1 × C**12**-H), 1.45 (3H, s, 1 × C**11**-H_3), 1.40 (3H, s, 1 × C**11**-H_3), 1.23 (3H, t, *J* = 7.0 Hz, 1 × C**1**-H_3), 1.08 (3H, t, *J* = 7.0 Hz, 1 × C**1**-H_3); ^{13}C NMR (101 MHz, $CDCl_3$): δ 173.0 (C**3**), 144.6 (C**9**), 143.2 (C**14**), 136.5 (C**4**), 128.5 (*two signals*), 128.4, 128.3 (C**7**, C**8**, C**15**, C**16**), 127.7 (C**5**), 125.8 (C**6**), 125.6 (C**17**), 46.8 (C**12**), 43.4 (C**2**), 39.9 (C**10**), 38.5 (C**2**), 31.6 (C**13**), 29.3 (C**11**), 29.1 (C**11**), 13.4 (C**1**), 12.1 (C**1**); HRMS (ESI$^+$): Calculated for $C_{22}H_{29}NO$: 324.2322. Found $[M+H]^+$: 324.2314.

(2k): 2-(2-([1,1'-Biphenyl]-4-yl)propan-2-yl)-*N,N*-diethyl-4-methylbenzamide

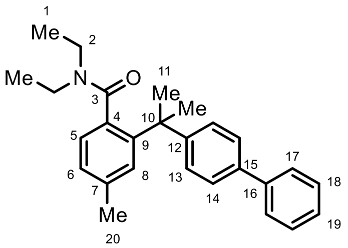

General Procedure E: The reaction was carried out with styrene derivative **9** (400 mol%) and was run for 72 h. Purification of the residue by FCC (hexane/EtOAc 0–20%) afforded the title compound (28.1 mg, 73%) as a yellow oil. ν_{max}/cm^{-1}: 2968 (m), 2934 (m), 1626 (s), 1423 (s); ^{1}H NMR (400 MHz, CDCl$_3$): δ 7.60 – 7.53 (2H, m, C**17**-H), 7.50 – 7.46 (2H, m, C**14**-H), 7.43 (2H, dd, *J* = 7.5, 1.5 Hz, C**18**-H), 7.36 – 7.30 (4H, m, C**8**-H, C**13**-H, C**19**-H), 7.02 (1H, dd, *J* = 7.5, 1.5 Hz, C**6**-H), 6.94 (1H, d, *J* = 7.5 Hz, C**5**-H), 3.20 – 2.97 (2H, m, C**2**-H$_2$), 2.88 (1H, dq, *J* = 14.5, 7.5 Hz, C**2**-H$_2$), 2.37 (3H, s, C**20**-H$_3$), 2.35 – 2.27 (1H, m, C**2**-H$_3$), 1.88 (3H, s, C**11**-H$_3$), 1.72 (3H, s, C**11**-H$_3$), 1.01 – 0.91 (6H, m, C**1**-H$_3$); ^{13}C NMR (101 MHz, CDCl$_3$): δ 171.8 (C**3**), 148.9 (C**12**), 146.6 (C**9**), 141.2 (C**16**), 138.4 (C**15**), 138.2 (C**7**), 133.8 (C**4**), 128.9 (C**18**), 128.8 (C**8**), 127.8 (C**13**), 127.6 (C**5**), 127.2 (C**19**), 127.0 (C**17**), 126.6 (C**14**), 126.5 (C**6**), 44.2 (C**2**), 43.9 (C**10**), 39.3 (C**2**), 33.2 (C**11**), 30.0 (C**11**), 21.7 (C**20**), 13.7 (C**1**), 13.0 (C**1**); HRMS: (ESI$^+$) calculated for $C_{27}H_{31}NO$ 386.2478. Found [M+H]$^+$ 386.2474.

(2l): 2-(2-([1,1'-Biphenyl]-4-yl)propan-2-yl)-*N,N*-diethyl-5-methylbenzamide

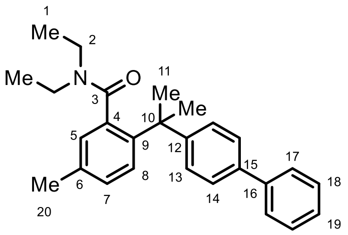

General Procedure E: The reaction was carried out with styrene derivative **9** (400 mol%) and was run for 72 h. Purification of the residue by FCC (hexane/EtOAc 0–20%) afforded the title compound (23.9 mg, 62%) as an orange oil. ν_{max}/cm^{-1}: 2968 (m), 2929 (m), 1628 (s), 1486 (s); ^{1}H NMR (400 MHz, CDCl$_3$): δ 7.62 – 7.53 (2H, m, C**17**-H), 7.48 (2H, d, *J* = 8.0 Hz, C**14**-H), 7.46 – 7.41 (2H, m, C**18**-H), 7.38 (1H, d, *J* = 8.0 Hz, C**8**-H), 7.36 – 7.31 (3H, m, C**13**-H, C**19**-H), 7.13 (1H, dd, *J* = 8.0, 2.0 Hz, C**7**-H), 6.91 – 6.84 (1H, m, C**5**-H), 3.26 – 3.03 (2H, m, C**2**-H$_2$), 2.91 (1H, dq, *J* = 14.5, 7.5 Hz, C**2**-H$_2$), 2.39 (1H, dq, *J* = 14.5, 7.5 Hz, C**2**-H$_2$), 2.31 (3H, s, C**20**-H$_3$), 1.86 (3H, s, C**11**-H$_3$), 1.71 (3H, s, C**11**-H$_3$), 1.09 – 0.92 (6H, m, C**1**-H$_3$); ^{13}C NMR (101 MHz, CDCl$_3$): δ 171.8 (C**3**), 149.1 (C**12**), 143.7 (C**9**), 141.1 (C**16**), 138.4 (C**15**), 136.4 (C**4**), 135.4 (C**6**), 129.3 (C**7**), 128.9 (C**18**), 128.3 (C**8**), 128.2 (C**5**), 127.7 (C**13**), 127.2 (C1**9**), 127.0 (C**17**), 126.6 (C**14**), 44.1 (C**2**), 43.7 (C**10**), 39.3 (C**2**), 33.0 (C**11**), 30.0 (C**11**), 20.8 (C**20**), 13.7 (C**1**), 12.9 (C**1**); HRMS: (ESI$^+$) calculated for $C_{27}H_{31}NO$ 386.2478. Found [M+Na]$^+$ 386.2475.

(2m): 2-(2-([1,1'-Biphenyl]-4-yl)propan-2-yl)-*N,N*-diethyl-4-methoxybenzamide

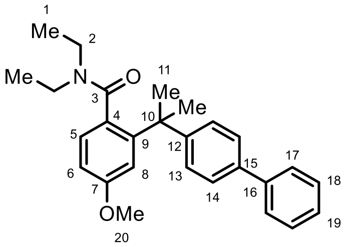

General Procedure E: The reaction was carried out with styrene derivative **9** (400 mol%) and was run for 72 h. Purification of the residue by FCC (hexane/EtOAc 0–30%) afforded the title compound (26.2 mg, 65%) as an orange oil. ν_{max}/cm^{-1}: 2968 (m), 2938 (m), 1619 (s), 1423 (s); ^{1}H NMR (400 MHz, $CDCl_3$): δ 7.59 – 7.53 (2H, m, C**17**-H), 7.50 – 7.40 (4H, m, C**14**-H, C**18**-H), 7.36 – 7.29 (3H, m, C**13**-H, C**19**-H), 7.10 (1H, d, *J* = 2.5 Hz, C**8**-H), 6.99 (1H, d, *J* = 8.5 Hz, C**5**-H), 6.73 (1H, dd, *J* = 8.5, 2.5 Hz, C**6**-H), 3.82 (3H, s, C**20**-H_3), 3.12 (1H, dq, *J* = 14.0, 7.0 Hz, C**2**-H_2), 3.02 (1H, dq, *J* = 14.0, 7.0 Hz, C**2**-H_2), 2.87 (1H, dq, *J* = 14.0, 7.0 Hz, C**2**-H_2), 2.31 (1H, dq, *J* = 14.0, 7.0 Hz, C**2**-H_2), 1.90 (3H, s, C**11**-H_3), 1.71 (3H, s, C**11**-H_3), 0.94 (6H, t, *J* = 7.0 Hz, C**1**-H_3); ^{13}C NMR (101 MHz, $CDCl_3$): δ 171.5 (C**3**), 159.6 (C**7**), 148.9 (C**9**), 148.4 (C**12**), 141.1 (C**16**), 138.5 (C**15**), 129.4 (C**4**), 128.9 (C**5**), 128.9 C**18**), 127.7 (C**13**), 127.2 (C1**9**), 127.0 (C**14**), 126.6 (C**17**), 114.9 (C**8**), 109.8 (C**6**), 55.4 (C**20**), 44.2 (C**2**), 44.1 (C**10**), 39.3 (C**2**), 33.3 (C**11**), 29.8 (C**11**), 13.8 (C**1**), 13.0 (C**1**); HRMS: (ESI$^+$) calculated for $C_{27}H_{31}NO_2$ 402.2428. Found $[M+H]^+$ 402.2423.

(4a): *N,N*-Diisopropyl-2-(2-phenylpropan-2-yl)furan-3-carboxamide

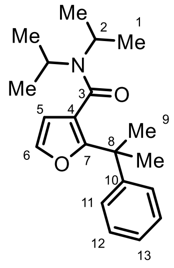

General Procedure D: The reaction was carried out with α-methylstyrene (150 mol%) and was run for 24 h. Purification of the residue by FCC (toluene/Et_2O 5–10%) afforded the title compound (40.2 mg, 94%) as a yellow oil. ν_{max}/cm^{-1}: 2969 (m), 2932 (m), 1623 (s), 1435 (s); ^{1}H NMR (400 MHz, $CDCl_3$): δ 7.29 – 7.21 (4H, m, C**11**-H, C**12**-H), 7.21 – 7.19 (1H, m, C**6**-H), 7.16 – 7.11 (1H, m, C**13**-H) 6.19 (1H, d, *J* = 1.9 Hz, C**5**-H), 3.98 (1H, hept, *J* = 7.0 Hz, C**2**-H), 3.38 (1H, hept, *J* = 7.0 Hz, C**2**-H), 1.71 (6H, s, C**9**-H), 1.43 (6H, d, *J* = 7.0 Hz, C**1**-H_3), 1.06 (6H, d, *J* = 7.0 Hz, C**1**-H_3); ^{13}C NMR (101 MHz, $CDCl_3$): δ 166.2 (C**3**), 157.5 (C**7**), 148.0 (C**10**), 140.2 (C**6**), 128.3 (C**12**), 126.2 (C**13**), 126.1 (C**11**), 117.9 (C**4**), 109.6 (C**5**), 50.9 (C**2**), 45.8 (C**2**), 41.6 (C**8**), 28.4 (C**9**), 20.7 (C**1**), 20.32 (C**1**); HRMS: (ESI$^+$) calculated for $C_{20}H_{27}NO_2$ 314.2115. Found $[M+H]^+$ 314.2124.

*The structure of compound **4a** was confirmed by single crystal X-ray diffraction of crystals obtained from $CHCl_3$ [CCDC deposition number: 2084176].*

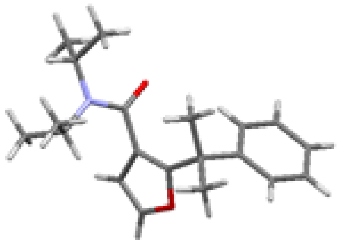

(4b): *N,N*-Diethyl-2-(2-phenylpropan-2-yl)furan-3-carboxamide

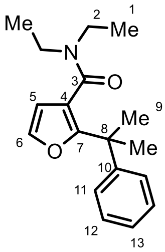

General Procedure D: The reaction was carried out with α-methylstyrene (150 mol%) and was run for 24 h. Purification of the residue by FCC (petroleum ether/EtOAc 50%) afforded the title compound (20.3 mg, 71%) as a colorless oil. ν_{max}/cm^{-1}: 2975 (m), 2986 (m), 1772 (m), 1631 (s), 1163 (s); ^{1}H NMR (400 MHz, $CDCl_3$): δ 7.30 – 7.23 (5H, m, C**6**-H, C**11**-H, C**12**-H), 7.20 – 7.09 (1H, m, C**13**-H), 6.26 (1H, s, C**5**–H), 3.39 (2H, q, *J* = 7.0 Hz, C**2**-H_2), 3.10 (2H, q, *J* = 7.0 Hz, C**2**-H_2), 1.68 (6H, s, C**9**–H_3), 1.12 – 0.97 (6H, m, C**1**-H_3); ^{13}C NMR (101 MHz, $CDCl_3$): δ 167.6 (C**3**), 157.8 (C**7**), 147.3 (C**10**), 140.6 (C**6**), 128.3 (C**12**), 126.4 (C**13**), 126.2 (C**11**), 115.4 (C**4**), 110.0 (C**5**), 43.4 (C**2**), 41.5 (C**8**), 39.4 (C**2**), 28.2 (C**9**), 14.0 (C**1**), 12.3 (C**1**); HRMS: (ESI$^+$) calculated for $C_{18}H_{23}NO_2Na$ 308.1621. Found [M+Na]$^+$ 308.1632.

(4c): *N,N*-Diisopropyl-2-(2-phenylbutan-2-yl)furan-3-carboxamide

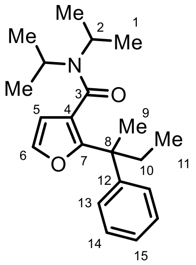

General Procedure D: The reaction was carried out with but-1-en-2-ylbenzene (150 mol%) and was run for 24 h. Purification of the residue by FCC (hexane/EtOAc 0–20%) afforded the title compound (25.1 mg, 77%) as a colorless oil. ν_{max}/cm^{-1}: 2968 (m), 2937 (m), 1629 (s), 1438 (s); ^{1}H NMR (400 MHz, $CDCl_3$): δ 7.28 – 7.24 (4H, m, C**13**-H, C**14**-H), 7.23 (1H, d, *J* = 2.0 Hz, C**6**-H), 7.18 – 7.11 (1H, m, C**15**-

H), 6.20 (1H, d, J = 2.0 Hz, C**5**-H), 3.99 (1H, hept, J = 6.5 Hz, C**2**-H), 3.38 (1H, hept, J = 6.5 Hz, C**2**-H), 2.38 – 2.21 (1H, m, C**10**-H_2), 2.15 – 2.01 (1H, m, C**10**-H_2), 1.63 (3H, s, C**9**-H_3), 1.49 – 1.38 (6H, m, C**1**-H_3), 1.09 (3H, d, J = 6.5 Hz, C**1**-H_3), 1.03 (3H, d, J = 6.5 Hz, C**1**-H_3), 0.76 (3H, t, J = 7.5 Hz, C**11**-H_3); ^{13}C NMR (101 MHz, $CDCl_3$): δ 166.3 (C**3**), 156.8 (C**7**), 147.5 (C**12**), 140.2 (C**6**), 128.3 (C**14**), 126.6 (C**13**), 126.1 (C**15**), 119.2 (C**4**), 109.6 (C**5**), 50.8 (C**2**), 45.8 (C**8**), 45.6 (C**2**), 32.8 (C**10**), 24.2 (C**9**), 20.8 (C**1**), 20.7 (C**1**), 20.4 (C**1**), 20.3 (C**1**), 9.2 (C**11**); HRMS: (ESI$^+$) calculated for $C_{21}H_{29}NO_2$ 328.2271. Found $[M+H]^+$ 328.2262; $[\alpha]^{25}_D$ = -24.2 (c = 0.20, $CHCl_3$).

General procedure D: The reaction was carried out on a 0.143 mmol scale, with styrene derivative (150 mol%), **(*S*)-L-5** and was run for 24 h. Purification of the residue by FCC (hexane/EtOAc 0–20%) afforded the title compound (26.7 mg, 57%, 60:40 *e.r.*) as a colorless oil.

Chiral SFC Conditions: (DAICEL CHIRALPAK-IE column (25 cm), CO_2:MeOH 97:3 to 94:6 over 30 minutes, 2 mL/min, 140 bar, 60 °C). Retention times: 19.0 minutes (major), 20.4 minutes (minor), *e.r.* = 60:40.

(4d): *N,N*-Diisopropyl-2-(2-phenylpentan-2-yl)furan-3-carboxamide

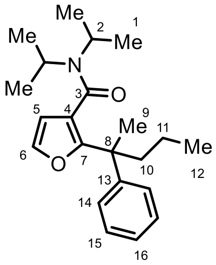

General Procedure D: The reaction was carried out with pent-1-en-2-ylbenzene (150 mol%) and was run for 24 h. Purification of the residue by FCC (hexane/EtOAc 0–10%) afforded the title compound (25.5 mg, 75%) as an orange oil. ν_{max}/cm^{-1}: 2961 (m), 2872 (m), 1630 (s), 1437 (s); ^{1}H NMR (400 MHz, $CDCl_3$): δ 7.30 – 7.24 (4H, m, C**14**-H, C**15**-H), 7.23 (1H, d, J = 2.0 Hz, C**6**-H), 7.19 – 7.11 (1H, m, C**16**-H), 6.20 (1H, d, J = 2.0 Hz, C**5**-H), 4.01 (1H, hept, J = 6.5 Hz, C**2**-H), 3.39 (1H, hept, J = 6.5 Hz, C**2**-H), 2.26 – 1.92 (2H, m, C**10**-H_2), 1.66 (3H, s, C**9**-H_3), 1.49 – 1.42 (6H, m, C**1**-H_3), 1.29 – 1.13 (2H, m, C**11**-H_2), 1.12 – 1.02 (6H, m, C**1**-H_3), 0.88 (3H, t, J = 7.0 Hz, C**12**-H_3); ^{13}C NMR (101 MHz, $CDCl_3$): δ 166.4 (C**3**), 157.0 (C**7**), 147.5 (C**13**), 140.3 (C**6**), 128.3 (C**15**), 126.6 (C**14**), 126.1 (C**16**), 118.9 (C**4**), 109.6 (C**5**), 50.9 (C**2**), 45.8 (C**2**), 45.3 (C**8**), 42.6 (C**10**), 24.7 (C**9**), 20.8 (C**1**), 20.7 (C**1**), 20.4 (C**1**), 20.3 (C**1**), 17.9 (C**11**), 14.8 (C**12**); HRMS: (ESI$^+$) calculated for $C_{22}H_{31}NO_2Na$ 364.2247. Found $[M+Na]^+$ 364.2249.

(4e): 2-(1-Cyclobutyl-1-phenylethyl)-*N,N*-diisopropylfuran-3-carboxamide

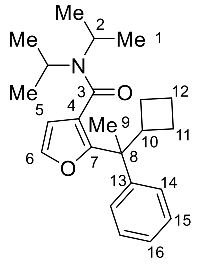

General Procedure D: The reaction was carried out with (1-cyclobutylvinyl)benzene (150 mol%) and was run for 24 h. Purification of the residue by FCC (hexane/EtOAc 20%) afforded the title compound (21.4 mg, 61%) as a colourless oil. R_f = 0.56 (hexane/EtOAc 20%); ν_{max}/cm^{-1}: 2969 (m), 2938 (m), 2967 (w), 1703 (w), 1673 (s), 1599 (m), 1511 (m); ^{1}H NMR (500 MHz, acetone-d_6) δ 7.43 (d, *J* = 2.0 Hz, 1H, C**6**-H), 7.29 – 7.20 (m, 4H, C**14**-H_2, C**15**-H_2), 7.18 – 7.14 (m, 1H, C**16**-H), 6.37 (d, *J* = 2.0 Hz, 1H, C**5**-H), 4.03 (p, *J* = 6.5 Hz, 1H, C**2**-H), 3.50 (p, *J* = 6.5 Hz, 1H, C**2**-H'), 3.44 – 3.34 (m, 1H, C**10**-H), 2.06 – 1.93 (m, 3H, C**11**-H_3), 1.92 – 1.76 (m, 2H, C**11**-H', C**12**-H), 1.69 – 1.60 (m, 4H, C**9**-H_3, C**12**-H'), 1.47 – 1.39 (m, 6H, C**1**-H_3), 1.17 (d, *J* = 6.5 Hz, 3H, C**1**-H_3'), 1.08 (d, *J* = 6.5 Hz, 3H, C**1**-H_3''); ^{13}C NMR (126 MHz, acetone-d_6) δ 166.5 (C**3**), 156.9 (C**7**), 148.1 (C**13**), 141.5 (C**6**), 129.0 (C**15**), 127.7 (C**14**), 126.8 (C**16**), 121.0 (C**4**), 110.6 (C**5**), 51.5 (C**2**), 48.0 (C**8**), 46.2 (C**2'**), 44.6 (C**10**), 25.3 (C**11**), 22.7 (C**9**), 21.0 (C**1**), 20.8 (C**1'**), 20.7 (C**1''**), 20.6 (C**1'''**), 18.1 (C**12**); HRMS: (ESI$^+$) calculated for $C_{23}H_{31}NO_2$: 353.24. Found $[M+H]^+$: 354.2430

(4f): *N,N*-Diisopropyl-2-(2-(3-(trifluoromethyl)phenyl)propan-2-yl)furan-3-carboxamide

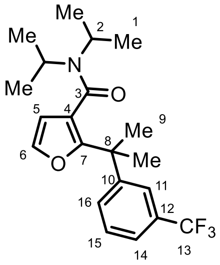

General Procedure D: The reaction was carried out on a 0.143 mmol scale, with 1-(Prop-1-en-2-yl)-3-(trifluoromethyl)benzene (150 mol%) and was run for 24 h. Purification of the residue by FCC (hexane/EtOAc 30%) afforded the title compound (51.3 mg, 94%) as a colorless oil. ν_{max}/cm^{-1}: 2970 (m), 2929 (m), 1629 (s), 1439 (m), 1328 (s); ^{1}H NMR (400 MHz, $CDCl_3$): δ 7.51 – 7.49 (2H, m, ArCH), 7.43 – 7.40 (2H, m, C**15**-H, ArCH), 7.25 (1H, d, *J* = 4.0 Hz, C**6**-H), 6.23 (1H, d, *J* = 4.0 Hz, C**5**-H), 3.96 (1H, hept, *J* = 6.5 Hz, C**2**-H), 3.39 (1H, hept, *J* = 6.5 Hz, C**2**-H), 1.73 (6H, s, C**9**-H_3), 1.43 (6H, d, , *J* = 8.0 Hz, C**1**-H_3), 1.06 (6H, d, *J* = 8.0 Hz, C**1**-H_3); ^{13}C NMR (126 MHz, $CDCl_3$): δ 166.0 (C**3**), 156.5 (C**7**), 149.0 (C**10**), 140.6 (C**6**), 130.3 (C**12**, q, *J* = 35.0 Hz), 130.3 (C**16**), 128.9 (C**15**), 124.4 (**C13**, q, *J* = 273.5 Hz), 123.17 (ArCH, q, *J* = 4.0 Hz), 122.4 (ArCH, q, *J* = 4.0 Hz), 118.4 (C**4**), 109.8 (C**5**), 50.9

(C**2**), 45.9 (C**2**), 41.7 (C**8**), 28.3 (C**9**), 20.7 (C**1**), 20.3 (C**1**); ^{19}F NMR (377 MHz, $CDCl_3$): δ -62.32; HRMS: (ESI$^+$) calculated for $C_{21}H_{26}F_3NO_2Na$ 404.1808. Found $[M+Na]^+$ 404.1828.

(4g): *N,N*-Diisopropyl-2-(2-(3-methoxyphenyl)propan-2-yl)furan-3-carboxamide

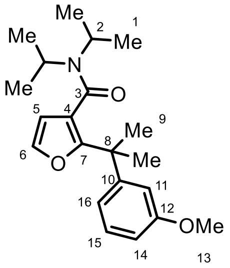

General Procedure D: The reaction was carried out on a 0.143 mmol scale, with 1-methoxy-3-(prop-1-en-2-yl)benzene (150 mol%) and was run for 24 h. Purification of the residue by FCC (petroleum ether/EtOAc 20%) afforded the title compound (39.8 mg, 81%) as a colorless solid. ν_{max}/cm^{-1}: 2972 (m), 2252 (m), 1622 (m), 904 (s); ^{1}H NMR (400 MHz, $CDCl_3$): δ 7.31 – 7.19 (2H, m, C**6**-H, C**15**-H), 6.92 – 6.85 (2H, m, C**14**-H, C**16**-H), 6.77 – 6.57 (1H, m, C**11**-H), 6.32 – 6.12 (1H, m, C**5**-H), 4.01 (1H, hept, *J* = 7.0 Hz, C**2**-H), 3.79 (3H, s, C**13**-H_3), 3.42 (1H, hept, *J* = 7.0 Hz, C**2**-H), 1.72 (6H, s, C**9**-H_3), 1.47 (6H, d, *J* = 7.0 Hz, C**1**-H_3), 1.09 (6H, d, *J* = 7.0 Hz, C**1**-H_3); ^{13}C NMR (101 MHz, $CDCl_3$): δ 166.2 (C**3**), 159.6 (C**12**), 157.3 (C**7**), 149.8 (C**10**), 140.3 (C**6**), 129.3 (C**15**), 118.8 (C**14**), 118.0 (C**4**), 112.7 (C**16**), 111.0 (C**11**), 109.7 (C**5**), 55.3 (C**13**), 50.9 (C**2**), 45.8 (C**2**), 41.7 (C**8**), 28.4 (C**9**), 21.3 (C**1**), 19.8 (C**1**); HRMS: (ESI$^+$) calculated for $C_{21}H_{29}NO_3Na$ 366.2045. Found $[M+Na]^+$ 366.2045. m.p. 74–76 °C ($CDCl_3$).

(4h): *N,N*-Diisopropyl-2-(2-(4-methoxyphenyl)propan-2-yl)furan-3-carboxamide

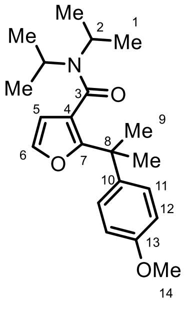

General Procedure D: The reaction was carried out on a 0.143 mmol scale, with 1-methoxy-4-(prop-1-en-2-yl)benzene (150 mol%) and was run for 24 h. Purification of the residue by FCC (petroleum ether/EtOAc 20%) afforded the title compound (40.3 mg, 82%) as a colorless oil. ν_{max}/cm^{-1}: 2968 (m), 2932 (m), 1627 (s), 1251 (s); ^{1}H NMR (400 MHz, $CDCl_3$): δ 7.25 – 7.19 (m, 2H, 2 × C**12**-H), 7.13 (t, *J* = 9.0 Hz, 1H, C**6**-H), 6.82 (d, *J* = 9.0 Hz, 2H, 2 × C**11**-H), 6.20 (s, 1H, C**5**-H), 3.99 (hept, *J* = 8.0 Hz, 1H, C**2**-H), 3.76 (s, 3H, C**14**-H_3), 3.40 (hept, *J* = 8.0 Hz, 1H, C**2**-H_2), 1.69 (s, 6H, 2 × C**9**-H_3), 1.44 (d, *J* = 8.0 Hz, 6H, 2 × C**1**-H_3), 1.08 (d, *J* = 8.0 Hz, 6H, 2 × C**1**-H_3); ^{13}C NMR (101 MHz, $CDCl_3$): δ 166.3

(C**3**), 157.9 (C**7**), 157.7 (C**10**), 140.2 (C**6**), 127.2 (C**12**), 117.7 (C**4**), 113.6 (C**11**), 113.3 (C**13**), 109.6 (C**5**), 55.3 (C**14**), 50.9 (C**2**), 45.8 (C**2**), 41.0 (C**8**), 28.6 (C**9**), 20.7 (C**1**), 20.3 (C**1**); HRMS: (ESI$^+$) calculated for $C_{21}H_{30}NO_3$ 344.2220. Found $[M+H]^+$ 344.2236.

(4i): The synthesis and data for this compound is provided later.

(4j): (*R*)-*N,N*-Diisopropyl-2-(2-(4-methylcyclohex-3-en-1-yl)propan-2-yl)furan-3-carboxamide

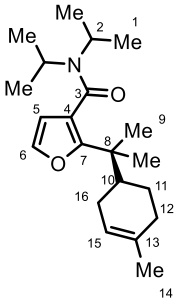

General Procedure D: The reaction was carried out on a 0.143 mmol scale, with (*S*)-(-)-limonene (150 mol%) and was run for 24 h. Purification of the residue by FCC (petroleum ether/EtOAc 20%) afforded the title compound (30.5 mg, 64%) as a brown oil. ν_{max}/cm^{-1}:2965 (m), 2925 (m), 1632 (s), 1332 (s); ^{1}H NMR (400 MHz, $CDCl_3$): δ 7.22 (d, 1H, *J* = 2.0 Hz, C**6-**H), 6.15 (d, 1H, *J* = 2.0 Hz, C**5-**H), 5.33 (s, 1H, C**15-**H), 3.99 (hept, 1H, *J* = 7.0 Hz, C**2-**H), 3.41 (hept, 1H, *J* = 7.0 Hz, C**2-**H), 2.05-1.85 (m, 4H, C**11/12-**H_2), 1.85-1.67 (m, 2H, C**16-**H_2), 1.60 (s, 3H, C**14-**H), 1.48 (d, 6H, *J* = 7.0 Hz, 2 × C**1-**H_3), 1.27 (s, 3H, C**9-**H_3), 1.26-1.16 (m, 1H, C**10-**H), 1.23 (s, 3H, C**9-**H_3), 1.13 (d, 6H, *J* = 7.0 Hz, 2 × C**1-**H_3); ^{13}C NMR (101 MHz, $CDCl_3$): δ 166.6 (C**3**), 158.2 (C**7**), 139.5 (C**6**), 133.7 (C**13**), 121.0 (C**15**), 117.5 (C**4**), 109.2 (C**5**), 50.7 (C**2**), 45.6 (C**2**), 43.1 (C**11**), 39.8 (C**8**), 31.3 (C**12**), 26.9 (C**16**), 24.6 (C**10**), 23.3 (C**14**), 23.2 (C**9**), 23.1 (C**9**), 20.2 (C**1**), 20.1 (C**1**); HRMS: (ESI$^+$) calculated for $C_{21}H_{33}NO_2Na$ 354.2404. Found $[M+Na]^+$ 354.2421; $[\alpha]^{22}_D$ -25.5 (c = 1.00, CH_2Cl_2).

(4k): *N,N*-Diisopropyl-2-(2-methylpentan-2-yl)furan-3-carboxamide

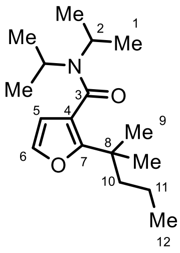

General Procedure D: The reaction was carried out on a 0.143 mmol scale, with pent-1-en-2-ylbenzene (150 mol%) and was run for 24 h. Purification of the residue by FCC (petroleum ether/EtOAc 30%) afforded the title compound (30.9 mg, 77%) as a brown oil. ν_{max}/cm^{-1}: 2962 (s), 2931 (m), 1634 (s), 1438 (s), 1333 (s); ^{1}H NMR (400 MHz, $CDCl_3$): δ 7.21 (1H, s, C**6-**H), 6.13 (1H, s, C**5-**H), 4.05 – 3.88 (1H, m, C**2-**H), 3.57 – 3.28 (1H, m, C**2–**H), 1.62 – 1.55 (2H, m, C**10-**H_2), 1.48 (6H, d, *J* = 6.0 Hz, C**1–**H_3),

1.27 (6H, s, C**9**–H̲3), 1.24 – 1.14 (2H, m, C**11**-H̲2), 1.12 (6H, d, J = 6.0 Hz, C**1**-H̲3), 0.86 – 0.78 (3H, m, C**12**-H̲3); ^{13}C NMR (101 MHz, CDCl3): δ 166.7 (C**3**), 159.1 (C**7**), 139.6 (C**6**), 117.2 (C**4**), 109.4 (C**5**), 50.8 (C**2**), 45.7 (C**2**), 45.0 (C**10**), 37.4 (C**8**), 26.7 (C**9**), 21.0 (C**1**), 20.3 (C**1**), 18.2 (C**11**), 14.7 (C**12**); HRMS: (ESI$^+$) calculated for $C_{17}H_{30}NO_2$ 280.2271. Found [M+H]$^+$ 280.2274.

(4l): 2-(4-(Benzyloxy)-2-methylbutan-2-yl)-*N*,*N*-diisopropylfuran-3-carboxamide

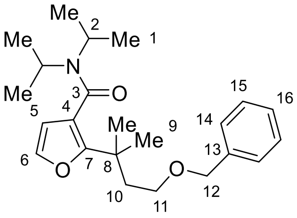

General Procedure D: The reaction was carried out with (((3-methylbut-3-en-1-yl)oxy)methyl)benzene (150 mol%) and was run for 24 h. Purification of the residue by FCC (hexane/EtOAc 10%) afforded the title compound (28.2 mg, 76%) as a yellow oil. R_f = 0.38 (hexane/EtOAc 20%); ν_{max}/cm^{-1}: 2967 (s), 2932 (m), 2872 (m), 1630 (s), 1515 (m); ^{1}H NMR (500 MHz, CDCl3) δ 7.33 – 7.21 (m, 6H, C**6**-H̲, C**14**-H̲2, C**15**-H̲2, C**16**-H̲), 6.15 (d, J = 2.0 Hz, 1H, C**5**-H̲), 4.43 (s, 2H, C**12**-H̲2), 3.95 (p, J = 7.0 Hz, 1H, C**2**-H̲), 3.48 – 3.37 (m, 3H, C**2**-H̲, C**11**-H̲2), 2.03 (t, J = 7.0 Hz, 2H, C**10**-H̲2), 1.49 (d, J = 7.0 Hz, 6H, C**1**-H̲3), 1.34 (s, 6H, C**9**-H̲3), 1.10 (d, J = 7.0 Hz, 6H, C**1**-H̲3); ^{13}C NMR (126 MHz, CDCl3) δ 166.6 (C**3**), 157.3 (C**7**), 139.9 (C**6**), 138.8 (C**13**), 128.4 (C**14**), 127.6 (C**15**), 127.5 (C**16**), 117.4 (C**4**), 109.5 (C**5**), 72.9 (C**12**), 67.8 (C**11**), 50.9 (C**2**), 45.8 (C**2**), 41.8 (C**10**), 36.3 (C**8**), 27.1 (C**9**), 20.7 (C**1**), 20.4 (C**1**); HRMS: (ESI$^+$) calculated for $C_{23}H_{33}NO_3$: 371.25. Found [M+H]$^+$: 372.2531.

(6a): *N*,*N*-Diisopropyl-1-methyl-4-(2-phenylpropan-2-yl)-1*H*-pyrrole-3-carboxamide

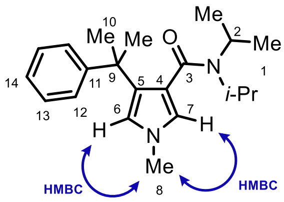

General Procedure D: The reaction was carried out with α-methylstyrene (400 mol%) and was run for 48 h. Purification of the residue by FCC (hexane/EtOAc 30–50%) afforded the title compound (28.3 mg, 87%) as an orange oil. ν_{max}/cm^{-1}: 2954 (m), 2929 (m), 1620 (s), 1440 (s); ^{1}H NMR (400 MHz, CDCl3): δ 7.36 (2H, dd, J = 8.0, 1.0 Hz, C**12**-H̲), 7.23 (2H, dd, J = 8.0, 1.0 Hz, C**13**-H̲), 7.15 – 7.06 (1H, m, C**14**-H̲), 6.41 (1H, d, J = 2.5 Hz, C**6**-H̲), 6.30 (1H, d, J = 2.5 Hz, C**7**-H̲), 3.99 – 3.10 (2H, m, C**2**-H̲), 3.55 (3H, s, C**8**-H̲3), 1.70 (6H, s, C**10**-H̲3), 1.48 – 0.80 (12H, m, C**1**-H̲3); ^{13}C NMR (101 MHz, CDCl3): δ 168.2 (C**3**), 150.8 (C**11**), 132.8 (C**5**), 127.9 (C**13**), 126.7 (C**12**), 125.3 (C**14**), 119.9 (C**4**), 119.7 (C**7**),

119.6 (C**6**), 39.1 (C**9**), 36.1 (C**8**), 31.2 (C**10**), 20.7 (C**1**); HRMS: (ESI$^+$) calculated for $C_{21}H_{30}N_2ONa$ 349.2250. Found $[M+H]^+$ 349.2267.

*Note: C**2** was not observed by ^{13}C NMR analysis.*

*The regiochemistry of **6a** and other C**5**-alkylated pyrroles **6b-e** was determined through 2D NMR spectroscopy (COSY, HSQC, HMBC) and corroborated through nuclear Overhauser effect experiments on example **6e** (see below). An HMBC correlation was observed between H**6** and C**8** as well as H**7** and C**8** in all cases. The ^{13}C chemical shift of C**5** also changes significantly between the starting material (108.4 ppm) and product (132.8 ppm), whereas the chemical shift of C**6** and C**7** changes only slightly (Starting material: C**6** = 121.2 ppm; C**7** = 123.9 ppm, vs. **6a**: C**6** = 119.6 ppm; C**7** = 119.7 ppm). This analysis, taken in combination with the nOe data discussed for **6e**, provides strong evidence for the C**5** alkylated regioisomer.*

(6b): *N,N*-Diisopropyl-1-methyl-4-(2-phenylbutan-2-yl)-1*H*-pyrrole-3-carboxamide

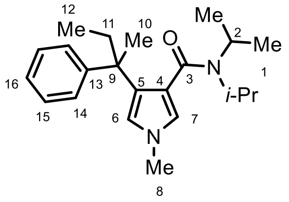

General Procedure D: The reaction was carried out with but-1-en-2-ylbenzene (150 mol%) and was run for 16 h. Purification of the residue by FCC (hexane/EtOAc 30%) afforded the title compound (25.0 mg, 73%) as an orange oil. ν_{max}/cm^{-1}: 2954 (m), 2927 (m), 1619 (s), 1440 (s); ^{1}H NMR (400 MHz, $CDCl_3$): δ 7.31 (2H, dd, *J* = 8.0, 1.5 Hz, C**14**-H), 7.25 – 7.19 (2H, m, C**15**-H), 7.15 – 7.07 (1H, m, C**16**-H), 6.41 (1H, d, *J* = 2.5 Hz, C**6**-H), 6.29 (1H, d, *J* = 2.5 Hz, C**7**-H), 4.01 – 3.58 (1H, m, C**2**-H), 3.55 (3H, s, C**8**-H_3), 3.40 – 2.96 (1H, m, C**2**-H), 2.35 – 2.20 (1H, m, C**11**-H_2), 2.02 (1H, dq, *J* = 14.5, 7.5 Hz, C**11**-H_2), 1.65 (3H, s, C**10**-H_3), 1.40 – 0.82 (12H, m, C**1**-H_3), 0.71 (1H, t, *J* = 7.5 Hz, C**12**-H_3); ^{13}C NMR (101 MHz, $CDCl_3$): δ 168.3 (C**3**), 149.4 (C**13**), 132.3 (C**5**), 127.8 (C**15**), 127.4 (C**14**), 125.2 (C**16**), 120.3 (C**7**), 120.0 (C**4**), 119.6 (C**6**), 42.7 (C**9**), 36.2 (C**8**), 34.3 (C**11**), 26.6 (C**10**), 20.7 (C**1**), 9.2 (C**12**); HRMS: (ESI$^+$) calculated for $C_{22}H_{33}N_2O$ 341.2587. Found $[M+H]^+$ 341.2582.

*Note: C**2** was not observed by ^{13}C NMR analysis.*

(6c): 4-(2-(4-Fluorophenyl)propan-2-yl)-N,N-diisopropyl-1-methyl-1*H*-pyrrole-3-carboxamide

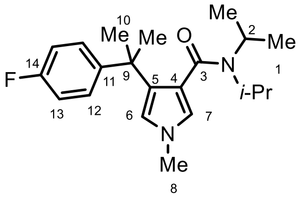

General Procedure D: The reaction was carried out with 4-fluoro-α-methyl-styrene (400 mol%) and was run for 48 h. Purification of the residue by FCC (hexane/EtOAc 20–40%) afforded the title compound (25.5 mg, 74%) as a colorless oil. ν_{max}/cm^{-1}: 2855 (m), 2932 (m), 1619 (s), 1506 (s); ^{1}H NMR (400 MHz, $CDCl_3$): δ 7.37 – 7.28 (2H, m, C**12**-H), 6.94 – 6.83 (2H, m, C**13**-H), 6.41 (1H, d, J = 2.5 Hz, C**6**-H), 6.31 (1H, d, J = 2.5 Hz, C**7**-H), 3.91 – 2.88 (2H, m, C**2**-H), 3.55 (3H, s, C**8**-H_3), 1.68 (6H, s, C**10**-H_3), 1.32 – 0.92 (12H, m, C**1**-H_3); ^{13}C NMR (101 MHz, $CDCl_3$): δ 168.0 (C**3**), 160.9 (d, J = 243.0 Hz, C**14**), 146.6 (d, J = 3.0 Hz, C**11**), 132.7 (C**5**), 128.2 (d, J = 7.5 Hz, C**12**), 119.8 (C**4**), 119.7 (C**6**), 119.6 (C**7**), 114.36 (d, J = 21.0 Hz, C**13**), 49.2 (C**2**), 45.9 (C**2**), 38.7 (C**9**), 36.2 (C**8**), 31.4 (C**10**), 20.7 (C**1**); ^{19}F NMR (377 MHz, $CDCl_3$): δ -119.29; HRMS: (ESI$^+$) calculated for $C_{21}H_{29}FNO$ 345.2337. Found $[M+H]^+$ 345.2326.

(6d): *N,N*-Diisopropyl-1-methyl-4-(2-methylpentan-2-yl)-1*H*-pyrrole-3-carboxamide

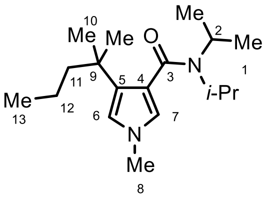

General Procedure D: The reaction was carried out with 2-methyl-1-pentene (400 mol%) and was run for 48 h. Purification of the residue by FCC (hexane/EtOAc 20–30%) afforded the title compound (24.3 mg, 83%) as a yellow oil. ν_{max}/cm^{-1}: 2957 (m), 2929 (m), 1623 (s), 1439 (s); ^{1}H NMR (400 MHz, $CDCl_3$): δ 6.40 (1H, d, J = 2.5 Hz, C**6**/**7**-H), 6.29 (1H, d, J = 2.5 Hz, C**6**/**7**-H), 4.16 – 3.26 (2H, m, C**2**-H), 3.54 (3H, s, C**8**-H_3), 1.64 – 1.52 (2H, m, C**11**-H_2), 1.39 – 1.07 (14H, m, C**1**-H_3, C**12**-H_2), 1.23 (6H, s, C**10**-H_3), 0.86 (3H, t, J = 7.5 Hz, C**13**-H_3); ^{13}C NMR (101 MHz, $CDCl_3$): δ 169.3 (C**3**), 131.8 (C**5**), 119.7 (C**4**), 119.1 (C**6**/**7**), 118.9 (C**6**/**7**), 45.9 (C**11**), 36.1 (C**8**), 34.8 (C**9**), 29.2 (C**10**), 20.8 (C**1**), 18.3 (C**12**), 15.0 (C**13**); HRMS: (ESI$^+$) calculated for $C_{18}H_{32}N_2O$ 293.2587. Found $[M+H]^+$ 293.2576.

*Note: C**2** was not observed by ^{13}C NMR analysis.*

(6e): *N,N*-Diisopropyl-1-methyl-4-(2-methyl-4-phenylbutan-2-yl)-1*H*-pyrrole-3-carboxamide

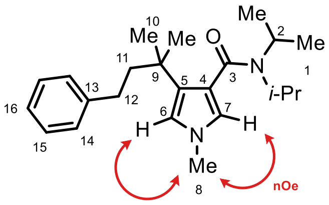

General Procedure D: The reaction was carried out with (3-methylbut-3-en-1-yl)benzene (64.0 µL, 0.40 mmol, 400 mol%) and the reaction was run for 48 h. Purification of the residue by FCC (hexane/EtOAc 20%) afforded the title compound (25.4 mg, 72%) as a colorless oil. ν_{max}/cm^{-1}: 2961 (s), 1620 (s), 1530 (m), 1439 (s); ^{1}H NMR (400 MHz, $CDCl_3$): δ 7.26 – 7.18 (4H, m, 2 × C**14**-H, 2 × C**15**-

H), 7.12 (1H, m, C**16**-H), 6.43 (1H, d, *J* = 2.5 Hz, C**6**/**7**-H), 6.36 (1H, d, *J* = 2.5 Hz, C**6**/**7**-H), 4.18 – 3.35 (2H, *br*. s, 2 × C**2**-H), 3.57 (3H, s, C**8**-H_3), 2.60 – 2.45 (2H, m, C**12**-H_2), 2.02 – 1.87 (2H, m, C**11**-H_2), 1.32 (18H, s, 4 × C**1**-H_3, 2 × C**9**-H_3); ^{13}C NMR (101 MHz, $CDCl_3$): δ 169.2 (C**3**), 143.8 (C**13**), 131.1 (C**5**), 128.6 (C**14**), 128.2 (C**15**), 125.4 (C**16**), 119.9 (C**4**), 119.1, 119.0 (C**6**, C**7**), 45.5 (C**11**), 36.1 (C**8**), 34.9 (C**10**), 31.7 (C**12**), 29.1 (C**9**), 20.7 (C**1**); *A signal corresponding to C**2** was not observed.* HRMS (ESI$^+$): Calculated for $C_{23}H_{35}N_2O$: 355.2744. Found $[M+H]^+$: 355.2746.

*An nOe was observed between C**8**-H_3 and C**6**-H. An nOe was observed between C**8**-H_3 and C**7**-H. If the product was instead C**7**-alkylated, an nOe between C**8**-H_3 and **both** C**6**-H **and** C**5**-H would not be expected. This was corroborated through nOe experiments on the starting material, where an nOe was observed between C**8**-H_3 and C**6**-H and C**7**-H, but **not** C**5**-H.*

(6f): *N,N*-diisopropyl-1-methyl-4-(2-((8*R*,9*S*,13*S*,14*S*)-13-methyl-17-oxo-7,8,9,11,12,13,14,15,16,17-decahydro-6*H*-cyclopenta[*a*]phenanthren-3-yl)propan-2-yl)-1*H*-pyrrole-3-carboxamide

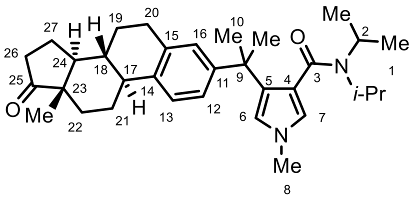

General Procedure D: The reaction was carried out with (8*R*,9*S*,13*S*,14*S*)-13-methyl-3-(prop-1-en-2-yl)-6,7,8,9,11,12,13,14,15,16-decahydro-17*H*-cyclopenta[*a*]phenanthren-17-one (150 mol%) and was run for 72 h. Purification of the residue by FCC (hexane/EtOAc 20–40%) afforded the title compound (26.6 mg, 53%) as a beige solid. v_{max}/cm^{-1}: 2962 (m), 2928 (m), 2859(m), 1737 (s), 1611 (s); ^{1}H NMR (400 MHz, $CDCl_3$): δ 7.15 – 7.08 (2H, m, C**12**-H, C**13**-H), 7.07 (1H, s, C**16**-H), 6.37 (1H, d, *J* = 2.5 Hz, C**6/7**-H), 6.31 (1H, d, *J* = 2.5 Hz, C**6/7**-H), 3.96 – 3.66 (1H, m, C**2**-H), 3.53 (3H, s, C**8**-H_3), 3.37 – 3.14 (1H, m, C**2**-H), 2.84 (2H, dd, *J* = 9.0, 4.0 Hz, C**19**-H_2), 2.47 (1H, dd, *J* = 18.5, 9.0 Hz, C**27**-H_2), 2.42 – 2.32 (1H, m, C**18**-H), 2.30 – 2.17 (1H, m, CH), 2.17 – 2.08 (1H, m, CH), 2.08 – 1.85 (3H, m, CH), 1.67 (6H, d, *J* = 4.5 Hz, C**10**-H_3), 1.64 – 1.12 (15H, m, CH, C**1**-H_3), 0.87 (3H, s, C**28**-H_3); ^{13}C NMR (101 MHz, $CDCl_3$): δ 221.2 (C**25**), 168.2 (C**3**), 148.1 (C**11**), 136.3 (C**14**), 135.4 (C**15**), 133.1 (C**5**), 127.1 (C**16**), 124.8 (C**12/13**), 124.7 (C**12/13**), 119.9 (C**4**), 119.6 (C**6/7**), 119.6 (C**6/7**), 50.7 (CH), 48.1 (C**9**), 44.5 (CH), 38.5 (CH), 38.4 (CH), 36.3 (C**8**), 36.0 (C**27**), 31.8 (CH), 31.2 (C**10**), 29.8 (C**19**), 26.8 (CH), 25.8 (C**18**), 21.7 (C**1**), 20.7 (C**23**), 14.0 (C**28**); m.p. 268–270 °C ($CDCl_3$). *A mass could not be observed by ESI or MALDI.*

*Note: C**2** was not observed by ^{13}C NMR analysis.*

(6g): (*S*)-*N*,*N*-diisopropyl-1-methyl-4-(2-(4-methylcyclohex-3-en-1-yl)propan-2-yl)-1*H*-pyrrole-3-carboxamide

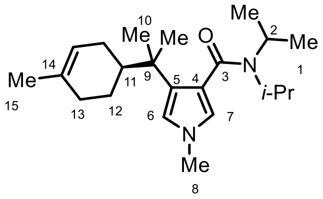

General Procedure D: The reaction was carried out with (*S*)-(-)-Limonene (64.6 µL, 0.40 mmol, 400 mol%) and the reaction was run for 48 h. Purification of the residue by FCC (hexane/EtOAc 10–20%) afforded the title compound (22.2 mg, 64%) as a colorless oil. $[\alpha]_D^{24}$ -20.3 (c = 0.12, $CHCl_3$); ν_{max}/cm^{-1}: 2962 (s), 2925 (m), 1616 (s), 1530 (m), 1439 (s); ^1H NMR (400 MHz, $CDCl_3$): δ 6.40 (1H, d, *J* = 2.5 Hz, C**6** *or* C**7**), 6.29 (1H, d, *J* = 2.5 Hz, C**6** *or* C**7**), 5.32 (1H, m, C**16**-H), 4.24 – 3.16 (2H, *br*. s, 2 × C**2**-H), 3.55 (3H, s, C**8**-H_3), 2.05 – 1.70 (6H, m, 2 × C**7**-H, C**11**-H, 1 × C**12**-H, 2 × C**13**-H), 1.59 (3H, s, C**15**-H_3), 1.54 – 0.94 (19H, m, 4 × C**1**-H_3, 2 × C**9**-H_3, 1 × C**12**-H); ^{13}C NMR (101 MHz, $CDCl_3$): δ 169.2 (C**3**), 133.7 (C**14**), 131.7 (C**5**), 121.6 (C**16**), 119.6 (C**4**), 119.3, 118.8 (C**6**, C**7**), 42.6 (C**11**), 37.1 (C**10**), 36.0 (C**8**), 31.6 (C**13**), 27.4 (C**17**), 25.7 (C**9**), 25.3 (C**9**), 24.6 (C**12**), 23.4 (C**15**), 20.7 (C**1**), 20.5 (C**1**). *A signal corresponding to C**2** was not observed.* HRMS (ESI$^+$): Calculated for $C_{22}H_{37}N_2O$: 345.2900. Found $[M+H]^+$: 345.2909.

(6h): *N*,*N*-Diisopropyl-2-(2-phenylpropan-2-yl)-1*H*-pyrrole-1-carboxamide

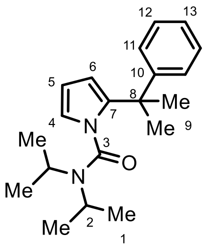

General Procedure D: The reaction was carried out with α-methylstyrene (400 mol%) and was run for 48 h. Purification of the residue by FCC (toluene/Et_2O 1–2%) afforded the title compound (31.3 mg, 72%) as a yellow oil. ν_{max}/cm^{-1}: 2969 (m), 2933 (m), 1685 (s), 1423 (s), 1322 (s); ^1H NMR (400 MHz, $CDCl_3$): δ 7.32 – 7.28 (2H, m, C**11**-H), 7.27 – 7.21 (2H, m, C**12**-H), 7.17 – 7.10 (1H, m, C**13**-H), 6.57 (1H, dd, *J* = 3.0, 1.5 Hz, C**4**-H), 6.20 (1H, dd, *J* = 3.0, 1.5 Hz, C**5**-H), 6.14 – 6.07 (1H, m, C**6**-H), 3.30 (2H, h, *J* = 6.5 Hz, C**2**-H), 1.77 (6H, s, C**9**-H_3), 1.12 (6H, d, *J* = 6.5 Hz, C**1**-H_3), 1.04 – 0.93 (6H, m, C**1**-H_3); ^{13}C NMR (101 MHz, $CDCl_3$): δ 152.8 (C**3**), 148.8 (C**10**), 142.2 (C**7**), 128.2 (C**12**), 126.7 (C**11**), 125.7 (C**13**), 120.1 (C**4**), 108.1 (C**5**), 107.4 (C**6**), 48.3 (C**2**), 40.0 (C**8**), 31.2 (C**9**), 20.7 (C**1**), 20.1 (C**1**); HRMS: (ESI$^+$) calculated for $C_{20}H_{29}N_2O$ 313.2274. Found $[M+H]^+$ 313.2283. m.p. 63–65 °C ($CDCl_3$).

(6i): *N,N*-Diisopropyl-2-(2-methyl-4-phenylbutan-2-yl)-1*H*-pyrrole-1-carboxamide

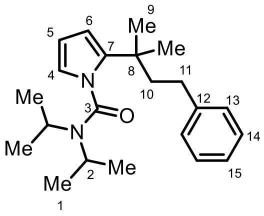

General Procedure D: The reaction was conducted with (3-methylbut-3-en-1-yl)benzene (21.9 mg, 0.15 mmol, 150 mol%) and the reaction was run for 48 h. Purification of the residue by FCC (hexane/EtOAc 2%) afforded the title compound (20.7 mg, 61%) as a colorless oil. ν_{max}/cm^{-1}: 2968 (m), 2935 (m), 1687 (s), 1431 (s), 1325 (s); ^{1}H NMR (400 MHz, $CDCl_3$): δ 7.25 – 7.16 (4H, m, 2 × C**13**-H, 2 × C**14**-H), 7.17 – 7.09 (1H, m, C**15**-H), 6.57 (1H, dd, *J* = 3.0, 1.5 Hz, C**4**-H), 6.10 (1H, m, C**5**-H), 6.03 (1H, dd, *J* = 3.5, 1.5 Hz, C**6**-H), 3.51 (2H, hept, 2 × C**2**-H), 2.49 (2H, dd, *J* = 9.5, 7.5 Hz, C**11**-H_2), 2.15 – 1.83 (2H, m, C**10**-H_2), 1.45 – 1.16 (18H, m, 4 × C**1**-H_3, 2 × C**9**-H_3); ^{13}C NMR (101 MHz, $CDCl_3$): δ 154.1 (C**3**), 143.4 (C**12**), 140.7 (C**7**), 128.6 (C**13**), 128.3 (C**14**), 125.5 (C**15**), 119.6 (C**4**), 107.7 (C**5**), 107.5 (C**6**), 44.7 (C**10**), 36.1 (C**8**), 31.7 (C**11**), 28.5 (C**9**), 20.6 (C**1**), 20.3 (C**1**); HRMS (ESI$^+$): Calculated for $C_{22}H_{32}N_2O$: 341.2587. Found [M+H]$^+$: 341.2587.

(8a): *N,N*-Diisopropyl-3-(2-phenylpropan-2-yl)thiophene-2-carboxamide

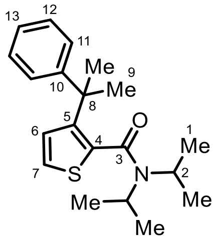

Modified General Procedure D: α-Methylstyrene (52.0 µL, 0.40 mmol, 400 mol%) was employed and the reaction was run at 140 °C for 48 h. Purification by flash column chromatography (hexane/EtOAc 10%) on silica gel afforded the title compound (19.8 mg, 60%) as a colorless oil. ν_{max}/cm^{-1}: 2968 (s), 2931 (m), 1623 (s), 1444 (s), 1316 (s); ^{1}H NMR (400 MHz, $CDCl_3$): δ 7.33 – 7.23 (4H, m, 2 × C**11**-H, 2 × C**12**-H), 7.16 (1H, m, C**13**-H), 7.06 (1H, d, *J* = 5.0 Hz, C**7**-H), 6.58 (1H, d, *J* = 5.0 Hz, C**6**-H), 4.02 (1H, m, 1 × C**2**-H), 3.44 (1H, m, 1 × C**2**-H), 1.72 (6H, s, 2 × C**9**-H_3), 1.53 – 1.09 (12H, m, 4 × C**1**-H_3); ^{13}C NMR (101 MHz, $CDCl_3$): δ 165.3 (C**3**), 150.3 (C**10**), 147.7 (C**5**), 132.0 (C**4**), 129.6 (C**6**), 128.1 (C**11**), 126.3 (C**12**), 125.8 (C**13**), 122.8 (C**7**), 42.7 (C**8**), 30.8 (C**9**), 20.0 (C**1**); *A signal corresponding to C***2** *was not observed*. HRMS (ESI$^+$): Calculated for $C_{20}H_{27}NOS$: 330.1886. Found [M+H]$^+$: 330.1885.

(8b): *N,N*-Diisopropyl-3-(2-methyl-4-phenylbutan-2-yl)thiophene-2-carboxamide

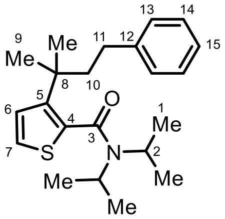

Modified General Procedure D: (3-methylbut-3-en-1-yl)benzene (58.5 mg, 0.40 mmol, 400 mol%) was employed and the reaction was run at 140 °C for 48 h. Purification of the residue by FCC (hexane/EtOAc 7%) afforded the title compound (30.8 mg, 86%) as a colorless oil. ν_{max}/cm^{-1}: 2967 (s), 1624 (s), 1444 (s), 1368 (s), 1316 (s); ^{1}H NMR (400 MHz, $CDCl_3$): δ 7.26 – 7.21 (2H, d, *J* = 8.5 Hz, 2 × C**14**-H), 7.19 (1H, d, *J* = 5.0 Hz, C**7**-H), 7.17 – 7.11 (3H, m, 2 × C**13**-H, C**15**-H), 6.98 (1H, d, *J* = 5.0 Hz, C**6**-H), 3.97 (1H, m, 1 × C**2**-H), 3.47 (1H, m, 1 × C**2**-H), 2.47 (2H, dd, *J* = 10.0, 7.5 Hz, C**11**-H_2), 2.00 (2H, m, C**10**-H_2) 1.51 (6H, s, 2 × C**1**-H_3), 1.41 (6H, s, 2 × C**9**-H_3), 1.18 (6H, s, 2 × C**1**-H_3); ^{13}C NMR (101 MHz, $CDCl_3$): δ 165.8 (C**3**), 146.3 (C**5**), 143.2 (C**12**), 132.4 (C**4**), 128.5 (C**13**), 128.4 (C**14**), 127.9 (C**6**), 125.6 (C**15**), 123.2 (C**7**), 51.4 (C**2**), 47.0 (C**10**), 46.1 (C**2**), 38.4 (C**8**), 31.6 (C**11**), 28.8 (C**9**), 20.2 (C**1**); HRMS (ESI^{+}): Calculated for $C_{22}H_{31}NOS$: 358.2199. Found $[M+H]^{+}$: 358.2196.

(8c): (*S*)-*N,N*-Diisopropyl-3-(2-(4-methylcyclohex-3-en-1-yl)propan-2-yl)thiophene-2-carboxamide

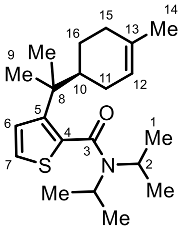

Modified General Procedure D: (*S*)-(-)-Limonene (64.6 µL, 0.40 mmol, 400 mol%) was employed and the reaction was run at 140 °C for 48 h. Purification of the residue by FCC (hexane/EtOAc 7%) afforded the title compound (18.6 mg, 54%) as a colorless oil. $[\alpha]_D^{24}$ – 6.53 (c = 0.022, $CHCl_3$); ν_{max}/cm^{-1}: 2965 (s), 2926 (s), 1626 (s), 1551 (m), 1444 (m), 1316 (s); ^{1}H NMR (400 MHz, $CDCl_3$): δ 7.13 (1H, m, C**7**-H), 6.93 (1H, d, *J* = 5.0 Hz, C**6**-H), 5.32 (1H, s, C**12**-H), 3.94 (1H, m, 1 × C**2**-H), 3.45 (1H, m, 1 × C**2**-H), 2.03 – 1.63 (6H, m, C**10**-H, 1 × C**11**-H, C**15**-H_2, C**16**-H_2), 1.60 (3H, s, C**14**-H_3), 1.54 – 1.35 (6H, m, 2 × C**1**-H_3), 1.30 (3H, s, 1 × C**9**-H_3), 1.25 (3H, s, 1 × C**9**-H_3), 1.22 – 1.11 (7H, m, 2 × C**1**-H_3, 1 × C**11**-H); ^{13}C NMR (101 MHz, $CDCl_3$): δ 165.7 (C**3**), 147.4 (C**5**), 133.9 (C**13**), 131.7 (C**4**), 128.0 (C**6**), 122.6 (C**7**), 121.4 (C**12**), 50.9 (C**2**), 46.1 (C**2**), 44.3 (C**10**), 40.6 (C**8**), 31.5 (C**15**), 27.1 (C**11**), 25.7 (C**9**), 24.6 (C**16**), 23.3 (C**14**), 19.9 (C**1**); *some of the ^{13}C signals were weak but could easily be identified by HSQC and HMBC analysis;* HRMS (ESI^{+}): Calculated for $C_{22}H_{33}NOS$: 348.2356. Found $[M+H]^{+}$: 348.2351.

Deuterium Labelling Experiments

Methyl-d_3-triphenylphosphonium iodide

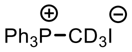

The title compound was prepared following a literature procedure.[20] To a suspension of PPh_3 (5.00 g, 19.1 mmol) in THF (33 mL) was added CD_3I (1.40 mL, 22.9 mmol). The mixture was heated to reflux for 1 h, before being cooled to ambient temperature. The resulting white solid was filtered and washed with benzene (2 × 20 mL) and dried under reduced pressure to afford the title compound (7.50 g, 97%) as a white solid. The material was used in the next step without further purification.

(*deuterio*-9):

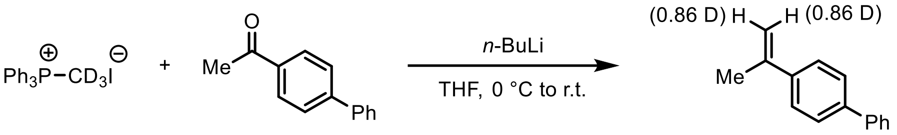

To a suspension of methyl-d_3-triphenylphosphonium iodide (2.75 g, 9.21 mmol) in THF (25 mL), under nitrogen, was added *n*-BuLi (5.76 mL, 9.21 mmol, 1.6 M in hexanes) dropwise at 0 ˚C. The resulting solution was stirred for 1 h, before 4-acetylbiphenyl (1.51 g, 7.67 mmol) in THF (25 mL) was added dropwise. The reaction was slowly warmed to ambient temperature and stirred for 4 h. NH_4Cl (10 mL) was added, and the mixture was extracted with EtOAc (3 × 10 mL). The organic extracts were combined, washed with brine (20 mL), dried over Na_2SO_4, filtered and concentrated *in vacuo*. Purification of the residue by FCC (hexane/EtOAc 5%) provided the title compound (600 mg, 40% yield, 86% deuteration) as a colorless solid. ^1H NMR (400 MHz, $CDCl_3$): δ 7.66 – 7.53 (6H, m), 7.50 – 7.40 (2H, m), 7.38 – 7.32 (1H, m), 5.45 – 5.42 (0.14H, m), 5.15 – 5.10 (0.14H, m), 2.20 (3H, s); ^2H NMR (61 MHz, $CHCl_3$): δ 5.48 (0.86D, s), 5.16 (0.86D, s); m.p. 120–122 °C ($CDCl_3$).

Deuterium Labelling Experiment

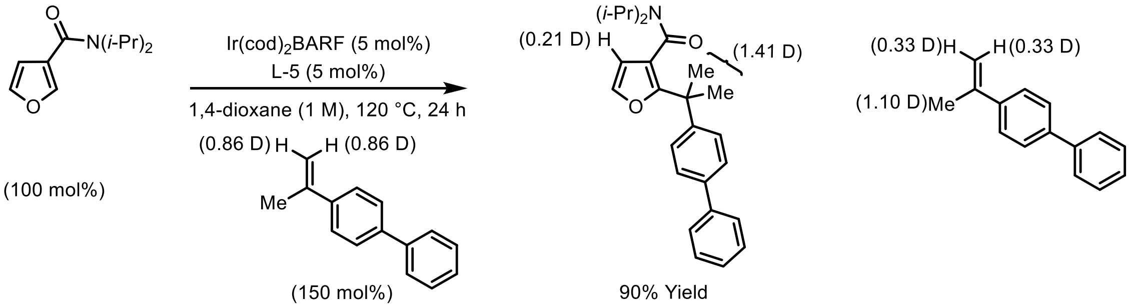

A flame-dried re-sealable tube, fitted with a magnetic stirrer, was charged with substrate **3a** (19.5mg, 0.1 mmol), $[Ir(cod)_2]BARF$ (5.0 mol%) and **L-5 (**5.0 mol%). The tube was fitted with a rubber septum and purged with nitrogen. *Deuterio*-**9** (29.4 mg, 0.15 mmol) in anhydrous 1,4-dioxane (0.1 mL) was

added and the tube was fitted with a Young's tap. The reaction mixture was then heated to 120 °C for 24 h before being cooled to ambient temperature and concentrated *in vacuo*. Purification of the residues by FCC (hexane/EtOAc 10–20%) afforded the *deuterio*-products.

(4i): 2-(2-([1,1'-Biphenyl]-4-yl)propan-2-yl)-*N,N*-diisopropylfuran-3-carboxamide

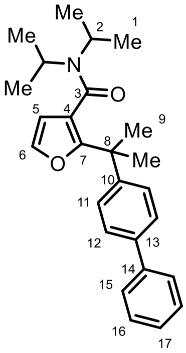

For comparison, the non-deuterated product was synthesized by **General Procedure D:** The reaction was carried out with styrene derivative (150 mol%) and was run for 24 h. Purification of the residue by FCC (hexane/EtOAc 0–20%) afforded the title compound (33.7 mg, 86%) as a colorless oil. ν_{max}/cm^{-1}: 2969 (m), 2927 (m), 1626 (s), 1438 (s); ^{1}H NMR (400 MHz, $CDCl_3$): δ 7.58 – 7.54 (2H, m, C**11**-H), 7.53 – 7.50 (2H, m, C**12**-H), 7.44 – 7.39 (2H, m, C**16**-H), 7.39 – 7.36 (2H, m, C**15**-H), 7.35 – 7.30 (1H, m, C**17**-H), 7.26 (1H, d, *J* = 2.0 Hz, C**6**-H), 6.24 (1H, d, *J* = 2.0 Hz, C**5**-H), 4.01 (1H, hept, *J* = 6.5 Hz, C**2**-H), 3.39 (1H, hept, *J* = 6.5 Hz, C**2**-H), 1.76 (6H, s, C**9**-H_3), 1.44 (6H, d, *J* = 6.5 Hz, C**1**-H_3), 1.09 (6H, d, *J* = 6.5 Hz, C**1**-H_3); ^{13}C NMR (101 MHz, $CDCl_3$): δ 166.2 (C**3**), 157.6 (C**7**), 147.1 (C**10**), 141.1 (C**14**), 140.2 (C**6**), 139.1 (C**13**), 128.8 (C**16**), 127.2 (C**11**), 127.2 (C**17**) 127.1 (C**12**), 126.6 (C**15**), 118.0 (C**4**), 109.7 (C**5**), 50.9 (C**2**), 45.8 (C**2**), 41.5 (C**8**), 28.5 (C**9**), 20.7 (C**1**), 20.4 (C**1**); HRMS: (ESI$^+$) calculated for $C_{26}H_{31}NO_2$ 390.2428. Found $[M+H]^+$ 390.2417.

The data for the deuterated products is presented below:

(*deuterio*-4i):

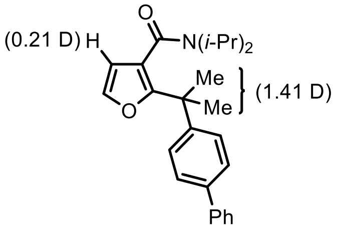

^{1}H NMR (400 MHz, $CDCl_3$): δ 7.57 – 7.53 (2H, m), 7.53 – 7.49 (2H, m), 7.44 – 7.39 (2H, m), 7.39 – 7.36 (2H, m), 7.34 – 7.29 (1H, m), 7.26 (1H, d, *J* = 2.0 Hz), 6.23 (0.79H, d, *J* = 2.0 Hz), 4.01 (1H, hept, *J* = 6.5 Hz), 3.38 (1H, hept, *J* = 6.5 Hz), 1.82 – 1.68 (4.59H, m), 1.44 (6H, d, *J* = 6.5 Hz), 1.08 (6H, d,

J = 6.5 Hz); ^{2}H NMR (77 MHz, $CHCl_3$): δ 6.28 (0.21D, s), 1.74 (1.41D, s). *Deuterium incorporation was calculated by integration of both ^{1}H NMR and ^{2}H NMR signals.*

(*deuterio*-9'):

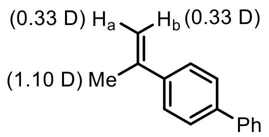

^{1}H NMR (400 MHz, $CDCl_3$): δ 7.70 – 7.53 (6H, m), 7.49 – 7.41 (2H, m), 7.40 – 7.31 (1H, m), 5.48 – 5.41 (0.67H, m, H_b), 5.15 – 5.06 (0.67H, m, H_a), 2.25 – 2.16 (1.9H, m); ^{2}H NMR (77 MHz, $CHCl_3$): δ 5.49 (0.33D, s), 5.16 (0.33D, s), 2.19 (1.10D, d, *J* = 2.5 Hz).

^{13}C KIE Determination Experiments

Procedure for large scale reactions:

An oven-dried re-sealable tube, fitted with a magnetic stirrer, was charged with furan substrate **3a** (391 mg, 2.00 mmol), **9** (389 mg, 2.00 mmol), $[Ir(cod)_2]$BARF (0.10 mmol, 5 mol%) and **L-5** (0.10 mmol, 5 mol%). The tube was fitted with a rubber septum and purged with nitrogen. Anhydrous 1,4-dioxane (2.0 mL) was added *via* syringe and the tube was sealed with a Young's tap. The reaction vessel was placed into a pre-heated heating block at 120 °C and stirred for 6 h. The reaction mixture was cooled to ambient temperature and concentrated *in vacuo*. The crude mixture was transferred into a 20 mL volumetric flask, which was previously charged with a known amount of internal standard (1,3,5-trimethoxybenzene, ~85 mg), and the flask was filled with $CDCl_3$, pre-treated over anhydrous K_2CO_3. Six aliquots of 0.4 mL each were taken from the solution and transferred into six NMR tubes, which were subsequently diluted with additional 0.3 mL of pre-treated $CDCl_3$ each. A ^{1}H NMR spectrum was recorded for each sample employing a 500 MHz instrument, using the following parameters: 16 scans, π/2 pulse, 6.5 s acquisition time and 40 s relaxation delay. The conversion of the alkene starting material (**F**) was determined by integration of the C**1**-$\underline{H}_b$ signal of **9** against the aromatic C-$\underline{H}$ signal of the internal standard. The remaining crude material was purified by FCC to recover unreacted **9**.

Quantitative ^{13}C NMR analysis: All NMR samples were prepared employing ~100 mg of recovered **9** in 0.7 mL of pre-treated $CDCl_3$. The ^{13}C NMR spectra were recorded at 126 MHz using inverse gated decoupling and employing a 500 MHz instrument equipped with a CryoProbe™. The spectra were recorded according to the following parameters: 1024 scans, π/6 pulse, 15 s relaxation delay. A total of five spectra were recorded for each sample. The resulting five FIDs were processed at the same time applying the same phase correction, a fifteenth order polynomial fit baseline correction and 256K zero filling. Integrations were numerically determined using a constant region for each peak corresponding

to eight times of the peak widths at half height (± $8w_{1/2}$). The peak belonging to C**8** of **9** was chosen as the internal standard and was set with an integration of 1000.

Formulas applied for the determination of ^{13}C KIEs: The formulas employed in the calculations for the determination of the KIE were reported by Saunders[21] and Singleton[22] and are summarized as follows:

F = conversion of starting material.

R/R_0 = proportion of the minor isotopic component in recovered material compared to the original starting material.

$\Delta(R/R_0)$ = $R/R_0((\Delta R/R)^2 + (\Delta R_0/R_0)^2)^{1/2}$

KIE = $\frac{\ln(1-F)}{\ln[(1-F)R/R_0]}$

ΔKIE_F = $\frac{\partial KIE}{\partial F}\Delta F = \frac{-ln(R/R_0)}{(1-F)ln^2[(1-F)R/R_0]}\Delta F$

ΔKIE_R = $\frac{\partial KIE}{\partial(R/R_0)}\Delta(R/R_0) = \frac{-ln(1-F)}{(R/R_0)ln^2[(1-F)R/R_0]}\Delta R/R_0$

ΔKIE = $KIE*((\Delta KIE_R/KIE)^2 + (\Delta KIE_F/KIE)^2)^{1/2}$

^{13}C KIE determination for the hydroarylation of 3a with 9:

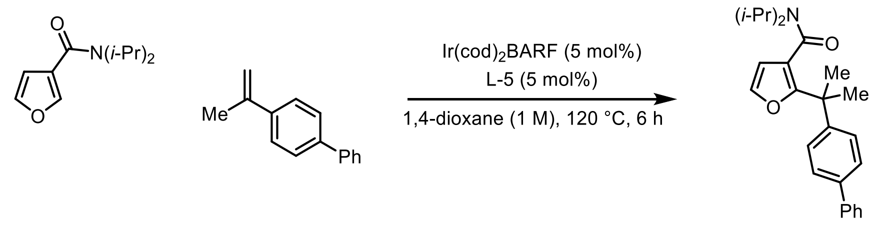

3a, (100 mol%) **9**, (100 mol%) **4i**

^{1}H NMR analysis provided a conversion of 59.7 ± 0.2% for the first experiment and of 64.0 ± 0.4% for the second experiment. Purification of the crude mixture by FCC (hexane/EtOAc 0–5%) afforded 128 mg (38% recovered) for the first sample and 122 mg (36% recovered) for the second sample.

Tables are reported on the next pages.

First experiment:

Conversion (F)						
**fid1**	**fid2**	**fid3**	**fid4**	**fid5**	**F**	**ΔF**
59.7	60.0	59.4	59.7	59.7	**59.7**	**0.2**

^{13}C-NMR integration of alkene starting material (R_0)							
**ppm peaks**	**fid1**	**fid2**	**fid3**	**fid4**	**fid5**	**R_0**	**ΔR_0**
**142.8 (C2)**	1037.3	1037.5	1040.8	1040.4	1040.7	**1039.3**	**1.8**
**140.2 (C7/4)**	2052.9	2054.5	2056.9	2058.0	2057.4	**2055.9**	**2.2**
**112.6 (C1)**	900.7	899.0	899.0	901.4	899.8	**900.0**	**1.1**
**21.9 (C3)**	955.7	956.5	957.3	957.7	957.9	**957.0**	**0.9**
**140.8 (C8)**	1000.0	1000.0	1000.0	1000.0	1000.0	**1000.0**	**0.0**

^{13}C-NMR integration of alkene from 59.7 ± 0.2 % conversion reaction (R)							
**ppm peaks**	**fid1**	**fid2**	**fid3**	**fid4**	**fid5**	**R**	**ΔR**
**142.8 (C2)**	1052.1	1051.8	1052.3	1051.5	1051.4	**1051.8**	**0.4**
**140.2 (C7/4)**	2058.1	2057.8	2057.3	2058.8	2056.7	**2057.7**	**0.8**
**112.6 (C1)**	927.1	927.6	928.3	927.3	928.7	**927.8**	**0.7**
**21.9 (C3)**	938.2	938.7	938.0	938.6	937.4	**938.2**	**0.5**
**140.8 (C8)**	1000.0	1000.0	1000.0	1000.0	1000.0	**1000.0**	**0.0**

Determination of ^{13}C KIEs						
**ppm peaks**	**R/R_0**	**$\Delta(R/R_0)$**	**ΔKIE_F**	**ΔKIE_R**	**KIE**	**ΔKIE**
**142.8 (C2)**	1.012008	0.001771	- 0.000078	0.001977	1.013308	0.001978
**140.2 (C7/4)**	1.000876	0.001119	- 0.000006	0.001233	1.000964	0.001233
**112.6 (C1)**	1.030912	0.001428	- 0.000208	0.001632	1.034659	0.001645

**21.9 (C3)**	0.980314	0.001082	0.000121	0.001163	0.978591	0.001169
**140.8 (C8)**	1	0	0	0	1	0

**Output**	
**C**	**KIE**
C2	**1.013 ± 0.002**
**C7/4**	**1.001 ± 0.001**
C1	**1.035 ± 0.002**
C3	**0.979 ± 0.001**

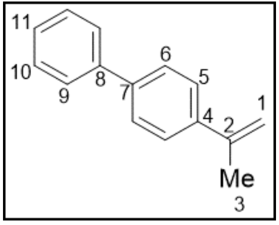

Second experiment:

**Conversion (F)**						
**fid1**	**fid2**	**fid3**	**fid4**	**fid5**	**F**	**ΔF**
64.4	63.6	64.1	64.1	63.6	**64.0**	**0.4**

**^{13}C-NMR integration of alkene SM (R_0)**							
**ppm peaks**	**fid1**	**fid2**	**fid3**	**fid4**	**fid5**	**R_0**	**ΔR_0**
**142.8 (C2)**	1037.3	1037.5	1040.8	1040.4	1040.7	**1039.3**	**1.8**
**140.2 (C7/4)**	2052.9	2054.5	2056.9	2058.0	2057.4	**2055.9**	**2.2**
**112.6 (C1)**	900.7	899.0	899.0	901.4	899.8	**900.0**	**1.1**
**21.9 (C3)**	955.7	956.5	957.3	957.7	957.9	**957.0**	**0.9**
**140.8 (C8)**	1000.0	1000.0	1000.0	1000.0	1000.0	**1000.0**	**0.0**

**^{13}C-NMR integration of alkene from 64.0 ± 0.4 % conversion reaction (R)**							
**ppm peaks**	**fid1**	**fid2**	**fid3**	**fid4**	**fid5**	**R**	**ΔR**
**142.8 (C2)**	1053.1	1052.1	1052.3	1054.3	1054.1	**1053.2**	**1.0**
**140.2 (C7/C4)**	2060.6	2062.7	2064.9	2068.0	2066.7	**2064.6**	**3.0**
**112.6 (C1)**	929.9	931.3	930.3	931.1	931.7	**930.9**	**0.7**
**21.9 (C3)**	940.3	941.2	939.0	940.4	940.8	**940.3**	**0.8**

**140.8 (C8)**	1000.0	1000.0	1000.0	1000.0	1000.0	**1000.0**	**0.0**

**Determination of ^{13}C KIEs**						
**ppm peaks**	**R/R_0**	**$\Delta(R/R_0)$**	**ΔKIE_F**	**ΔKIE_R**	**KIE**	**ΔKIE**
**142.8 (C2)**	1.013316	0.001986	-0.000127	0.001971	1.013132	0.001975
**140.2 (C7/4)**	1.004202	0.001794	-0.000040	0.001765	1.004126	0.001766
**112.6 (C1)**	1.034312	0.001469	-0.000337	0.001489	1.034187	0.001526
**21.9 (C3)**	0.982571	0.001276	0.000159	0.001230	0.983063	0.001240
**140.8 (C8)**	1	0	0	0	1	0

**Output**	
**C**	**KIE**
C2	**1.013 ± 0.002**
**C7/4**	**1.004 ± 0.002**
C1	**1.034 ± 0.002**
C3	**0.983 ± 0.001**

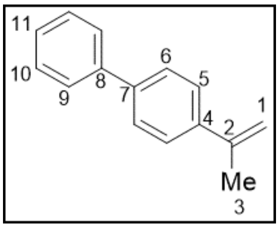

^{13}C KIE determination for 9 under Ir(I) catalysis

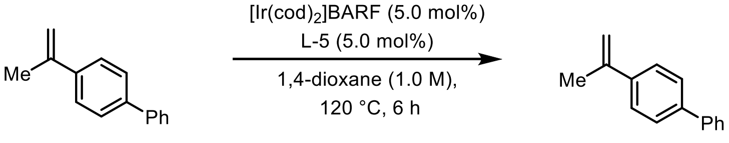

9, (100 mol%)

The reaction was carried out on a 1.00 mmol scale, in the absence of furan **3a**. Purification of the crude mixture by FCC (hexane/EtOAc 0–1%) afforded 193 mg (97% recovered).

**^{13}C-NMR integration of alkene SM (R_0)**								
**ppm peaks**	**fid1**	**fid2**	**fid3**	**fid4**	**fid5**	**fid6**	**R_0**	**ΔR_0**
**142.8 (C2)**	1038.3	1042.3	1046.1	1047.6	1041.8	1039.7	**1042.6**	**3.6**

**140.2 (C7/4)**	2058.6	2061.6	2067.7	2070.1	2062.7	2061.2	**2063.7**	**4.4**
**112.6 (C1)**	892.3	896.8	902.4	907.6	905.5	905.0	**901.6**	**5.9**
**21.9 (C3)**	957.5	959.0	961.1	961.4	959.6	959.4	**959.7**	**1.4**
**140.8 (C8)**	1000.0	1000.0	1000.0	1000.0	1000.0	1000.0	**1000.0**	**0.0**

^{13}C-NMR integration of recovered alkene

ppm peaks	fid1	fid2	fid3	fid4	fid5	fid6	R	ΔR
**142.8 (C2)**	1040.6	1043.6	1043.0	1041.5	1043.5	1042.7	**1042.5**	**1.2**
**140.2 (C7/4)**	2057.0	2057.9	2059.0	2057.6	2060.7	2060.9	**2058.9**	**1.6**
**112.6 (C1)**	918.2	920.3	920.5	919.5	918.9	919.7	**919.5**	**0.9**
**21.9 (C3)**	942.3	942.8	944.4	945.3	946.2	946.9	**944.7**	**1.8**
**140.8 (C8)**	1000.0	1000.0	1000.0	1000.0	1000.0	1000.0	**1000.0**	**0.0**

ppm peaks	R/R_0	$\Delta(R/R_0)$
**142.8 (C2)**	0.999856	0.003639
**140.2 (C7/4)**	0.997674	0.002249
**112.6 (C1)**	1.019872	0.006721
**21.9 (C3)**	0.984352	0.002415
**140.8 (C8)**	1	0

Output

C	KIE
C2	**1.000 ± 0.004**
**C7/4**	**0.998 ± 0.002**
C1	**1.020 ± 0.007**
C3	**0.984 ± 0.002**

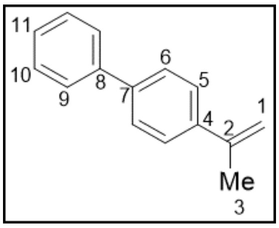

Comparison of the relative ratio of ^{13}C at C1:C3 depending upon method of synthesis of 9:

As described earlier, alkene **9** can be made via either Wittig methylenation or elimination of the corresponding benzylic alcohol. The former method was used for the experiments outlined in Scheme 2 of the main paper. As can be seen from the data below, the relative ratio of carbon-13 at C1:C3 varies depending on the method used. The implications of this on KIE data analysis are discussed in the main paper.

**^{13}C-NMR integration of alkene prepared by methylenation (R_0)**							
**ppm peaks**	**fid1**	**fid2**	**fid3**	**fid4**	**fid5**	**fid6**	**R_0**
**142.8 (C2)**	1038.3	1042.3	1046.1	1047.6	1041.8	1039.7	**1042.6**
**140.2 (C7/4)**	2058.6	2061.6	2067.7	2070.1	2062.7	2061.2	**2063.7**
**112.6 (C1)**	892.3	896.8	902.4	907.6	905.5	905.0	**901.6**
**21.9 (C3)**	957.5	959.0	961.1	961.4	959.6	959.4	**959.7**
**140.8 (C8)**	1000.0	1000.0	1000.0	1000.0	1000.0	1000.0	**1000.0**

**^{13}C-NMR integration of alkene prepared by Grignard addition & elimination (R)**							
**ppm peaks**	**fid1**	**fid2**	**fid3**	**fid4**	**fid5**	**fid6**	**R**
**142.8 (C2)**	1025.4	1023.1	1020.4				**1022.9**
**140.2 (C7/4)**	2110.2	2108.3	2101.6				**2106.7**
**112.6 (C1)**	930.4	930.9	929.2				**930.2**
**21.9 (C3)**	936.4	935.8	932.3				**934.9**
**140.8 (C8)**	1000.0	1000.0	1000.0				**1000.0**

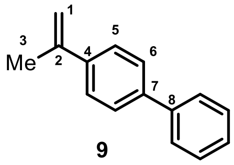

9

**Route to 9**	**Rel. ratio of ^{13}C at C1:C3**
Methylenation	0.939
Elimination	0.995

The values of 0.939 and 0.995 were obtained as follows:

(901.6/959.7) = 0.939 and (930.2/934.9) = 0.995.

These values indicate that the relative ratio of ^{13}C at C1:C3 is different depending upon the synthetic route to prepare alkene ***9****.*

NMR profiling Experiments

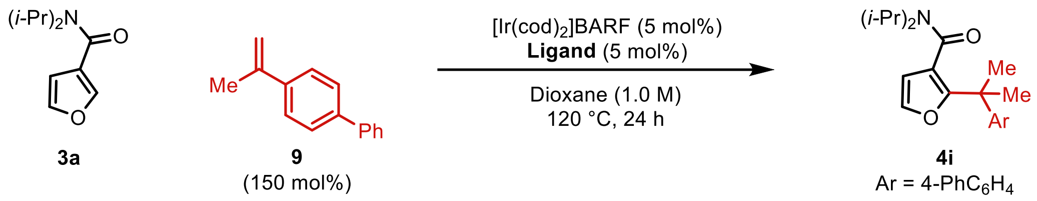

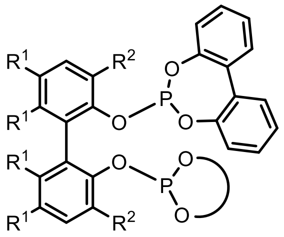

Entry		Ligand	Yield of 300e
1	◆	**L-5**, R^1=Me, R^2=*t*-Bu	86%
2	■	**L-6**, R^1=Me, R^2=Me	76%
3	▲	**L-7**, R^1=Me, R^2=H	19%
4	◇	**L-8**, R^1 = R^2 = H	12%

Time (h)	Conversion to 4i for ligands L-5-8 (%)			
	**L-5**	**L-6**	**L-7**	**L-8**
**2**	11	6	1.3	1.6
**3**	13	11	2.4	3.1
**6**	69	34	4.7	4.2
**9**	80	54	4.8	7.3
**12**	86	77	14	7.3
**24**	86	76	19	12

The data in the table above was obtained by measuring the conversion of **3a** to **4i** at various time intervals. The conversion was measured by ^{1}H NMR analysis against 1,4-dinitrobenzene as an internal standard.

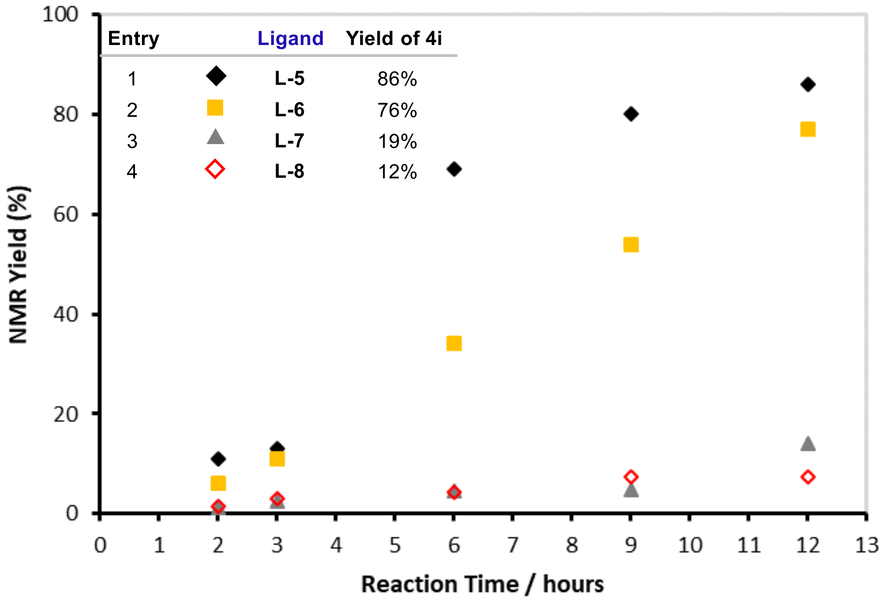

Computational Details

Possible conformations of the intermediate **III-L-8** and transition state (TS) **TS-III-L-8** were generated manually, accounting for eight and ten conceivable arrangements of the ligand, hydride, and substrates, respectively, including those identified as the lowest in energy in previous DFT studies of carbometallation on a similar system.[23] All conformations were subsequently optimized by DFT calculations carried out using Gaussian16 (Revision A.03)[24] with the B3LYP density functional,[25,26] the SDD[27] ECP for Ir, and the split-valence polarized 6-31G(d) basis set[28] for all other atoms. Single point energy (SPE) calculations were used to correct the Gibbs free energies derived from the original B3LYP calculations.[29] These were performed using B3LYP/SDD(Ir) and the polarized triple-ζ valence quality (def2-TZVPP) basis set[30] for all other atoms. The integral equation formalism version of the polarizable continuum model (IEFPCM)[31] (dioxane) was used to incorporate the effect of the dioxane solvent. An intrinsic reaction coordinate analysis (IRC) was performed on the lowest energy conformation of **TS-III-L-8** using the same methods, confirming that the TS corresponds to the intended carbometallation step; energies and coordinates for the reactant and product complexes resulting from each direction of this analysis are included below. The lowest energy conformations **III-L-8** and **TS-III-L-8** were subsequently reoptimized with their R groups varied to obtain conformations for the intermediates **III-L-5-7** and transition state structures **TS-III-L-5-7**. All DFT calculations were performed using an ultrafine integration grid. All temperature (393.15 K) and concentration-corrected (1 mol/l) quasiharmonic (Grimme approximation[32]) free energies were calculated with GoodVibes.[33] Similar methods have been used previously in the successful modelling of ruthenium-catalyzed reactions using diphosphine ligands.[34,35]

Energies and molecular geometries of all computed structures

All energies in Hartrees, coordinates in Å. Cartesian coordinates generated by ESIgen software.[36] For each species, conformations are ordered from lowest energy (first) to highest energy (last).

III-L-5 Conformation 1

B3LYP/6-31G(d),SDD(Ir) Energy = -3922.747597
B3LYP/def2-TZVPP,SDD(Ir)/IEFPCM(dioxane) Energy = -3924.093969
B3LYP/def2-TZVPP,SDD(Ir)/IEFPCM(dioxane)//B3LYP/6-31G(d),SDD(Ir) Free Energy (Quasiharmonic) = -3923.067026
Number of Imaginary Frequencies = 0

Frequencies (Top 3 out of 423)

1. 8.6494 cm^{-1}
2. 17.4681 cm^{-1}
3. 21.7027 cm^{-1}

B3LYP/6-31G(d),SDD(Ir) Molecular Geometry in Cartesian Coordinates

O	-2.275421	-1.673545	0.867066
C	-1.693480	-1.724525	2.148658
C	-2.447254	-1.277673	3.251148
C	-0.426035	-2.325357	2.272909
C	-1.754127	-1.269428	4.468837
C	0.217010	-2.320113	3.532529
C	-0.443040	-1.727756	4.625928
H	-2.258030	-0.902644	5.354614
C	0.119881	-3.152145	1.143322
C	1.358574	-2.849486	0.533609
C	-0.567264	-4.330725	0.779241
C	2.037721	-3.768061	-0.292725
C	-0.024246	-5.140390	-0.235599
C	1.250731	-4.854825	-0.711317

H	1.678912	-5.550272	-1.424770
O	1.939571	-1.610089	0.898684
P	1.802361	-0.162437	0.166009
P	-1.672056	-0.831651	-0.397498
O	2.756801	-0.439489	-1.133718
O	2.741510	0.733905	1.132935
O	-3.030064	-0.237316	-1.075685
O	-1.340591	-2.090263	-1.394033
C	3.485409	0.568576	-1.773825
C	3.132614	0.882715	-3.083363
C	4.589011	1.154614	-1.130752
C	3.882639	1.820902	-3.790950
H	2.281188	0.382155	-3.532866
C	5.319806	2.102642	-1.869470
C	4.977042	2.434983	-3.178293
H	3.613142	2.067735	-4.813454
H	6.165112	2.592764	-1.395909
H	5.565391	3.170379	-3.718918
C	4.122561	0.551088	1.301314
C	4.557160	0.229763	2.584337
C	5.016624	0.786936	0.242465
C	5.921809	0.127366	2.846132
H	3.818698	0.072026	3.363536
C	6.387716	0.665378	0.539079
C	6.839310	0.344297	1.816185
H	6.263204	-0.124837	3.845477
H	7.106169	0.807251	-0.262286
H	7.904882	0.255200	2.004158
C	-4.023656	-1.006887	-1.697770
C	-5.300686	-0.966711	-1.142733
C	-3.758184	-1.678135	-2.902969
C	-6.351866	-1.623147	-1.781016
H	-5.458376	-0.413329	-0.224223
C	-4.837946	-2.335508	-3.519918
C	-6.117993	-2.312217	-2.972557
H	-7.346788	-1.595652	-1.346839
H	-4.654695	-2.886760	-4.437283
H	-6.928204	-2.834880	-3.471725
C	-1.247115	-1.891384	-2.774944
C	0.009062	-1.994697	-3.368997
C	-2.416080	-1.698472	-3.529143
C	0.123820	-1.883406	-4.754672
H	0.872808	-2.187575	-2.742802
C	-2.267025	-1.578973	-4.922564
C	-1.016998	-1.666106	-5.531199
H	1.099426	-1.973933	-5.223443
H	-3.151079	-1.406338	-5.529011
H	-0.934941	-1.570916	-6.609764
Ir	-0.098549	0.955430	-0.297017
C	-0.181768	2.804185	2.319250
C	-0.737761	1.565105	2.142074
C	0.844469	2.715720	-0.725376
H	-0.255631	0.697727	2.578927
H	-1.784896	1.458434	1.892664
C	0.076052	3.728346	-1.292359
O	2.121282	3.146095	-0.627857
C	-1.327708	3.396764	-1.392508
C	0.962237	4.838515	-1.553068
C	2.184062	4.429255	-1.134328
O	-1.685418	2.252380	-0.914039
N	-2.271385	4.180179	-1.938709
H	0.739524	5.805542	-1.976312
H	3.161252	4.885690	-1.121693
C	-1.973796	5.525434	-2.429814
C	-3.672643	3.755885	-1.944834
H	-2.871012	5.920571	-2.908349
H	-1.692917	6.196498	-1.610951
H	-1.174068	5.500687	-3.174699
H	-3.727039	2.692575	-1.721103
H	-4.244143	4.315229	-1.194026
H	-4.102186	3.946020	-2.933041
H	0.156486	0.584079	-1.802772
C	-0.918166	4.062543	2.052429
C	-0.230435	5.288377	1.953128
C	-2.323898	4.089375	1.946797
C	-0.913640	6.485732	1.747156
H	0.850858	5.310676	2.026745
C	-3.007943	5.285640	1.747411
H	-2.893066	3.173008	2.048942
C	-2.306214	6.490673	1.646054
H	-0.357504	7.416245	1.677584
H	-4.093164	5.280910	1.693783
H	-2.841842	7.425591	1.506421
C	1.211355	2.921528	2.885686
H	1.211354	3.581700	3.760729
H	1.598363	1.945035	3.177112
H	1.907638	3.343330	2.152595
C	0.206226	-1.648705	5.990019
H	-0.412929	-1.072674	6.683891
H	0.355070	-2.642888	6.431079
H	1.192501	-1.171528	5.946405
C	1.568275	-2.966677	3.736865
H	1.641741	-3.426735	4.726348
H	1.765201	-3.740187	2.992709
H	2.379597	-2.230653	3.659570
C	-1.863071	-4.751427	1.433584
H	-2.728533	-4.438898	0.835035
H	-1.914536	-5.839944	1.529842
H	-1.982046	-4.324131	2.430165
C	-0.761515	-6.350036	-0.762658
H	-0.865169	-7.133074	-0.000298
H	-1.774274	-6.093082	-1.094592
H	-0.231239	-6.789013	-1.612858
C	-3.955495	-0.926899	3.194549
C	3.541237	-3.806186	-0.691049
C	4.442559	-2.912811	0.183310
H	5.490904	-3.135356	-0.045494
H	4.288035	-1.854127	0.002971
H	4.287961	-3.108469	1.250021
C	3.722346	-3.439272	-2.181553
H	3.411793	-2.410493	-2.379916
H	4.776726	-3.535652	-2.467747
H	3.141143	-4.107084	-2.828206
C	4.071401	-5.253570	-0.481527
H	3.909959	-5.590402	0.548526
H	3.614639	-5.985794	-1.152384
H	5.149220	-5.270804	-0.676893
C	-4.733931	-2.111566	2.568232
H	-4.573805	-3.027744	3.147790
H	-5.808929	-1.895055	2.579856
H	-4.438227	-2.304070	1.535481
C	-4.226429	0.364895	2.391217
H	-3.833970	0.319169	1.373688
H	-5.307500	0.540585	2.325952
H	-3.787788	1.231438	2.899189
C	-4.540247	-0.704123	4.606860
H	-4.416620	-1.584019	5.247094
H	-4.093582	0.158846	5.113351
H	-5.614502	-0.508555	4.520410

III-L-5 Conformation 2

B3LYP/6-31G(d),SDD(Ir) Energy = -3922.711258
B3LYP/def2-TZVPP,SDD(Ir)/IEFPCM(dioxane) Energy = -3924.053317
B3LYP/def2-TZVPP,SDD(Ir)/IEFPCM(dioxane)//B3LYP/6-31G(d),SDD(Ir) Free Energy (Quasiharmonic) = -3923.023944
Number of Imaginary Frequencies = 0

Frequencies (Top 3 out of 423)

1. 12.9193 cm^{-1}
2. 16.9645 cm^{-1}
3. 24.2672 cm^{-1}

B3LYP/6-31G(d),SDD(Ir) Molecular Geometry in Cartesian Coordinates

O	-2.136440	1.115020	0.967010

C	-1.372410	1.856300	1.879580
C	-1.720279	3.219770	2.035440
C	-0.419810	1.229070	2.701980
C	-0.961819	3.937070	2.963810
C	0.295920	2.001900	3.655540
C	0.041511	3.378010	3.756530
H	-1.173279	4.989050	3.105600
C	-0.227280	-0.260510	2.795600
C	1.018310	-0.820940	2.440620
C	-1.139361	-1.022520	3.558050
C	1.510059	-2.011421	3.010500
C	-0.746081	-2.302940	3.991720
C	0.564139	-2.727440	3.761360
H	0.861029	-3.666310	4.212870
O	1.852600	-0.044051	1.618750
P	1.779610	0.125319	-0.006930
P	-1.946280	-0.077340	-0.122120
O	3.327460	-0.395601	-0.254720
O	1.955600	1.735159	-0.233840
O	-2.983500	0.388811	-1.305700
O	-2.849871	-1.183969	0.686910
C	4.188910	0.054809	-1.248180
C	4.621649	-0.874911	-2.192680
C	4.693160	1.367699	-1.215300
C	5.555080	-0.498842	-3.156850
H	4.232119	-1.886181	-2.149370
C	5.622480	1.720928	-2.211950
C	6.049140	0.807378	-3.172590
H	5.894399	-1.223942	-3.890680
H	6.006970	2.736278	-2.228900
H	6.768220	1.113178	-3.926450
C	3.005620	2.494339	0.312800
C	2.661071	3.464869	1.249360
C	4.318030	2.345939	-0.165610
C	3.647091	4.310839	1.754930
H	1.627031	3.549079	1.562650
C	5.291901	3.206268	0.376020
C	4.967881	4.174748	1.321910
H	3.382651	5.070509	2.484440
H	6.322301	3.093758	0.053210
H	5.745161	4.819218	1.721220
C	-4.382230	0.455361	-1.257110
C	-4.962030	1.675021	-1.603240
C	-5.162980	-0.689309	-1.023540
C	-6.348020	1.795212	-1.672720
H	-4.316980	2.516681	-1.820020
C	-6.561250	-0.529968	-1.079600
C	-7.152490	0.689052	-1.395090
H	-6.793660	2.749352	-1.937940
H	-7.188451	-1.388218	-0.859950
H	-8.234440	0.773332	-1.426370
C	-3.477501	-2.253629	0.046230
C	-3.033781	-3.536449	0.355140
C	-4.594511	-2.033979	-0.775400
C	-3.679592	-4.644049	-0.189190
H	-2.191901	-3.635670	1.026340
C	-5.211721	-3.171559	-1.330760
C	-4.764982	-4.459079	-1.048540
H	-3.338672	-5.646229	0.055480
H	-6.053591	-3.031428	-2.001480
H	-5.266312	-5.313699	-1.492670
Ir	-0.022631	-0.910710	-1.259670
C	0.382690	1.420380	-3.142930
C	-0.453680	0.289450	-3.168140
C	1.172259	-2.124181	-2.364420
C	1.810820	1.301929	-3.587580
C	-0.128349	2.786860	-2.961510
H	-1.523060	0.458260	-3.113910
H	-0.153520	-0.486250	-3.866990
C	1.369769	-3.429171	-1.934360
O	1.756329	-1.972841	-3.575940
H	2.139040	0.267239	-3.654300
H	1.877340	1.758359	-4.586230
H	2.498490	1.862219	-2.951210
C	-1.403809	3.115990	-3.472700
C	0.696741	3.835420	-2.499110
C	0.722299	-3.710360	-0.675690
C	2.152489	-4.098581	-2.951030
C	2.355169	-3.173291	-3.918740
C	-1.818429	4.442610	-3.552820
H	-2.043320	2.335900	-3.870120
C	0.265291	5.156300	-2.551660
H	1.671001	3.612779	-2.081470
O	0.133009	-2.722470	-0.081970
N	0.694098	-4.928020	-0.102540
H	2.524458	-5.110971	-2.974010
H	2.870649	-3.183831	-4.866420
C	-0.986049	5.466040	-3.094230
H	-2.785629	4.678721	-3.986550
H	0.910661	5.948130	-2.183200
C	1.268608	-6.104921	-0.755720
C	0.093778	-5.145130	1.211720
H	-1.308788	6.501100	-3.161110
H	0.934938	-6.990890	-0.213210
H	0.919788	-6.188770	-1.787400
H	2.364348	-6.082641	-0.739080
H	-0.125301	-4.188110	1.677330
H	-0.824712	-5.736790	1.119180
H	0.798998	-5.693550	1.845320
H	-1.176231	-1.705520	-2.039670
C	-1.686311	-3.180310	4.786560
H	-1.225441	-4.146190	5.014770
H	-1.970001	-2.717800	5.740580
H	-2.619721	-3.371389	4.242570
C	-2.491840	-0.491079	3.971870
H	-3.293591	-1.038729	3.461760
H	-2.651260	-0.615909	5.049260
H	-2.615720	0.565551	3.736860
C	1.307610	1.365199	4.584540
H	1.474630	1.983029	5.468840
H	0.986600	0.378770	4.926690
H	2.277240	1.238409	4.086910
C	0.784891	4.267790	4.730180
H	0.498741	5.314840	4.591640
H	0.568251	4.007140	5.773910
H	1.871701	4.197569	4.603790
C	-2.896779	3.909661	1.312310
C	2.986849	-2.493381	2.960600
C	3.345019	-3.127311	1.598170
H	3.221589	-2.430611	0.769790
H	4.394559	-3.446801	1.606930
H	2.730859	-4.013511	1.407470
C	3.956929	-1.323061	3.260660
H	4.983629	-1.704972	3.301440
H	3.918830	-0.542921	2.500790
H	3.732389	-0.871991	4.234070
C	3.257079	-3.567151	4.041450
H	4.324669	-3.810861	4.038240
H	2.999639	-3.215321	5.046420
H	2.716788	-4.502141	3.854610
C	-4.220129	3.169421	1.628510
H	-4.423099	3.198841	2.705520
H	-5.055089	3.664601	1.119000
H	-4.205880	2.126321	1.312850
C	-2.620479	3.960001	-0.202170
H	-1.715849	4.542060	-0.404490
H	-2.483839	2.967731	-0.630250
H	-3.453279	4.448291	-0.722950
C	-3.092259	5.370841	1.775820
H	-3.305449	5.442581	2.847820
H	-2.225109	6.001850	1.550410
H	-3.950099	5.796091	1.243650

III-L-6 Conformation 1

B3LYP/6-31G(d),SDD(Ir) Energy = -3686.904881
B3LYP/def2-TZVPP,SDD(Ir)/IEFPCM(dioxane) Energy = -3688.163525

B3LYP/def2-TZVPP,SDD(Ir)/IEFPCM(dioxane)//B3LYP/6-31G(d),SDD(Ir) Free Energy (Quasiharmonic) = -3687.298689
Number of Imaginary Frequencies = 0

Frequencies (Top 3 out of 369)

1. 10.6718 cm^{-1}
2. 18.3991 cm^{-1}
3. 19.7628 cm^{-1}

B3LYP/6-31G(d),SDD(Ir) Molecular Geometry in Cartesian Coordinates

O	-2.789214	-0.344015	1.131984
C	-2.252891	-0.871464	2.315780
C	-2.415287	-0.122010	3.483316
C	-1.681148	-2.150523	2.297407
C	-1.886231	-0.667574	4.655199
C	-1.148544	-2.670632	3.498911
C	-1.237965	-1.905903	4.682725
H	-1.987087	-0.106312	5.581335
C	-1.788611	-2.997825	1.063077
C	-0.671371	-3.249057	0.248016
C	-3.014482	-3.631376	0.763764
C	-0.682691	-4.209946	-0.770274
C	-3.081720	-4.518037	-0.331122
C	-1.918780	-4.806304	-1.043119
H	-1.962768	-5.546458	-1.839172
O	0.536361	-2.599115	0.584857
P	1.251610	-1.317331	-0.113377
P	-1.910594	0.273266	-0.095509
O	1.564415	-1.921493	-1.600195
O	2.689506	-1.386615	0.629039
O	-2.706140	1.652901	-0.433364
O	-2.462805	-0.732766	-1.265433
C	2.694569	-1.555102	-2.340610
C	2.492029	-0.851709	-3.524667
C	3.968358	-1.981630	-1.928984
C	3.584851	-0.541422	-4.333245
H	1.483132	-0.563882	-3.801396
C	5.050028	-1.648319	-2.762902
C	4.867133	-0.938229	-3.947777
H	3.432900	0.006218	-5.258540
H	6.050302	-1.945550	-2.462388
H	5.724349	-0.697329	-4.569324
C	3.535254	-2.504499	0.516578
C	3.764325	-3.248418	1.670081
C	4.184303	-2.780804	-0.698001
C	4.668464	-4.308617	1.635145
H	3.238410	-2.982602	2.581064
C	5.084943	-3.861961	-0.702113
C	5.328812	-4.615477	0.443429
H	4.851248	-4.892313	2.532224
H	5.584035	-4.119203	-1.631428
H	6.027192	-5.445792	0.403357
C	-4.043332	1.693017	-0.854766
C	-4.962857	2.305753	-0.008058
C	-4.396136	1.230944	-2.132906
C	-6.282918	2.465016	-0.426591
H	-4.630301	2.659681	0.961911
C	-5.736058	1.401498	-2.524851
C	-6.669389	2.009623	-1.688997
H	-7.004100	2.940245	0.231573
H	-6.045186	1.030193	-3.497294
H	-7.697503	2.121059	-2.019723
C	-2.476488	-0.342731	-2.606831
C	-1.610532	-0.994093	-3.483099
C	-3.418373	0.600828	-3.051620
C	-1.657882	-0.695152	-4.844742
H	-0.928878	-1.739633	-3.088067
C	-3.434010	0.888688	-4.428027
C	-2.567252	0.254824	-5.315636
H	-0.991886	-1.207215	-5.533187
H	-4.136164	1.630097	-4.797477
H	-2.605567	0.498652	-6.373034
Ir	0.419665	0.770365	-0.121068
C	1.660034	1.818403	2.552548
C	0.486735	1.121779	2.432351
C	2.181827	1.665817	-0.621847
H	0.459775	0.065369	2.674321
H	-0.462803	1.641183	2.444763
C	2.140199	3.018675	-0.945179
O	3.440612	1.212480	-0.812894
C	0.834655	3.614476	-0.758701
C	3.471127	3.395432	-1.361305
C	4.212894	2.265246	-1.262492
O	-0.095946	2.839944	-0.311539
N	0.526962	4.895348	-1.015763
H	3.839075	4.356682	-1.684888
H	5.247239	2.031122	-1.459942
C	1.539186	5.867111	-1.429748
C	-0.832412	5.389295	-0.785238
H	1.054037	6.836995	-1.546526
H	2.325676	5.964265	-0.675003
H	1.984261	5.589304	-2.390150
H	-1.510186	4.543494	-0.689397
H	-0.870190	5.989528	0.131763
H	-1.137326	6.010555	-1.632568
H	0.237082	0.595795	-1.673609
C	1.703753	3.299501	2.587294
C	2.903583	3.990546	2.327050
C	0.571184	4.059651	2.943337
C	2.966233	5.381203	2.407167
H	3.796089	3.441110	2.049440
C	0.633981	5.448075	3.028344
H	-0.358529	3.560426	3.193224
C	1.832277	6.116019	2.759467
H	3.906020	5.888799	2.208427
H	-0.247231	6.008606	3.327602
H	1.885037	7.198075	2.841374
C	2.958047	1.078006	2.758796
H	3.456522	1.439867	3.665668
H	2.788120	0.004768	2.847790
H	3.648038	1.228652	1.921833
C	-0.682234	-2.428902	5.987176
H	-0.805665	-1.695286	6.788954
H	-1.185336	-3.352168	6.301873
H	0.386452	-2.664652	5.910078
C	-0.516468	-4.042770	3.553337
H	0.553983	-3.975626	3.788279
H	-0.971718	-4.656767	4.339531
H	-0.615243	-4.580252	2.609981
C	-4.257572	-3.383741	1.588198
H	-4.944699	-2.706897	1.062884
H	-4.803844	-4.314850	1.770684
H	-4.031698	-2.934820	2.556215
C	-4.378083	-5.193776	-0.713434
H	-4.744440	-5.856906	0.080760
H	-5.172466	-4.463066	-0.907690
H	-4.251844	-5.798526	-1.616037
C	-3.149881	1.196112	3.482769
H	-2.690671	1.930694	2.809330
H	-4.185240	1.069527	3.145235
H	-3.170580	1.625703	4.488458
C	0.544928	-4.678482	-1.516962
H	0.706812	-4.120184	-2.444488
H	1.450357	-4.575637	-0.915073
H	0.427873	-5.734154	-1.781814

III-L-6 Conformation 2

B3LYP/6-31G(d),SDD(Ir) Energy = -3686.874278
B3LYP/def2-TZVPP,SDD(Ir)/IEFPCM(dioxane) Energy = -3688.127703
B3LYP/def2-TZVPP,SDD(Ir)/IEFPCM(dioxane)//B3LYP/6-31G(d),SDD(Ir) Free Energy (Quasiharmonic) = -3687.259105
Number of Imaginary Frequencies = 0

Frequencies (Top 3 out of 369)

1. 15.2270 cm^{-1}
2. 17.0379 cm^{-1}
3. 25.4149 cm^{-1}

B3LYP/6-31G(d),SDD(Ir) Molecular Geometry in Cartesian Coordinates

O	-1.891561	1.787119	0.786660
C	-0.864611	2.642510	1.204180
C	-0.823441	3.919950	0.633300
C	-0.054641	2.278870	2.288120
C	0.108929	4.818570	1.153210
C	0.871429	3.218150	2.801390
C	0.954519	4.498820	2.217850
H	0.154278	5.818020	0.726680
C	-0.201100	0.955290	2.972820
C	0.771330	-0.033310	2.763980
C	-1.187060	0.751580	3.962410
C	0.895600	-1.156750	3.585000
C	-1.128090	-0.410530	4.760060
C	-0.075620	-1.311790	4.578470
H	-0.001060	-2.169190	5.244310
O	1.761380	0.201540	1.799880
P	1.802720	-0.225860	0.227510
P	-1.939670	0.437889	-0.120540
O	3.025890	-1.323869	0.376000
O	2.587510	1.044421	-0.429770
O	-2.839260	0.864639	-1.425400
O	-3.052660	-0.301451	0.828310
C	4.015090	-1.543029	-0.583630
C	4.080751	-2.809609	-1.161260
C	4.981430	-0.557139	-0.856860
C	5.104031	-3.112299	-2.057930
H	3.335891	-3.548399	-0.889830
C	5.992720	-0.887969	-1.777740
C	6.058200	-2.143929	-2.375180
H	5.152771	-4.100899	-2.504800
H	6.733320	-0.134628	-2.028540
H	6.852470	-2.365348	-3.081550
C	3.829209	1.513471	0.037740
C	3.855159	2.780111	0.613350
C	4.993600	0.764761	-0.187040
C	5.077019	3.329701	1.000270
H	2.921949	3.316621	0.743880
C	6.208600	1.341661	0.226930
C	6.255469	2.604301	0.811680
H	5.106729	4.319151	1.446900
H	7.125230	0.774772	0.096500
H	7.209569	3.019282	1.122040
C	-4.167490	1.317469	-1.396910
C	-4.421851	2.576909	-1.935490
C	-5.206140	0.473119	-0.971950
C	-5.731341	3.047269	-2.013700
H	-3.591591	3.167709	-2.301870
C	-6.516090	0.981988	-1.048460
C	-6.781881	2.250698	-1.554900
H	-5.926461	4.031359	-2.429280
H	-7.333890	0.363608	-0.691470
H	-7.804441	2.613248	-1.595330
C	-3.920820	-1.277081	0.333940
C	-3.767910	-2.579091	0.804030
C	-4.979600	-0.917331	-0.515470
C	-4.668649	-3.568061	0.413640
H	-2.946229	-2.787271	1.476150
C	-5.864520	-1.940131	-0.905460
C	-5.716849	-3.247901	-0.451800
H	-4.552869	-4.582901	0.783340
H	-6.673710	-1.695952	-1.586760
H	-6.417479	-4.012902	-0.772410
Ir	-0.230590	-1.033210	-0.895130
C	0.538330	0.486730	-3.072000
C	-0.502810	-0.451820	-3.068540
C	0.626000	-2.756910	-1.549740
C	1.937900	0.049370	-3.423440
C	0.276339	1.945110	-3.139920
H	-1.522640	-0.092760	-3.138010
H	-0.317390	-1.400700	-3.560890
C	0.543681	-3.882850	-0.739160
O	1.112661	-3.124730	-2.756210
H	2.074500	-1.024760	-3.305290
H	2.095810	0.306870	-4.481050
H	2.702670	0.578401	-2.854390
C	-0.855461	2.405550	-3.846710
C	1.226599	2.895200	-2.716110
C	-0.044319	-3.609120	0.549210
C	1.018981	-5.004370	-1.521260
C	1.352201	-4.487220	-2.727990
C	-1.015501	3.759160	-4.136800
H	-1.584951	1.696850	-4.222010
C	1.049909	4.250130	-2.984700
H	2.097959	2.571850	-2.161740
O	-0.421240	-2.390460	0.778860
N	-0.223409	-4.533580	1.510170
H	1.100831	-6.045070	-1.249130
H	1.752801	-4.909210	-3.636470
C	-0.063511	4.686510	-3.707040
H	-1.875641	4.086179	-4.714050
H	1.792349	4.965280	-2.642880
C	0.199931	-5.923380	1.332440
C	-0.918039	-4.219160	2.758610
H	-0.185042	5.740630	-3.939670
H	0.101981	-6.434200	2.291390
H	-0.423129	-6.445690	0.597830
H	1.247351	-5.975960	1.025460
H	-1.100719	-3.149770	2.818900
H	-1.867769	-4.765441	2.809690
H	-0.296139	-4.520190	3.607710
H	-1.542570	-1.752580	-1.460780
C	-2.156700	-0.665741	5.837180
H	-1.970890	-1.618941	6.341040
H	-2.148730	0.120089	6.603160
H	-3.173850	-0.694251	5.427200
C	-2.281861	1.764359	4.205020
H	-3.257750	1.357979	3.909830
H	-2.353321	2.028659	5.266510
H	-2.124361	2.684079	3.640710
C	1.766809	2.884460	3.974250
H	1.816529	3.717990	4.682510
H	1.425179	2.004210	4.519650
H	2.793759	2.686101	3.638670
C	1.912909	5.541590	2.748020
H	1.902448	6.437690	2.120560
H	1.650928	5.851050	3.768060
H	2.944029	5.170201	2.789720
C	-1.781341	4.335090	-0.448790
H	-1.636661	3.749170	-1.360520
H	-2.819551	4.194659	-0.129950
H	-1.639461	5.389390	-0.702520
C	2.040570	-2.127910	3.443450
H	1.954320	-2.740490	2.539130
H	2.999080	-1.604079	3.372270
H	2.079371	-2.799560	4.306580

III-L-7 Conformation 1

B3LYP/6-31G(d),SDD(Ir) Energy = -3608.277656
B3LYP/def2-TZVPP,SDD(Ir)/IEFPCM(dioxane) Energy = -3609.508774
B3LYP/def2-TZVPP,SDD(Ir)/IEFPCM(dioxane)//B3LYP/6-31G(d),SDD(Ir) Free Energy (Quasiharmonic) = -3608.695659
Number of Imaginary Frequencies = 0

Frequencies (Top 3 out of 351)

1. 5.7754 cm^{-1}
2. 14.7926 cm^{-1}
3. 15.9197 cm^{-1}

B3LYP/6-31G(d),SDD(Ir) Molecular Geometry in Cartesian Coordinates

O	-0.284394	2.877449	0.329717
C	-1.093248	2.827036	1.470709
C	-0.531318	2.903305	2.737959
C	-2.480488	2.811223	1.271512
C	-1.381181	2.931742	3.843095
H	0.545208	2.957562	2.857097
C	-3.328788	2.838413	2.396655
C	-2.770527	2.898084	3.695216
H	-0.953317	2.994165	4.840217
C	-2.989486	2.761715	-0.135064
C	-3.033634	1.523632	-0.791571
C	-3.387166	3.918606	-0.834851
C	-3.487040	1.396293	-2.098168
C	-3.835544	3.808968	-2.170722
C	-3.877487	2.550673	-2.775237
H	-3.546414	0.423825	-2.568622
H	-4.233248	2.464449	-3.798553
O	-2.696080	0.398380	-0.016857
P	-1.680819	-0.830607	-0.289159
P	0.763734	1.748420	-0.187364
O	-2.216929	-1.332329	-1.751529
O	-2.284034	-1.935437	0.728989
O	2.200721	2.147995	0.483335
O	0.872093	2.387442	-1.686699
C	-2.225503	-2.671033	-2.159105
C	-1.429434	-3.013855	-3.247975
C	-3.093153	-3.588613	-1.543886
C	-1.467225	-4.316271	-3.743047
H	-0.797992	-2.254271	-3.697071
C	-3.101198	-4.896712	-2.060162
C	-2.301816	-5.261123	-3.141575
H	-0.849462	-4.588884	-4.593411
H	-3.743160	-5.636991	-1.592229
H	-2.334531	-6.279749	-3.516431
C	-3.613758	-2.387285	0.632046
C	-4.473328	-2.058648	1.675495
C	-4.003924	-3.211868	-0.434990
C	-5.773622	-2.560215	1.676346
H	-4.110682	-1.421795	2.475569
C	-5.325228	-3.695374	-0.408423
C	-6.199019	-3.380207	0.628960
H	-6.449436	-2.308340	2.487967
H	-5.669394	-4.313460	-1.231996
H	-7.213242	-3.767411	0.614945
C	2.869204	3.356613	0.231974
C	3.077297	4.207385	1.314043
C	3.397120	3.628297	-1.041541
C	3.819117	5.374872	1.142626
H	2.667723	3.941223	2.282830
C	4.136303	4.816361	-1.184405
C	4.348068	5.680162	-0.112814
H	3.978775	6.041026	1.985026
H	4.531324	5.067464	-2.164127
H	4.918097	6.592394	-0.259752
C	1.981825	2.115039	-2.490682
C	1.790878	1.325102	-3.621626
C	3.216429	2.719017	-2.199337
C	2.857751	1.102368	-4.491907
H	0.804744	0.916098	-3.815964
C	4.274898	2.469654	-3.090896
C	4.103951	1.671753	-4.220575
H	2.712774	0.492826	-5.378845
H	5.246043	2.909311	-2.883416
H	4.940610	1.501076	-4.891456
Ir	0.543687	-0.620306	-0.081437
C	0.974193	-1.637799	2.806264
C	0.664300	-0.341994	2.482597
C	0.938979	-2.606972	-0.283854
H	-0.353328	0.012127	2.604935
H	1.436388	0.417340	2.440664
C	2.268744	-2.983133	-0.444853
O	0.162587	-3.711255	-0.350290
C	3.196903	-1.875537	-0.346712
C	2.282223	-4.416865	-0.623428
C	0.983932	-4.801414	-0.559476
O	2.689998	-0.715378	-0.095534
N	4.527649	-1.971404	-0.494776
H	3.121102	-5.079107	-0.770725
H	0.481441	-5.752568	-0.639640
C	5.194320	-3.258082	-0.694019
C	5.378152	-0.790095	-0.335208
H	6.259533	-3.072222	-0.837678
H	5.071297	-3.909140	0.177804
H	4.813299	-3.763194	-1.585980
H	4.762648	0.105545	-0.378669
H	5.900163	-0.824102	0.628458
H	6.116282	-0.767328	-1.142433
H	0.578883	-0.582361	-1.652340
C	2.369978	-2.094451	3.010509
C	2.685022	-3.467519	3.003024
C	3.410218	-1.183080	3.284248
C	3.983859	-3.910996	3.249648
H	1.913073	-4.199813	2.795316
C	4.706303	-1.625177	3.535931
H	3.200842	-0.120386	3.334211
C	4.999845	-2.992048	3.519374
H	4.197450	-4.976197	3.245342
H	5.484340	-0.903134	3.767431
H	6.007899	-3.337217	3.731712
C	-0.131102	-2.626962	3.084996
H	-0.005903	-3.052905	4.087536
H	-1.109839	-2.151635	3.018048
H	-0.124101	-3.458023	2.372187
C	-3.658323	2.939828	4.916897
H	-4.300927	2.052824	4.985106
H	-3.063437	2.991001	5.833313
H	-4.325891	3.810508	4.903813
C	-4.831259	2.808235	2.235997
H	-5.292845	3.715083	2.647179
H	-5.127961	2.725284	1.189440
H	-5.269391	1.961123	2.778239
C	-3.331133	5.281683	-0.184279
H	-2.679358	5.958734	-0.750235
H	-4.322983	5.750449	-0.157211
H	-2.953980	5.234715	0.838372
C	-4.266664	5.032137	-2.944862
H	-5.098456	5.553110	-2.454152
H	-3.451040	5.760101	-3.040313
H	-4.591812	4.762905	-3.953852

III-L-7 Conformation 2

B3LYP/6-31G(d),SDD(Ir) Energy = -3608.249572
B3LYP/def2-TZVPP,SDD(Ir)/IEFPCM(dioxane) Energy = -3609.475624
B3LYP/def2-TZVPP,SDD(Ir)/IEFPCM(dioxane)//B3LYP/6-31G(d),SDD(Ir) Free Energy (Quasiharmonic) = -3608.659378
Number of Imaginary Frequencies = 0

Frequencies (Top 3 out of 351)

1. 15.0771 cm^{-1}
2. 19.9362 cm^{-1}
3. 21.4745 cm^{-1}

B3LYP/6-31G(d),SDD(Ir) Molecular Geometry in Cartesian Coordinates

O	-1.887664	1.533639	0.913937
C	-1.027142	2.634119	0.876473
C	-1.148038	3.584563	-0.127714
C	-0.141353	2.800522	1.950723
C	-0.375565	4.741841	-0.049674
H	-1.844395	3.433709	-0.942247
C	0.628587	3.979430	2.022731
C	0.499524	4.964858	1.016279

H	-0.470218	5.493329	-0.828730
C	0.026165	1.687801	2.937842
C	0.947027	0.678426	2.620885
C	-0.660181	1.624726	4.165543
C	1.245747	-0.352086	3.501199
C	-0.383047	0.570168	5.065807
C	0.571844	-0.391393	4.721279
H	2.001613	-1.086297	3.244028
H	0.803807	-1.183077	5.429440
O	1.653233	0.828676	1.425839
P	1.755936	-0.116782	0.109177
P	-1.981350	0.244880	-0.073044
O	2.952855	-1.131588	0.626042
O	2.574913	0.902057	-0.860462
O	-2.929268	0.693242	-1.334037
O	-3.047125	-0.522637	0.903547
C	4.036195	-1.543679	-0.157229
C	4.169366	-2.911180	-0.389807
C	5.011777	-0.624615	-0.589589
C	5.278917	-3.396099	-1.079880
H	3.402828	-3.580616	-0.018522
C	6.113648	-1.144508	-1.293562
C	6.252851	-2.507889	-1.538657
H	5.377790	-4.462824	-1.257770
H	6.865793	-0.453348	-1.661679
H	7.116188	-2.872962	-2.086528
C	3.744184	1.554945	-0.427762
C	3.676038	2.928990	-0.220127
C	4.937352	0.829507	-0.311226
C	4.836966	3.621861	0.121958
H	2.721326	3.432437	-0.329086
C	6.088187	1.555505	0.046402
C	6.043504	2.931014	0.259517
H	4.798200	4.695355	0.282333
H	7.024826	1.021009	0.171840
H	6.948733	3.462181	0.537534
C	-4.248784	1.159360	-1.198209
C	-4.516585	2.459002	-1.620268
C	-5.267154	0.295322	-0.763644
C	-5.824158	2.941266	-1.588521
H	-3.703533	3.072650	-1.991088
C	-6.574635	0.812687	-0.735037
C	-6.855262	2.115982	-1.136970
H	-6.032655	3.956062	-1.913657
H	-7.376837	0.175937	-0.374739
H	-7.875311	2.485497	-1.096722
C	-3.917220	-1.494441	0.406994
C	-3.721154	-2.814289	0.807154
C	-5.014071	-1.114369	-0.383896
C	-4.624654	-3.798245	0.408770
H	-2.866193	-3.042622	1.432029
C	-5.903115	-2.130160	-0.779550
C	-5.716144	-3.454682	-0.391983
H	-4.475987	-4.827691	0.721370
H	-6.745990	-1.867924	-1.411821
H	-6.419518	-4.215871	-0.715526
Ir	-0.249794	-1.213868	-0.766755
C	0.344383	-0.035032	-3.167386
C	-0.683850	-0.969466	-2.972526
C	0.683581	-2.986497	-1.129425
C	1.707170	-0.537633	-3.580250
C	0.079627	1.413717	-3.344526
H	-1.714862	-0.640632	-3.021773
H	-0.513635	-1.974557	-3.345760
C	0.685312	-3.933332	-0.108668
O	1.162169	-3.560967	-2.254430
H	1.810338	-1.607626	-3.402795
H	1.809779	-0.350122	-4.658579
H	2.525640	-0.014650	-3.085629
C	-1.189401	1.861987	-3.769438
C	1.120354	2.362034	-3.268573
C	0.129639	-3.439754	1.127155
C	1.200677	-5.162804	-0.672241
C	1.476495	-4.878235	-1.967450
C	-1.405527	3.197753	-4.099417
H	-2.005382	1.159487	-3.882955
C	0.899052	3.699448	-3.585963
H	2.106988	2.055587	-2.948675
O	-0.317078	-2.222870	1.126006
N	0.037014	-4.147469	2.266620
H	1.342981	-6.124466	-0.203945
H	1.877592	-5.451087	-2.788927
C	-0.363290	4.123540	-4.006841
H	-2.383698	3.509958	-4.454290
H	1.718770	4.408725	-3.518386
C	0.547295	-5.514214	2.373443
C	-0.655461	-3.611378	3.440803
H	-0.529786	5.162607	-4.277101
H	0.556335	-5.793393	3.428273
H	-0.084853	-6.227843	1.832213
H	1.571038	-5.577194	1.997662
H	-0.939578	-2.577992	3.259340
H	-1.548442	-4.211195	3.654491
H	0.009678	-3.652521	4.309320
H	-1.536926	-2.087180	-1.147598
C	-1.094492	0.484559	6.395754
H	-0.753913	-0.382685	6.969140
H	-0.919758	1.377375	7.009229
H	-2.181237	0.397452	6.270435
C	-1.682719	2.673389	4.536070
H	-2.666739	2.219049	4.705653
H	-1.408435	3.188140	5.465573
H	-1.794236	3.426732	3.754441
C	1.606862	4.203605	3.153191
H	2.638065	4.236072	2.777999
H	1.421080	5.160842	3.654822
H	1.554470	3.414156	3.903974
C	1.293721	6.248920	1.083298
H	1.102759	6.874963	0.206764
H	1.037261	6.837853	1.973131
H	2.373950	6.062275	1.134229

III-L-8 Conformation 1

B3LYP/6-31G(d),SDD(Ir) Energy = -3451.012917
B3LYP/def2-TZVPP,SDD(Ir)/IEFPCM(dioxane) Energy = -3452.186438
B3LYP/def2-TZVPP,SDD(Ir)/IEFPCM(dioxane)//B3LYP/6-31G(d),SDD(Ir) Free Energy (Quasiharmonic) = -3451.474351Number of Imaginary Frequencies = 0

Frequencies (Top 3 out of 315)

1. 9.8821 cm^{-1}
2. 13.1135 cm^{-1}
3. 17.4750 cm^{-1}

B3LYP/6-31G(d),SDD(Ir) Molecular Geometry in Cartesian Coordinates

O	-0.611496	2.944089	-0.442540
C	-0.251392	3.208050	-1.764315
C	-1.174527	3.058626	-2.797947
C	1.024281	3.748206	-1.990159
C	-0.826351	3.436132	-4.095878
H	-2.161611	2.667433	-2.582021
C	1.340892	4.135909	-3.300727
C	0.432506	3.980520	-4.348253
H	-1.546561	3.318498	-4.900200
H	2.323882	4.556589	-3.491493
H	0.706544	4.286910	-5.353111
C	2.014459	3.945462	-0.896221
C	2.577403	2.863027	-0.202747
C	2.469712	5.231362	-0.574334
C	3.564317	3.038216	0.763306
C	3.440097	5.430895	0.407105
H	2.042185	6.079997	-1.100261
C	3.984805	4.333469	1.072415

H	4.003261	2.182213	1.257230
H	3.770372	6.437035	0.646165
H	4.749800	4.475360	1.829995
O	2.147701	1.595968	-0.623334
P	1.878030	0.149328	0.035729
P	-1.213268	1.569099	0.192655
O	2.568927	0.304753	1.507188
O	2.912202	-0.820009	-0.753316
O	-2.797659	1.502816	-0.194331
O	-1.223472	2.188067	1.704449
C	3.094037	-0.803493	2.191297
C	2.458798	-1.202280	3.363145
C	4.280051	-1.404661	1.738412
C	3.002841	-2.240423	4.118572
H	1.555462	-0.689387	3.674660
C	4.801998	-2.450360	2.519371
C	4.176264	-2.866236	3.692666
H	2.512976	-2.554770	5.035159
H	5.707148	-2.948290	2.184957
H	4.604403	-3.677917	4.272987
C	4.303963	-0.634261	-0.671934
C	4.966512	-0.223051	-1.824258
C	4.983648	-0.947074	0.516349
C	6.356539	-0.116298	-1.812527
H	4.389771	-0.002943	-2.716504
C	6.384762	-0.825701	0.497533
C	7.065309	-0.418738	-0.647709
H	6.880328	0.203812	-2.708005
H	6.938603	-1.038940	1.406851
H	8.147219	-0.330414	-0.628252
C	-3.775777	2.404895	0.260705
C	-4.483369	3.112465	-0.707380
C	-4.097329	2.487671	1.625935
C	-5.530641	3.949338	-0.326584
H	-4.219000	2.992614	-1.752290
C	-5.154788	3.346028	1.979024
C	-5.864254	4.068373	1.023488
H	-6.078566	4.504613	-1.081704
H	-5.403856	3.456088	3.029968
H	-6.670213	4.726506	1.333102
C	-1.993366	1.585712	2.702393
C	-1.325399	0.957436	3.750626
C	-3.391433	1.717212	2.678498
C	-2.057917	0.408418	4.802493
H	-0.240710	0.935427	3.739291
C	-4.103678	1.142352	3.746451
C	-3.452018	0.492545	4.792546
H	-1.542007	-0.076752	5.625660
H	-5.187545	1.208349	3.745774
H	-4.031483	0.061555	5.603388
Ir	-0.232797	-0.584168	-0.023811
C	-0.324914	-1.653326	-2.841294
C	-0.607700	-0.345660	-2.535444
C	0.175791	-2.561760	0.223934
H	0.138997	0.420295	-2.713471
H	-1.633881	-0.005300	-2.456919
C	-0.900630	-3.411979	0.455684
O	1.322914	-3.268903	0.327193
C	-2.186724	-2.751498	0.367846
C	-0.352375	-4.722995	0.716344
C	0.992233	-4.576620	0.625898
O	-2.175261	-1.489873	0.091871
N	-3.372386	-3.352950	0.552169
H	-0.864669	-5.646507	0.937158
H	1.825147	-5.253503	0.735579
C	-3.484620	-4.795430	0.770167
C	-4.617202	-2.589987	0.442791
H	-4.543687	-5.051919	0.818787
H	-3.033449	-5.354323	-0.054947
H	-3.016469	-5.091942	1.714217
H	-4.390737	-1.526255	0.463243
H	-5.130467	-2.831825	-0.495501
H	-5.270403	-2.843196	1.283395
H	-0.188535	-0.565018	1.548924
C	-1.382568	-2.682080	-2.988176
C	-1.063286	-4.053098	-2.942083
C	-2.721322	-2.326423	-3.249344
C	-2.040840	-5.027724	-3.139285
H	-0.044527	-4.366043	-2.742422
C	-3.697286	-3.298842	-3.451130
H	-2.997998	-1.281119	-3.331519
C	-3.361595	-4.654939	-3.396408
H	-1.766155	-6.078241	-3.105389
H	-4.717016	-2.998128	-3.674751
H	-4.120195	-5.413031	-3.570221
C	1.090213	-2.058253	-3.177389
H	1.117702	-2.524889	-4.169018
H	1.757011	-1.195019	-3.173812
H	1.489951	-2.782436	-2.460397

III-L-8 Conformation 2

B3LYP/6-31G(d),SDD(Ir) Energy = -3451.012915
B3LYP/def2-TZVPP,SDD(Ir)/IEFPCM(dioxane) Energy = -3452.185583
B3LYP/def2-TZVPP,SDD(Ir)/IEFPCM(dioxane)//B3LYP/6-31G(d),SDD(Ir) Free Energy (Quasiharmonic) = -3451.472301
Number of Imaginary Frequencies = 0

Frequencies (Top 3 out of 315)

1. 7.1104 cm^{-1}
2. 12.2198 cm^{-1}
3. 20.3041 cm^{-1}

B3LYP/6-31G(d),SDD(Ir) Molecular Geometry in Cartesian Coordinates

O	1.495872	1.702122	0.625443
C	2.165611	2.876039	0.250010
C	2.701348	3.634706	1.287852
C	2.196318	3.307263	-1.087382
C	3.296817	4.864304	1.013674
H	2.650683	3.246950	2.299584
C	2.806636	4.553096	-1.327172
C	3.346691	5.325545	-0.301940
H	3.714433	5.454393	1.823794
H	2.832506	4.923190	-2.347540
H	3.799232	6.285443	-0.531175
C	1.702688	2.526163	-2.255830
C	0.417595	1.966352	-2.356251
C	2.551728	2.372972	-3.367747
C	-0.008430	1.303579	-3.510050
C	2.148119	1.699275	-4.517739
H	3.555932	2.781258	-3.308564
C	0.861012	1.163500	-4.590315
H	-1.024650	0.928096	-3.560754
H	2.833262	1.599817	-5.354241
H	0.525543	0.650914	-5.487051
O	-0.470892	2.160918	-1.301423
P	-1.295869	0.979117	-0.527712
P	1.796003	0.133610	0.387505
O	-2.565955	0.823999	-1.551987
O	-1.922308	1.850705	0.690509
O	2.511785	-0.435974	1.733421
O	3.027809	0.227697	-0.665385
C	-3.917843	0.780667	-1.202761
C	-4.630146	-0.332188	-1.644522
C	-4.549943	1.859323	-0.557718
C	-6.005199	-0.405924	-1.435981
H	-4.087223	-1.114709	-2.161646
C	-5.937980	1.746512	-0.349370
C	-6.660772	0.637496	-0.779147
H	-6.560888	-1.270282	-1.787728
H	-6.450175	2.551596	0.168715
H	-7.731279	0.589823	-0.603858
C	-2.568400	3.079979	0.446614
C	-1.922913	4.236080	0.872442

C	-3.842056	3.093715	-0.138854
C	-2.557941	5.466826	0.717600
H	-0.935220	4.157550	1.314327
C	-4.451313	4.353578	-0.290911
C	-3.824588	5.523053	0.131207
H	-2.061766	6.375324	1.045031
H	-5.424887	4.408216	-0.768011
H	-4.320962	6.478831	-0.006337
C	3.784318	-0.024705	2.170165
C	3.845172	0.624289	3.400637
C	4.935328	-0.357452	1.437803
C	5.084665	0.977211	3.931689
H	2.922944	0.827182	3.935869
C	6.169887	0.021133	1.997102
C	6.249725	0.677239	3.222746
H	5.137404	1.481060	4.891993
H	7.078262	-0.194430	1.443041
H	7.220008	0.956314	3.621874
C	3.954875	-0.806545	-0.853135
C	3.959800	-1.445459	-2.088911
C	4.893827	-1.091602	0.149834
C	4.916941	-2.426111	-2.344557
H	3.226122	-1.155629	-2.833884
C	5.840359	-2.091897	-0.135567
C	5.854298	-2.753275	-1.361629
H	4.930014	-2.929568	-3.306547
H	6.565901	-2.356849	0.627529
H	6.598951	-3.520735	-1.550343
Ir	-0.063210	-1.005222	-0.105271
C	-1.332217	-0.921917	2.704721
C	-0.418594	-1.849601	2.268794
C	0.797656	-2.848174	-0.210690
H	0.623637	-1.755013	2.552751
H	-0.737677	-2.835687	1.955442
C	0.003471	-3.911643	-0.623790
O	2.033050	-3.306235	0.074820
C	-1.374824	-3.541502	-0.869916
C	0.829325	-5.097946	-0.564039
C	2.044596	-4.670407	-0.143425
O	-1.697547	-2.307721	-0.667689
N	-2.334305	-4.400252	-1.257917
H	0.574024	-6.122149	-0.788518
H	2.985089	-5.163297	0.046336
C	-2.045194	-5.772251	-1.673098
C	-3.735347	-3.982000	-1.304324
H	-2.850288	-6.108304	-2.330175
H	-1.112152	-5.815393	-2.236442
H	-1.988266	-6.454689	-0.816161
H	-3.870234	-3.083362	-0.705808
H	-4.047632	-3.783126	-2.337219
H	-4.358101	-4.785705	-0.899160
H	0.348726	-0.895783	-1.623056
C	-2.793975	-1.173599	2.677478
C	-3.713358	-0.113412	2.790563
C	-3.306400	-2.485742	2.613247
C	-5.086202	-0.351873	2.813997
H	-3.357426	0.908395	2.845690
C	-4.677495	-2.723896	2.644156
H	-2.627994	-3.331529	2.582208
C	-5.574650	-1.656797	2.739795
H	-5.774231	0.484320	2.892524
H	-5.046683	-3.745340	2.615258
H	-6.644342	-1.843162	2.771040
C	-0.844445	0.333891	3.388015
H	-1.210591	0.350261	4.422154
H	0.247163	0.367903	3.404330
H	-1.207848	1.239564	2.897227

III-L-8 Conformation 3

B3LYP/6-31G(d),SDD(Ir) Energy = -3451.013357
B3LYP/def2-TZVPP,SDD(Ir)/IEFPCM(dioxane) Energy = -3452.185893
B3LYP/def2-TZVPP,SDD(Ir)/IEFPCM(dioxane)//B3LYP/6-31G(d),SDD(Ir) Free Energy (Quasiharmonic) = -3451.472262
Number of Imaginary Frequencies = 0

Frequencies (Top 3 out of 315)

1. 9.4695 cm^{-1}
2. 14.6353 cm^{-1}
3. 21.9093 cm^{-1}

B3LYP/6-31G(d),SDD(Ir) Molecular Geometry in Cartesian Coordinates

O	1.015508	-1.754891	0.154578
C	1.459531	-2.844076	0.916869
C	1.939048	-3.938778	0.201823
C	1.322861	-2.862924	2.315597
C	2.310558	-5.099492	0.877358
H	2.017333	-3.863787	-0.877327
C	1.709298	-4.050910	2.964595
C	2.193226	-5.154491	2.266460
H	2.685096	-5.952331	0.319349
H	1.601737	-4.104218	4.043684
H	2.470619	-6.054679	2.806362
C	0.870480	-1.715461	3.150705
C	-0.314283	-0.990960	2.936088
C	1.645342	-1.349376	4.266910
C	-0.717477	0.033747	3.795515
C	1.266233	-0.318502	5.123108
H	2.574491	-1.881817	4.445229
C	0.078287	0.375895	4.887565
H	-1.663698	0.533216	3.617371
H	1.892703	-0.064005	5.972704
H	-0.239550	1.170317	5.556353
O	-1.141894	-1.373768	1.881753
P	-1.694686	-0.375276	0.712744
P	1.540605	-0.242583	-0.034663
O	-3.024794	0.204550	1.485112
O	-2.328555	-1.463640	-0.308573
O	2.456474	-0.192161	-1.374828
O	2.641882	-0.160216	1.157539
C	-4.277273	0.394209	0.897072
C	-4.794468	1.687421	0.933999
C	-5.027971	-0.691526	0.408481
C	-6.077502	1.937023	0.451151
H	-4.180861	2.472664	1.360239
C	-6.312879	-0.402631	-0.089412
C	-6.835390	0.887598	-0.073134
H	-6.483833	2.943652	0.487681
H	-6.903856	-1.215688	-0.500009
H	-7.831715	1.071358	-0.463391
C	-3.230157	-2.452096	0.130230
C	-2.782870	-3.769102	0.144829
C	-4.547318	-2.094311	0.449476
C	-3.673348	-4.784371	0.490460
H	-1.750628	-3.978514	-0.114389
C	-5.419478	-3.141284	0.800160
C	-4.993520	-4.467099	0.819484
H	-3.334685	-5.815809	0.509166
H	-6.439896	-2.899479	1.081099
H	-5.689642	-5.251292	1.101045
C	3.694234	-0.853724	-1.488274
C	3.780169	-1.866446	-2.439579
C	4.804246	-0.426557	-0.741483
C	5.004851	-2.493013	-2.665721
H	2.890600	-2.137357	-2.998478
C	6.023316	-1.083834	-0.989727
C	6.128647	-2.099805	-1.936159
H	5.078646	-3.282836	-3.407290
H	6.895002	-0.797013	-0.409555
H	7.084976	-2.587464	-2.098560
C	3.673826	0.787899	1.166114
C	3.640581	1.766343	2.154611
C	4.736652	0.666143	0.258216

C	4.688677	2.681519	2.241881
H	2.805680	1.786808	2.847312
C	5.774625	1.608378	0.367286
C	5.753860	2.604586	1.341121
H	4.672912	3.448175	3.010697
H	6.601441	1.558088	-0.334802
H	6.570973	3.317417	1.399135
Ir	-0.146618	1.218143	-0.117799
C	-1.246937	0.318500	-2.976591
C	-0.330429	1.295811	-2.681127
C	0.989751	2.860117	-0.512936
C	-2.720335	0.639031	-2.912818
C	-0.849560	-1.005396	-3.513516
H	0.727611	1.153174	-2.869544
H	-0.672640	2.320705	-2.595762
C	0.360026	4.099655	-0.532862
O	2.296734	3.026974	-0.800267
H	-2.888727	1.631170	-2.488017
H	-3.143756	0.608786	-3.925039
H	-3.275965	-0.086356	-2.313324
C	0.348117	-1.149955	-4.243320
C	-1.691397	-2.128380	-3.398907
C	-1.069843	4.027697	-0.316965
C	1.371034	5.077133	-0.872853
C	2.519707	4.372574	-1.015588
C	0.693793	-2.369790	-4.820928
H	0.992387	-0.291232	-4.398388
C	-1.337034	-3.352280	-3.963031
H	-2.622260	-2.053912	-2.850757
O	-1.583276	2.852005	-0.160743
N	-1.886659	5.096396	-0.301717
H	1.276175	6.143818	-1.004599
H	3.536244	4.643010	-1.254397
C	-0.144053	-3.478909	-4.677098
H	1.606907	-2.447781	-5.404819
H	-2.000280	-4.205369	-3.852823
C	-1.387824	6.470918	-0.336115
C	-3.339096	4.934324	-0.255649
H	0.122300	-4.428138	-5.133333
H	-2.164129	7.125890	0.064850
H	-0.501871	6.575147	0.291973
H	-1.154252	6.794349	-1.357674
H	-3.597127	3.894628	-0.441937
H	-3.728995	5.235272	0.724589
H	-3.796024	5.567299	-1.023705
H	0.148106	1.543190	1.393584

III-L-8 Conformation 4

B3LYP/6-31G(d),SDD(Ir) Energy = -3451.011244
B3LYP/def2-TZVPP,SDD(Ir)/IEFPCM(dioxane) Energy = -3452.183518
B3LYP/def2-TZVPP,SDD(Ir)/IEFPCM(dioxane)//B3LYP/6-31G(d),SDD(Ir) Free Energy (Quasiharmonic) = -3451.470957
Number of Imaginary Frequencies = 0

Frequencies (Top 3 out of 315)

1. 8.1063 cm^{-1}
2. 12.2369 cm^{-1}
3. 18.0652 cm^{-1}

B3LYP/6-31G(d),SDD(Ir) Molecular Geometry in Cartesian Coordinates

O	-0.662723	-1.957268	-0.329521
C	-0.943980	-2.999976	-1.219747
C	-1.247635	-4.232694	-0.646127
C	-0.815771	-2.830882	-2.609465
C	-1.432911	-5.350804	-1.456601
H	-1.341562	-4.296300	0.432745
C	-1.007559	-3.983175	-3.396475
C	-1.306701	-5.224155	-2.839919
H	-1.669812	-6.310488	-1.007289
H	-0.896214	-3.893310	-4.472638
H	-1.436242	-6.087227	-3.485715
C	-0.570455	-1.539533	-3.315249
C	0.480070	-0.646856	-3.039640
C	-1.406947	-1.210172	-4.400094
C	0.709728	0.487781	-3.821921
C	-1.206575	-0.069610	-5.173181
H	-2.239786	-1.869531	-4.623825
C	-0.137253	0.781312	-4.888272
H	1.563704	1.119301	-3.598813
H	-1.876737	0.146876	-5.999638
H	0.046079	1.663231	-5.494703
O	1.360404	-0.945150	-2.001568
P	1.712781	0.046591	-0.754817
P	-1.413627	-0.579089	0.049138
O	3.047076	0.772494	-1.350480
O	2.317811	-1.009945	0.310083
O	-2.171358	-0.800307	1.475729
O	-2.654320	-0.638859	-1.013609
C	4.279848	0.927450	-0.699749
C	4.778701	2.227056	-0.632832
C	5.028367	-0.177064	-0.249643
C	6.038113	2.462438	-0.086327
H	4.166152	3.030949	-1.018496
C	6.290177	0.098970	0.312777
C	6.794524	1.393128	0.396348
H	6.423341	3.476570	-0.037671
H	6.875865	-0.730395	0.697176
H	7.772254	1.564539	0.836152
C	3.259199	-1.976817	-0.095670
C	2.841883	-3.302415	-0.144449
C	4.576854	-1.584760	-0.362892
C	3.765214	-4.291017	-0.479614
H	1.807921	-3.539586	0.078718
C	5.482488	-2.605179	-0.708851
C	5.086505	-3.938751	-0.766817
H	3.450773	-5.329289	-0.524659
H	6.505361	-2.335878	-0.953082
H	5.807434	-4.702000	-1.043597
C	-3.225184	-1.712470	1.658962
C	-2.992688	-2.784832	2.516193
C	-4.478224	-1.477836	1.070248
C	-4.031492	-3.669651	2.803490
H	-2.008229	-2.898519	2.958342
C	-5.503198	-2.391370	1.376703
C	-5.289159	-3.471382	2.229627
H	-3.857848	-4.507668	3.471932
H	-6.476980	-2.254302	0.916500
H	-6.101570	-4.159970	2.440730
C	-3.838878	0.078185	-0.819216
C	-4.120809	1.111877	-1.708541
C	-4.744373	-0.324483	0.176586
C	-5.335891	1.788402	-1.608504
H	-3.392989	1.356905	-2.475071
C	-5.955627	0.385796	0.260222
C	-6.252071	1.426969	-0.617118
H	-5.565855	2.589897	-2.304295
H	-6.667539	0.113710	1.033689
H	-7.198826	1.951554	-0.529482
Ir	-0.001164	1.291178	0.004237
C	1.350126	0.785320	2.922589
C	0.213014	1.467071	2.573648
C	0.756161	3.180330	-0.186031
C	2.689411	1.473544	2.821546
C	1.316812	-0.571671	3.515305
H	-0.773407	1.056647	2.756841
H	0.277354	2.540160	2.433533
C	-0.107482	4.237812	0.099545
O	1.910290	3.693346	-0.669541
H	2.586612	2.481673	2.413524
H	3.151308	1.538311	3.814484
H	3.386364	0.922002	2.183090
C	0.176234	-1.028370	4.206516
C	2.447661	-1.410637	3.482627

C	-1.422325	3.824415	0.545456
C	0.583054	5.459343	-0.242098
C	1.797159	5.069255	-0.698490
C	0.165046	-2.275545	4.826871
H	-0.694042	-0.387358	4.294962
C	2.430893	-2.664097	4.090270
H	3.344458	-1.091780	2.964508
O	-1.651749	2.555609	0.577493
N	-2.404689	4.658941	0.923773
H	0.239403	6.480345	-0.180514
H	2.660754	5.602743	-1.064612
C	1.290465	-3.101850	4.766359
H	-0.715843	-2.592884	5.378259
H	3.313027	-3.295836	4.042191
C	-2.206653	6.100424	1.071042
C	-3.736378	4.142647	1.249504
H	1.283699	-4.071578	5.255979
H	-2.974663	6.483960	1.745841
H	-1.232405	6.313900	1.513769
H	-2.295678	6.623058	0.111082
H	-3.833270	3.121977	0.887128
H	-3.899252	4.160319	2.333906
H	-4.490510	4.773269	0.768486
H	-0.419799	1.468030	-1.499037

III-L-8 Conformation 5

B3LYP/6-31G(d),SDD(Ir) Energy = -3451.006805
B3LYP/def2-TZVPP,SDD(Ir)/IEFPCM(dioxane) Energy = -3452.181498
B3LYP/def2-TZVPP,SDD(Ir)/IEFPCM(dioxane)//B3LYP/6-31G(d),SDD(Ir) Free Energy (Quasiharmonic) = -3451.468406
Number of Imaginary Frequencies = 0

Frequencies (Top 3 out of 315)

1. 14.9165 cm^{-1}
2. 17.1499 cm^{-1}
3. 20.9944 cm^{-1}

B3LYP/6-31G(d),SDD(Ir) Molecular Geometry in Cartesian Coordinates

O	-2.477609	1.539417	-0.640272
C	-2.340091	1.949610	-1.963895
C	-2.867752	1.193823	-3.008140
C	-1.774577	3.215764	-2.184069
C	-2.826041	1.693448	-4.311534
H	-3.332764	0.238281	-2.797031
C	-1.761599	3.700864	-3.499290
C	-2.276766	2.951137	-4.557730
H	-3.241434	1.106007	-5.125268
H	-1.327203	4.678636	-3.686319
H	-2.251243	3.350337	-5.567044
C	-1.221123	4.035131	-1.072020
C	-0.118411	3.596351	-0.323196
C	-1.746542	5.298809	-0.772862
C	0.452737	4.370353	0.681481
C	-1.201148	6.085297	0.242160
H	-2.601400	5.654602	-1.340523
C	-0.105325	5.618546	0.967161
H	1.320535	4.014934	1.220849
H	-1.630166	7.057725	0.463360
H	0.331151	6.227092	1.753558
O	0.416695	2.369760	-0.733590
P	1.159468	1.095282	-0.106026
P	-2.026732	0.147281	0.072523
O	1.493033	1.579473	1.418341
O	2.611048	1.103417	-0.824046
O	-3.295342	-0.876581	-0.094154
O	-2.254556	0.754946	1.573637
C	2.667964	1.244155	2.107938
C	2.519895	0.520806	3.288525
C	3.917125	1.726869	1.679526
C	3.637001	0.250481	4.076823
H	1.529202	0.184445	3.571113
C	5.026470	1.425441	2.490902
C	4.895627	0.701635	3.673036
H	3.522791	-0.303139	5.004346
H	6.008231	1.761358	2.171717
H	5.773518	0.491076	4.276240
C	3.436056	2.238512	-0.749123
C	3.628086	2.974367	-1.913960
C	4.091216	2.542554	0.453744
C	4.500947	4.061067	-1.895891
H	3.103513	2.679296	-2.816576
C	4.960737	3.648552	0.441520
C	5.165159	4.398731	-0.714021
H	4.658141	4.641534	-2.799791
H	5.466608	3.926474	1.361216
H	5.838788	5.249850	-0.690607
C	-4.601754	-0.557273	0.324283
C	-5.578689	-0.442816	-0.660112
C	-4.901945	-0.464291	1.691509
C	-6.903796	-0.216172	-0.292198
H	-5.298107	-0.541621	-1.703198
C	-6.246711	-0.227954	2.030975
C	-7.236318	-0.105971	1.059095
H	-7.667882	-0.123734	-1.057899
H	-6.506803	-0.119633	3.079337
H	-8.263443	0.081734	1.356392
C	-2.609543	-0.021699	2.677422
C	-1.709152	-0.065432	3.739123
C	-3.881570	-0.613876	2.756727
C	-2.052515	-0.739777	4.909422
H	-0.763339	0.454068	3.634496
C	-4.189416	-1.308580	3.941339
C	-3.292797	-1.375829	5.004214
H	-1.357828	-0.762952	5.744135
H	-5.153423	-1.801983	4.018975
H	-3.565440	-1.914570	5.906614
Ir	0.065171	-0.841619	-0.429349
C	-0.870214	-2.973523	-2.076631
C	-0.951791	-2.996010	-0.693489
C	1.870610	-1.770458	-0.623986
H	-1.914200	-2.833547	-0.220468
H	-0.230074	-3.568317	-0.122367
C	2.405512	-2.452337	0.464681
O	2.742019	-1.823549	-1.650928
C	1.565568	-2.437577	1.653221
C	3.699321	-2.946489	0.047241
C	3.845283	-2.535579	-1.236801
O	0.487634	-1.745090	1.602906
N	1.852364	-3.122063	2.780981
H	4.430801	-3.509491	0.605442
H	4.625056	-2.657896	-1.972221
C	3.063080	-3.927874	2.928770
C	0.968269	-3.046855	3.943187
H	2.981031	-4.500892	3.853897
H	3.169029	-4.634167	2.101552
H	3.960197	-3.301211	2.989326
H	0.098870	-2.440743	3.699407
H	0.640890	-4.053560	4.226639
H	1.499353	-2.601459	4.793558
H	-0.065347	-0.363143	-1.932032
C	0.247757	-3.594456	-2.818213
C	0.636789	-3.113164	-4.082835
C	0.905865	-4.727280	-2.301954
C	1.662507	-3.728327	-4.794832
H	0.156481	-2.234990	-4.501334
C	1.921620	-5.350992	-3.022201
H	0.594246	-5.145435	-1.350486
C	2.306342	-4.851051	-4.268493
H	1.957907	-3.333848	-5.762576
H	2.404434	-6.235161	-2.616365
H	3.096981	-5.339318	-4.831107
C	-2.028599	-2.457234	-2.891130
H	-2.814420	-2.058764	-2.249318
H	-1.724169	-1.683912	-3.602119

H	-2.438299	-3.288196	-3.479593

III-L-8 Conformation 6

B3LYP/6-31G(d),SDD(Ir) Energy = -3450.997913
B3LYP/def2-TZVPP,SDD(Ir)/IEFPCM(dioxane) Energy = -3452.169955
B3LYP/def2-TZVPP,SDD(Ir)/IEFPCM(dioxane)//B3LYP/6-31G(d),SDD(Ir) Free Energy (Quasiharmonic) = -3451.456428
Number of Imaginary Frequencies = 0

Frequencies (Top 3 out of 315)

1. 9.8839 cm^{-1}
2. 15.6872 cm^{-1}
3. 18.2492 cm^{-1}

B3LYP/6-31G(d),SDD(Ir) Molecular Geometry in Cartesian Coordinates

O	1.817048	-0.957926	1.813184
C	1.200197	-2.153670	2.179072
C	1.640230	-3.361217	1.642598
C	0.251185	-2.107959	3.210410
C	1.119482	-4.560316	2.131171
H	2.412130	-3.359635	0.885107
C	-0.243887	-3.326714	3.693710
C	0.182045	-4.545560	3.164308
H	1.468012	-5.502187	1.717756
H	-0.980523	-3.306231	4.491410
H	-0.212259	-5.476244	3.561134
C	-0.266724	-0.820283	3.753844
C	-1.252012	-0.124259	3.041512
C	0.131367	-0.305586	4.992795
C	-1.851065	1.030479	3.536747
C	-0.440769	0.863866	5.498168
H	0.891886	-0.832669	5.561355
C	-1.431526	1.527639	4.772306
H	-2.641664	1.512845	2.971500
H	-0.122756	1.245911	6.463586
H	-1.894186	2.425324	5.171947
O	-1.700290	-0.720907	1.865307
P	-1.692892	-0.177106	0.335233
P	1.956348	-0.089816	0.457656
O	-3.112456	0.662773	0.352906
O	-2.106439	-1.556329	-0.418029
O	2.752923	-0.964835	-0.664771
O	3.123464	0.848952	1.094066
C	-4.116602	0.591169	-0.615334
C	-4.485287	1.791670	-1.220777
C	-4.802372	-0.609741	-0.882287
C	-5.539696	1.820619	-2.130657
H	-3.945280	2.692138	-0.952678
C	-5.851012	-0.549469	-1.820169
C	-6.220254	0.641292	-2.438805
H	-5.827301	2.758700	-2.596511
H	-6.376556	-1.466892	-2.066879
H	-7.035282	0.647790	-3.155975
C	-3.190610	-2.336432	0.028026
C	-2.903308	-3.561536	0.620773
C	-4.500091	-1.895262	-0.205962
C	-3.955100	-4.392239	1.004092
H	-1.868059	-3.846610	0.773769
C	-5.539002	-2.752541	0.201189
C	-5.274731	-3.983511	0.796302
H	-3.743405	-5.349956	1.469930
H	-6.566691	-2.431595	0.061596
H	-6.098799	-4.619760	1.104466
C	4.102964	-1.355918	-0.615686
C	4.359120	-2.717381	-0.768695
C	5.138197	-0.406873	-0.560166
C	5.674290	-3.176588	-0.810385
H	3.523712	-3.397375	-0.891958
C	6.454630	-0.905488	-0.589920
C	6.726346	-2.265615	-0.706164
H	5.871741	-4.238190	-0.924043
H	7.275576	-0.200194	-0.507090
H	7.755399	-2.611086	-0.719885
C	3.926837	1.652534	0.284043
C	3.783425	3.034475	0.385348
C	4.912673	1.058602	-0.519421
C	4.624533	3.866588	-0.351718
H	3.029063	3.434405	1.053056
C	5.736079	1.924723	-1.262834
C	5.596167	3.308248	-1.185572
H	4.520113	4.944622	-0.273978
H	6.490173	1.496477	-1.916017
H	6.247416	3.948706	-1.772540
Ir	0.246691	1.021198	-0.489011
C	-0.190289	-0.604045	-2.987913
C	0.724200	0.412497	-2.783618
C	0.108243	2.434964	0.926132
C	-1.590106	-0.246528	-3.427406
C	0.196174	-2.034975	-3.019712
H	1.781658	0.190798	-2.711184
H	0.470177	1.407704	-3.137000
C	-0.621651	3.587120	0.640338
O	0.718526	2.574348	2.115200
H	-1.783671	0.820356	-3.302544
H	-1.695392	-0.502798	-4.490347
H	-2.360538	-0.802270	-2.890200
C	1.519688	-2.413991	-3.324719
C	-0.760645	-3.057436	-2.862081
C	-1.226090	3.578735	-0.672869
C	-0.427892	4.480835	1.761574
C	0.380711	3.816613	2.622233
C	1.871981	-3.755093	-3.452902
H	2.273184	-1.655371	-3.501747
C	-0.404821	-4.399223	-2.976619
H	-1.788298	-2.807711	-2.632096
O	-1.047866	2.518165	-1.385309
N	-1.937214	4.597407	-1.196786
H	-0.821520	5.472780	1.920185
H	0.789478	4.046327	3.593322
C	0.912369	-4.754638	-3.273593
H	2.893692	-4.018029	-3.711633
H	-1.160973	-5.167468	-2.843404
C	-2.223046	5.818514	-0.444364
C	-2.324142	4.596924	-2.608759
H	1.185702	-5.800627	-3.380704
H	-3.007521	6.367018	-0.969166
H	-2.590494	5.578809	0.555263
H	-1.341099	6.465500	-0.365586
H	-2.037395	3.651974	-3.063153
H	-3.407279	4.730808	-2.700528
H	-1.823308	5.419968	-3.132272
H	1.462877	1.941641	-0.994222

III-L-8 Conformation 7

B3LYP/6-31G(d),SDD(Ir) Energy = -3450.998028
B3LYP/def2-TZVPP,SDD(Ir)/IEFPCM(dioxane) Energy = -3452.169697
B3LYP/def2-TZVPP,SDD(Ir)/IEFPCM(dioxane)//B3LYP/6-31G(d),SDD(Ir) Free Energy (Quasiharmonic) = -3451.456017
Number of Imaginary Frequencies = 0

Frequencies (Top 3 out of 315)

1. 5.4217 cm^{-1}
2. 15.9700 cm^{-1}
3. 19.6123 cm^{-1}

B3LYP/6-31G(d),SDD(Ir) Molecular Geometry in Cartesian Coordinates

O	-2.292211	1.804150	0.684238
C	-1.801331	3.080845	0.412551

C	-2.209119	3.764962	-0.729702
C	-1.021708	3.697506	1.402052
C	-1.821322	5.093995	-0.909505
H	-2.861984	3.277758	-1.440984
C	-0.660786	5.037004	1.204931
C	-1.051710	5.734831	0.061326
H	-2.145091	5.629857	-1.796991
H	-0.055468	5.526391	1.962213
H	-0.762265	6.773654	-0.065411
C	-0.547834	2.959320	2.606227
C	0.519390	2.059603	2.483663
C	-1.077892	3.177955	3.882591
C	1.067743	1.405248	3.583245
C	-0.556964	2.515372	4.996128
H	-1.903598	3.874196	3.996611
C	0.513888	1.633003	4.844965
H	1.920388	0.747930	3.452119
H	-0.979093	2.699039	5.979546
H	0.935303	1.130632	5.710836
O	1.084047	1.956622	1.214688
P	1.381170	0.663562	0.276546
P	-2.240735	0.354935	-0.026444
O	2.730541	0.079283	1.012150
O	1.980695	1.452518	-1.013308
O	-2.873614	0.469537	-1.529939
O	-3.485600	-0.206137	0.856938
C	3.968531	-0.198076	0.426487
C	4.484018	-1.470454	0.670340
C	4.711106	0.779217	-0.262887
C	5.756002	-1.809389	0.216823
H	3.879708	-2.172469	1.233323
C	5.986703	0.397479	-0.722400
C	6.509504	-0.870526	-0.489581
H	6.155222	-2.799833	0.414926
H	6.570446	1.121057	-1.282960
H	7.499204	-1.123153	-0.857760
C	2.927239	2.483668	-0.851633
C	2.509737	3.783248	-1.120045
C	4.245447	2.169004	-0.494347
C	3.430477	4.825756	-1.034411
H	1.473384	3.963223	-1.385740
C	5.146661	3.246809	-0.401946
C	4.751131	4.554690	-0.669043
H	3.114255	5.843517	-1.241842
H	6.169198	3.047560	-0.096940
H	5.471726	5.362385	-0.584074
C	-4.223426	0.672736	-1.872372
C	-4.479238	1.708641	-2.769190
C	-5.232889	-0.210111	-1.451049
C	-5.776977	1.932434	-3.224145
H	-3.652097	2.312011	-3.127530
C	-6.535793	0.055321	-1.915137
C	-6.812675	1.109132	-2.780908
H	-5.972543	2.742710	-3.919674
H	-7.344510	-0.582660	-1.572997
H	-7.831874	1.281957	-3.112168
C	-4.140589	-1.388978	0.510210
C	-3.996627	-2.488193	1.353631
C	-4.992495	-1.404815	-0.604771
C	-4.696785	-3.660926	1.075368
H	-3.353794	-2.400575	2.221964
C	-5.672276	-2.608889	-0.868730
C	-5.527717	-3.723081	-0.045787
H	-4.592320	-4.520174	1.731008
H	-6.315880	-2.665487	-1.741128
H	-6.066554	-4.636697	-0.277722
Ir	-0.403731	-0.948171	-0.039582
C	0.374829	-0.864572	-2.851182
C	-0.638206	-1.647515	-2.326886
C	-0.454600	-1.386862	1.920158
H	-1.664606	-1.347388	-2.500607
H	-0.484259	-2.706507	-2.155944
C	0.326721	-2.444657	2.377449
O	-1.216698	-0.923972	2.926100
C	1.124702	-3.062244	1.341008
C	0.004403	-2.614803	3.777445
C	-0.926320	-1.669661	4.054786
O	1.025173	-2.562645	0.156662
N	1.947348	-4.113399	1.529201
H	0.394256	-3.328724	4.486183
H	-1.459087	-1.383579	4.947676
C	2.065708	-4.779281	2.826940
C	2.601676	-4.753158	0.385188
H	2.861209	-5.522649	2.758976
H	2.336028	-4.063915	3.607508
H	1.138101	-5.291307	3.107639
H	2.619839	-4.064194	-0.456451
H	3.626800	-5.017780	0.660010
H	2.067523	-5.667382	0.096149
H	-1.524630	-2.096649	-0.101027
C	1.741425	-1.387353	-3.099914
C	2.015662	-2.771020	-3.069570
C	2.785680	-0.521744	-3.479696
C	3.276673	-3.261594	-3.394060
H	1.231535	-3.476112	-2.820278
C	4.049929	-1.012783	-3.799022
H	2.615913	0.546956	-3.520872
C	4.302163	-2.384081	-3.756889
H	3.455114	-4.333330	-3.384033
H	4.836766	-0.321539	-4.085434
H	5.284250	-2.768409	-4.016965
C	0.028002	0.495402	-3.413568
H	0.666661	1.286578	-3.016176
H	0.170006	0.474897	-4.501827
H	-1.011701	0.748352	-3.202297

III-L-8 Conformation 8

B3LYP/6-31G(d),SDD(Ir) Energy = -3450.996528
B3LYP/def2-TZVPP,SDD(Ir)/IEFPCM(dioxane) Energy = -3452.168542
B3LYP/def2-TZVPP,SDD(Ir)/IEFPCM(dioxane)//B3LYP/6-31G(d),SDD(Ir) Free Energy (Quasiharmonic) = -3451.453770
Number of Imaginary Frequencies = 0

Frequencies (Top 3 out of 315)

1. 9.1074 cm^{-1}
2. 17.1446 cm^{-1}
3. 22.3946 cm^{-1}

B3LYP/6-31G(d),SDD(Ir) Molecular Geometry in Cartesian Coordinates

O	-1.958027	1.304678	-0.345461
C	-2.957167	2.104396	0.226544
C	-3.971478	2.548922	-0.618414
C	-2.857241	2.522739	1.564604
C	-4.937707	3.432073	-0.141736
H	-3.995830	2.193079	-1.641947
C	-3.847705	3.422485	2.007896
C	-4.871625	3.872946	1.179609
H	-5.727639	3.775544	-0.802731
H	-3.786567	3.791573	3.026657
H	-5.606995	4.573052	1.564147
C	-1.834139	2.079238	2.554034
C	-0.445493	2.063310	2.335717
C	-2.266125	1.753822	3.855806
C	0.463168	1.796754	3.360608
C	-1.373744	1.465197	4.883555
H	-3.333408	1.728623	4.052037
C	0.000214	1.497459	4.639143
H	1.525432	1.849792	3.147055
H	-1.751113	1.226299	5.873440
H	0.709434	1.296373	5.436125
O	0.047594	2.430453	1.083778
P	0.937605	1.493082	0.096147
P	-1.693162	-0.282017	-0.413095
O	2.435850	1.897398	0.603554

O	0.785706	2.293224	-1.299228
O	-2.258928	-0.805521	-1.848330
O	-2.832465	-0.812236	0.622474
C	3.522466	2.207734	-0.226981
C	4.677398	1.454226	-0.035291
C	3.486992	3.301971	-1.110557
C	5.833751	1.759731	-0.748805
H	4.649888	0.647654	0.686133
C	4.667037	3.572913	-1.829127
C	5.824553	2.819618	-1.657500
H	6.734823	1.172819	-0.596492
H	4.660181	4.390639	-2.543061
H	6.714938	3.060225	-2.230311
C	0.998997	3.684171	-1.351163
C	-0.108488	4.500836	-1.551425
C	2.306531	4.182039	-1.281648
C	0.078413	5.875064	-1.688293
H	-1.094707	4.052204	-1.599019
C	2.461302	5.574348	-1.414141
C	1.366717	6.410965	-1.616165
H	-0.778661	6.523212	-1.843881
H	3.457199	5.999659	-1.337363
H	1.518557	7.481987	-1.709484
C	-3.600420	-0.698728	-2.248962
C	-3.864951	0.108264	-3.353002
C	-4.593002	-1.465375	-1.620259
C	-5.167127	0.188514	-3.845057
H	-3.046458	0.649537	-3.817055
C	-5.898746	-1.352418	-2.132667
C	-6.186696	-0.538392	-3.225242
H	-5.381396	0.813706	-4.706526
H	-6.697297	-1.907631	-1.650330
H	-7.205841	-0.473014	-3.593666
C	-3.420235	-2.082316	0.546278
C	-3.183361	-2.957337	1.603989
C	-4.295816	-2.398223	-0.507128
C	-3.818830	-4.198165	1.623057
H	-2.511022	-2.643074	2.393349
C	-4.914181	-3.661598	-0.461151
C	-4.684053	-4.551297	0.585116
H	-3.638920	-4.883172	2.446206
H	-5.574582	-3.947277	-1.274149
H	-5.176865	-5.518723	0.587520
Ir	0.481273	-0.738642	-0.111486
C	0.741669	-2.934866	-1.688933
C	0.149188	-3.050071	-0.434032
C	0.486242	-1.269210	1.928637
H	-0.923356	-3.208278	-0.369237
H	0.727128	-3.452464	0.391204
C	1.712626	-1.656622	2.467399
O	-0.463546	-1.435301	2.877940
C	2.808830	-1.607376	1.525469
C	1.468653	-2.087867	3.827411
C	0.139028	-1.923975	4.021383
O	2.538387	-1.238044	0.318764
N	4.083882	-1.937640	1.819074
H	2.160052	-2.468857	4.562794
H	-0.516338	-2.089806	4.862268
C	4.508221	-2.307374	3.167760
C	5.088091	-2.085927	0.764158
H	5.597050	-2.238776	3.212852
H	4.095979	-1.616624	3.905115
H	4.216045	-3.333256	3.424068
H	4.660496	-1.794314	-0.191558
H	5.957871	-1.458581	0.988717
H	5.415241	-3.130244	0.707209
H	0.795016	-0.436769	-1.701152
C	2.177738	-3.245547	-1.907194
C	2.952638	-2.527293	-2.835048
C	2.744690	-4.369248	-1.280554
C	4.259847	-2.915373	-3.117840
H	2.538146	-1.645120	-3.313044
C	4.044726	-4.773530	-1.586790
H	2.148960	-4.958615	-0.589927
C	4.806516	-4.046688	-2.503382
H	4.849413	-2.342278	-3.827579
H	4.455444	-5.663946	-1.119037
H	5.817429	-4.362731	-2.744945
C	-0.088761	-2.824692	-2.941930
H	-1.107968	-2.506315	-2.736359
H	0.356349	-2.148667	-3.675760
H	-0.109446	-3.823078	-3.401603

III-L-8 Conformation 9

B3LYP/6-31G(d),SDD(Ir) Energy = -3450.983621
B3LYP/def2-TZVPP,SDD(Ir)/IEFPCM(dioxane) Energy = -3452.152446
B3LYP/def2-TZVPP,SDD(Ir)/IEFPCM(dioxane)//B3LYP/6-31G(d),SDD(Ir) Free Energy (Quasiharmonic) = -3451.437712
Number of Imaginary Frequencies = 0

Frequencies (Top 3 out of 315)

1. 13.5693 cm^{-1}
2. 17.5212 cm^{-1}
3. 20.7542 cm^{-1}

B3LYP/6-31G(d),SDD(Ir) Molecular Geometry in Cartesian Coordinates

O	-1.954451	1.316473	1.509109
C	-1.225396	2.489777	1.691458
C	-1.518542	3.625010	0.939584
C	-0.298126	2.522784	2.742318
C	-0.886414	4.831266	1.244053
H	-2.249132	3.567012	0.143127
C	0.311099	3.748746	3.039194
C	0.018531	4.898837	2.303906
H	-1.118702	5.717629	0.661463
H	1.029481	3.788914	3.852752
H	0.495130	5.840766	2.558901
C	0.085176	1.285685	3.480861
C	1.052572	0.434497	2.928133
C	-0.431597	0.961666	4.740502
C	1.522917	-0.688651	3.603699
C	0.012633	-0.170391	5.426070
H	-1.182064	1.610035	5.182872
C	0.991682	-0.988946	4.859480
H	2.307191	-1.292510	3.159822
H	-0.393504	-0.402555	6.405884
H	1.359856	-1.854428	5.403282
O	1.624463	0.848607	1.729450
P	1.762055	0.168015	0.261265
P	-1.998850	0.248917	0.281706
O	3.044035	-0.829367	0.557175
O	2.471969	1.412995	-0.506364
O	-3.039070	0.851344	-0.830752
O	-2.947974	-0.782533	1.124857
C	4.126407	-0.999616	-0.316042
C	4.346582	-2.282064	-0.814015
C	5.016912	0.058152	-0.582621
C	5.461448	-2.537727	-1.610600
H	3.643928	-3.067642	-0.563787
C	6.126161	-0.229789	-1.398819
C	6.352243	-1.505500	-1.908625
H	5.629014	-3.539113	-1.995860
H	6.813370	0.575151	-1.641171
H	7.218393	-1.690643	-2.536537
C	3.602859	2.057831	0.033631
C	3.438472	3.347670	0.527556
C	4.848790	1.419456	-0.020953
C	4.555724	4.044616	0.986186
H	2.446100	3.785094	0.550667
C	5.954746	2.146088	0.455873
C	5.814776	3.440523	0.950578
H	4.440759	5.051707	1.375620
H	6.932064	1.673236	0.451285
H	6.686958	3.973888	1.316193

C	-4.385466	1.162594	-0.568332
C	-4.777671	2.488753	-0.730622
C	-5.307977	0.145588	-0.273698
C	-6.116523	2.839611	-0.564396
H	-4.036234	3.228273	-1.011228
C	-6.649654	0.531854	-0.103041
C	-7.053672	1.857232	-0.240704
H	-6.422163	3.874321	-0.686405
H	-7.379393	-0.228904	0.156652
H	-8.096708	2.121297	-0.095996
C	-3.764839	-1.712418	0.479803
C	-3.447646	-3.062593	0.610012
C	-4.926463	-1.283468	-0.183015
C	-4.291618	-4.026308	0.060348
H	-2.548111	-3.332142	1.150297
C	-5.753179	-2.277733	-0.737062
C	-5.445202	-3.630888	-0.620398
H	-4.048848	-5.079903	0.162814
H	-6.644780	-1.973150	-1.276669
H	-6.103416	-4.373731	-1.060528
Ir	-0.197051	-0.901436	-0.737499
C	0.175306	0.732124	-2.865329
C	-0.765356	-0.308276	-2.839472
C	0.842976	-2.501066	-1.447406
C	1.552652	0.445452	-3.415964
C	-0.216672	2.159423	-2.744298
H	-1.820792	-0.068404	-2.794849
H	-0.530175	-1.204709	-3.404753
C	0.968756	-3.607118	-0.610770
O	1.298958	-2.830947	-2.674827
H	1.757379	-0.624471	-3.448185
H	1.578042	0.836747	-4.443024
H	2.349327	0.944284	-2.864373
C	-1.530380	2.567785	-3.058212
C	0.739377	3.162656	-2.485319
C	0.451440	-3.381468	0.716407
C	1.538707	-4.674920	-1.404348
C	1.721845	-4.148786	-2.639030
C	-1.871020	3.917665	-3.104731
H	-2.284699	1.832066	-3.307651
C	0.393384	4.511065	-2.517347
H	1.757259	2.888013	-2.244781
O	-0.081593	-2.222381	0.949484
N	0.476338	-4.278152	1.717226
H	1.774331	-5.688875	-1.120089
H	2.116710	-4.536555	-3.565080
C	-0.912043	4.895849	-2.830609
H	-2.881615	4.205300	-3.380822
H	1.149744	5.263007	-2.312152
C	1.098724	-5.593394	1.565464
C	-0.184356	-4.008832	2.997055
H	-1.175843	5.948690	-2.878960
H	1.199611	-6.039236	2.556326
H	0.490063	-6.261997	0.945546
H	2.096994	-5.502817	1.131572
H	-0.586657	-2.998993	2.997657
H	-0.993606	-4.731217	3.156749
H	0.538939	-4.106996	3.813004
H	-1.432885	-1.798104	-1.220719

III-L-8 Conformation 10

B3LYP/6-31G(d),SDD(Ir) Energy = -3450.983182
B3LYP/def2-TZVPP,SDD(Ir)/IEFPCM(dioxane) Energy = -3452.151957
B3LYP/def2-TZVPP,SDD(Ir)/IEFPCM(dioxane)//B3LYP/6-31G(d),SDD(Ir) Free Energy (Quasiharmonic) = -3451.437228
Number of Imaginary Frequencies = 0

Frequencies (Top 3 out of 315)

1. 14.5283 cm^{-1}
2. 16.8598 cm^{-1}
3. 21.6566 cm^{-1}

B3LYP/6-31G(d),SDD(Ir) Molecular Geometry in Cartesian Coordinates

O	-2.385060	1.944202	0.803395
C	-1.585651	3.084489	0.836574
C	-1.707318	4.041475	-0.170889
C	-0.776225	3.300857	1.959546
C	-0.998721	5.238978	-0.073698
H	-2.385547	3.860094	-0.995537
C	-0.079775	4.515234	2.038629
C	-0.185349	5.479131	1.035205
H	-1.101191	5.988488	-0.853048
H	0.550772	4.696406	2.904089
H	0.354391	6.417095	1.125990
C	-0.625852	2.274985	3.032243
C	0.392283	1.317338	2.936716
C	-1.427673	2.269013	4.180488
C	0.641808	0.399814	3.955024
C	-1.204770	1.346467	5.203674
H	-2.222223	3.003782	4.270319
C	-0.165796	0.419544	5.092861
H	1.470906	-0.293433	3.858311
H	-1.828933	1.364215	6.091984
H	0.029770	-0.279538	5.901180
O	1.236771	1.404403	1.835585
P	1.489285	0.378537	0.594387
P	-2.236965	0.595778	-0.106339
O	2.668300	-0.540101	1.293119
O	2.348093	1.374054	-0.363396
O	-2.934039	0.941778	-1.554114
O	-3.444181	-0.153289	0.699267
C	3.812678	-0.992055	0.628349
C	3.990575	-2.370294	0.528502
C	4.796897	-0.085459	0.191908
C	5.161038	-2.881007	-0.029515
H	3.212176	-3.026062	0.900503
C	5.960820	-0.632600	-0.378260
C	6.148020	-2.007795	-0.489497
H	5.298223	-3.955888	-0.102122
H	6.725697	0.045042	-0.745207
H	7.061056	-2.394572	-0.932134
C	3.474649	2.072427	0.106752
C	3.368090	3.454575	0.228392
C	4.671181	1.383117	0.351344
C	4.487616	4.194767	0.605204
H	2.413145	3.931568	0.033863
C	5.780060	2.157847	0.738809
C	5.695637	3.542386	0.862155
H	4.413241	5.273536	0.704611
H	6.715038	1.653872	0.963661
H	6.569130	4.109891	1.168519
C	-4.273116	1.331513	-1.728895
C	-4.500284	2.568626	-2.326970
C	-5.323756	0.450261	-1.424752
C	-5.804170	2.980190	-2.597213
H	-3.652535	3.187016	-2.601985
C	-6.628961	0.898688	-1.698079
C	-6.872987	2.144536	-2.269814
H	-5.980020	3.946291	-3.060292
H	-7.462810	0.252334	-1.442046
H	-7.893937	2.459594	-2.461979
C	-4.172572	-1.193762	0.120583
C	-4.018147	-2.472203	0.651208
C	-5.104951	-0.915776	-0.891796
C	-4.797117	-3.520074	0.163486
H	-3.294654	-2.618580	1.444412
C	-5.868302	-1.995543	-1.372516
C	-5.720965	-3.280490	-0.856212
H	-4.681815	-4.517819	0.576589
H	-6.578452	-1.815938	-2.173896
H	-6.324744	-4.092256	-1.250263
Ir	-0.370408	-0.830091	-0.397599
C	0.615725	0.359359	-2.826437
C	-0.362561	-0.640046	-2.657102

C	0.606266	-2.614175	-0.470886
H	-1.384923	-0.330782	-2.852136
H	-0.139090	-1.645773	-2.993582
C	0.475416	-3.460865	0.628310
O	1.218823	-3.290880	-1.466029
C	-0.255488	-2.872474	1.723376
C	1.051835	-4.734119	0.254289
C	1.488998	-4.571965	-1.017162
O	-0.716521	-1.675703	1.543490
N	-0.500208	-3.474471	2.901140
H	1.128282	-5.645874	0.826418
H	1.987244	-5.218448	-1.722636
C	0.034608	-4.795724	3.226507
C	-1.385086	-2.864456	3.896244
H	-0.042632	-4.938875	4.305935
H	-0.526217	-5.596942	2.730249
H	1.088828	-4.864529	2.951120
H	-1.709421	-1.886831	3.548736
H	-2.257106	-3.508464	4.061765
H	-0.850865	-2.747759	4.844687
H	-1.562005	-1.760509	-0.936288
C	0.168784	1.799535	-2.786566
H	0.332970	2.247987	-3.774791
H	-0.891219	1.872243	-2.548393
H	0.737155	2.388257	-2.062449
C	1.999819	0.081637	-3.234987
C	2.424420	-1.219618	-3.585751
C	2.924075	1.139850	-3.390718
C	3.709096	-1.447852	-4.065054
H	1.751711	-2.059616	-3.481475
C	4.206521	0.910217	-3.874415
H	2.642452	2.151179	-3.125655
C	4.604782	-0.385555	-4.213312
H	4.010960	-2.455991	-4.332476
H	4.895865	1.741428	-3.987805
H	5.605294	-0.565681	-4.596141

TS-III-L-5 Conformation 1

B3LYP/6-31G(d),SDD(Ir) Energy = -3922.722407
B3LYP/def2-TZVPP,SDD(Ir)/IEFPCM(dioxane) Energy = -3924.066043
B3LYP/def2-TZVPP,SDD(Ir)/IEFPCM(dioxane)//B3LYP/6-31G(d),SDD(Ir) Free Energy (Quasiharmonic) = -3923.037395
Number of Imaginary Frequencies = 1

Frequencies (Top 3 out of 423)

1. -363.9740 cm^{-1}
2. 9.4483 cm^{-1}
3. 23.4952 cm^{-1}

B3LYP/6-31G(d),SDD(Ir) Molecular Geometry in Cartesian Coordinates

C	0.196773	2.733079	-1.279000
C	1.490853	2.814993	-1.974390
C	2.427666	1.243288	-1.112410
C	3.241727	1.491954	0.009990
O	3.247393	0.710924	-2.084130
C	4.561295	1.086198	-0.303830
C	4.498193	0.624118	-1.594680
H	5.244541	0.215235	-2.260670
H	5.440885	1.085524	0.322760
H	0.055946	3.425269	-0.454850
H	-0.650337	2.757793	-1.964520
C	2.569499	1.920588	1.246010
O	1.329568	1.676023	1.334160
N	3.222452	2.525614	2.256210
C	2.529283	2.766258	3.521530
H	3.230702	2.599314	4.344340
H	2.161788	3.798789	3.574160
H	1.690600	2.078832	3.612780
C	4.566364	3.089958	2.146440
H	4.575109	4.066348	2.640800
H	5.309581	2.448545	2.635710
H	4.837825	3.242117	1.102760
Ir	0.406063	0.712418	-0.526610
P	-1.787066	0.790248	0.099940
P	1.018383	-1.494245	-0.140010
O	-2.705912	-0.410798	0.688380
O	-2.152401	1.895740	1.265330
O	-2.687374	1.320372	-1.148610
O	0.335167	-2.665172	-1.045750
O	2.611982	-1.697353	-0.480900
O	0.946439	-2.242155	1.325090
C	-2.396998	-1.617919	1.347010
C	-2.254735	3.267840	1.059940
C	-4.017362	1.747188	-1.029150
C	-0.895082	-2.603916	-1.731790
C	3.355857	-2.849106	-0.196100
C	1.844031	-1.929449	2.344800
C	-2.015683	-2.701141	0.530880
C	-2.620278	-1.746608	2.734200
C	-1.351281	4.086436	1.737320
C	-3.305112	3.796465	0.289920
C	-4.982375	0.996853	-1.694690
C	-4.327536	2.945380	-0.365470
C	-0.904873	-2.706576	-3.138180
C	-2.071432	-2.583691	-0.963220
C	3.877483	-3.558189	-1.276020
C	3.664765	-3.187898	1.131740
C	1.358214	-1.244237	3.456420
C	3.165419	-2.405145	2.284840
C	-1.704559	-3.945552	1.117170
C	-2.252734	-2.992010	3.267090
C	-1.466404	5.472277	1.639200
C	-3.382386	5.198786	0.195400
C	-6.309493	1.421709	-1.690420
C	-5.678334	3.341456	-0.369310
C	-2.176733	-2.669270	-3.724420
C	-3.331742	-2.604715	-1.602530
C	4.711048	-4.651773	-1.046590
H	3.628545	-3.241327	-2.282310
C	4.502620	-4.299922	1.332070
C	2.198925	-1.010131	4.543870
H	0.321066	-0.930932	3.461470
C	3.993590	-2.143969	3.391630
C	-1.780279	-4.067172	2.515220
H	-2.361295	-3.151209	4.332470
C	-2.480992	6.029251	0.856210
H	-0.770821	6.112853	2.173980
C	-6.658158	2.595161	-1.018120
H	-7.065426	0.838493	-2.207720
C	-3.375082	-2.610725	-3.007490
H	-2.253053	-2.720660	-4.803070
C	5.020426	-5.026014	0.262750
H	5.112745	-5.208794	-1.887760
H	4.731798	-4.602393	2.349510
C	3.522363	-1.457357	4.508480
H	1.817358	-0.490469	5.418310
H	5.024158	-2.485594	3.364010
H	5.658852	-5.883767	0.451230
H	4.184054	-1.279510	5.351230
H	-0.117259	0.203560	-1.917270
H	-0.575163	3.616732	2.332000
H	-4.670160	0.100442	-2.218270
H	-7.691296	2.928266	-0.997360
H	-5.956970	4.245768	0.163000
H	-4.164324	5.636009	-0.417850
H	-2.572677	7.107102	0.762750
C	-1.405575	-5.354274	3.213240
H	-1.463075	-5.241634	4.299860
H	-2.069489	-6.181911	2.931840
H	-0.385147	-5.667729	2.961650
C	-1.290534	-5.139194	0.288490
H	-0.201935	-5.278459	0.324130
H	-1.745139	-6.060032	0.667690
H	-1.572934	-5.033653	-0.759820

C	-4.605173	-2.665299	-0.786910
H	-4.900218	-1.672387	-0.425420
H	-5.437044	-3.062285	-1.372960
H	-4.483216	-3.301879	0.093620
C	-4.682962	-2.627878	-3.770530
H	-4.504752	-2.546909	-4.846780
H	-5.241837	-3.556956	-3.600010
H	-5.345559	-1.803235	-3.482600
C	0.347566	-2.964112	-4.008990
C	-3.269983	-0.687585	3.666370
C	-2.222118	0.354640	4.123330
H	-1.756236	0.869828	3.282250
H	-2.699895	1.113432	4.755700
H	-1.438991	-0.126774	4.721900
C	-3.836206	-1.348822	4.947910
H	-4.541130	-2.154559	4.715620
H	-3.055208	-1.750796	5.601610
H	-4.374023	-0.591060	5.527450
C	-4.471300	0.013801	2.986280
H	-4.180747	0.632869	2.140440
H	-5.203203	-0.724256	2.637480
H	-4.973037	0.658173	3.717380
C	1.360101	-1.801127	-3.919010
H	0.903256	-0.863425	-4.255670
H	1.742202	-1.647199	-2.911030
H	2.216700	-2.005871	-4.573940
C	-0.010775	-3.122220	-5.503260
H	0.905694	-3.325725	-6.067600
H	-0.694839	-3.958847	-5.680340
H	-0.457310	-2.213408	-5.922550
C	1.005690	-4.293255	-3.560980
H	1.314640	-4.270767	-2.514490
H	0.309176	-5.129292	-3.692390
H	1.890389	-4.498819	-4.176590
C	2.462728	3.925018	-1.682330
C	2.179573	4.926319	-0.733840
C	3.682079	4.010772	-2.385690
C	3.080468	5.964585	-0.497540
H	1.240543	4.920954	-0.193400
C	4.583124	5.045268	-2.143330
H	3.935655	3.263491	-3.128440
C	4.288058	6.027789	-1.195590
H	2.828162	6.736856	0.223950
H	5.513074	5.088784	-2.703100
H	4.984912	6.840976	-1.014060
C	1.409621	2.406933	-3.439230
H	0.724957	1.568266	-3.570720
H	1.011035	3.272425	-3.984560
H	2.368330	2.144638	-3.884230

TS-III-L-5 Conformation 2

B3LYP/6-31G(d),SDD(Ir) Energy = -3922.722407
B3LYP/def2-TZVPP,SDD(Ir)/IEFPCM(dioxane) Energy = -3924.066042
B3LYP/def2-TZVPP,SDD(Ir)/IEFPCM(dioxane)//B3LYP/6-31G(d),SDD(Ir) Free Energy (Quasiharmonic) = -3923.037393
Number of Imaginary Frequencies = 1

Frequencies (Top 3 out of 423)

1. -363.9752 cm^{-1}
2. 9.4485 cm^{-1}
3. 23.4928 cm^{-1}

B3LYP/6-31G(d),SDD(Ir) Molecular Geometry in Cartesian Coordinates

C	0.196745	2.733054	-1.279046
C	1.490803	2.814957	-1.974479
C	2.427640	1.243257	-1.112529
C	3.241746	1.491928	0.009827
O	3.247317	0.710880	-2.084279
C	4.561302	1.086162	-0.304038
C	4.498138	0.624068	-1.594880
H	5.244460	0.215175	-2.260909
H	5.440924	1.085488	0.322510
H	0.055954	3.425260	-0.454904
H	-0.650389	2.757758	-1.964535
C	2.569579	1.920572	1.245880
O	1.329648	1.676024	1.334087
N	3.222585	2.525603	2.256049
C	2.529470	2.766258	3.521391
H	3.230937	2.599350	4.344175
H	2.161948	3.798775	3.574019
H	1.690814	2.078805	3.612705
C	4.566492	3.089943	2.146211
H	4.575261	4.066344	2.640560
H	5.309732	2.448533	2.635451
H	4.837900	3.242096	1.102511
Ir	0.406056	0.712404	-0.526635
P	-1.787052	0.790265	0.100004
P	1.018369	-1.494254	-0.140028
O	-2.705896	-0.410774	0.688460
O	-2.152321	1.895759	1.265408
O	-2.687398	1.320414	-1.148512
O	0.335116	-2.665179	-1.045745
O	2.611954	-1.697388	-0.480950
O	0.946446	-2.242149	1.325085
C	-2.396977	-1.617900	1.347083
C	-2.254642	3.267857	1.060037
C	-4.017370	1.747243	-1.028993
C	-0.895152	-2.603918	-1.731750
C	3.355824	-2.849144	-0.196148
C	1.844059	-1.929441	2.344773
C	-2.015690	-2.701126	0.530949
C	-2.620228	-1.746579	2.734280
C	-1.351150	4.086431	1.737382
C	-3.305045	3.796501	0.290057
C	-4.982428	0.996928	-1.694505
C	-4.327507	2.945434	-0.365292
C	-0.904970	-2.706584	-3.138142
C	-2.071479	-2.583672	-0.963156
C	3.877420	-3.558250	-1.276078
C	3.664757	-3.187928	1.131683
C	1.358279	-1.244204	3.456395
C	3.165446	-2.405155	2.284790
C	-1.704562	-3.945539	1.117234
C	-2.252681	-2.991991	3.267165
C	-1.466250	5.472277	1.639267
H	-0.575013	3.616717	2.332027
C	-3.382303	5.198824	0.195551
C	-6.309536	1.421801	-1.690176
H	-4.670238	0.100513	-2.218104
C	-5.678295	3.341532	-0.369081
C	-2.176846	-2.669258	-3.724346
C	-3.331798	-2.604684	-1.602432
C	4.710978	-4.651837	-1.046647
H	3.628457	-3.241393	-2.282363
C	4.502599	-4.299954	1.332009
C	2.199011	-1.010101	4.543817
H	0.321129	-0.930893	3.461462
C	3.993639	-2.143978	3.391562
C	-1.780254	-4.067152	2.515286
H	-2.361220	-3.151190	4.332550
C	-2.480865	6.029277	0.856325
H	-0.770637	6.112844	2.174026
H	-4.164255	5.636062	-0.417670
C	-6.658157	2.595252	-1.017851
H	-7.065499	0.838599	-2.207451
H	-5.956895	4.245845	0.163249
C	-3.375172	-2.610690	-3.007389
H	-2.253196	-2.720653	-4.802993
C	5.020377	-5.026067	0.262685
H	5.112650	-5.208871	-1.887821
H	4.731796	-4.602422	2.349446
C	3.522438	-1.457348	4.508410
H	1.817471	-0.490425	5.418263
H	5.024199	-2.485610	3.363923

H	-2.572537	7.107129	0.762881
H	-7.691289	2.928369	-0.997044
H	5.658800	-5.883827	0.451162
H	4.184149	-1.279499	5.351143
H	-0.117329	0.203527	-1.917272
C	-4.683077	-2.627826	-3.770397
H	-4.504891	-2.546863	-4.846659
H	-5.241962	-3.556892	-3.599861
H	-5.345646	-1.803166	-3.482453
C	-4.605218	-2.665243	-0.786782
H	-4.900242	-1.672329	-0.425285
H	-5.437108	-3.062222	-1.372808
H	-4.483245	-3.301827	0.093744
C	-1.290568	-5.139187	0.288541
H	-1.745181	-6.060013	0.667755
H	-1.572996	-5.033645	-0.759761
H	-0.201975	-5.278454	0.324161
C	-1.405545	-5.354259	3.213298
H	-1.463012	-5.241620	4.299916
H	-2.069467	-6.181888	2.931914
H	-0.385116	-5.667722	2.961679
C	0.347436	-2.964149	-4.008983
C	-3.269909	-0.687560	3.666463
C	-4.471233	0.013841	2.986399
H	-4.180678	0.632915	2.140560
H	-5.203145	-0.724203	2.637601
H	-4.972947	0.658221	3.717511
C	-3.836116	-1.348795	4.948009
H	-4.541058	-2.154521	4.715727
H	-3.055114	-1.750781	5.601694
H	-4.373919	-0.591032	5.527566
C	-2.222028	0.354658	4.123417
H	-1.756153	0.869836	3.282330
H	-2.699784	1.113446	4.755798
H	-1.438895	-0.126770	4.721966
C	-0.010941	-3.122261	-5.503235
H	-0.457468	-2.213443	-5.922521
H	0.905516	-3.325780	-6.067600
H	-0.695022	-3.958877	-5.680298
C	1.005554	-4.293292	-3.560976
H	1.314536	-4.270799	-2.514489
H	0.309028	-5.129319	-3.692367
H	1.890235	-4.498876	-4.176606
C	1.359996	-1.801171	-3.919026
H	0.903155	-0.863465	-4.255683
H	1.742129	-1.647252	-2.911056
H	2.216575	-2.005942	-4.573978
C	1.409512	2.406886	-3.439317
H	0.724831	1.568231	-3.570777
H	1.010919	3.272383	-3.984642
H	2.368203	2.144575	-3.884349
C	2.462683	3.924986	-1.682466
C	2.179591	4.926265	-0.733942
C	3.681994	4.010756	-2.385899
C	3.080491	5.964534	-0.497681
H	1.240594	4.920886	-0.193434
C	4.583042	5.045260	-2.143582
H	3.935524	3.263502	-3.128685
C	4.288034	6.027762	-1.195803
H	2.828231	6.736793	0.223839
H	5.512958	5.088790	-2.703416
H	4.984893	6.840951	-1.014310

TS-III-L-5 Conformation 3

B3LYP/6-31G(d),SDD(Ir) Energy = -3922.719236
B3LYP/def2-TZVPP,SDD(Ir)/IEFPCM(dioxane) Energy = -3924.062877
B3LYP/def2-TZVPP,SDD(Ir)/IEFPCM(dioxane)//B3LYP/6-31G(d),SDD(Ir) Free Energy (Quasiharmonic) = -3923.033666
Number of Imaginary Frequencies = 1

Frequencies (Top 3 out of 423)

1. -362.2048 cm^{-1}
2. 8.8396 cm^{-1}
3. 18.6666 cm^{-1}

B3LYP/6-31G(d),SDD(Ir) Molecular Geometry in Cartesian Coordinates

C	1.135550	2.870290	-0.224290
C	0.025170	3.825780	-0.042050
C	-1.548030	2.598150	-0.287170
C	-2.192590	2.586240	-1.541190
O	-2.498570	2.962040	0.640030
C	-3.551560	2.938790	-1.339830
C	-3.668710	3.155930	0.010780
H	-4.503570	3.445610	0.632710
H	-4.357650	2.987910	-2.056700
H	1.616040	2.970490	-1.196770
H	1.876590	2.870360	0.570430
C	-0.161550	4.788290	-1.212380
H	0.587000	5.584740	-1.105780
H	0.029460	4.283910	-2.161130
H	-1.149620	5.251060	-1.251270
C	-0.196210	4.419320	1.319350
C	-0.928980	5.611270	1.475690
C	0.364260	3.839350	2.473150
C	-1.080200	6.208110	2.726030
H	-1.380640	6.095580	0.617680
C	0.214680	4.435890	3.722670
H	0.919650	2.911950	2.401130
C	-0.507730	5.623920	3.856240
H	-1.641550	7.133850	2.813940
H	0.667360	3.971960	4.594320
H	-0.620960	6.089850	4.830860
C	-1.426570	2.045760	-2.678770
O	-0.418060	1.333030	-2.397170
N	-1.763780	2.277420	-3.962530
C	-1.052410	1.585580	-5.038200
H	-1.780960	1.225010	-5.770820
H	-0.354360	2.268050	-5.538470
H	-0.504340	0.741580	-4.625680
C	-2.778530	3.237260	-4.390900
H	-2.413960	3.754070	-5.283870
H	-3.721170	2.736150	-4.643350
H	-2.957060	3.983840	-3.618100
Ir	-0.024460	1.045530	-0.154070
P	1.941370	-0.105990	-0.251270
P	-1.616280	-0.605540	0.235320
O	2.183910	-1.699780	-0.065910
O	2.784320	0.056460	-1.656000
O	2.985400	0.512550	0.834950
O	-1.594300	-1.393830	1.662190
O	-3.121390	0.051250	0.239700
O	-1.876660	-1.898940	-0.751200
C	1.349560	-2.823510	-0.229970
C	3.533620	1.173700	-2.014190
C	4.357230	0.221860	0.850510
C	-0.498380	-1.535430	2.537480
C	-4.316820	-0.677690	0.277970
C	-2.504220	-1.761780	-1.987530
C	0.485840	-3.127590	0.840780
C	1.506440	-3.667300	-1.349880
C	3.128490	1.880730	-3.145600
C	4.712210	1.493420	-1.318360
C	4.845390	-0.457140	1.963180
C	5.202330	0.705140	-0.161740
C	-0.572120	-0.972750	3.828210
C	0.564000	-2.357980	2.125870
C	-5.130700	-0.516130	1.397230
C	-4.725320	-1.434880	-0.832440
C	-1.734040	-1.945310	-3.134020
C	-3.894890	-1.565080	-2.051170
C	-0.370460	-4.245180	0.755360
C	0.597920	-4.735990	-1.401610
C	3.893250	2.953450	-3.600340
C	5.453180	2.591910	-1.792920

C	6.213400	-0.695530	2.079560
C	6.577230	0.437990	-0.020990
C	0.555850	-1.188270	4.630830
C	1.652470	-2.591470	2.996670
C	-6.378000	-1.137210	1.439680
H	-4.779450	0.097960	2.218420
C	-5.985690	-2.055260	-0.757600
C	-2.346210	-1.929680	-4.386590
H	-0.671500	-2.125260	-3.021920
C	-4.482740	-1.540870	-3.329110
C	-0.340780	-5.026620	-0.412360
H	0.633500	-5.404620	-2.252450
C	5.055830	3.314680	-2.914870
H	3.583170	3.503590	-4.484070
C	7.080360	-0.251940	1.078510
H	6.597950	-1.226040	2.945500
C	1.658420	-1.960020	4.252590
H	0.574000	-0.762500	5.626120
C	-6.804270	-1.913110	0.359690
H	-7.010160	-1.016790	2.314360
H	-6.312380	-2.672410	-1.589200
C	-3.724640	-1.721440	-4.484030
H	-1.749690	-2.090190	-5.280260
H	-5.553040	-1.374030	-3.407620
H	-7.768920	-2.410551	0.389140
H	-4.210230	-1.708840	-5.455530
H	0.179450	1.056060	1.401410
H	2.217690	1.573250	-3.648340
H	4.143040	-0.773110	2.726220
H	8.146380	-0.444040	1.152470
H	7.254110	0.769220	-0.802570
H	6.352140	2.882580	-1.258050
H	5.652790	4.156100	-3.253320
C	-1.272540	-6.202600	-0.595130
H	-1.152660	-6.646520	-1.587740
H	-1.086350	-6.992560	0.143960
H	-2.322790	-5.906770	-0.484250
C	-1.314440	-4.617570	1.874950
H	-2.335640	-4.282130	1.650620
H	-1.355170	-5.702730	2.012540
H	-1.025000	-4.170380	2.826890
C	2.761450	-3.541120	2.597820
H	3.477300	-3.063280	1.917850
H	3.319020	-3.894260	3.467920
H	2.364450	-4.417480	2.077700
C	2.789720	-2.148400	5.241570
H	2.643540	-1.516150	6.122260
H	2.857040	-3.186420	5.591500
H	3.766280	-1.895290	4.812200
C	-1.825320	-0.277870	4.411360
C	2.595950	-3.549080	-2.450690
C	2.172760	-2.525220	-3.530360
H	1.993970	-1.533420	-3.112740
H	2.961820	-2.430920	-4.286900
H	1.263980	-2.863440	-4.043240
C	2.804500	-4.904320	-3.172090
H	3.037670	-5.710980	-2.468510
H	1.938970	-5.207120	-3.770380
H	3.648410	-4.809470	-3.863530
C	3.974370	-3.170710	-1.855670
H	3.997050	-2.167660	-1.435790
H	4.264630	-3.879170	-1.070870
H	4.733220	-3.221270	-2.644980
C	-2.195570	1.002610	3.631790
H	-1.380070	1.732820	3.662060
H	-2.432150	0.809060	2.586940
H	-3.074820	1.471780	4.091900
C	-1.618030	0.144560	5.882570
H	-2.538840	0.609880	6.250460
H	-1.400920	-0.708310	6.534380
H	-0.813530	0.880570	5.993950
C	-3.004630	-1.283030	4.392830
H	-3.244490	-1.620310	3.382710
H	-2.768340	-2.164770	4.999580
H	-3.900620	-0.816050	4.820610

TS-III-L-5 Conformation 4

B3LYP/6-31G(d),SDD(Ir) Energy = -3922.658159
B3LYP/def2-TZVPP,SDD(Ir)/IEFPCM(dioxane) Energy = -3924.007789
B3LYP/def2-TZVPP,SDD(Ir)/IEFPCM(dioxane)//B3LYP/6-31G(d),SDD(Ir) Free Energy (Quasiharmonic) = -3922.983031
Number of Imaginary Frequencies = 1

Frequencies (Top 3 out of 423)

1. -274.8907 cm^{-1}
2. 8.4367 cm^{-1}
3. 11.0922 cm^{-1}

B3LYP/6-31G(d),SDD(Ir) Molecular Geometry in Cartesian Coordinates

C	-0.925634	2.790209	0.316530
C	-2.293144	2.807167	-0.250860
C	-2.712652	0.994966	0.769170
C	-4.030691	0.472555	0.620050
O	-2.714533	1.596256	2.054840
C	-4.776602	0.805984	1.772850
C	-3.924833	1.494685	2.598560
H	-4.054553	1.931545	3.578950
H	-5.805311	0.543372	1.977060
H	-0.237105	3.297160	-0.366980
H	-0.819895	3.207879	1.314590
C	-2.304484	2.371707	-1.709120
H	-2.008825	3.221827	-2.337940
H	-1.561403	1.579948	-1.896270
H	-3.264713	1.993516	-2.055430
C	-3.310396	3.793896	0.178870
C	-4.325916	4.223304	-0.704860
C	-3.245146	4.412496	1.447410
C	-5.228387	5.217913	-0.339690
H	-4.390856	3.808904	-1.703700
C	-4.156098	5.400144	1.814750
H	-2.466056	4.130897	2.144280
C	-5.152818	5.805703	0.925660
H	-5.985578	5.542302	-1.047470
H	-4.078978	5.862285	2.794590
H	-5.857769	6.581212	1.210570
C	-4.602170	-0.549346	-0.336800
O	-4.603498	-1.711906	0.050430
N	-5.119410	-0.167707	-1.546320
C	-5.636819	-1.230608	-2.408280
H	-6.675999	-1.481359	-2.155610
H	-5.597329	-0.890517	-3.447430
H	-5.024818	-2.123467	-2.287340
C	-5.720722	1.142292	-1.754700
H	-5.459523	1.534823	-2.744550
H	-6.816772	1.075481	-1.697360
H	-5.384143	1.841283	-0.989360
Ir	-0.633342	0.665199	0.223590
P	1.636158	1.083722	0.022230
P	-0.594188	-1.649881	-0.086890
O	2.899479	0.128834	0.374870
O	2.146947	1.644593	-1.445980
O	1.956626	2.335372	1.008360
O	-0.134277	-2.604310	1.152410
O	-2.007458	-2.282513	-0.567250
O	0.398412	-2.232640	-1.269540
C	3.184991	-1.210546	0.011580
C	1.890036	2.932082	-1.917560
C	3.133885	3.093684	0.960350
C	0.553352	-2.111589	2.283590
C	-2.195256	-3.554283	-1.129440
C	0.058002	-2.043780	-2.610810
C	2.616862	-2.208757	0.831220
C	4.085411	-1.501285	-1.031070
C	1.121065	3.045651	-3.075520

C	2.476884	4.052293	-1.299530
C	3.971065	3.042035	2.070870
C	3.379354	3.950064	-0.125180
C	-0.093098	-1.993510	3.526120
C	1.928842	-1.838008	2.111450
C	-2.987605	-4.460314	-0.429070
C	-1.705056	-3.834852	-2.415140
C	0.801821	-1.131559	-3.357890
C	-0.940357	-2.839501	-3.197510
C	2.795904	-3.566916	0.503100
C	4.154493	-2.865655	-1.363060
C	0.892814	4.302261	-3.635170
H	0.729417	2.142521	-3.533340
C	2.211562	5.307043	-1.879230
C	5.105504	3.850337	2.114300
H	3.713686	2.378855	2.889810
C	4.536963	4.747426	-0.054940
C	0.697511	-1.438579	4.549040
C	2.686961	-1.336007	3.187790
C	-3.271983	-5.701744	-0.995600
H	-3.393445	-4.169525	0.530600
C	-2.004904	-5.097123	-2.958450
C	0.553451	-0.989709	-4.723410
H	1.588590	-0.570338	-2.869970
C	-1.173187	-2.670712	-4.574250
C	3.518664	-3.886065	-0.661670
H	4.770203	-3.157864	-2.205600
C	1.431932	5.437732	-3.026170
H	0.302734	4.391140	-4.542650
H	2.624511	6.193543	-1.407860
C	5.390003	4.702077	1.044580
H	5.763194	3.811977	2.977340
H	4.773642	5.397736	-0.891520
C	2.037461	-1.089197	4.414110
H	0.243791	-1.295280	5.524320
C	-2.772623	-6.024444	-2.259610
H	-3.886082	-6.411955	-0.449920
H	-1.610624	-5.349792	-3.938300
C	-0.440328	-1.759851	-5.331880
H	1.144780	-0.291139	-5.308690
H	-1.952456	-3.261533	-5.046450
H	1.251791	6.422702	-3.445880
H	6.277552	5.327008	1.063770
H	-2.981801	-6.994454	-2.700530
H	-0.641758	-1.655351	-6.393870
H	-0.328821	0.434979	1.720200
C	2.776840	-0.508296	5.598720
H	2.090080	-0.305217	6.425680
H	3.549851	-1.191405	5.973720
H	3.281049	0.431964	5.345170
C	4.172481	-1.083615	3.060030
H	4.380119	-0.042484	2.779710
H	4.688901	-1.274684	4.004850
H	4.626322	-1.717614	2.295990
C	2.232575	-4.681007	1.354450
H	2.933067	-5.519636	1.418000
H	2.012535	-4.352577	2.371420
H	1.300176	-5.070098	0.924860
C	3.670296	-5.316325	-1.125990
H	4.192086	-5.362225	-2.086390
H	4.242237	-5.921885	-0.410950
H	2.696857	-5.806067	-1.248760
C	-1.504217	-2.484562	3.940540
C	5.052950	-0.529963	-1.767080
C	5.410628	0.718947	-0.932500
H	4.575328	1.404416	-0.817880
H	5.767519	0.442507	0.066020
H	6.218348	1.263528	-1.434490
C	6.401371	-1.253262	-2.037470
H	6.822692	-1.675921	-1.118800
H	6.320382	-2.055552	-2.776300
H	7.119710	-0.529071	-2.436480
C	4.472609	-0.095974	-3.132430
H	3.574319	0.513535	-3.009640
H	5.210879	0.502837	-3.679910
H	4.222511	-0.966105	-3.750380
C	-1.318246	-3.586832	5.017710
H	-0.776396	-3.226021	5.897080
H	-2.297565	-3.947533	5.353480
H	-0.765695	-4.441111	4.610450
C	-2.355167	-3.101203	2.816970
H	-2.639058	-2.377984	2.049420
H	-1.846105	-3.938743	2.334740
H	-3.284246	-3.482024	3.258210
C	-2.311869	-1.312743	4.547840
H	-1.835319	-0.886693	5.436220
H	-2.437060	-0.510303	3.813880
H	-3.307588	-1.663214	4.844130

TS-III-L-6 Conformation 1

B3LYP/6-31G(d),SDD(Ir) Energy = -3686.875626
B3LYP/def2-TZVPP,SDD(Ir)/IEFPCM(dioxane) Energy = -3688.131158
B3LYP/def2-TZVPP,SDD(Ir)/IEFPCM(dioxane)//B3LYP/6-31G(d),SDD(Ir) Free Energy (Quasiharmonic) = -3687.265009
Number of Imaginary Frequencies = 1

Frequencies (Top 3 out of 369)

1. -363.3802 cm^{-1}
2. 6.9441 cm^{-1}
3. 22.2218 cm^{-1}

B3LYP/6-31G(d),SDD(Ir) Molecular Geometry in Cartesian Coordinates

C	-1.465130	-2.266786	-1.119260
C	-2.512435	-1.706607	-1.989520
C	-2.442488	0.222642	-1.414300
C	-3.347955	0.648390	-0.424990
O	-2.697512	0.983674	-2.537040
C	-4.146546	1.682067	-0.971000
C	-3.703414	1.835903	-2.260440
H	-4.003059	2.496926	-3.061180
H	-4.914221	2.273573	-0.494250
H	-1.806924	-2.789813	-0.229810
H	-0.734405	-2.860383	-1.667520
C	-3.162859	0.107348	0.930470
O	-2.025014	-0.379592	1.201040
N	-4.132289	0.144837	1.863820
C	-3.818412	-0.239846	3.239790
H	-4.430397	0.360949	3.918350
H	-4.037612	-1.301304	3.410450
H	-2.764901	-0.050455	3.439380
C	-5.542767	0.399089	1.574710
H	-6.147663	-0.340246	2.109230
H	-5.843318	1.399371	1.909300
H	-5.745238	0.288001	0.510590
Ir	-0.544574	-0.390974	-0.558050
P	1.178286	-1.573319	0.349220
P	0.168175	1.801590	-0.322300
O	2.671971	-1.017272	0.648210
O	0.870940	-2.300436	1.794310
O	1.546155	-2.844292	-0.598780
O	1.413790	2.328169	-1.225090
O	-1.020246	2.853050	-0.733790
O	0.649331	2.438775	1.117130
C	3.123922	0.210814	1.163280
C	0.140630	-3.484350	1.907990
C	2.416026	-3.865050	-0.187260
C	2.319243	1.509351	-1.919170
C	-0.914874	4.239209	-0.559980
C	-0.261626	2.801393	2.106100
C	3.523950	1.200331	0.251350
C	3.291943	0.341723	2.545620
C	-1.084790	-3.425430	2.569200
C	0.687769	-4.700115	1.460700
C	3.629195	-3.982580	-0.858490

C	2.008108	-4.784576	0.791650
C	2.151312	1.374013	-3.299010
C	3.398648	0.952342	-1.221050
C	-0.859077	5.029778	-1.704820
C	-0.974059	4.794559	0.728290
C	-0.275313	2.056833	3.284550
C	-1.048206	3.956930	1.948540
C	4.054960	2.411226	0.741980
C	3.816703	1.557088	2.995290
C	-1.803250	-4.598743	2.796350
C	-0.066221	-5.864368	1.695520
C	4.486346	-5.036188	-0.545580
C	2.898069	-5.831894	1.091780
C	3.112535	0.616054	-3.973550
C	4.360901	0.206403	-1.933770
C	-0.858655	6.418348	-1.582370
H	-0.823921	4.546708	-2.675620
C	-0.968577	6.197969	0.820580
C	-1.096119	2.451420	4.340460
H	0.380550	1.196868	3.364560
C	-1.875283	4.322617	3.025890
C	4.185172	2.591975	2.134230
H	3.957014	1.691337	4.065570
C	-1.292590	-5.821728	2.354340
H	-2.754190	-4.558375	3.320140
C	4.120468	-5.959274	0.437040
H	5.434785	-5.133646	-1.065230
C	4.203640	0.030515	-3.325310
H	3.011254	0.490135	-5.049190
C	-0.913720	7.002699	-0.314840
H	-0.811470	7.038738	-2.472360
H	-0.986323	6.656030	1.805010
C	-1.903329	3.584417	4.207130
H	-1.095924	1.882460	5.265890
H	-2.505396	5.201403	2.924260
H	-0.903881	8.083329	-0.209930
H	-2.546367	3.898153	5.024210
H	0.285196	-0.440371	-1.891240
H	-1.451121	-2.457666	2.892580
H	3.874512	-3.255052	-1.624620
H	4.787261	-6.776380	0.694980
H	2.623733	-6.544442	1.863890
H	0.320241	-6.814452	1.339360
H	-1.847668	-6.740063	2.520340
C	4.736333	3.878590	2.702670
H	4.733823	3.857400	3.796320
H	5.768445	4.059941	2.376390
H	4.147141	4.746855	2.382450
C	4.482710	3.518012	-0.193780
H	3.799437	4.374638	-0.123850
H	5.483043	3.887604	0.059160
H	4.498997	3.193262	-1.235080
C	5.563276	-0.386397	-1.234840
H	5.509717	-1.482316	-1.203360
H	6.491239	-0.129645	-1.758860
H	5.650469	-0.034518	-0.205810
C	5.212263	-0.760564	-4.126130
H	4.908252	-0.838001	-5.173980
H	6.205637	-0.294202	-4.102430
H	5.335064	-1.779395	-3.737760
C	1.005207	2.031642	-4.026610
H	0.033544	1.661961	-3.677750
H	1.010977	3.117412	-3.873910
H	1.072556	1.840322	-5.101600
C	2.973493	-0.766725	3.519560
H	1.895662	-0.894315	3.669480
H	3.352975	-1.730318	3.165830
H	3.426095	-0.553218	4.492320
C	-3.965268	-2.027385	-1.761860
C	-4.951784	-1.537036	-2.642000
C	-4.384885	-2.843751	-0.694450
C	-6.298576	-1.842635	-2.458310
H	-4.671138	-0.908619	-3.479220
C	-5.733227	-3.153200	-0.515630
H	-3.660548	-3.266517	-0.008570
C	-6.697463	-2.652051	-1.392270
H	-7.036343	-1.455338	-3.155280
H	-6.027843	-3.803347	0.303570
H	-7.745965	-2.900262	-1.254990
C	-2.094164	-1.640181	-3.453420
H	-1.044492	-1.356930	-3.543740
H	-2.219453	-2.650610	-3.863600
H	-2.690449	-0.958086	-4.057850

TS-III-L-6 Conformation 2

B3LYP/6-31G(d),SDD(Ir) Energy = -3686.872767
B3LYP/def2-TZVPP,SDD(Ir)/IEFPCM(dioxane) Energy = -3688.128484
B3LYP/def2-TZVPP,SDD(Ir)/IEFPCM(dioxane)//B3LYP/6-31G(d),SDD(Ir) Free Energy (Quasiharmonic) = -3687.261412
Number of Imaginary Frequencies = 1

Frequencies (Top 3 out of 369)

1. -361.1220 cm^{-1}
2. 8.8728 cm^{-1}
3. 20.4280 cm^{-1}

B3LYP/6-31G(d),SDD(Ir) Molecular Geometry in Cartesian Coordinates

C	-1.603445	-2.507129	0.328950
C	-0.717960	-3.503459	0.966679
C	1.057962	-2.606034	0.780352
C	1.914637	-3.037652	-0.250733
O	1.748619	-2.775362	1.961967
C	3.132826	-3.464682	0.336886
C	2.963388	-3.279191	1.686123
H	3.609028	-3.460273	2.533619
H	4.035571	-3.814511	-0.141687
H	-1.933098	-2.809069	-0.666326
H	-2.448481	-2.189394	0.933293
C	-0.515564	-4.767068	0.134063
H	-1.404777	-5.396978	0.269473
H	-0.448450	-4.521578	-0.927280
H	0.363624	-5.348147	0.419507
C	-0.834257	-3.717356	2.449767
C	-0.415889	-4.925397	3.038398
C	-1.398271	-2.739200	3.290263
C	-0.569316	-5.153694	4.405191
H	0.021991	-5.710380	2.432828
C	-1.549046	-2.965611	4.655649
H	-1.717495	-1.788687	2.879883
C	-1.136056	-4.174535	5.221104
H	-0.248805	-6.100809	4.829606
H	-1.994620	-2.195831	5.279211
H	-1.257532	-4.351546	6.285890
C	1.483770	-2.760710	-1.632901
O	0.583943	-1.882497	-1.785235
N	2.019495	-3.381376	-2.701612
C	1.665776	-2.928997	-4.048011
H	2.541016	-3.038337	-4.694437
H	0.843569	-3.528097	-4.458713
H	1.371169	-1.881663	-4.013857
C	2.867167	-4.569636	-2.629676
H	2.540336	-5.280522	-3.395184
H	3.918799	-4.320374	-2.816501
H	2.773931	-5.053437	-1.658462
Ir	-0.162708	-0.905204	0.167188
P	-1.834316	0.434611	-0.599052
P	1.614354	0.575397	0.359431
O	-1.872223	2.054453	-0.523417
O	-2.257122	0.225076	-2.175343
O	-3.226610	0.101480	0.175681
O	1.577411	1.690532	1.542368
O	3.015381	-0.214258	0.676142
O	2.096371	1.565655	-0.864536

C	-0.882851	3.024785	-0.761986
C	-3.055695	-0.835270	-2.610229
C	-4.461647	0.621276	-0.240891
C	0.436486	2.062652	2.271682
C	4.263912	0.420168	0.719446
C	2.838620	1.103622	-1.947765
C	-0.178072	3.532104	0.341185
C	-0.744797	3.544383	-2.053278
C	-2.472634	-1.778332	-3.454064
C	-4.422654	-0.871753	-2.282782
C	-5.092389	1.530006	0.603232
C	-5.064646	0.144926	-1.415516
C	0.297099	1.556870	3.566056
C	-0.448581	2.994722	1.713776
C	4.907746	0.491082	1.952051
C	4.871117	0.863727	-0.466348
C	2.226678	1.096721	-3.200436
C	4.196852	0.775952	-1.783187
C	0.765587	4.560549	0.138489
C	0.202832	4.559295	-2.216586
C	-3.256039	-2.797009	-3.994800
C	-5.183627	-1.917262	-2.836503
C	-6.361459	2.003676	0.275009
C	-6.341662	0.647685	-1.724419
C	-0.808270	2.004797	4.295005
C	-1.547024	3.436711	2.480932
C	6.195235	1.020164	2.026159
H	4.394410	0.124521	2.834745
C	6.168621	1.396232	-0.359677
C	2.967199	0.746037	-4.328675
H	1.186844	1.396043	-3.272977
C	4.914121	0.413149	-2.937598
C	0.966120	5.064535	-1.162946
H	0.336185	4.982363	-3.209796
C	-4.615618	-2.867172	-3.682044
H	-2.807157	-3.531024	-4.657898
C	-6.984044	1.564106	-0.895725
H	-6.859199	2.713269	0.929091
C	-1.729377	2.922775	3.782804
H	-0.945648	1.632378	5.307790
C	6.826228	1.473889	0.865532
H	6.699541	1.080792	2.985937
H	6.653097	1.772889	-1.255562
C	4.313694	0.396522	-4.194711
H	2.496600	0.756357	-5.307720
H	5.960315	0.139488	-2.836453
H	7.824990	1.896778	0.915605
H	4.897837	0.121414	-5.068020
H	-0.609055	-0.432237	1.594186
H	-1.413073	-1.695671	-3.668742
H	-4.584601	1.840288	1.510096
H	-7.967823	1.936468	-1.164695
H	-6.823947	0.319074	-2.640122
H	-6.236877	-1.985088	-2.581619
H	-5.232183	-3.661214	-4.092308
C	1.976578	6.156221	-1.426820
H	2.025236	6.396568	-2.492835
H	1.728870	7.080461	-0.889163
H	2.982734	5.862794	-1.102652
C	1.567211	5.129384	1.286355
H	2.621094	4.830193	1.212771
H	1.546431	6.225127	1.280878
H	1.195724	4.792202	2.255022
C	-2.514018	4.465611	1.940722
H	-3.505512	4.029861	1.762026
H	-2.653718	5.288123	2.651921
H	-2.170169	4.892415	0.997457
C	-2.891210	3.372701	4.638444
H	-2.904257	2.841193	5.594407
H	-2.842528	4.447184	4.857265
H	-3.855335	3.196860	4.145002
C	1.299381	0.595036	4.153997
H	1.324192	-0.354690	3.606608
H	2.312118	1.013881	4.125047
H	1.053143	0.373996	5.196657
C	-1.589109	3.085977	-3.217676
H	-1.283381	2.103759	-3.594574
H	-2.642584	2.995146	-2.935699
H	-1.512495	3.801828	-4.041314

TS-III-L-6 Conformation 3

B3LYP/6-31G(d),SDD(Ir) Energy = -3686.82124
B3LYP/def2-TZVPP,SDD(Ir)/IEFPCM(dioxane) Energy = -3688.079548
B3LYP/def2-TZVPP,SDD(Ir)/IEFPCM(dioxane)//B3LYP/6-31G(d),SDD(Ir) Free Energy (Quasiharmonic) = -3687.216306
Number of Imaginary Frequencies = 1

Frequencies (Top 3 out of 369)

1. -281.2765 cm^{-1}
2. 14.2354 cm^{-1}
3. 16.8058 cm^{-1}

B3LYP/6-31G(d),SDD(Ir) Molecular Geometry in Cartesian Coordinates

C	0.465606	-2.664421	-1.560380
C	-0.877135	-3.254758	-1.703390
C	-1.685672	-1.331937	-2.219690
C	-3.070531	-1.187535	-2.496140
O	-1.061522	-1.316028	-3.497160
C	-3.240401	-1.107784	-3.901450
C	-1.988281	-1.192667	-4.448190
H	-1.632101	-1.172487	-5.469200
H	-4.172531	-0.963393	-4.429550
H	1.056555	-3.163302	-0.788750
H	1.018986	-2.594642	-2.496070
C	-4.230301	-0.763323	-1.625630
O	-4.536539	0.425618	-1.703770
N	-4.906792	-1.660192	-0.860000
C	-6.026661	-1.175010	-0.057250
H	-6.980102	-1.318558	-0.583180
H	-6.058112	-1.731170	0.885040
H	-5.890140	-0.113430	0.142300
C	-4.856925	-3.098272	-1.066270
H	-4.708265	-3.617822	-0.115140
H	-5.796295	-3.451050	-1.514850
H	-4.036135	-3.351983	-1.735670
Ir	-0.144211	-0.732030	-0.809960
P	1.981410	-0.452743	0.036350
P	-1.103947	1.168362	0.142360
O	2.734342	0.978346	0.118490
O	2.354249	-1.098904	1.506720
O	3.017889	-1.218905	-0.953850
O	-0.894735	2.620002	-0.559640
O	-2.706068	1.058795	0.366210
O	-0.611277	1.559501	1.668470
C	2.445554	2.161796	0.830220
C	2.661597	-2.452484	1.690350
C	4.385668	-1.325047	-0.652380
C	0.027315	2.801060	-1.608370
C	-3.444546	2.027726	1.068800
C	-1.127878	0.928312	2.800420
C	1.770806	3.190427	0.152950
C	2.963324	2.314425	2.120100
C	1.800245	-3.200843	2.490510
C	3.851466	-2.993536	1.169480
C	5.281120	-0.615059	-1.445520
C	4.807287	-2.201278	0.358850
C	-0.463385	2.809451	-2.916150
C	1.371106	3.039998	-1.285680
C	-4.319145	2.832527	0.344940
C	-3.349086	2.085216	2.466860
C	-0.264889	0.125151	3.546040
C	-2.442977	1.192864	3.224750
C	1.511858	4.399958	0.832810
C	2.680797	3.524006	2.762410

C	2.104983	-4.530153	2.780650
H	0.909536	-2.727301	2.888720
C	4.125843	-4.339067	1.473860
C	6.650359	-0.766441	-1.232350
H	4.894571	0.035822	-2.222440
C	6.194377	-2.327960	0.555000
C	0.479886	3.004459	-3.930100
C	2.294436	3.238586	-2.334360
C	-5.124603	3.742009	1.029870
H	-4.376145	2.709337	-0.729340
C	-4.172554	3.013337	3.128140
C	-0.708450	-0.447079	4.739110
H	0.749831	-0.021141	3.194250
C	-2.863538	0.596265	4.426720
C	1.953098	4.552007	2.163920
H	3.062117	3.669865	3.770620
C	3.270532	-5.101205	2.266280
H	1.434542	-5.113022	3.405020
H	5.024813	-4.790028	1.064800
C	7.105998	-1.622712	-0.226920
H	7.356230	-0.215472	-1.846400
H	6.553006	-2.979551	1.346140
C	1.838866	3.200757	-3.670550
H	0.135356	3.015560	-4.961770
C	-5.048543	3.834169	2.421970
H	-5.810102	4.375710	0.475100
H	-4.102394	3.099167	4.208470
C	-2.014240	-0.213716	5.177930
H	-0.031381	-1.060660	5.326670
H	-3.880638	0.770917	4.764780
H	3.513790	-6.137536	2.479940
H	8.170738	-1.737734	-0.048430
H	-5.667782	4.547760	2.957170
H	-2.368690	-0.654936	6.104750
H	0.397041	0.146719	-1.966070
C	2.797426	3.405555	-4.820690
H	3.610605	2.669034	-4.809210
H	2.282296	3.319206	-5.781710
H	3.269678	4.395615	-4.784950
C	3.754456	3.513714	-2.056040
H	4.390455	2.703543	-2.436310
H	4.086128	4.432433	-2.554730
H	3.956277	3.620244	-0.989650
C	0.772080	5.538089	0.167120
H	1.272571	6.495528	0.348360
H	0.689230	5.403819	-0.912080
H	-0.246150	5.629481	0.568140
C	1.678400	5.821237	2.936380
H	2.049620	5.743027	3.962300
H	2.159792	6.693157	2.475130
H	0.604911	6.041389	2.983930
C	-1.935665	2.674163	-3.216450
H	-2.384676	1.775464	-2.782180
H	-2.493403	3.527324	-2.810500
H	-2.104035	2.649864	-4.297210
C	3.824953	1.278404	2.801800
H	3.229701	0.508695	3.304710
H	4.476712	0.764043	2.090520
H	4.452594	1.758313	3.558760
C	-1.181616	-4.069298	-2.937280
H	-0.702328	-5.049939	-2.809230
H	-2.251006	-4.232316	-3.098010
H	-0.744805	-3.620869	-3.828350
C	-1.522136	-3.691857	-0.429680
C	-1.506464	-2.879547	0.716760
C	-2.079298	-4.977676	-0.302400
C	-2.035635	-3.302426	1.931840
H	-1.109082	-1.851028	0.698860
C	-2.586318	-5.424356	0.918800
H	-2.103509	-5.648156	-1.153890
C	-2.575457	-4.588596	2.036070
H	-2.023974	-2.634756	2.788130
H	-2.995500	-6.427715	0.992000
H	-2.981968	-4.932835	2.982520

TS-III-L-6 Conformation 4

B3LYP/6-31G(d),SDD(Ir) Energy = -3686.819091
B3LYP/def2-TZVPP,SDD(Ir)/IEFPCM(dioxane) Energy = -3688.079559
B3LYP/def2-TZVPP,SDD(Ir)/IEFPCM(dioxane)//B3LYP/6-31G(d),SDD(Ir) Free Energy (Quasiharmonic) = -3687.216074
Number of Imaginary Frequencies = 1

Frequencies (Top 3 out of 369)

1. -278.2218 cm^{-1}
2. 10.2573 cm^{-1}
3. 18.4411 cm^{-1}

B3LYP/6-31G(d),SDD(Ir) Molecular Geometry in Cartesian Coordinates

C	-1.964028	2.168803	0.243568
C	-3.212506	1.503487	-0.189780
C	-2.706709	-0.187626	0.993498
C	-3.620511	-1.275196	1.047422
O	-2.889882	0.491382	2.225809
C	-4.337596	-1.199654	2.263614
C	-3.852185	-0.101931	2.927779
H	-4.091816	0.335035	3.887297
H	-5.091573	-1.889666	2.616325
H	-1.602437	2.845557	-0.537888
H	-2.005365	2.694598	1.193734
C	-3.098788	0.962134	-1.608490
H	-3.287153	1.777429	-2.319847
H	-2.077752	0.604112	-1.821485
H	-3.777781	0.139409	-1.828239
C	-4.549085	1.944646	0.270455
C	-5.697058	1.737109	-0.526578
C	-4.710150	2.668449	1.472670
C	-6.941102	2.228637	-0.142038
H	-5.616669	1.221297	-1.475985
C	-5.957941	3.150805	1.860217
H	-3.850769	2.869090	2.099751
C	-7.078842	2.931802	1.057485
H	-7.802671	2.071593	-0.784318
H	-6.052130	3.709617	2.786725
H	-8.049795	3.315009	1.357342
C	-3.670928	-2.555598	0.249808
O	-3.046625	-3.502537	0.719878
N	-4.405881	-2.642062	-0.897036
C	-4.400639	-3.918918	-1.609736
H	-5.206129	-4.575794	-1.254802
H	-4.544993	-3.729090	-2.677707
H	-3.445589	-4.416476	-1.447769
C	-5.539154	-1.773163	-1.176490
H	-5.519716	-1.435174	-2.219600
H	-6.483745	-2.311334	-1.014441
H	-5.524785	-0.906496	-0.515668
Ir	-0.753377	0.395173	0.239317
P	1.101667	1.699404	-0.126981
P	0.320462	-1.674353	-0.024108
O	2.546583	1.419171	0.546333
O	1.527173	2.039735	-1.685675
O	0.827903	3.181789	0.482368
O	1.093992	-2.309891	1.262411
O	-0.629951	-2.876624	-0.553509
O	1.556582	-1.763688	-1.112693
C	3.458931	0.352357	0.392298
C	0.786054	2.947483	-2.451087
C	1.675700	4.269239	0.220181
C	1.321999	-1.544786	2.421098
C	-0.134119	-4.162628	-0.836525
C	1.371975	-2.005026	-2.474987
C	3.415936	-0.700263	1.320669
C	4.448753	0.460972	-0.589013
C	0.064757	2.450941	-3.535761
C	0.860795	4.324380	-2.171644

C	2.418285	4.783276	1.278240
C	1.672230	4.861315	-1.052685
C	0.458647	-1.727547	3.504548
C	2.438725	-0.695429	2.459987
C	-0.484117	-5.200044	0.022864
C	0.609936	-4.371110	-2.006378
C	1.747474	-0.995447	-3.361135
C	0.943119	-3.264645	-2.931546
C	4.350264	-1.751937	1.201000
C	5.351837	-0.602551	-0.679116
C	-0.617782	3.335789	-4.371445
H	0.072445	1.382908	-3.729583
C	0.155658	5.188660	-3.027545
C	3.191571	5.926051	1.077982
H	2.372895	4.289007	2.242816
C	2.463745	6.010514	-1.225032
C	0.717763	-0.956607	4.642101
C	2.671309	0.065194	3.626211
C	-0.072721	-6.497578	-0.279974
H	-1.093612	-4.978326	0.889718
C	1.012360	-5.690121	-2.282250
C	1.681275	-1.218496	-4.736339
H	2.109701	-0.057018	-2.958401
C	0.877455	-3.455517	-4.323667
C	5.311020	-1.708745	0.169138
H	6.128556	-0.552701	-1.438966
C	-0.575455	4.707417	-4.111981
H	-1.172402	2.953768	-5.223534
H	0.178438	6.254920	-2.823495
C	3.213630	6.539311	-0.176948
H	3.774799	6.332901	1.898481
H	2.500152	6.478134	-2.204455
C	1.785648	-0.058743	4.719150
H	0.064815	-1.071463	5.504488
C	0.678791	-6.741589	-1.432237
H	-0.339583	-7.314320	0.384147
H	1.612489	-5.879612	-3.167208
C	1.238154	-2.451670	-5.218897
H	1.982784	-0.433846	-5.424337
H	0.527012	-4.412127	-4.699615
H	-1.107933	5.402609	-4.753915
H	3.819881	7.424790	-0.341616
H	1.009251	-7.749142	-1.666392
H	1.177243	-2.633335	-6.287727
H	-0.287220	0.404146	1.711523
C	1.999337	0.741572	5.983099
H	2.009722	1.820665	5.784956
H	1.208203	0.543220	6.711966
H	2.957909	0.500366	6.460090
C	3.859423	0.993109	3.737944
H	3.537672	2.040127	3.815197
H	4.446130	0.778313	4.639157
H	4.525589	0.913267	2.878286
C	4.345287	-2.925081	2.154759
H	5.360139	-3.171848	2.484711
H	3.742689	-2.734004	3.043589
H	3.940404	-3.822309	1.667885
C	6.314911	-2.823966	-0.009345
H	6.943940	-2.647184	-0.886563
H	6.978045	-2.920688	0.859954
H	5.821790	-3.794865	-0.140422
C	-0.660081	-2.738927	3.468927
H	-1.340417	-2.598072	2.623170
H	-0.259728	-3.756478	3.379828
H	-1.247439	-2.690927	4.390807
C	4.598758	1.663937	-1.489537
H	3.945994	1.609264	-2.367231
H	4.352997	2.592014	-0.966012
H	5.630516	1.733664	-1.847047

TS-III-L-7 Conformation 1

B3LYP/6-31G(d),SDD(Ir) Energy = -3608.24455

B3LYP/def2-TZVPP,SDD(Ir)/IEFPCM(dioxane) Energy = -3609.472172
B3LYP/def2-TZVPP,SDD(Ir)/IEFPCM(dioxane)//B3LYP/6-31G(d),SDD(Ir) Free Energy (Quasiharmonic) = -3608.656898
Number of Imaginary Frequencies = 1

Frequencies (Top 3 out of 351)

1. -361.3735 cm^{-1}
2. 8.0018 cm^{-1}
3. 19.0796 cm^{-1}

B3LYP/6-31G(d),SDD(Ir) Molecular Geometry in Cartesian Coordinates

C	-1.613189	-2.117630	-1.317079
C	-2.573348	-1.443878	-2.208170
C	-2.374732	0.450335	-1.554880
C	-3.281479	0.903491	-0.580268
O	-2.525578	1.277049	-2.650291
C	-3.977718	2.018382	-1.106123
C	-3.475094	2.191907	-2.370799
H	-3.692992	2.907073	-3.151262
H	-4.715291	2.647132	-0.629316
H	-2.030436	-2.628095	-0.451666
H	-0.916903	-2.761734	-1.852533
C	-3.187917	0.295836	0.756311
O	-2.100372	-0.283819	1.048682
N	-4.184143	0.367845	1.658029
C	-3.933896	-0.103024	3.021456
H	-4.642152	0.389287	3.692196
H	-4.064462	-1.189564	3.094729
H	-2.917082	0.155709	3.317317
C	-5.566649	0.714242	1.330757
H	-6.231992	-0.026984	1.784911
H	-5.831025	1.704086	1.721725
H	-5.726065	0.687591	0.254030
Ir	-0.563158	-0.349594	-0.662509
P	1.089205	-1.633301	0.219529
P	0.304199	1.756924	-0.232046
O	2.650433	-1.213287	0.074047
O	1.030111	-2.016596	1.820758
O	1.149094	-3.093390	-0.490657
O	1.530803	2.336231	-1.130201
O	-0.836470	2.920353	-0.400037
O	0.928408	2.144629	1.239470
C	3.319784	-0.138282	0.680279
C	0.234912	-3.061104	2.306671
C	2.008631	-4.105279	-0.032276
C	2.287911	1.595613	-2.047098
C	-0.630533	4.255245	-0.024630
C	0.111200	2.392016	2.340494
C	3.754604	0.913584	-0.138198
C	3.614875	-0.193479	2.036670
C	-0.822871	-2.726996	3.148816
C	0.575792	-4.397301	2.030434
C	3.066126	-4.475018	-0.856988
C	1.727376	-4.766702	1.172850
C	1.933516	1.646977	-3.388541
C	3.420023	0.900581	-1.599167
C	-0.591005	5.209546	-1.037376
C	-0.573525	4.604663	1.334297
C	0.136138	1.475643	3.390485
C	-0.617173	3.592803	2.416734
C	4.490788	1.965282	0.448114
C	4.339681	0.853715	2.599824
H	3.291367	-1.042277	2.627739
C	-1.577677	-3.738605	3.740782
C	-0.211807	-5.394170	2.634031
C	3.886167	-5.537618	-0.481076
C	2.575705	-5.830871	1.527935
C	2.723959	0.968923	-4.313970
H	1.061451	2.215383	-3.695299
C	4.211462	0.218165	-2.547463
C	-0.493972	6.559996	-0.705751

H	-0.642810	4.882622	-2.070678
C	-0.473760	5.974261	1.637768
C	-0.589233	1.740452	4.551976
H	0.741706	0.581574	3.288209
C	-1.348410	3.825766	3.595413
C	4.778920	1.935159	1.830880
H	4.576432	0.824447	3.660224
C	-1.272318	-5.076220	3.479127
H	-2.397652	-3.483099	4.405985
C	3.640094	-6.214541	0.715891
H	4.713883	-5.832480	-1.118984
C	3.855971	0.251467	-3.915548
H	2.458820	1.003552	-5.367418
C	-0.436864	6.941831	0.636587
H	-0.459301	7.307842	-1.492294
H	-0.401353	6.273657	2.679083
C	-1.338620	2.916269	4.650952
H	-0.559147	1.036733	5.379089
H	-1.933184	4.737571	3.674693
H	-0.352292	7.990863	0.903537
H	-1.906888	3.129294	5.551640
H	0.299112	-0.391941	-1.975140
H	-1.032419	-1.678575	3.327125
H	3.225040	-3.933211	-1.783314
H	4.280315	-7.037031	1.019956
H	2.399848	-6.347834	2.466517
H	0.013563	-6.434630	2.420504
H	-1.858798	-5.870841	3.930462
C	5.559588	3.052265	2.482555
H	5.670552	2.878030	3.556692
H	6.566042	3.149676	2.055857
H	5.065691	4.022994	2.349983
C	4.972975	3.133403	-0.380681
H	4.479897	4.063459	-0.069760
H	6.051098	3.290226	-0.256050
H	4.774771	2.992602	-1.444132
C	5.443001	-0.548166	-2.122200
H	5.327814	-1.621771	-2.320123
H	6.327544	-0.218815	-2.680699
H	5.652870	-0.427988	-1.058491
C	4.691483	-0.467568	-4.948931
H	4.258696	-0.359494	-5.947651
H	5.716342	-0.076778	-4.986885
H	4.772066	-1.540412	-4.733564
C	-4.056583	-1.652064	-2.056516
C	-4.588505	-2.474237	-1.045533
C	-4.959439	-1.046840	-2.954694
C	-5.963801	-2.680476	-0.937333
H	-3.930780	-2.981984	-0.350515
C	-6.333267	-1.248949	-2.841640
H	-4.591380	-0.409423	-3.750229
C	-6.843710	-2.066510	-1.830750
H	-6.345941	-3.337732	-0.161123
H	-7.005337	-0.774234	-3.550890
H	-7.913839	-2.233778	-1.749224
C	-2.086384	-1.359479	-3.649881
H	-1.013883	-1.163366	-3.686164
H	-2.279330	-2.338639	-4.106916
H	-2.595756	-0.605454	-4.248506

TS-III-L-7 Conformation 2

B3LYP/6-31G(d),SDD(Ir) Energy = -3608.24165
B3LYP/def2-TZVPP,SDD(Ir)/IEFPCM(dioxane) Energy = -3609.469681
B3LYP/def2-TZVPP,SDD(Ir)/IEFPCM(dioxane)//B3LYP/6-31G(d),SDD(Ir) Free Energy (Quasiharmonic) = -3608.654061
Number of Imaginary Frequencies = 1

Frequencies (Top 3 out of 351)

1. -358.1860 cm^{-1}
2. 8.0990 cm^{-1}
3. 17.3624 cm^{-1}

B3LYP/6-31G(d),SDD(Ir) Molecular Geometry in Cartesian Coordinates

C	-1.286180	-2.725311	0.418344
C	-0.293087	-3.585863	1.095111
C	1.348880	-2.456086	0.959387
C	2.297535	-2.786945	-0.026027
O	2.009033	-2.493358	2.170008
C	3.539997	-3.021139	0.616888
C	3.292881	-2.827473	1.952745
H	3.923270	-2.894216	2.828076
H	4.501228	-3.250151	0.180883
H	-1.547203	-3.079995	-0.580539
H	-2.182123	-2.512993	0.995044
C	0.103072	-4.816754	0.283795
H	-0.699324	-5.557928	0.396029
H	0.172815	-4.571477	-0.777448
H	1.041105	-5.273976	0.604946
C	-0.425550	-3.800174	2.576735
C	0.103217	-4.951997	3.188337
C	-1.110521	-2.879953	3.392230
C	-0.061091	-5.184052	4.553460
H	0.635884	-5.691833	2.602263
C	-1.271226	-3.109270	4.755773
H	-1.513860	-1.970556	2.963277
C	-0.748348	-4.263484	5.344349
H	0.346117	-6.088666	4.995890
H	-1.809178	-2.384448	5.360077
H	-0.877436	-4.443343	6.407767
C	1.885938	-2.607119	-1.429798
O	0.877850	-1.870267	-1.641838
N	2.545148	-3.168364	-2.460955
C	2.169885	-2.798937	-3.827545
H	3.040239	-2.935292	-4.474135
H	1.348762	-3.427087	-4.194026
H	1.862615	-1.754013	-3.853032
C	3.532336	-4.238149	-2.327967
H	3.312909	-5.014506	-3.067845
H	4.548371	-3.866393	-2.507385
H	3.479978	-4.691174	-1.339095
Ir	-0.069381	-0.953566	0.254296
P	-1.849495	0.196035	-0.557304
P	1.478044	0.771990	0.373429
O	-2.163007	1.716201	-0.081530
O	-2.010364	0.352004	-2.188981
O	-3.253755	-0.498941	-0.128481
O	1.374392	1.871925	1.567462
O	3.008793	0.215710	0.544674
O	1.671749	1.833794	-0.868944
C	-1.431896	2.883056	-0.353822
C	-2.545162	-0.673764	-2.978789
C	-4.486766	-0.046009	-0.626392
C	0.290568	2.034336	2.439892
C	4.135631	1.047731	0.480776
C	2.367929	1.498403	-2.027649
C	-0.762050	3.504532	0.709473
C	-1.473995	3.435039	-1.627871
C	-1.697786	-1.291741	-3.894834
C	-3.916065	-0.978232	-2.905929
C	-5.360856	0.559193	0.270688
C	-4.834907	-0.297894	-1.962236
C	0.353847	1.429496	3.688011
C	-0.774793	2.865477	2.065174
C	4.869873	1.227137	1.649767
C	4.545597	1.585619	-0.749849
C	1.644618	1.392915	-3.214813
C	3.770344	1.392509	-1.998551
C	-0.082706	4.716723	0.463352
C	-0.796086	4.630123	-1.854385
H	-2.033276	2.941625	-2.414241
C	-2.211463	-2.245541	-4.772334
C	-4.401434	-1.951241	-3.797920
C	-6.630802	0.936924	-0.162294
C	-6.120711	0.099298	-2.370805

C	-0.686431	1.647933	4.588670
H	1.205998	0.808954	3.945227
C	-1.822008	3.076846	2.987084
C	6.053998	1.961586	1.611471
H	4.507497	0.784664	2.571788
C	5.742698	2.323864	-0.756600
C	2.319282	1.166627	-4.414347
H	0.567209	1.513280	-3.181256
C	4.420948	1.151100	-3.222091
C	-0.096676	5.278841	-0.832717
H	-0.819108	5.072696	-2.846830
C	-3.567189	-2.577349	-4.720991
H	-1.556371	-2.725294	-5.493905
C	-7.009113	0.707345	-1.487288
H	-7.319093	1.409970	0.531587
C	-1.776006	2.459400	4.258283
H	-0.646111	1.183434	5.570481
C	6.490819	2.509946	0.403373
H	6.628560	2.107222	2.521295
H	6.072069	2.774766	-1.687942
C	3.711305	1.039619	-4.415930
H	1.760339	1.100791	-5.343569
H	5.502242	1.048930	-3.226239
H	7.407201	3.091112	0.366387
H	4.244110	0.862755	-5.345658
H	-0.601612	-0.508600	1.660904
H	-0.650695	-1.011691	-3.904370
H	-5.038463	0.716197	1.294552
H	-7.993297	1.007114	-1.834200
H	-6.412326	-0.059880	-3.404608
H	-5.451399	-2.223508	-3.749160
H	-3.974466	-3.324122	-5.395869
C	0.628292	6.572140	-1.122373
H	0.530788	6.848583	-2.176174
H	0.235384	7.403302	-0.522947
H	1.698696	6.496931	-0.893456
C	0.669625	5.424488	1.566566
H	1.747334	5.437986	1.358831
H	0.351894	6.470218	1.656042
H	0.524890	4.945429	2.535924
C	-2.995831	3.962940	2.637992
H	-3.930970	3.388518	2.613837
H	-3.132016	4.753428	3.385971
H	-2.874023	4.438950	1.664097
C	-2.882600	2.677704	5.263745
H	-2.698328	2.108982	6.179813
H	-2.975806	3.734998	5.542793
H	-3.859176	2.370025	4.869260

TS-III-L-7 Conformation 3

B3LYP/6-31G(d),SDD(Ir) Energy = -3608.188051
B3LYP/def2-TZVPP,SDD(Ir)/IEFPCM(dioxane) Energy = -3609.420791
B3LYP/def2-TZVPP,SDD(Ir)/IEFPCM(dioxane)//B3LYP/6-31G(d),SDD(Ir) Free Energy (Quasiharmonic) = -3608.608325
Number of Imaginary Frequencies = 1

Frequencies (Top 3 out of 351)

1. -275.7520 cm^{-1}
2. 9.9615 cm^{-1}
3. 10.7785 cm^{-1}

B3LYP/6-31G(d),SDD(Ir) Molecular Geometry in Cartesian Coordinates

C	-2.093909	2.071403	0.118069
C	-3.290220	1.277348	-0.238401
C	-2.651486	-0.256301	1.092424
C	-3.451956	-1.409305	1.303506
O	-2.860967	0.539188	2.249319
C	-4.133953	-1.264833	2.533768
C	-3.737715	-0.058415	3.052321
H	-3.989059	0.460584	3.966951
H	-4.803756	-1.979200	2.992083
H	-1.781330	2.698977	-0.723644
H	-2.163216	2.670782	1.021299
C	-3.143872	0.620653	-1.605249
H	-3.416102	1.346383	-2.383515
H	-2.096918	0.336024	-1.809503
H	-3.747812	-0.276688	-1.735157
C	-4.655646	1.645371	0.200439
C	-5.789456	1.250957	-0.544428
C	-4.866765	2.476236	1.322807
C	-7.068332	1.667237	-0.186223
H	-5.674670	0.643404	-1.434291
C	-6.148308	2.884229	1.684366
H	-4.021397	2.818670	1.906031
C	-7.254544	2.480714	0.934407
H	-7.920074	1.364526	-0.788186
H	-6.281546	3.528815	2.548251
H	-8.252588	2.805538	1.213498
C	-3.392757	-2.772256	0.660395
O	-2.661536	-3.588700	1.214985
N	-4.149792	-3.062795	-0.435344
C	-4.056554	-4.407371	-1.000512
H	-4.829186	-5.066481	-0.582371
H	-4.193729	-4.348457	-2.084792
H	-3.076603	-4.824729	-0.775072
C	-5.331330	-2.302956	-0.811539
H	-5.326228	-2.086397	-1.886929
H	-6.242173	-2.875774	-0.587964
H	-5.373535	-1.366873	-0.254674
Ir	-0.769914	0.380768	0.224596
P	1.016796	1.760154	-0.179501
P	0.412470	-1.631677	0.011224
O	2.415114	1.518697	0.602647
O	1.546102	2.076425	-1.710981
O	0.665780	3.247401	0.372919
O	1.252031	-2.193967	1.287824
O	-0.479524	-2.896611	-0.474142
O	1.633539	-1.681203	-1.096151
C	3.391849	0.527157	0.395415
C	0.819436	2.944984	-2.538811
C	1.510317	4.346380	0.142794
C	1.464979	-1.427501	2.442797
C	0.090441	-4.159013	-0.729551
C	1.454288	-1.969824	-2.449493
C	3.519753	-0.500826	1.340533
C	4.253567	0.655905	-0.685438
C	0.154491	2.403588	-3.637559
C	0.852779	4.329554	-2.293752
C	2.169067	4.898565	1.236084
C	1.583214	4.908400	-1.141185
C	0.587226	-1.586555	3.507651
C	2.582503	-0.580410	2.509627
C	-0.167108	-5.187026	0.173007
C	0.816454	-4.356980	-1.912048
C	1.791282	-0.972743	-3.364869
C	1.075849	-3.258249	-2.869346
C	4.545250	-1.454770	1.157951
C	5.256608	-0.294808	-0.851291
H	4.138687	1.485994	-1.373048
C	-0.502838	3.252339	-4.529295
H	0.183119	1.330697	-3.797732
C	0.177944	5.156988	-3.208008
C	2.933561	6.051241	1.060432
H	2.067044	4.425904	2.207133
C	2.363011	6.068738	-1.288092
C	0.823188	-0.866191	4.676976
H	-0.252422	-2.268148	3.414129
C	2.805577	0.143795	3.700749
C	0.314112	-6.466422	-0.104668
H	-0.759725	-4.973548	1.053611
C	1.289382	-5.657687	-2.162004
C	1.724518	-1.235199	-4.733051
H	2.130005	-0.014769	-2.988579
C	1.005630	-3.487936	-4.255425

C	5.413983	-1.352537	0.048474
H	5.935138	-0.208585	-1.695834
C	-0.491512	4.631645	-4.311594
H	-1.014480	2.835734	-5.391948
H	0.171787	6.228645	-3.033069
C	3.030052	6.635836	-0.204639
H	3.452247	6.488405	1.908230
H	2.456482	6.514610	-2.273895
C	1.914672	0.000497	4.789941
H	0.148467	-0.985338	5.520680
C	1.043225	-6.701037	-1.272999
H	0.119888	-7.275795	0.592859
H	1.877165	-5.837444	-3.057188
C	1.320287	-2.495067	-5.179676
H	1.995754	-0.459831	-5.443855
H	0.691598	-4.467645	-4.603040
H	-1.003590	5.298301	-4.998797
H	3.630017	7.529106	-0.349060
H	1.427031	-7.693815	-1.488184
H	1.257411	-2.706831	-6.242835
H	-0.275051	0.468012	1.685413
C	2.139824	0.760349	6.076378
H	2.155968	1.845072	5.911865
H	1.350896	0.544553	6.802630
H	3.099187	0.498533	6.540446
C	3.994839	1.066760	3.837902
H	3.673541	2.103798	3.999155
H	4.611208	0.791785	4.702662
H	4.631816	1.048829	2.952711
C	4.727674	-2.597507	2.130471
H	5.765044	-2.658905	2.479783
H	4.083940	-2.501871	3.005801
H	4.497096	-3.558057	1.651796
C	6.511523	-2.367152	-0.171118
H	7.064934	-2.152459	-1.089802
H	7.231992	-2.374870	0.656779
H	6.111804	-3.385676	-0.251062

TS-III-L-7 Conformation 4

B3LYP/6-31G(d),SDD(Ir) Energy = -3608.190707
B3LYP/def2-TZVPP,SDD(Ir)/IEFPCM(dioxane) Energy = -3609.420878
B3LYP/def2-TZVPP,SDD(Ir)/IEFPCM(dioxane)//B3LYP/6-31G(d),SDD(Ir) Free Energy (Quasiharmonic) = -3608.607704
Number of Imaginary Frequencies = 1

Frequencies (Top 3 out of 351)

1. -286.6588 cm^{-1}
2. 12.4940 cm^{-1}
3. 16.0997 cm^{-1}

B3LYP/6-31G(d),SDD(Ir) Molecular Geometry in Cartesian Coordinates

C	0.476490	-2.797799	-1.405869
C	-0.897479	-3.306980	-1.582579
C	-1.534800	-1.392260	-2.287129
C	-2.859430	-1.121750	-2.707489
O	-0.792550	-1.504530	-3.496759
C	-2.886840	-1.090150	-4.124479
C	-1.604060	-1.331040	-4.539129
H	-1.152770	-1.401250	-5.519539
H	-3.742590	-0.876920	-4.750039
H	1.005521	-3.278749	-0.579459
H	1.073630	-2.812319	-2.315099
C	-4.029830	-0.535050	-1.959849
O	-4.109710	0.692710	-1.987309
N	-4.954280	-1.338440	-1.370579
C	-6.098150	-0.717651	-0.707479
H	-6.972480	-0.693721	-1.371689
H	-6.351740	-1.296551	0.186171
H	-5.839220	0.301039	-0.424449
C	-5.083400	-2.759611	-1.650519
H	-5.201049	-3.320941	-0.718979
H	-5.960420	-2.947681	-2.285769
H	-4.194779	-3.122020	-2.166779
Ir	-0.098880	-0.812779	-0.761349
P	2.015900	-0.469659	0.066241
P	-1.074600	1.093000	0.172101
O	2.678070	1.001691	-0.079659
O	2.479300	-0.889629	1.594511
O	3.057860	-1.313179	-0.851229
O	-0.898321	2.539730	-0.544789
O	-2.666810	0.964720	0.453821
O	-0.542180	1.524210	1.675711
C	2.426989	2.177881	0.649641
C	2.790890	-2.218299	1.921081
C	4.434100	-1.343388	-0.567249
C	-0.044211	2.735501	-1.640979
C	-3.388771	1.965170	1.131441
C	-1.024500	0.957980	2.854501
C	1.779299	3.241131	0.003831
C	2.913229	2.280001	1.946131
C	1.943750	-2.887579	2.802241
C	3.976460	-2.802389	1.439691
C	5.293430	-0.701508	-1.452589
C	4.899630	-2.088858	0.526041
C	-0.577611	2.626070	-2.918229
C	1.292859	3.091341	-1.408009
C	-4.263591	2.757520	0.393621
C	-3.269311	2.069180	2.524081
C	-0.123340	0.231251	3.633231
C	-2.335620	1.218130	3.294971
C	1.591189	4.444411	0.719131
C	2.715899	3.472251	2.636921
H	3.438800	1.444851	2.394641
C	2.266551	-4.177539	3.223821
H	1.053820	-2.384339	3.164531
C	4.271601	-4.104499	1.880121
C	6.670140	-0.793278	-1.252999
H	4.874750	-0.149228	-2.287129
C	6.292470	-2.160518	0.703981
C	0.257779	2.870661	-4.006599
H	-1.622811	2.359640	-3.042319
C	2.125459	3.332901	-2.521899
C	-5.056231	3.690710	1.062111
H	-4.324941	2.609440	-0.677439
C	-4.081231	3.019280	3.168441
C	-0.527630	-0.282340	4.866121
H	0.891570	0.106041	3.274601
C	-2.717320	0.677680	4.536121
C	2.058159	4.555501	2.047981
H	3.088159	3.564091	3.653831
C	3.433501	-4.786989	2.759221
H	1.609801	-4.699409	3.913381
H	5.170081	-4.587178	1.507691
C	7.168770	-1.523248	-0.171219
H	7.348820	-0.294778	-1.938419
H	6.683490	-2.711648	1.554041
C	1.601119	3.217651	-3.830509
H	-0.144701	2.796151	-5.013489
C	-4.965041	3.820920	2.450111
H	-5.741491	4.315399	0.496771
H	-3.993211	3.140250	4.244061
C	-1.833270	-0.066460	5.314291
H	0.179510	-0.836769	5.476681
H	-3.731540	0.847190	4.885341
H	3.690271	-5.792629	3.077911
H	8.239610	-1.591358	-0.005679
H	-5.574711	4.552359	2.972091
H	-2.158720	-0.465490	6.270391
H	0.470620	0.040471	-1.923939
C	2.472819	3.480621	-5.036399
H	3.346389	2.817101	-5.060939
H	1.913899	3.331071	-5.964679
H	2.858399	4.508051	-5.042839
C	3.573509	3.725891	-2.338529

H	4.244939	2.979421	-2.782019
H	3.791519	4.678481	-2.836949
H	3.839429	3.829441	-1.285729
C	0.887079	5.623761	0.088041
H	1.491808	6.534971	0.167401
H	0.667419	5.457651	-0.967309
H	-0.063251	5.829461	0.597331
C	1.860509	5.830731	2.833421
H	2.258829	5.731841	3.847291
H	2.363718	6.683611	2.360271
H	0.799179	6.096411	2.914911
C	-1.191969	-4.190280	-2.772149
H	-0.870579	-5.208420	-2.510479
H	-2.251989	-4.228520	-3.037729
H	-0.615919	-3.884280	-3.644189
C	-1.661359	-3.572520	-0.330119
C	-2.587699	-4.624780	-0.227839
C	-1.440560	-2.789970	0.819901
C	-3.265869	-4.870930	0.967081
H	-2.776449	-5.269640	-1.078629
C	-2.138259	-3.003140	2.004141
H	-0.645110	-2.023390	0.860641
C	-3.056819	-4.054970	2.079561
H	-3.962199	-5.702510	1.024961
H	-1.954220	-2.366470	2.864101
H	-3.595019	-4.241170	3.004211

TS-III-L-8 Conformation 1

B3LYP/6-31G(d),SDD(Ir) Energy = -3450.979011
B3LYP/def2-TZVPP,SDD(Ir)/IEFPCM(dioxane) Energy = -3452.148857
B3LYP/def2-TZVPP,SDD(Ir)/IEFPCM(dioxane)//B3LYP/6-31G(d),SDD(Ir) Free Energy (Quasiharmonic) = -3451.434373
Number of Imaginary Frequencies = 1

Frequencies (Top 3 out of 315)

1. -360.9837 cm^{-1}
2. 11.0016 cm^{-1}
3. 19.0429 cm^{-1}

B3LYP/6-31G(d),SDD(Ir) Molecular Geometry in Cartesian Coordinates

C	-0.816820	2.326933	-1.334064
C	0.363616	2.808617	-2.072712
C	1.749155	1.533092	-1.364325
C	2.525367	1.954339	-0.269730
O	2.629217	1.273787	-2.396481
C	3.886063	1.942329	-0.659639
C	3.881730	1.523936	-1.966329
H	4.673895	1.361212	-2.683355
H	4.764812	2.162742	-0.071533
H	-1.099604	2.893639	-0.449526
H	-1.678227	2.153197	-1.977628
C	1.834258	2.116566	1.018992
O	0.722194	1.524624	1.152265
N	2.353011	2.825185	2.038053
C	1.684745	2.775537	3.340016
H	2.411818	3.040752	4.111188
H	0.844005	3.478584	3.380153
H	1.314876	1.766572	3.524036
C	3.439551	3.793954	1.897717
H	3.148948	4.723317	2.397815
H	4.362697	3.424479	2.360026
H	3.617921	4.023542	0.848458
Ir	-0.004808	0.422320	-0.727998
P	-2.070966	-0.277502	-0.095448
P	1.215733	-1.502376	-0.304500
O	-2.592823	-1.774356	-0.454914
O	-2.494177	-0.190328	1.493595
O	-3.241183	0.580890	-0.822480
O	1.159329	-2.753819	-1.346494
O	2.820989	-1.194973	-0.227516
O	0.994012	-2.368947	1.075110
C	-2.171071	-2.999762	0.074182
C	-2.961761	0.994160	2.077520
C	-4.598204	0.395563	-0.506496
C	0.242842	-2.910859	-2.389105
C	3.762848	-2.149024	0.186026
C	1.502933	-1.936889	2.299579
C	-1.422714	-3.853752	-0.745485
C	-2.585023	-3.374899	1.351794
C	-2.175139	1.575827	3.068701
C	-4.230114	1.498447	1.738722
C	-5.414922	-0.170291	-1.479822
C	-5.097653	0.861692	0.718919
C	0.653254	-2.585551	-3.680690
C	-1.019037	-3.460147	-2.127853
C	4.663767	-2.618402	-0.765400
C	3.838392	-2.523150	1.537020
C	0.595147	-1.536677	3.278394
C	2.883680	-2.020487	2.553363
C	-1.073344	-5.109538	-0.227994
C	-2.222238	-4.628890	1.841618
H	-3.189976	-2.692240	1.937513
C	-2.646103	2.695602	3.752620
C	-4.671374	2.635760	2.439000
C	-6.782937	-0.286245	-1.238712
C	-6.480871	0.726186	0.934877
C	-0.218610	-2.795845	-4.748820
H	1.649209	-2.182576	-3.834510
C	-1.878275	-3.658911	-3.218353
C	5.680897	-3.488848	-0.376848
H	4.560160	-2.292096	-1.794973
C	4.873380	-3.403869	1.898525
C	1.059437	-1.193617	4.548206
H	-0.461724	-1.520242	3.035439
C	3.322496	-1.657495	3.839354
C	-1.463841	-5.496381	1.052958
H	-0.490580	-5.784463	-0.847851
H	-2.542404	-4.929409	2.834957
C	-3.897050	3.228545	3.433384
H	-2.040560	3.146801	4.533535
C	-7.314906	0.161898	-0.027192
H	-7.427912	-0.727242	-1.992642
C	-1.488099	-3.329671	-4.516612
H	0.097170	-2.548991	-5.758174
H	-2.859550	-4.087366	-3.036185
C	5.785139	-3.880686	0.959749
H	6.384115	-3.861369	-1.115617
H	4.941642	-3.732533	2.931220
C	2.428078	-1.248696	4.826452
H	0.353414	-0.896521	5.318553
H	4.385627	-1.697290	4.057901
H	-1.184307	-6.475303	1.430446
H	-2.168857	-3.500468	-5.345172
H	6.568591	-4.565815	1.268957
H	2.797642	-0.982823	5.812465
H	-0.382560	-0.169062	-2.134268
H	-1.209087	1.136465	3.289044
H	-4.971057	-0.503165	-2.412025
H	-8.378061	0.066156	0.171183
H	-6.896141	1.055049	1.882774
H	-5.636821	3.061294	2.182652
H	-4.269358	4.104491	3.956028
C	0.313874	2.467465	-3.557258
H	-0.343812	3.208324	-4.029938
H	1.280531	2.512215	-4.057195
H	-0.111045	1.474912	-3.713528
C	0.994005	4.137637	-1.753377
C	2.086943	4.610676	-2.508346
C	0.501975	4.961621	-0.723800
C	2.666385	5.848937	-2.240145
H	2.494195	4.009586	-3.313011
C	1.079245	6.203812	-0.460468
H	-0.353907	4.651948	-0.136399
C	2.166233	6.652598	-1.213083

H	3.504144	6.190531	-2.841398
H	0.665292	6.829325	0.325570
H	2.610067	7.623172	-1.011168

TS-III-L-8 Conformation 2

B3LYP/6-31G(d),SDD(Ir) Energy = -3450.978428
B3LYP/def2-TZVPP,SDD(Ir)/IEFPCM(dioxane) Energy = -3452.147949
B3LYP/def2-TZVPP,SDD(Ir)/IEFPCM(dioxane)//B3LYP/6-31G(d),SDD(Ir) Free Energy (Quasiharmonic) = -3451.433057
Number of Imaginary Frequencies = 1

Frequencies (Top 3 out of 315)

1. -360.3937 cm^{-1}
2. 12.3945 cm^{-1}
3. 18.7982 cm^{-1}

B3LYP/6-31G(d),SDD(Ir) Molecular Geometry in Cartesian Coordinates

C	-0.136105	2.668221	-0.881369
C	1.164011	2.799250	-1.560037
C	2.104533	1.186159	-0.780222
C	2.913463	1.388880	0.352308
O	2.922742	0.677343	-1.766795
C	4.232343	0.987049	0.030512
C	4.172332	0.567192	-1.274907
H	4.915754	0.169644	-1.950721
H	5.108781	0.958270	0.660917
H	-0.293462	3.297883	-0.008506
H	-0.980949	2.731463	-1.565753
C	2.226699	1.772540	1.595958
O	0.983801	1.533627	1.657046
N	2.869059	2.334219	2.636398
C	2.155797	2.544134	3.896659
H	2.838568	2.334954	4.725948
H	1.810413	3.582523	3.976940
H	1.297392	1.876589	3.947755
C	4.216534	2.896758	2.563849
H	4.219268	3.860094	3.083226
H	4.949299	2.240771	3.049419
H	4.506975	3.074911	1.529511
Ir	0.083292	0.622704	-0.235359
P	0.709363	-1.567826	0.215023
P	-2.133583	0.496901	0.262489
O	0.047135	-2.461638	1.409843
O	0.619096	-2.661488	-1.017968
O	2.276032	-1.674655	0.651627
O	-2.691001	0.416204	1.789391
O	-2.938753	1.793817	-0.303227
O	-3.008121	-0.737930	-0.362627
C	-1.265471	-2.945306	1.474080
C	1.575122	-2.667432	-2.038450
C	2.947340	-2.905210	0.744301
C	-2.000556	0.098591	2.959318
C	-4.340251	1.862464	-0.353414
C	-3.375055	-0.758502	-1.710932
C	-2.143785	-2.365146	2.397102
C	-1.634830	-4.037927	0.689730
C	1.176922	-2.280774	-3.315946
C	2.869719	-3.149295	-1.773135
C	3.343788	-3.330287	2.008247
C	3.278242	-3.610818	-0.423914
C	-1.708595	1.141974	3.836466
C	-1.731056	-1.237514	3.284391
C	-4.967779	2.790630	0.471354
C	-5.046289	1.088808	-1.287602
C	-2.798496	-1.729841	-2.526905
C	-4.376257	0.111051	-2.177342
C	-3.435207	-2.904036	2.494680
C	-2.925367	-4.552569	0.805505
H	-0.911467	-4.473896	0.010546
C	2.082814	-2.364755	-4.372939
C	3.761286	-3.217915	-2.858329
C	4.099563	-4.495207	2.132669
C	4.038916	-4.782991	-0.265809
C	-1.115134	0.859778	5.066013
H	-1.966126	2.156913	3.551839
C	-1.117611	-1.493027	4.520442
C	-6.347476	2.966418	0.377900
H	-4.367994	3.370209	1.165238
C	-6.436830	1.286140	-1.356191
C	-3.206630	-1.840250	-3.856047
H	-2.057708	-2.398100	-2.101332
C	-4.758667	-0.018511	-3.524372
C	-3.828489	-3.983351	1.705452
H	-4.130436	-2.465472	3.204422
H	-3.219155	-5.403820	0.198393
C	3.378609	-2.832185	-4.141792
H	1.775086	-2.069318	-5.371613
C	4.447717	-5.221642	0.991539
H	4.409680	-4.834819	3.116305
C	-0.809553	-0.460548	5.405394
H	-0.906190	1.668215	5.761129
H	-0.905003	-2.523373	4.790170
C	-7.082144	2.211106	-0.538913
H	-6.844028	3.688441	1.019049
H	-7.015099	0.684811	-2.051222
C	-4.183991	-0.976688	-4.356504
H	-2.767414	-2.601659	-4.494137
H	-5.513914	0.654811	-3.918576
H	-4.832928	-4.384585	1.800270
H	-0.352935	-0.688241	6.364068
H	-8.158153	2.336277	-0.612189
H	-4.501771	-1.051265	-5.392110
H	-0.366889	0.138976	-1.664122
H	0.160904	-1.931680	-3.467678
H	3.059211	-2.742327	2.874577
H	5.028157	-6.134834	1.081044
H	4.290523	-5.364297	-1.147872
H	4.771355	-3.575984	-2.681057
H	4.089483	-2.900154	-4.959923
C	2.124760	3.900434	-1.205108
C	1.829251	4.845860	-0.205107
C	3.345711	4.035587	-1.897741
C	2.717015	5.880410	0.089951
H	0.890005	4.797742	0.332411
C	4.234218	5.065597	-1.597611
H	3.610590	3.330349	-2.676963
C	3.925135	5.993839	-0.600529
H	2.454066	6.609438	0.851398
H	5.165602	5.148722	-2.150456
H	4.612277	6.803998	-0.374173
C	1.097401	2.464584	-3.044652
H	0.417413	1.630814	-3.224485
H	0.702247	3.354457	-3.551655
H	2.061779	2.226075	-3.491136

TS-III-L-8 Conformation 3

B3LYP/6-31G(d),SDD(Ir) Energy = -3450.976182
B3LYP/def2-TZVPP,SDD(Ir)/IEFPCM(dioxane) Energy = -3452.146158
B3LYP/def2-TZVPP,SDD(Ir)/IEFPCM(dioxane)//B3LYP/6-31G(d),SDD(Ir) Free Energy (Quasiharmonic) = -3451.431050
Number of Imaginary Frequencies = 1

Frequencies (Top 3 out of 315)

1. -357.2130 cm^{-1}
2. 11.7749 cm^{-1}
3. 17.3602 cm^{-1}

B3LYP/6-31G(d),SDD(Ir) Molecular Geometry in Cartesian Coordinates

C	-1.320704	2.342874	0.974784
C	-0.262084	3.360705	1.148019
C	1.368051	2.283972	0.743514
C	2.149528	1.836853	1.825533
O	2.201108	3.035779	-0.059447
C	3.466563	2.334571	1.655769
C	3.427514	3.058099	0.490228
H	4.179343	3.622357	-0.043169
H	4.344976	2.159885	2.259381
H	-1.737978	1.997223	1.922496
H	-2.114832	2.616243	0.285625
C	-0.027495	3.756839	2.603507
H	-0.824955	4.456142	2.887664
H	-0.110206	2.886478	3.256914
H	0.934465	4.242520	2.779180
C	-0.178562	4.480391	0.149161
C	0.405332	5.713073	0.495678
C	-0.711265	4.347855	-1.146925
C	0.438741	6.774743	-0.407662
H	0.826232	5.865246	1.482728
C	-0.674635	5.406219	-2.050362
H	-1.150442	3.408069	-1.459420
C	-0.100379	6.626544	-1.685467
H	0.883393	7.719371	-0.107962
H	-1.098890	5.277733	-3.042031
H	-0.075747	7.453123	-2.389743
C	1.539673	0.830213	2.712985
O	0.529852	0.209013	2.266881
N	2.024976	0.542676	3.934950
C	1.458963	-0.591233	4.669935
H	2.207593	-0.950853	5.379816
H	0.556583	-0.294623	5.218118
H	1.208254	-1.391386	3.973815
C	2.991926	1.367727	4.657028
H	2.643008	1.501546	5.685879
H	3.978951	0.890700	4.685346
H	3.076560	2.352435	4.199823
Ir	-0.095589	0.787868	0.124266
P	-1.944348	-0.477399	-0.235012
P	1.505337	-0.580634	-0.851965
O	-2.160228	-1.256637	-1.645458
O	-2.309661	-1.679997	0.828192
O	-3.295603	0.421504	-0.233536
O	1.671243	-0.608248	-2.472451
O	3.020788	-0.205765	-0.362455
O	1.492249	-2.208311	-0.614578
C	-1.482976	-2.375121	-2.145030
C	-2.975456	-1.424219	2.034984
C	-4.571381	-0.161831	-0.323826
C	0.769099	-0.084601	-3.401669
C	4.142200	-0.985099	-0.685463
C	1.949291	-2.785487	0.569141
C	-0.606755	-2.193943	-3.222475
C	-1.769323	-3.638225	-1.628455
C	-2.275074	-1.649036	3.217285
C	-4.332853	-1.057346	2.028259
C	-5.299999	0.059298	-1.487780
C	-5.099327	-0.855919	0.775349
C	1.056722	1.155643	-3.968651
C	-0.339936	-0.842921	-3.799310
C	5.087358	-0.428182	-1.542041
C	4.329616	-2.235335	-0.075039
C	1.013328	-3.407757	1.393602
C	3.328196	-2.828105	0.843138
C	0.005246	-3.335038	-3.761141
C	-1.145277	-4.755337	-2.182214
H	-2.478843	-3.732261	-0.814314
C	-2.928064	-1.507462	4.440880
C	-4.959888	-0.913009	3.278894
C	-6.604225	-0.424325	-1.578146
C	-6.415636	-1.335765	0.652157
C	0.213028	1.670192	-4.952825
H	1.938354	1.696772	-3.640606
C	-1.174654	-0.301601	-4.787884
C	6.264783	-1.124346	-1.810653
H	4.891297	0.543983	-1.982057
C	5.525735	-2.914953	-0.366995
C	1.443660	-4.086854	2.533435
H	-0.034581	-3.367038	1.117139
C	3.730060	-3.510103	2.005850
C	-0.255220	-4.604395	-3.247420
H	0.689880	-3.214059	-4.595421
H	-1.364526	-5.742776	-1.786689
C	-4.273921	-1.135430	4.470479
H	-2.388732	-1.689292	5.366077
C	-7.160936	-1.124716	-0.505270
H	-7.180057	-0.256672	-2.483240
C	-0.906643	0.941923	-5.360663
H	0.436201	2.633224	-5.402547
H	-2.036955	-0.876726	-5.112427
C	6.483283	-2.370892	-1.219630
H	7.003881	-0.696452	-2.481244
H	5.687671	-3.896340	0.068619
C	2.806052	-4.132574	2.842572
H	0.718463	-4.586380	3.169580
H	4.787752	-3.545187	2.250113
H	0.228152	-5.473381	-3.683471
H	-1.563439	1.334581	-6.131160
H	7.393234	-2.924118	-1.431122
H	3.149373	-4.659084	3.728245
H	-0.397362	1.383544	-1.295549
H	-1.231416	-1.935547	3.156784
H	-4.840648	0.612013	-2.300392
H	-8.172999	-1.512105	-0.572152
H	-6.845867	-1.897421	1.475909
H	-6.001548	-0.607884	3.305724
H	-4.788600	-1.015777	5.419038

TS-III-L-8 Conformation 4

B3LYP/6-31G(d),SDD(Ir) Energy = -3450.97552
B3LYP/def2-TZVPP,SDD(Ir)/IEFPCM(dioxane) Energy = -3452.145039
B3LYP/def2-TZVPP,SDD(Ir)/IEFPCM(dioxane)//B3LYP/6-31G(d),SDD(Ir) Free Energy (Quasiharmonic) = -3451.429674
Number of Imaginary Frequencies = 1

Frequencies (Top 3 out of 315)

1. -356.1813 cm^{-1}
2. 11.3338 cm^{-1}
3. 16.3484 cm^{-1}

B3LYP/6-31G(d),SDD(Ir) Molecular Geometry in Cartesian Coordinates

C	0.708982	2.021406	-1.975735
C	-0.572324	2.737512	-2.140791
C	-1.835260	1.416435	-1.300268
C	-2.602081	0.603113	-2.155647
O	-2.725306	2.109568	-0.509323
C	-3.971778	0.819890	-1.857133
C	-3.975410	1.749290	-0.846452
H	-4.772390	2.220577	-0.289657
H	-4.845388	0.339648	-2.272259
H	1.075850	1.584044	-2.905998
H	1.495384	2.588004	-1.485578
C	-1.075839	2.778730	-3.580454
H	-0.517766	3.568055	-4.101626
H	-0.862474	1.837756	-4.090447
H	-2.141862	2.996468	-3.669967
C	-0.808519	3.981200	-1.333833
C	-1.742153	4.946879	-1.754631
C	-0.079224	4.242797	-0.158483
C	-1.926418	6.131136	-1.042580
H	-2.326370	4.792398	-2.654293
C	-0.264288	5.424184	0.554098
H	0.636028	3.517124	0.209562
C	-1.188377	6.375859	0.115501

H	-2.644453	6.864301	-1.398555
H	0.319625	5.604156	1.452271
H	-1.328694	7.299640	0.669444
C	-1.859496	-0.387282	-2.956963
O	-0.674672	-0.644253	-2.588724
N	-2.392144	-1.009257	-4.025626
C	-1.650515	-2.089023	-4.679574
H	-2.349602	-2.890607	-4.937972
H	-1.174002	-1.726499	-5.598875
H	-0.885703	-2.469276	-4.005037
C	-3.648899	-0.624339	-4.664343
H	-3.515186	-0.665595	-5.749716
H	-4.461887	-1.308189	-4.391168
H	-3.924110	0.394991	-4.396963
Ir	0.011789	0.440124	-0.690022
P	-1.094428	-1.013631	0.750373
P	2.147459	-0.268513	-0.366953
O	-0.604069	-2.549493	1.005992
O	-1.310284	-0.535285	2.314862
O	-2.623411	-1.314543	0.271109
O	2.717608	-1.665689	-0.975799
O	3.221314	0.799796	-0.962494
O	2.682570	-0.454492	1.169967
C	0.558403	-2.978878	1.657012
C	-2.292395	0.404485	2.645556
C	-3.548639	-1.980829	1.092005
C	2.011081	-2.761632	-1.471733
C	4.596935	0.733572	-0.689384
C	2.995224	0.648868	1.967809
C	1.571692	-3.563542	0.887782
C	0.639709	-2.899048	3.047063
C	-1.880186	1.663364	3.075214
C	-3.647584	0.028247	2.626171
C	-3.968978	-3.246648	0.696400
C	-4.083989	-1.328070	2.214146
C	1.988585	-2.940864	-2.853936
C	1.458725	-3.704398	-0.594319
C	5.449472	0.442474	-1.749508
C	5.072850	1.060179	0.589891
C	2.177212	0.910859	3.065237
C	4.164202	1.387560	1.713965
C	2.701226	-4.051616	1.560988
C	1.775090	-3.389601	3.690597
H	-0.183045	-2.466334	3.604283
C	-2.831681	2.590664	3.498236
C	-4.583303	0.985955	3.055965
C	-4.959178	-3.899176	1.429568
C	-5.078953	-2.012510	2.934625
C	1.388256	-4.080117	-3.388496
H	2.456991	-2.196714	-3.489802
C	0.846829	-4.834207	-1.158348
C	6.828216	0.470013	-1.545733
H	5.024144	0.210973	-2.720365
C	6.467864	1.076549	0.765335
C	2.510214	1.946621	3.938050
H	1.307294	0.284447	3.230519
C	4.467395	2.431142	2.606420
C	2.808896	-3.963180	2.947825
H	3.497880	-4.506840	0.980004
H	1.844665	-3.331236	4.772859
C	-4.186356	2.250642	3.486666
H	-2.514474	3.573155	3.834427
C	-5.515004	-3.278575	2.550719
H	-5.289720	-4.888775	1.128745
C	0.808314	-5.025679	-2.539090
H	1.387740	-4.233680	-4.464022
H	0.417636	-5.577628	-0.492970
C	7.336727	0.787766	-0.284254
H	7.499674	0.243185	-2.368306
H	6.867196	1.300980	1.749851
C	3.655106	2.711856	3.703011
H	1.881814	2.148494	4.800726
H	5.352781	3.032526	2.423109
H	3.692197	-4.349691	3.447087
H	0.345867	-5.919085	-2.948279
H	8.409169	0.803360	-0.115586
H	3.918216	3.523644	4.374513
H	0.302485	1.362868	0.548879
H	-0.820755	1.896654	3.081100
H	-3.518401	-3.701144	-0.179745
H	-6.279864	-3.784205	3.132233
H	-5.495899	-1.544333	3.821311
H	-5.638088	0.726889	3.041883
H	-4.933005	2.967832	3.814430

TS-III-L-8 Conformation 5

B3LYP/6-31G(d),SDD(Ir) Energy = -3450.963797
B3LYP/def2-TZVPP,SDD(Ir)/IEFPCM(dioxane) Energy = -3452.134997
B3LYP/def2-TZVPP,SDD(Ir)/IEFPCM(dioxane)//B3LYP/6-31G(d),SDD(Ir) Free Energy (Quasiharmonic) = -3451.420029
Number of Imaginary Frequencies = 1

Frequencies (Top 3 out of 315)

1. -357.5903 cm^{-1}
2. 12.6477 cm^{-1}
3. 18.8418 cm^{-1}

B3LYP/6-31G(d),SDD(Ir) Molecular Geometry in Cartesian Coordinates

C	-0.320973	2.773620	0.639436
C	0.139365	3.301525	-0.658511
C	1.030401	1.610093	-1.529267
C	2.427428	1.527377	-1.468122
O	0.680071	1.539493	-2.852967
C	2.919426	1.379179	-2.796161
C	1.806821	1.395793	-3.593391
H	1.666349	1.307733	-4.660703
H	3.935491	1.247554	-3.137814
H	0.226482	3.108729	1.515621
H	-1.391909	2.911220	0.782063
C	3.001163	1.517499	-0.122184
O	2.191812	1.305855	0.842021
N	4.298297	1.726736	0.144571
C	4.792604	1.552854	1.512606
H	5.801550	1.133081	1.468099
H	4.829385	2.516053	2.035741
H	4.135862	0.876085	2.056500
C	5.254244	2.262504	-0.823181
H	5.900049	2.980558	-0.309611
H	5.883483	1.469581	-1.245139
H	4.736988	2.792069	-1.622039
Ir	0.163817	0.690516	0.347845
P	-1.959477	0.081529	0.095897
P	0.946580	-1.500448	0.122568
O	-2.205345	-1.144993	-0.910801
O	-2.784536	-0.401206	1.426024
O	-2.927405	1.259820	-0.482581
O	0.331530	-2.595200	-0.917700
O	2.574047	-1.520905	-0.087539
O	0.745616	-2.502439	1.401321
C	-2.785841	-2.418814	-0.916141
C	-3.314382	0.536964	2.324293
C	-4.322972	1.102096	-0.550529
C	0.106249	-2.467032	-2.285600
C	3.393379	-2.634224	0.165467
C	1.451997	-2.280208	2.584670
C	-2.246907	-3.303102	-1.864675
C	-3.866307	-2.766092	-0.111279
C	-2.740851	0.630860	3.589154
C	-4.455360	1.275316	1.964057
C	-4.901912	0.997619	-1.811172
C	-5.083993	1.146309	0.628309
C	1.124920	-2.064450	-3.146691
C	-1.143447	-2.887330	-2.770557
C	4.109453	-3.155764	-0.908973

C	3.562410	-3.114838	1.474687
C	0.736215	-1.877339	3.708994
C	2.826119	-2.567610	2.639525
C	-2.831875	-4.571475	-1.976421
C	-4.416829	-4.043707	-0.236955
H	-4.275232	-2.054105	0.592415
C	-3.305125	1.485070	4.536329
C	-5.000130	2.128527	2.939132
C	-6.290847	0.937752	-1.919528
C	-6.481339	1.081962	0.486456
C	0.911003	-2.087265	-4.526670
H	2.080525	-1.755744	-2.740369
C	-1.324406	-2.913713	-4.160649
C	5.016982	-4.192502	-0.698320
H	3.955607	-2.742645	-1.899827
C	4.481705	-4.164371	1.654484
C	1.397326	-1.719476	4.926200
H	-0.332476	-1.718596	3.615553
C	3.468914	-2.387075	3.877612
C	-3.903704	-4.946941	-1.166826
H	-2.424465	-5.269715	-2.701800
H	-5.256222	-4.322729	0.393038
C	-4.436551	2.235238	4.208857
H	-2.862700	1.560834	5.524996
C	-7.079849	0.980843	-0.767632
H	-6.752039	0.856132	-2.899005
C	-0.311452	-2.521336	-5.036614
H	1.710225	-1.785199	-5.197806
H	-2.285183	-3.236590	-4.550947
C	5.202434	-4.698221	0.589420
H	5.570694	-4.603128	-1.537232
H	4.609288	-4.576870	2.650612
C	2.770253	-1.964188	5.006295
H	0.842321	-1.410180	5.806822
H	4.534947	-2.583066	3.945775
H	-4.334610	-5.938694	-1.263592
H	-0.479626	-2.554102	-6.108800
H	5.898342	-5.513184	0.763280
H	3.294586	-1.835514	5.948371
H	-0.112973	0.412634	1.900149
H	-1.868587	0.029630	3.818638
H	-4.262280	0.975372	-2.687399
H	-8.161504	0.927899	-0.844971
H	-7.097982	1.093857	1.380207
H	-5.870349	2.725186	2.682507
H	-4.878301	2.905557	4.939813
C	-0.973437	3.562322	-1.658539
H	-1.728196	2.779475	-1.619579
H	-1.445027	4.508642	-1.359811
H	-0.630566	3.670553	-2.686360
C	1.292049	4.264118	-0.724039
C	1.683355	4.830569	-1.956286
C	1.984967	4.674465	0.430976
C	2.717114	5.759312	-2.029387
H	1.183837	4.539640	-2.872979
C	3.018827	5.609017	0.357499
H	1.713371	4.280973	1.401995
C	3.391842	6.155228	-0.870691
H	2.990874	6.182552	-2.991672
H	3.521981	5.920554	1.268642
H	4.190301	6.889667	-0.925455

TS-III-L-8 Conformation 6

B3LYP/6-31G(d),SDD(Ir) Energy = -3450.956841
B3LYP/def2-TZVPP,SDD(Ir)/IEFPCM(dioxane) Energy = -3452.128686
B3LYP/def2-TZVPP,SDD(Ir)/IEFPCM(dioxane)//B3LYP/6-31G(d),SDD(Ir) Free Energy (Quasiharmonic) = -3451.413388
Number of Imaginary Frequencies = 1

Frequencies (Top 3 out of 315)

1. -363.8067 cm^{-1}
2. 10.6048 cm^{-1}
3. 17.3406 cm^{-1}

B3LYP/6-31G(d),SDD(Ir) Molecular Geometry in Cartesian Coordinates

C	1.133690	2.099090	-1.687590
C	1.050060	3.203670	-0.710090
C	-0.259900	2.393629	0.725940
C	-1.572900	2.886289	0.738940
O	0.225570	2.507089	2.000720
C	-1.875390	3.293249	2.072590
C	-0.738120	3.035669	2.789820
H	-0.483360	3.162759	3.831670
H	-2.797871	3.684449	2.475820
H	0.673210	2.349040	-2.644490
H	2.127970	1.691920	-1.840720
C	-2.332340	2.697269	-0.502140
O	-1.826660	1.904529	-1.365300
N	-3.494160	3.308999	-0.775220
C	-4.248080	2.933258	-1.975020
H	-5.315190	2.949558	-1.735570
H	-4.055091	3.640469	-2.790610
H	-3.956690	1.933569	-2.292000
C	-4.044211	4.412409	0.009440
H	-4.463971	5.153008	-0.677730
H	-4.843261	4.067498	0.676800
H	-3.263181	4.898399	0.592760
Ir	-0.151430	0.659539	-0.715820
P	1.548600	-0.677660	-0.219550
P	-1.730050	-0.801481	0.210440
O	1.350111	-1.558360	1.106430
O	1.990451	-1.813640	-1.314970
O	2.948480	0.110550	0.030920
O	-1.501859	-1.704201	1.549500
O	-3.184260	-0.060201	0.396050
O	-2.120889	-2.114561	-0.686510
C	1.374991	-2.893740	1.523210
C	2.774521	-1.472930	-2.425860
C	4.164910	-0.571790	0.196470
C	-1.078269	-1.303761	2.813440
C	-4.412840	-0.735452	0.492280
C	-2.810209	-1.960031	-1.890360
C	0.654711	-3.148181	2.701720
C	2.109841	-3.883070	0.877450
C	2.182351	-1.518880	-3.684880
C	4.141780	-1.194770	-2.251190
C	4.762970	-0.530590	1.452080
C	4.776420	-1.181020	-0.911430
C	-1.730050	-0.279561	3.497180
C	-0.062119	-2.061571	3.419840
C	-5.142830	-0.565882	1.666110
C	-4.923579	-1.451952	-0.603150
C	-2.134289	-2.240451	-3.075050
C	-4.176629	-1.633841	-1.871110
C	0.691141	-4.450680	3.217120
C	2.113641	-5.174690	1.409540
H	2.672461	-3.651200	-0.016260
C	2.959891	-1.277620	-4.817320
C	4.898630	-0.954680	-3.411270
C	6.016160	-1.116219	1.628370
C	6.038621	-1.763949	-0.701100
C	-1.379110	0.000699	4.819730
H	-2.518180	0.276889	3.004670
C	0.259141	-1.767021	4.752510
C	-6.414550	-1.125332	1.778180
H	-4.712580	0.006828	2.480400
C	-6.207399	-2.008462	-0.457880
C	-2.815649	-2.169941	-4.289370
H	-1.090699	-2.531121	-3.023100
C	-4.834809	-1.558632	-3.112000
C	1.407611	-5.461610	2.576920
H	0.135191	-4.666181	4.124960
H	2.680401	-5.952470	0.906360
C	4.320390	-0.994370	-4.678340

H	2.503771	-1.313580	-5.801980
C	6.652971	-1.733629	0.549020
H	6.490370	-1.091009	2.604760
C	-0.389470	-0.749201	5.452940
H	-1.897140	0.791109	5.355810
H	1.043091	-2.343650	5.234750
C	-6.947569	-1.849912	0.710820
H	-6.980620	-0.998192	2.696000
H	-6.614169	-2.593932	-1.276700
C	-4.167069	-1.817011	-4.307090
H	-2.294009	-2.391841	-5.215550
H	-5.887179	-1.290772	-3.129740
H	1.413202	-6.466170	2.988540
H	-0.120500	-0.545191	6.484910
H	-7.932389	-2.299752	0.791180
H	-4.702839	-1.751782	-5.249250
H	-0.225750	-0.179661	-2.078560
H	1.126211	-1.751830	-3.762120
H	4.247160	-0.031110	2.265350
H	7.625311	-2.198069	0.682020
H	6.528821	-2.262169	-1.532170
H	5.953680	-0.719799	-3.306500
H	4.930680	-0.801530	-5.555340
C	0.240499	4.393399	-1.214170
H	-0.060191	5.089859	-0.430400
H	0.902399	4.935670	-1.903620
H	-0.631351	4.076199	-1.786020
C	2.226920	3.608990	0.125510
C	3.517740	3.111850	-0.124370
C	2.090489	4.608410	1.111940
C	4.621550	3.587220	0.584270
H	3.677600	2.366120	-0.890460
C	3.188109	5.075880	1.825530
H	1.111559	5.017950	1.337540
C	4.463169	4.565840	1.564220
H	5.608330	3.192561	0.360510
H	3.051369	5.843120	2.582190
H	5.323639	4.935670	2.114420

TS-III-L-8 Conformation 7

B3LYP/6-31G(d),SDD(Ir) Energy = -3450.922785
B3LYP/def2-TZVPP,SDD(Ir)/IEFPCM(dioxane) Energy = -3452.097324
B3LYP/def2-TZVPP,SDD(Ir)/IEFPCM(dioxane)//B3LYP/6-31G(d),SDD(Ir) Free Energy (Quasiharmonic) = -3451.385525
Number of Imaginary Frequencies = 1

Frequencies (Top 3 out of 315)

1. -275.0545 cm^{-1}
2. 10.4273 cm^{-1}
3. 12.2116 cm^{-1}

B3LYP/6-31G(d),SDD(Ir) Molecular Geometry in Cartesian Coordinates

C	0.768028	2.647007	-0.037200
C	-0.485991	3.192594	-0.602817
C	-1.593533	2.060528	0.818287
C	-3.006435	2.174026	0.878770
O	-1.127486	2.826233	1.919125
C	-3.345564	3.016299	1.962422
C	-2.159787	3.383441	2.546861
H	-1.929206	4.005264	3.400836
H	-4.340249	3.286153	2.289342
H	1.543663	2.581031	-0.807969
H	1.160971	3.150095	0.841504
C	-0.846880	2.523327	-1.923091
H	-0.296870	3.014441	-2.737240
H	-0.533171	1.464785	-1.941815
H	-1.910987	2.543482	-2.156471
C	-0.906181	4.598894	-0.412303
C	-1.759992	5.234718	-1.341280
C	-0.399659	5.378392	0.651033
C	-2.088276	6.581188	-1.214796
H	-2.145463	4.687114	-2.193161
C	-0.736315	6.723626	0.780098
H	0.275630	4.933797	1.371050
C	-1.582814	7.330736	-0.149300
H	-2.731357	7.049193	-1.954286
H	-0.326703	7.301806	1.603150
H	-1.838728	8.381630	-0.051303
C	-4.074270	1.310901	0.256987
O	-4.417543	0.336175	0.922276
N	-4.635772	1.644482	-0.937859
C	-5.681914	0.779256	-1.479240
H	-6.679155	1.142138	-1.196844
H	-5.607387	0.769831	-2.571164
H	-5.549542	-0.229975	-1.093190
C	-4.560064	2.978001	-1.513284
H	-4.279866	2.926473	-2.572664
H	-5.535709	3.479028	-1.447066
H	-3.829657	3.582703	-0.975696
Ir	-0.016320	0.673389	0.294870
P	2.114503	-0.174959	0.235804
P	-1.123890	-1.396825	0.268167
O	2.538525	-1.325571	1.298172
O	2.811451	-0.738335	-1.149048
O	3.152987	0.987280	0.691431
O	-1.381701	-2.173507	1.678027
O	-2.579551	-1.387844	-0.446560
O	-0.392708	-2.633832	-0.541240
C	2.219385	-2.691254	1.321167
C	3.271706	0.167402	-2.118002
C	4.545759	0.804766	0.638491
C	-0.767499	-1.789002	2.873884
C	-3.341906	-2.566605	-0.587004
C	-0.551479	-2.898235	-1.903252
C	1.266601	-3.151893	2.238929
C	2.931244	-3.556995	0.492865
C	2.582487	0.244006	-3.326778
C	4.449718	0.898224	-1.881131
C	5.237520	0.738444	1.843028
C	5.196521	0.796974	-0.604845
C	-1.499402	-1.005018	3.764705
C	0.527576	-2.241923	3.166640
C	-4.470125	-2.715655	0.214465
C	-2.986835	-3.497895	-1.571690
C	0.593846	-2.845496	-2.697489
C	-1.786884	-3.328508	-2.420984
C	1.027745	-4.534143	2.279318
C	2.672806	-4.925195	0.555227
H	3.679385	-3.152509	-0.179849
C	3.068074	1.069032	-4.342356
H	1.694981	-0.363687	-3.469872
C	4.910501	1.722374	-2.922402
C	6.629529	0.659717	1.825019
H	4.680750	0.758566	2.773829
C	6.599761	0.715085	-0.591350
C	-0.926139	-0.646306	4.984877
H	-2.502935	-0.694551	3.490241
C	1.081225	-1.860882	4.398196
C	-5.285499	-3.832592	0.031937
H	-4.700358	-1.947087	0.941394
C	-3.828109	-4.614032	-1.729052
C	0.520732	-3.191151	-4.046617
H	1.532263	-2.560010	-2.237815
C	-1.831456	-3.656620	-3.788333
C	1.717181	-5.414604	1.447610
H	0.290057	-4.912830	2.980557
H	3.223978	-5.605462	-0.087227
C	4.233766	1.810075	-4.137499
H	2.539849	1.125101	-5.289608
H	5.807810	2.312047	-2.760561
C	7.310105	0.647760	0.605079
H	7.178174	0.606163	2.760411
H	7.131122	0.685525	-1.537990
C	0.367526	-1.069570	5.299040

H	-1.492114	-0.042950	5.688565
H	2.079703	-2.206772	4.649366
C	-4.963594	-4.782115	-0.940682
H	-6.167652	-3.959824	0.652399
H	-3.565464	-5.367034	-2.465933
C	-0.699804	-3.588185	-4.596514
H	1.416322	-3.156274	-4.660314
H	-2.780519	-3.966273	-4.215606
H	1.514592	-6.479853	1.502656
H	4.615903	2.456658	-4.921587
H	8.393443	0.579107	0.585016
H	0.815752	-0.796717	6.249754
H	-5.590426	-5.657955	-1.078980
H	-0.769500	-3.850942	-5.647773
H	0.167116	0.520719	1.821031

TS-III-L-8 Conformation 8

B3LYP/6-31G(d),SDD(Ir) Energy = -3450.92544
B3LYP/def2-TZVPP,SDD(Ir)/IEFPCM(dioxane) Energy = -3452.097512
B3LYP/def2-TZVPP,SDD(Ir)/IEFPCM(dioxane)//B3LYP/6-31G(d),SDD(Ir) Free Energy (Quasiharmonic) = -3451.384898
Number of Imaginary Frequencies = 1

Frequencies (Top 3 out of 315)

1. -284.5582 cm^{-1}
2. 15.1468 cm^{-1}
3. 16.3371 cm^{-1}

B3LYP/6-31G(d),SDD(Ir) Molecular Geometry in Cartesian Coordinates

C	0.960624	-2.642517	-1.013829
C	-0.306240	-3.396505	-0.943838
C	-1.322968	-1.801088	-1.944061
C	-2.698378	-1.854952	-2.272344
O	-0.652307	-2.041553	-3.176259
C	-2.823340	-2.131946	-3.656931
C	-1.547367	-2.234448	-4.144284
H	-1.154972	-2.434935	-5.132026
H	-3.742207	-2.207234	-4.221946
H	1.624371	-2.845978	-0.169917
H	1.486490	-2.748583	-1.960067
C	-3.901560	-1.328736	-1.533462
O	-4.183860	-0.151674	-1.757000
N	-4.644435	-2.146501	-0.742344
C	-5.835554	-1.601325	-0.095681
H	-6.737448	-1.820348	-0.682774
H	-5.942985	-2.053340	0.895251
H	-5.728968	-0.522523	0.002736
C	-4.548191	-3.597677	-0.766277
H	-4.517392	-3.992600	0.253466
H	-5.415324	-4.031641	-1.283560
H	-3.641866	-3.904422	-1.287909
Ir	0.074984	-0.690860	-0.706644
P	2.143553	0.163008	-0.186495
P	-1.178831	1.170917	-0.051169
O	2.513506	1.651161	-0.714672
O	2.778359	0.191294	1.336510
O	3.258949	-0.683452	-1.007741
O	-1.375445	2.429146	-1.062155
O	-2.678429	0.829232	0.460723
O	-0.604085	2.014847	1.247480
C	2.120282	2.910746	-0.241031
C	3.356743	-0.955533	1.904247
C	4.633033	-0.414079	-0.876851
C	-0.672366	2.554442	-2.265346
C	-3.523745	1.820300	0.998334
C	-0.863475	1.667670	2.573557
C	1.213505	3.662347	-0.999730
C	2.717350	3.413149	0.913750
C	2.711279	-1.538133	2.993168
C	4.596426	-1.424976	1.434070
C	5.291714	0.142149	-1.968018
C	5.304185	-0.796069	0.294044
C	-1.294837	2.131058	-3.438133
C	0.596844	3.150002	-2.261517
C	-4.597792	2.256774	0.228344
C	-3.304855	2.265221	2.308824
C	0.227641	1.313038	3.367252
C	-2.156814	1.793006	3.114107
C	0.899843	4.949767	-0.538405
C	2.385170	4.695164	1.348615
H	3.436860	2.803409	1.448659
C	3.299659	-2.622888	3.643696
H	1.768834	-1.122692	3.333360
C	5.161141	-2.521569	2.108724
C	6.671151	0.334148	-1.904166
H	4.720341	0.407936	-2.850995
C	6.694108	-0.587361	0.330443
C	-0.631728	2.296826	-4.654424
H	-2.282611	1.684039	-3.377930
C	1.241453	3.300278	-3.498371
C	-5.497284	3.168829	0.780748
H	-4.719777	1.858134	-0.771119
C	-4.228505	3.184625	2.836769
C	0.041821	1.041161	4.723182
H	1.212313	1.284192	2.916209
C	-2.314552	1.500782	4.480964
C	1.473262	5.464049	0.623248
H	0.196052	5.548297	-1.109172
H	2.846792	5.093223	2.247395
C	4.527543	-3.115575	3.198110
H	2.800815	-3.075986	4.495170
H	6.107837	-2.917613	1.753969
C	7.371617	-0.030407	-0.751645
H	7.194459	0.768722	-2.750405
H	7.239597	-0.852361	1.231251
C	0.638498	2.877629	-4.683825
H	-1.110560	1.978592	-5.575870
H	2.221944	3.766972	-3.521867
C	-5.312660	3.631965	2.085739
H	-6.338206	3.518166	0.188960
H	-4.071463	3.566920	3.840963
C	-1.236791	1.125443	5.279687
H	0.895313	0.777079	5.341203
H	-3.308218	1.568867	4.913704
H	1.214867	6.464839	0.955643
H	4.990632	-3.962466	3.695202
H	8.444612	0.124513	-0.693150
H	1.154760	3.013817	-5.629446
H	-6.006061	4.348934	2.514975
H	-1.392797	0.911408	6.332808
H	0.400455	-0.017262	-2.065045
C	-0.929920	-3.504204	0.405392
C	-1.671924	-4.630297	0.801349
C	-0.760042	-2.474026	1.351225
C	-2.225739	-4.711146	2.080009
H	-1.811419	-5.459664	0.117224
C	-1.339480	-2.528310	2.614648
H	-0.084031	-1.618550	1.166613
C	-2.076324	-3.658075	2.983552
H	-2.778179	-5.600867	2.368031
H	-1.202073	-1.706010	3.310132
H	-2.518901	-3.718975	3.973326
C	-0.514645	-4.547700	-1.898702
H	-0.000485	-5.421292	-1.473204
H	-1.565425	-4.812482	-2.044072
H	-0.059924	-4.345507	-2.867444

IRC Reactant from TS-III-L-8

B3LYP/6-31G(d),SDD(Ir) Energy = -3451.006693
B3LYP/def2-TZVPP,SDD(Ir)/IEFPCM(dioxane) Energy = -3452.179366

B3LYP/def2-TZVPP,SDD(Ir)/IEFPCM(dioxane)//B3LYP/6-31G(d),SDD(Ir) Free Energy (Quasiharmonic) = -3451.46662
Number of Imaginary Frequencies = 0

Frequencies (Top 3 out of 315)

1. 11.0629 cm^{-1}
2. 15.9681 cm^{-1}
3. 19.6842 cm^{-1}

B3LYP/6-31G(d),SDD(Ir) Molecular Geometry in Cartesian Coordinates

C	-0.525103	2.79987	-1.163642
C	0.443735	3.219875	-2.059923
C	2.206603	0.970409	-1.156052
C	3.03909	1.45523	-0.158931
O	2.936451	0.746723	-2.27442
C	4.371501	1.505356	-0.714265
C	4.250367	1.071363	-1.992589
H	4.948505	0.94003	-2.804882
H	5.295707	1.806989	-0.245617
H	-0.54004	3.182642	-0.147534
H	-1.485749	2.514553	-1.573191
C	2.383299	1.657302	1.12307
O	1.144928	1.337512	1.214954
N	3.006749	2.139102	2.217528
C	2.309736	2.132884	3.503614
H	3.050656	2.051782	4.302881
H	1.728012	3.052197	3.648698
H	1.637876	1.276495	3.551808
C	4.336117	2.745067	2.174614
H	4.420038	3.453734	3.001784
H	5.131611	1.996763	2.278575
H	4.474225	3.299006	1.244928
Ir	0.254604	0.549314	-0.720426
P	-1.997251	0.157628	-0.094585
P	0.905698	-1.566522	-0.34286
O	-2.730393	-1.216956	-0.549106
O	-2.423388	0.223832	1.497063
O	-3.036936	1.212875	-0.770457
O	0.578542	-2.706189	-1.439946
O	2.508551	-1.706438	-0.157764
O	0.333466	-2.325931	0.984653
C	-2.647786	-2.536922	-0.096892
C	-2.68348	1.452327	2.123986
C	-4.40674	1.207957	-0.449471
C	-0.308861	-2.676534	-2.520654
C	3.110445	-2.931998	0.181222
C	0.905435	-2.207784	2.256759
C	-2.140677	-3.492995	-0.986095
C	-3.163154	-2.883658	1.150136
C	-1.790095	1.886794	3.099546
C	-3.86618	2.153022	1.827974
C	-5.300603	0.803164	-1.434836
C	-4.827509	1.688283	0.799902
C	0.190247	-2.330364	-3.774207
C	-1.63017	-3.100951	-2.332832
C	3.894061	-3.55036	-0.787339
C	2.98409	-3.433903	1.484385
C	0.1189	-1.640636	3.256942
C	2.171754	-2.756822	2.52245
C	-2.147706	-4.832722	-0.574685
C	-3.149975	-4.223484	1.537506
H	-3.575153	-2.113484	1.791965
C	-2.071044	3.047684	3.819591
C	-4.117633	3.322517	2.566793
C	-6.669895	0.873377	-1.181778
C	-6.213959	1.743914	1.026697
C	-0.652857	-2.395532	-4.883716
H	1.227876	-2.026959	-3.868726
C	-2.454592	-3.161869	-3.465505
C	4.58199	-4.7184	-0.463242
H	3.963673	-3.104463	-1.7738
C	3.685934	-4.61684	1.779161
C	0.5991	-1.591903	4.565009
H	-0.863585	-1.263701	2.997056
C	2.633051	-2.679538	3.849653
C	-2.642545	-5.198656	0.67689
H	-1.75844	-5.588488	-1.250581
H	-3.549017	-4.503612	2.507963
C	-3.238414	3.76603	3.552298
H	-1.38135	3.387053	4.58701
C	-7.125367	1.343755	0.052228
H	-7.375761	0.559037	-1.944517
C	-1.976389	-2.812565	-4.729001
H	-0.271053	-2.131184	-5.86535
H	-3.481631	-3.493453	-3.343553
C	4.47463	-5.252149	0.823311
H	5.195193	-5.209006	-1.212978
H	3.588367	-5.048489	2.770623
C	1.864023	-2.107218	4.859675
H	-0.016251	-1.160788	5.349451
H	3.618118	-3.073672	4.080516
H	-2.639147	-6.242642	0.975124
H	-2.63386	-2.873955	-5.591023
H	4.999084	-6.167421	1.080241
H	2.248847	-2.06899	5.874397
H	-0.16532	0.127086	-2.181429
H	-0.891085	1.308785	3.278931
H	-4.915024	0.44768	-2.38444
H	-8.190208	1.393039	0.2583
H	-6.572071	2.091242	1.99123
H	-5.013797	3.894563	2.346188
H	-3.462276	4.67311	4.105562
C	0.303149	2.838688	-3.511827
H	0.290238	3.737183	-4.139794
H	1.146138	2.219999	-3.838278
H	-0.61329	2.270322	-3.680917
C	1.562868	4.101693	-1.68094
C	2.66156	4.284053	-2.545721
C	1.535944	4.841993	-0.479403
C	3.698259	5.15277	-2.216145
H	2.71541	3.733727	-3.477849
C	2.569663	5.714345	-0.153518
H	0.685749	4.763081	0.188671
C	3.656671	5.871359	-1.019457
H	4.535733	5.274234	-2.89664
H	2.518347	6.289886	0.766272
H	4.457576	6.561516	-0.769547

IRC Product from TS-III-L-8

B3LYP/6-31G(d),SDD(Ir) Energy = -3451.002941
B3LYP/def2-TZVPP,SDD(Ir)/IEFPCM(dioxane) Energy = - -3452.174491
B3LYP/def2-TZVPP,SDD(Ir)/IEFPCM(dioxane)//B3LYP/6-31G(d),SDD(Ir) Free Energy (Quasiharmonic) = -3451.458969
Number of Imaginary Frequencies = 0

Frequencies (Top 3 out of 315)

1. 11.4078 cm^{-1}
2. 15.6156 cm^{-1}
3. 20.2731 cm^{-1}

B3LYP/6-31G(d),SDD(Ir) Molecular Geometry in Cartesian Coordinates

C	-2.349522	0.990383	-0.823753
C	-2.259238	2.248913	-1.720839
C	-0.795877	2.670935	-1.629313
C	0.03916	3.006091	-0.551628
O	-0.177503	3.073409	-2.777353
C	1.199582	3.650637	-1.113412
C	1.013548	3.652802	-2.454731
H	1.612292	3.987757	-3.288286
H	2.063288	4.007961	-0.573794
H	-2.706193	1.225921	0.183724
H	-3.011678	0.246517	-1.263855

C	-0.229996	2.796774	0.903328
O	-0.424047	1.615758	1.301419
N	-0.194129	3.826212	1.759402
C	-0.307651	3.557598	3.196056
H	0.236156	4.336063	3.737245
H	-1.357761	3.569304	3.510509
H	0.12526	2.583518	3.422353
C	-0.241181	5.234334	1.361955
H	-1.111022	5.708773	1.828553
H	0.664784	5.753221	1.693549
H	-0.339884	5.326705	0.282111
Ir	-0.32513	0.364701	-0.606692
P	-1.034437	-1.665366	-0.090443
P	2.010134	-0.014317	-0.367184
O	-0.225032	-2.965945	-0.600676
O	-1.215257	-1.990737	1.504443
O	-2.490261	-1.998752	-0.707457
O	2.858516	-0.814063	-1.50561
O	2.866737	1.371371	-0.21879
O	2.555072	-0.855683	0.936899
C	0.981811	-3.538406	-0.174815
C	-2.375987	-1.619152	2.206457
C	-3.16791	-3.186035	-0.368195
C	2.321615	-1.526641	-2.581398
C	4.231348	1.390496	0.115477
C	2.651662	-0.269824	2.200975
C	2.055696	-3.529144	-1.07259
C	1.047208	-4.18339	1.058395
C	-2.244221	-0.658292	3.204732
C	-3.588592	-2.283585	1.95579
C	-3.328353	-4.141023	-1.366032
C	-3.728937	-3.325212	0.910255
C	2.259894	-0.90113	-3.824982
C	1.949349	-2.865294	-2.405443
C	5.130506	1.805941	-0.861899
C	4.632086	1.094889	1.427406
C	1.787204	-0.721924	3.195842
C	3.668039	0.66503	2.4675
C	3.233884	-4.179464	-0.679745
C	2.233403	-4.818665	1.42467
H	0.178355	-4.192232	1.706265
C	-3.348095	-0.337253	3.993771
C	-4.68389	-1.931453	2.764713
C	-4.072903	-5.288084	-1.095407
C	-4.469791	-4.495721	1.153672
C	1.801645	-1.618866	-4.92976
H	2.583642	0.130802	-3.916907
C	1.493677	-3.566072	-3.531364
C	6.480194	1.938358	-0.538146
H	4.763476	2.024573	-1.859268
C	5.999263	1.235549	1.723578
C	1.913367	-0.228934	4.494449
H	1.040467	-1.465508	2.940864
C	3.76545	1.149724	3.784075
C	3.327543	-4.815739	0.557332
H	4.07988	-4.181649	-1.360583
H	2.295377	-5.324858	2.383457
C	-4.570201	-0.97583	3.771652
H	-3.252601	0.408061	4.777841
C	-4.642065	-5.464685	0.168165
H	-4.204874	-6.039647	-1.86771
C	1.416611	-2.953019	-4.782236
H	1.754382	-1.138026	-5.902283
H	1.204806	-4.606655	-3.415464
C	6.913307	1.652848	0.758678
H	7.188283	2.258323	-1.296536
H	6.344677	0.990312	2.723411
C	2.902361	0.713877	4.787316
H	1.24772	-0.587775	5.274218
H	4.53101	1.885268	4.013155
H	4.24952	-5.314882	0.839511
H	1.066029	-3.518303	-5.640433
H	7.964032	1.744836	1.016102
H	3.006757	1.103259	5.795692
H	-0.306974	-0.209106	-2.073264
H	-1.28214	-0.179439	3.348522
H	-2.879281	-3.969512	-2.338468
H	-5.215572	-6.359913	0.388129
H	-4.898849	-4.646489	2.139678
H	-5.640723	-2.410772	2.581631
H	-5.435958	-0.726702	4.377706
C	-2.602183	1.864963	-3.181264
H	-3.651387	1.55832	-3.219621
H	-2.468949	2.706506	-3.866029
H	-1.981978	1.029668	-3.522219
C	-3.173417	3.417095	-1.297512
C	-2.860215	4.739555	-1.650275
C	-4.376061	3.17919	-0.619065
C	-3.71255	5.793764	-1.318197
H	-1.950601	4.95358	-2.206607
C	-5.232983	4.232447	-0.290181
H	-4.656267	2.16489	-0.35218
C	-4.9031	5.543991	-0.632776
H	-3.450261	6.808709	-1.604798
H	-6.161762	4.023205	0.233528
H	-5.56974	6.362293	-0.376245

Copies of ^{1}H and ^{13}C NMR Spectra for Novel Compounds

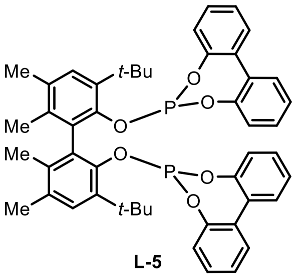

L-5

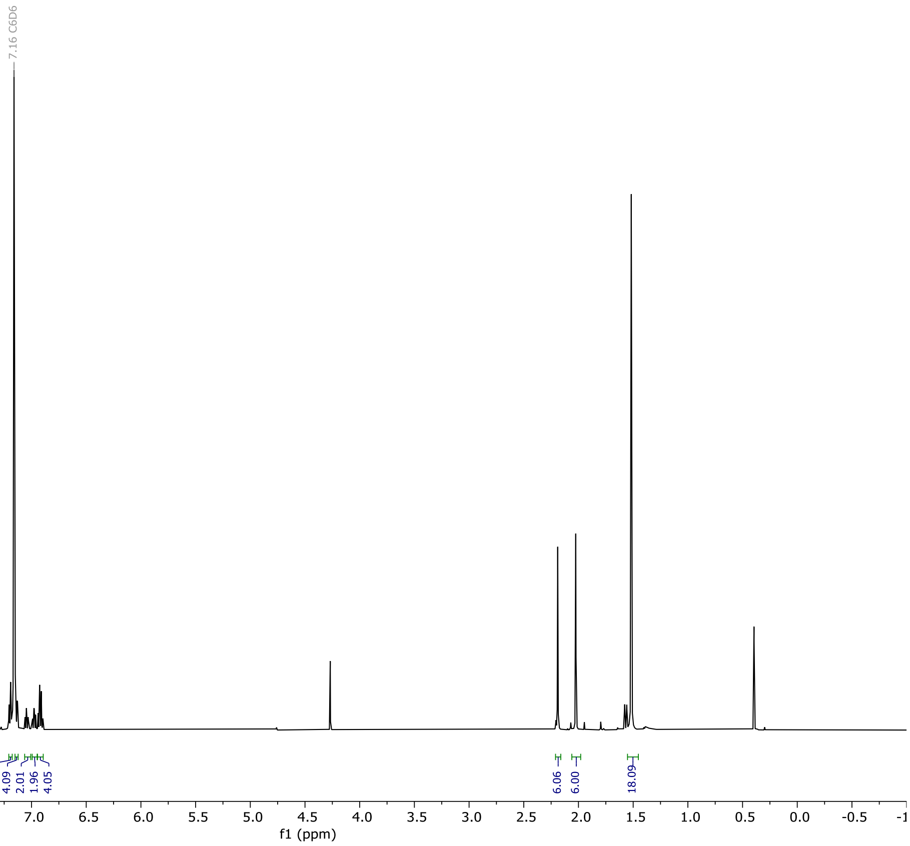

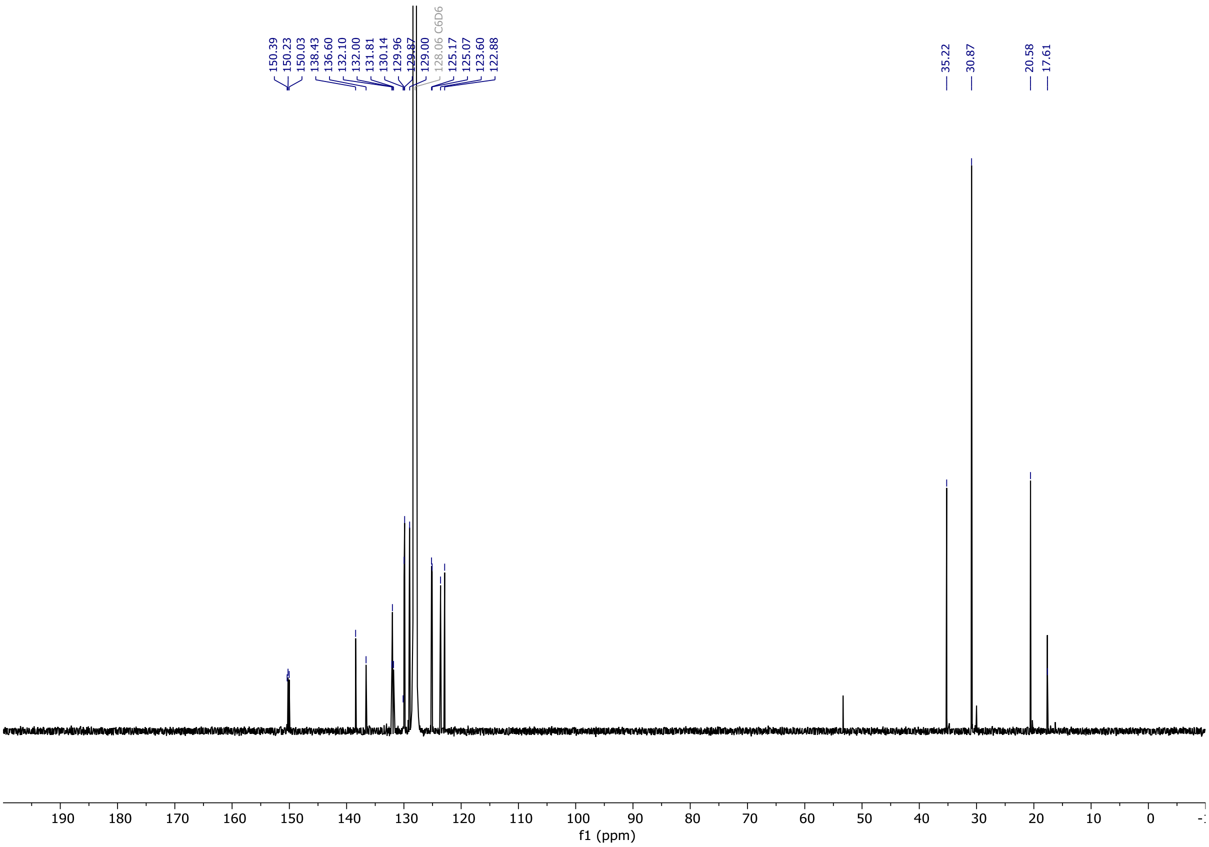

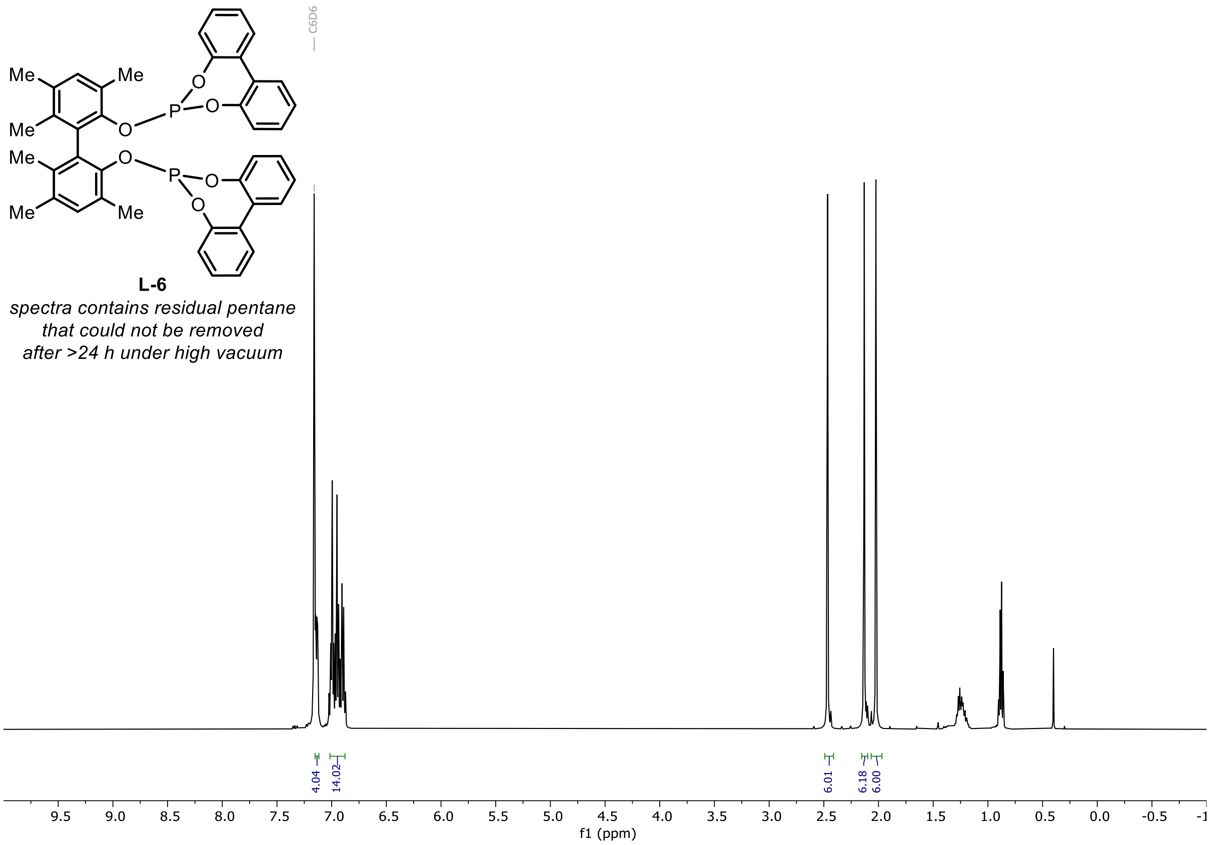

C6D6
Me
Me
Me
Me
Me
Me
O
P
O
O
O
P
O
O
L-6
spectra contains residual pentane
that could not be removed
after >24 h under high vacuum
4.04
14.02
6.01
6.18
6.00
9.5
9.0
8.5
8.0
7.5
7.0
6.5
6.0
5.5
5.0
4.5
4.0
3.5
3.0
2.5
2.0
1.5
1.0
0.5
0.0
-0.5
f1 (ppm)

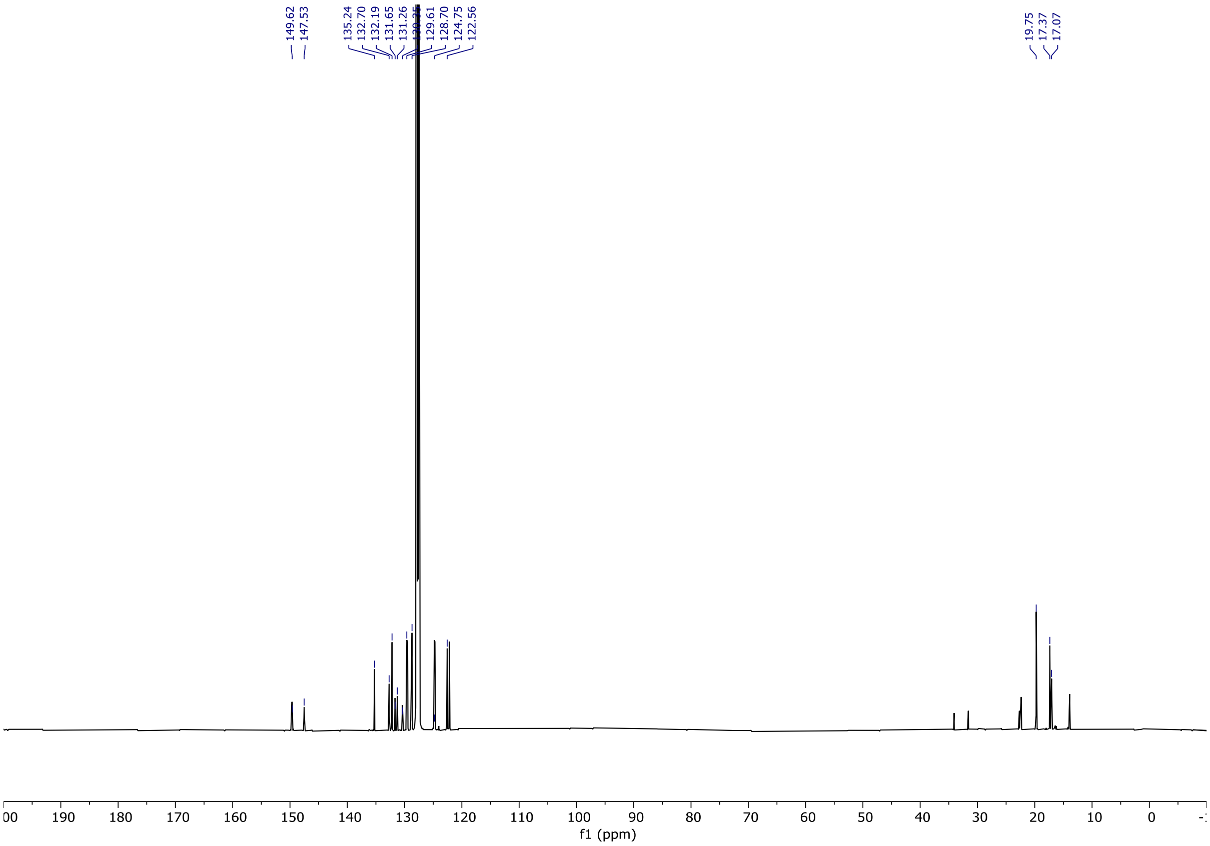

149.62
147.53
135.24
132.70
132.19
131.65
131.26
129.61
128.70
124.75
122.56
19.75
17.37
17.07
190
180
170
160
150
140
130
120
110
100
90
80
70
60
50
40
30
20
10
0
f1 (ppm)

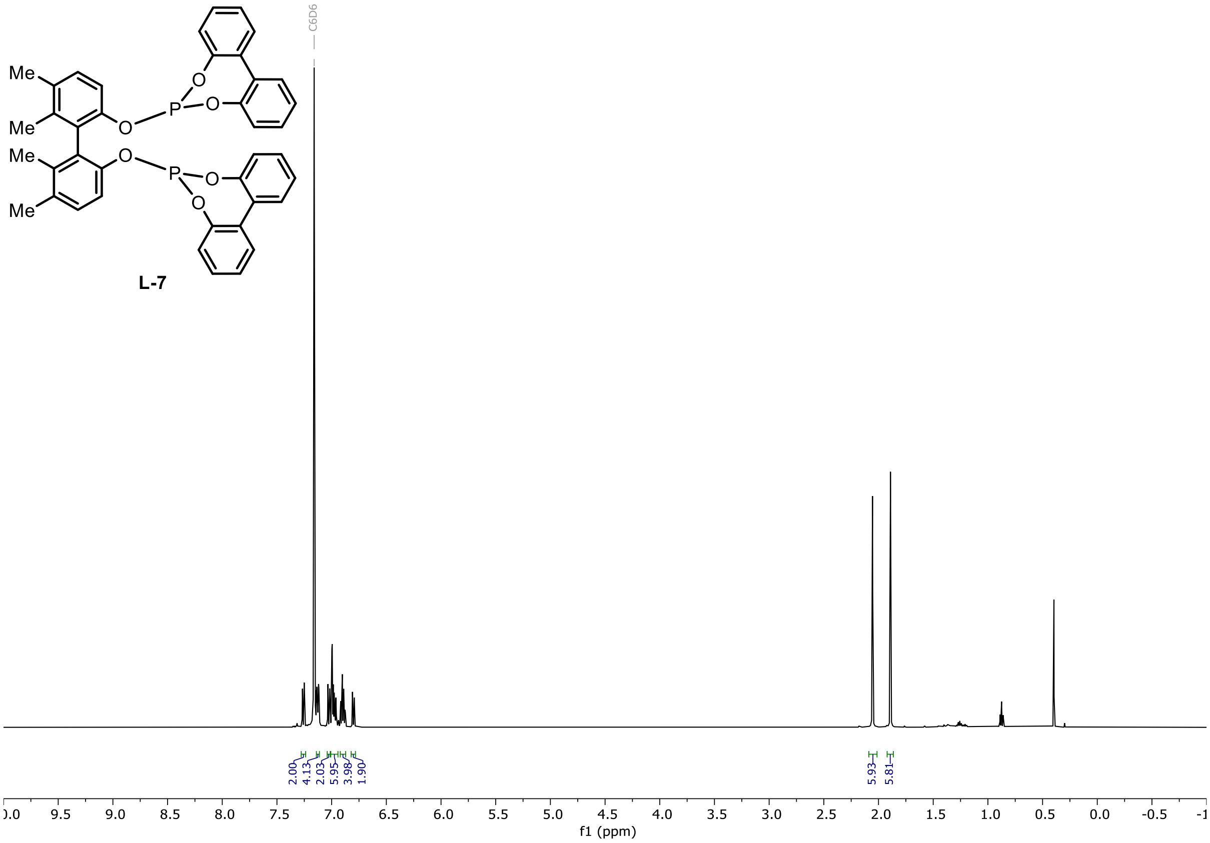
Me
Me
Me
Me
O
P
O
O
O
P
O
O
L-7
C6D6
2.00
4.13
2.03
5.95
3.98
1.90
5.93
5.81
f1 (ppm)

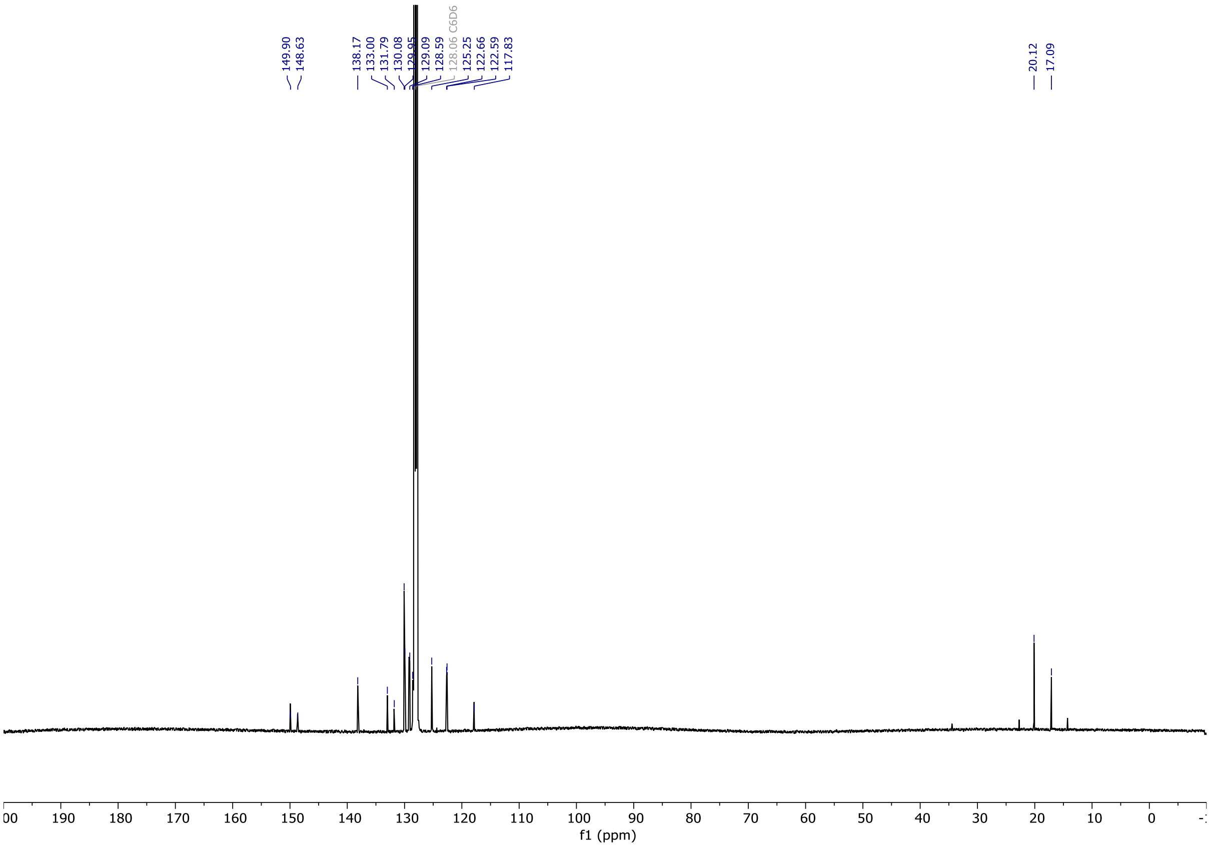
149.90
148.63
138.17
133.00
131.79
130.08
129.95
129.09
128.59
128.06 C6D6
125.25
122.66
122.59
117.83
20.12
17.09
f1 (ppm)

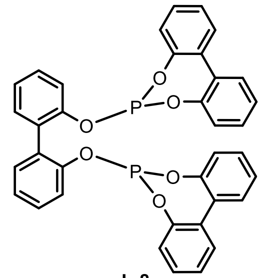

L-8

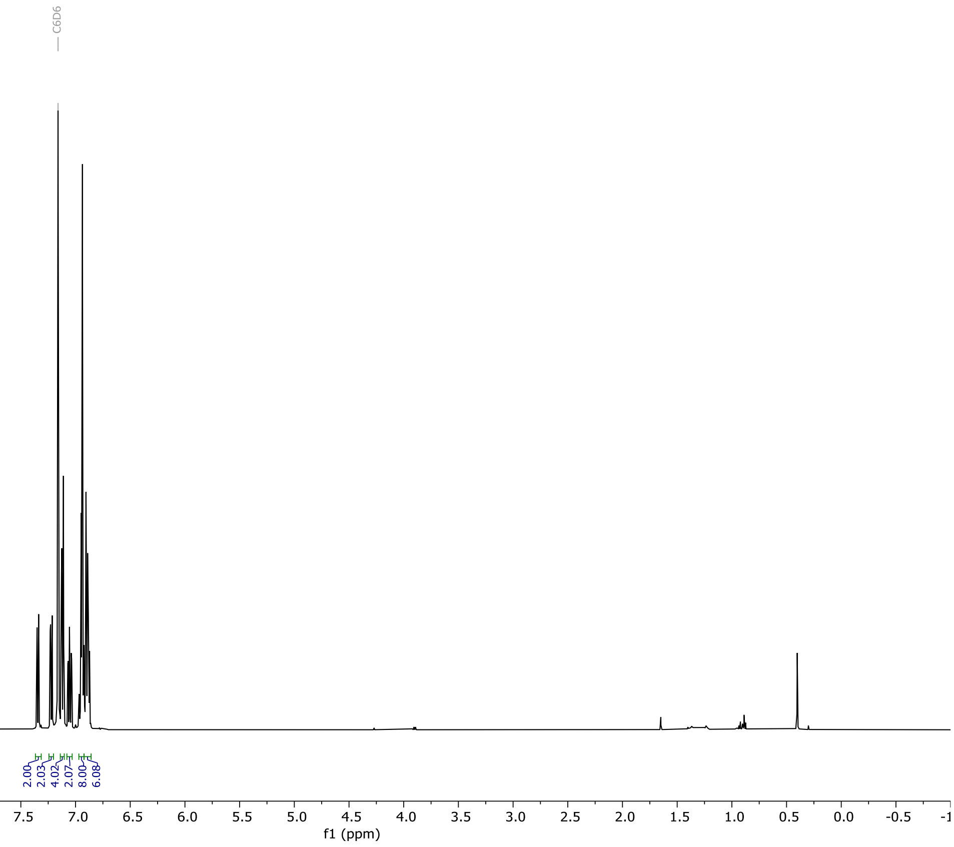
C6D6
2.00
2.03
4.02
2.07
8.00
6.08
9.5 9.0 8.5 8.0 7.5 7.0 6.5 6.0 5.5 5.0 4.5 4.0 3.5 3.0 2.5 2.0 1.5 1.0 0.5 0.0 -0.5
f1 (ppm)

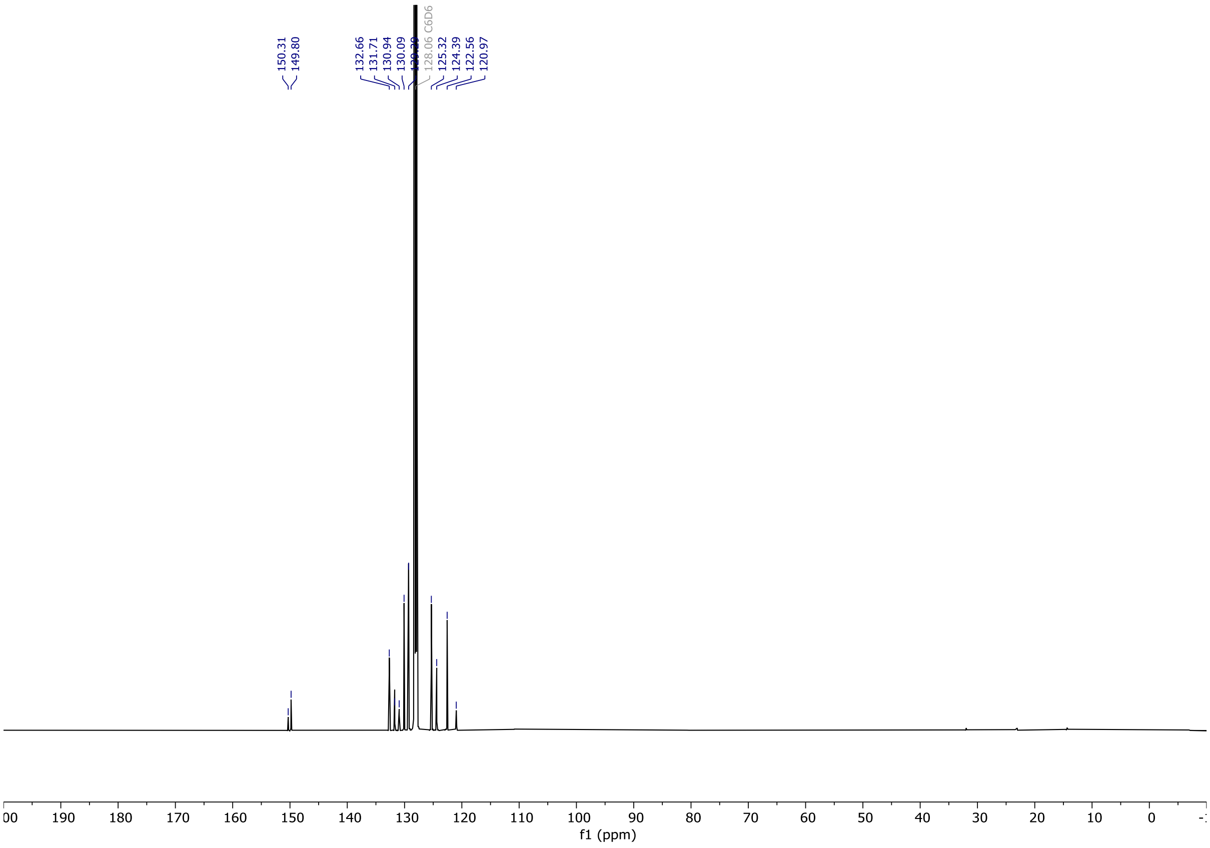
150.31
149.80
132.66
131.71
130.94
130.09
128.06 C6D6
125.32
124.39
122.56
120.97
190 180 170 160 150 140 130 120 110 100 90 80 70 60 50 40 30 20 10 0
f1 (ppm)

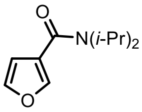
N(i-Pr)2
O

3a

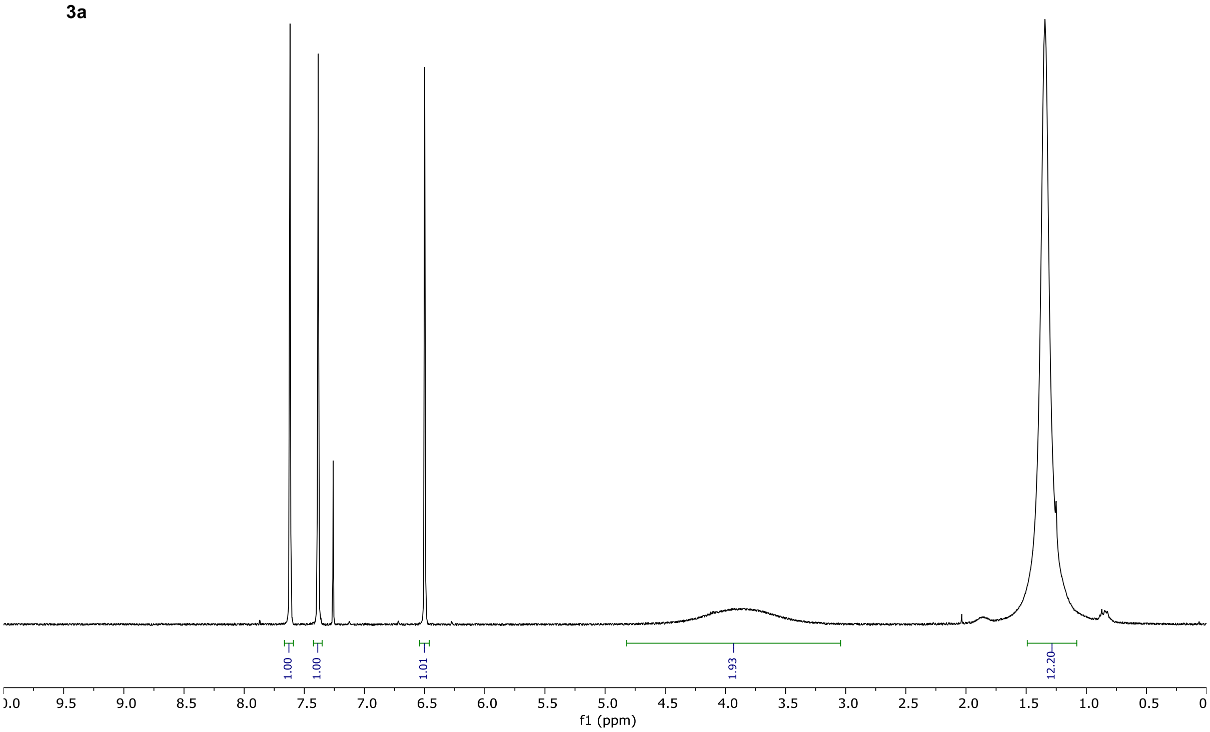
1.00
1.00
1.01
1.93
12.20
f1 (ppm)

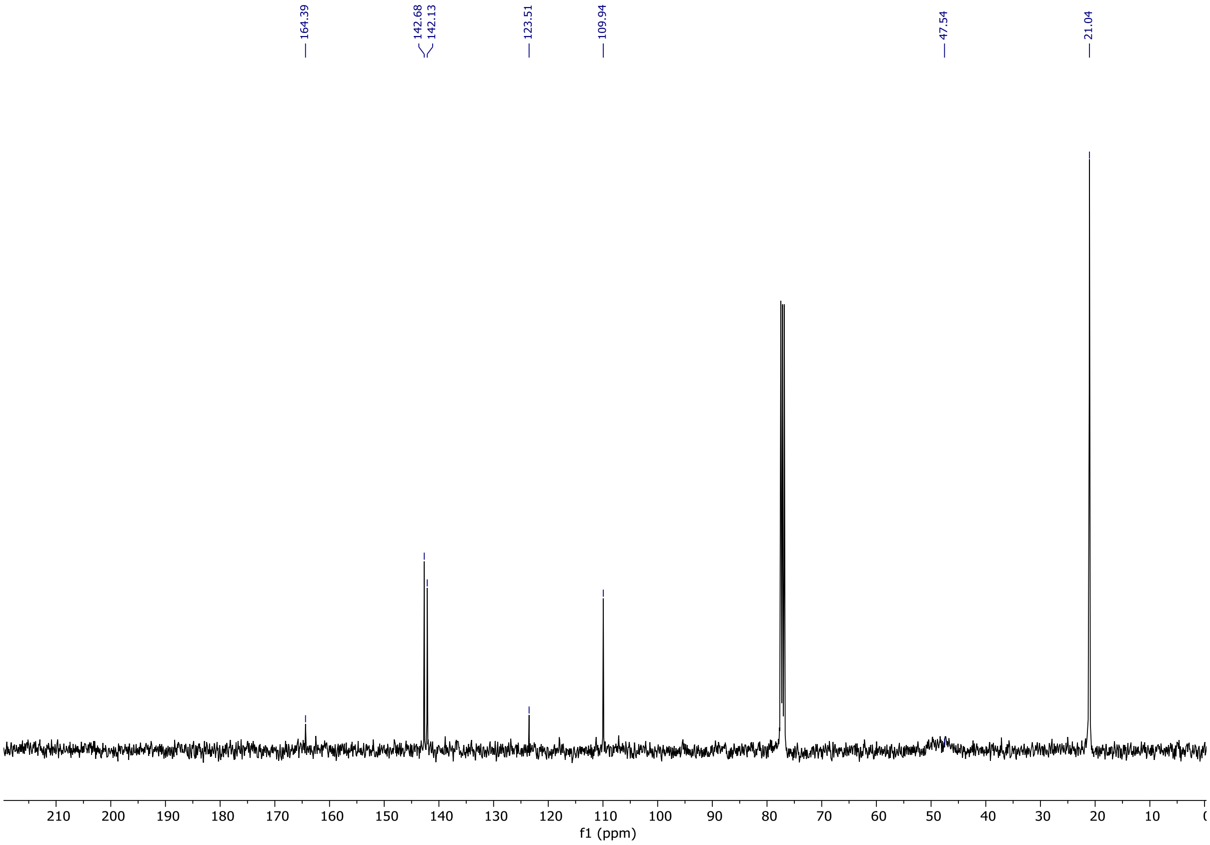
164.39
142.68
142.13
123.51
109.94
47.54
21.04
f1 (ppm)

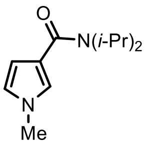
O
N(i-Pr)2
N
Me

5

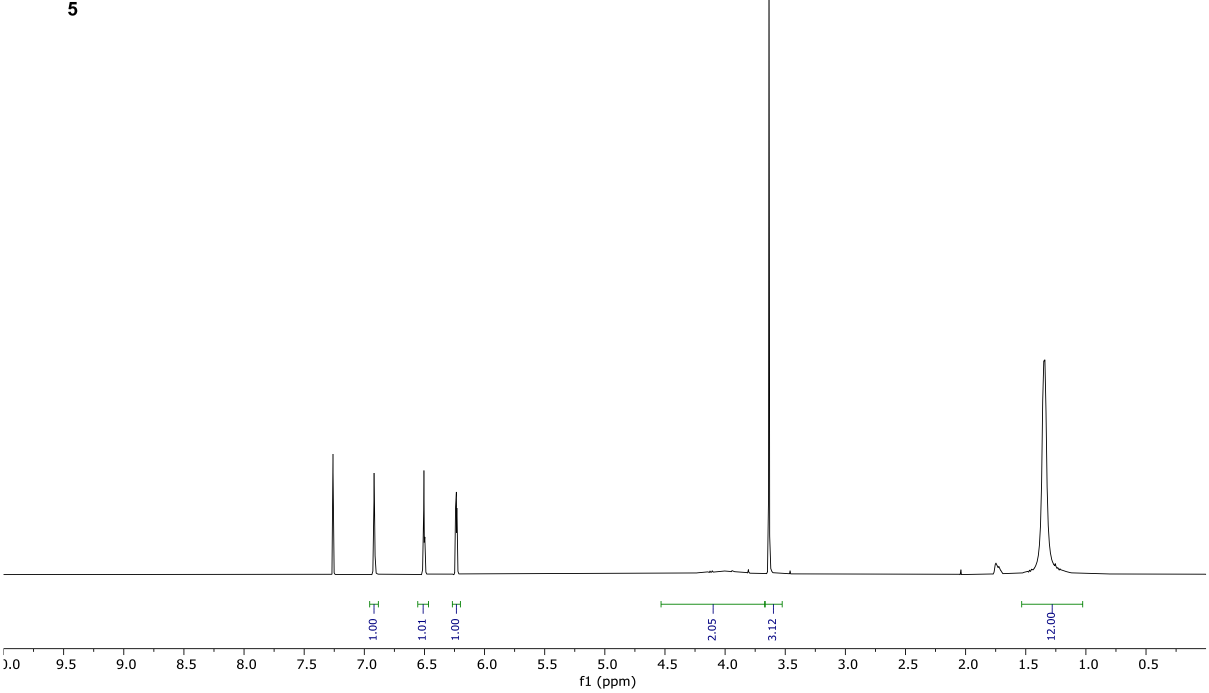
1.00
1.01
1.00
2.05
3.12
12.00
f1 (ppm)

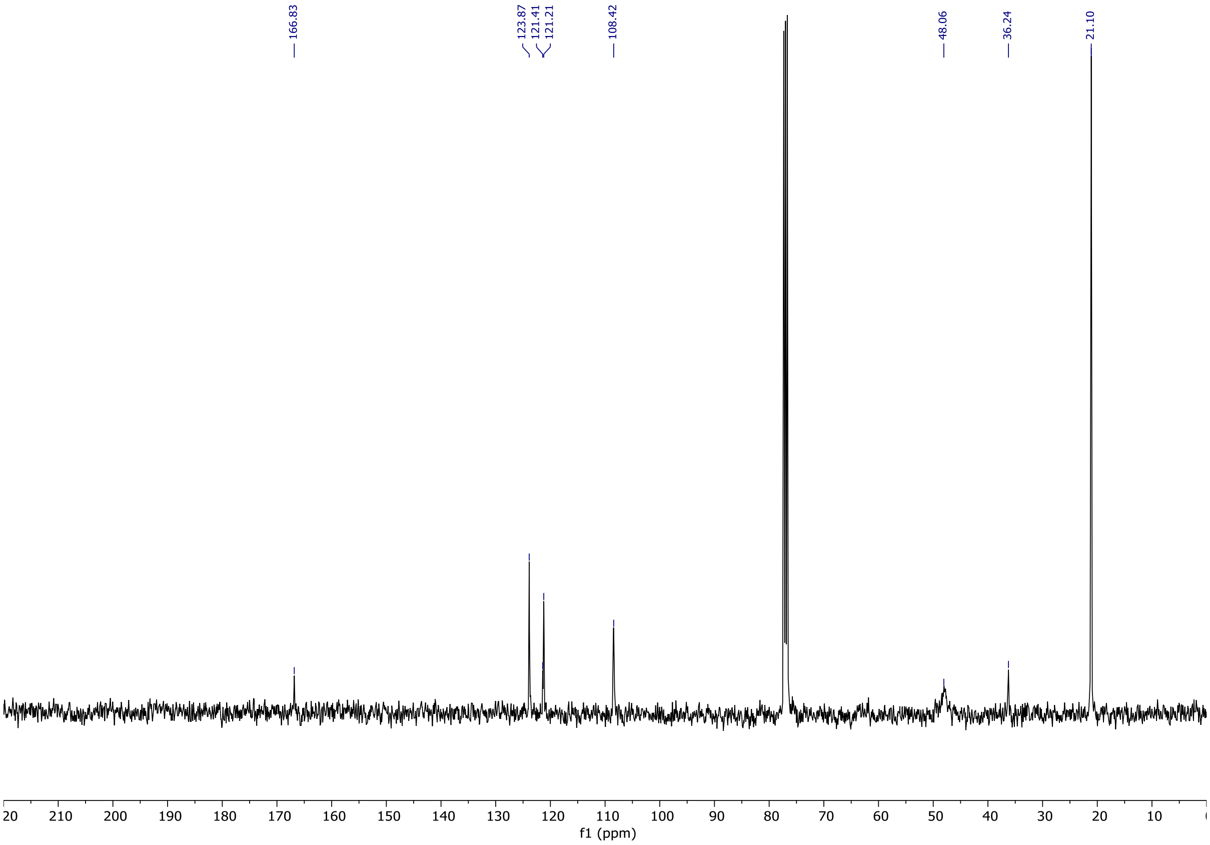
166.83
123.87
121.41
121.21
108.42
48.06
36.24
21.10
f1 (ppm)

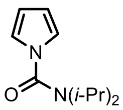
N
O
N(i-Pr)2

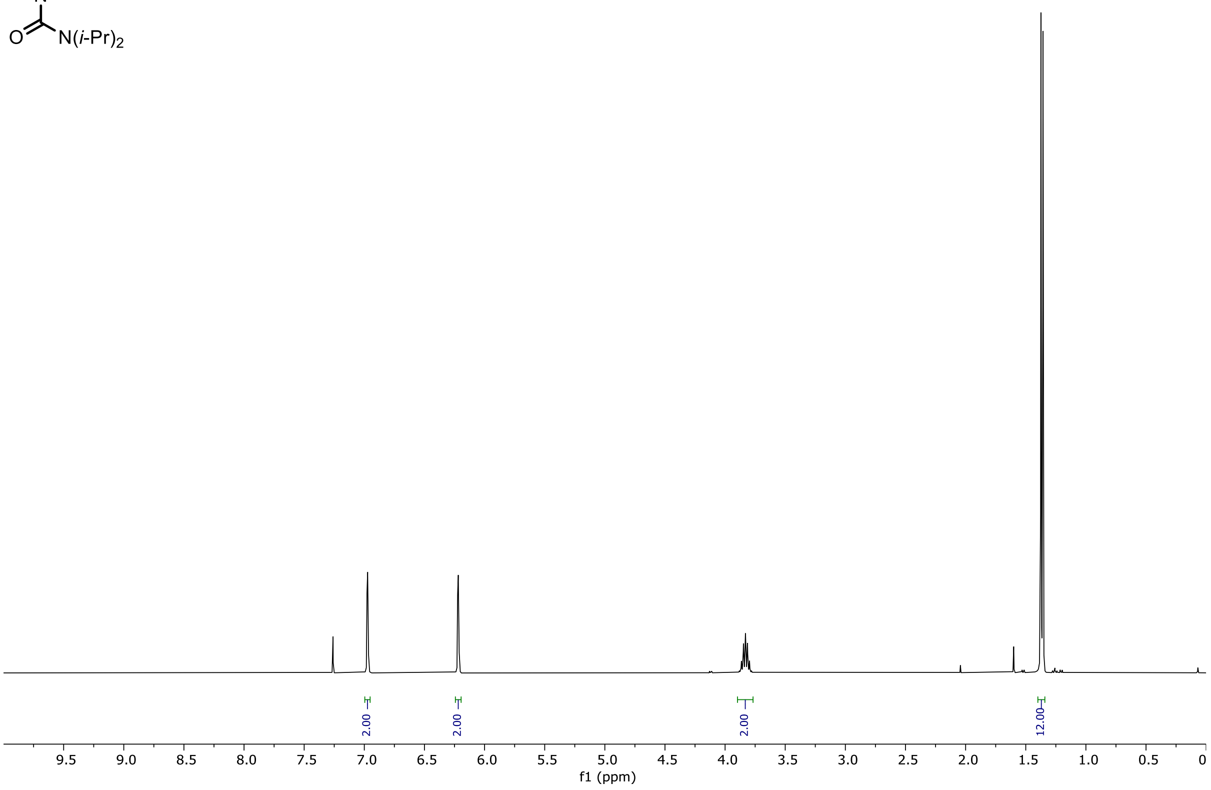
2.00
2.00
2.00
12.00
9.5
9.0
8.5
8.0
7.5
7.0
6.5
6.0
5.5
5.0
4.5
4.0
3.5
3.0
2.5
2.0
1.5
1.0
0.5
0
f1 (ppm)

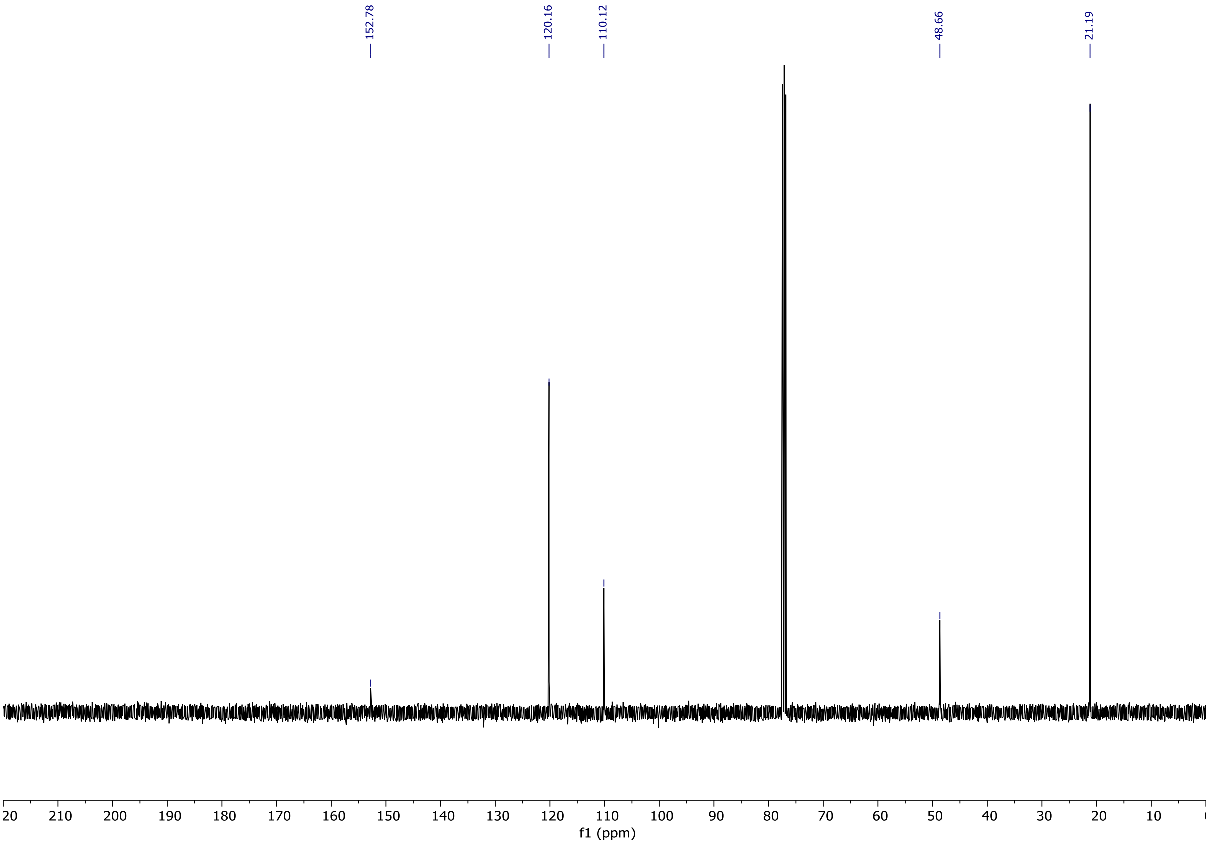
152.78
120.16
110.12
48.66
21.19
210
200
190
180
170
160
150
140
130
120
110
100
90
80
70
60
50
40
30
20
10
f1 (ppm)

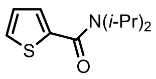
N(i-Pr)2
S
O

7

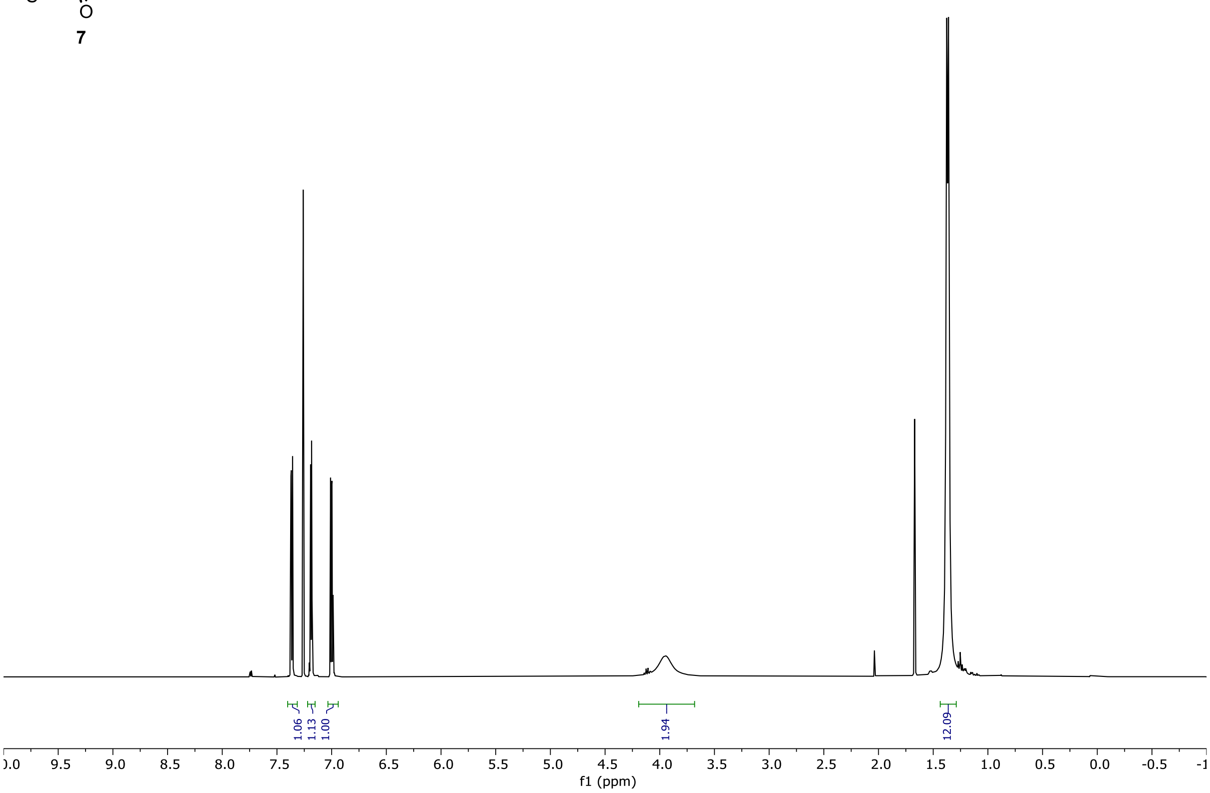
1.06
1.13
1.00
1.94
12.09
f1 (ppm)

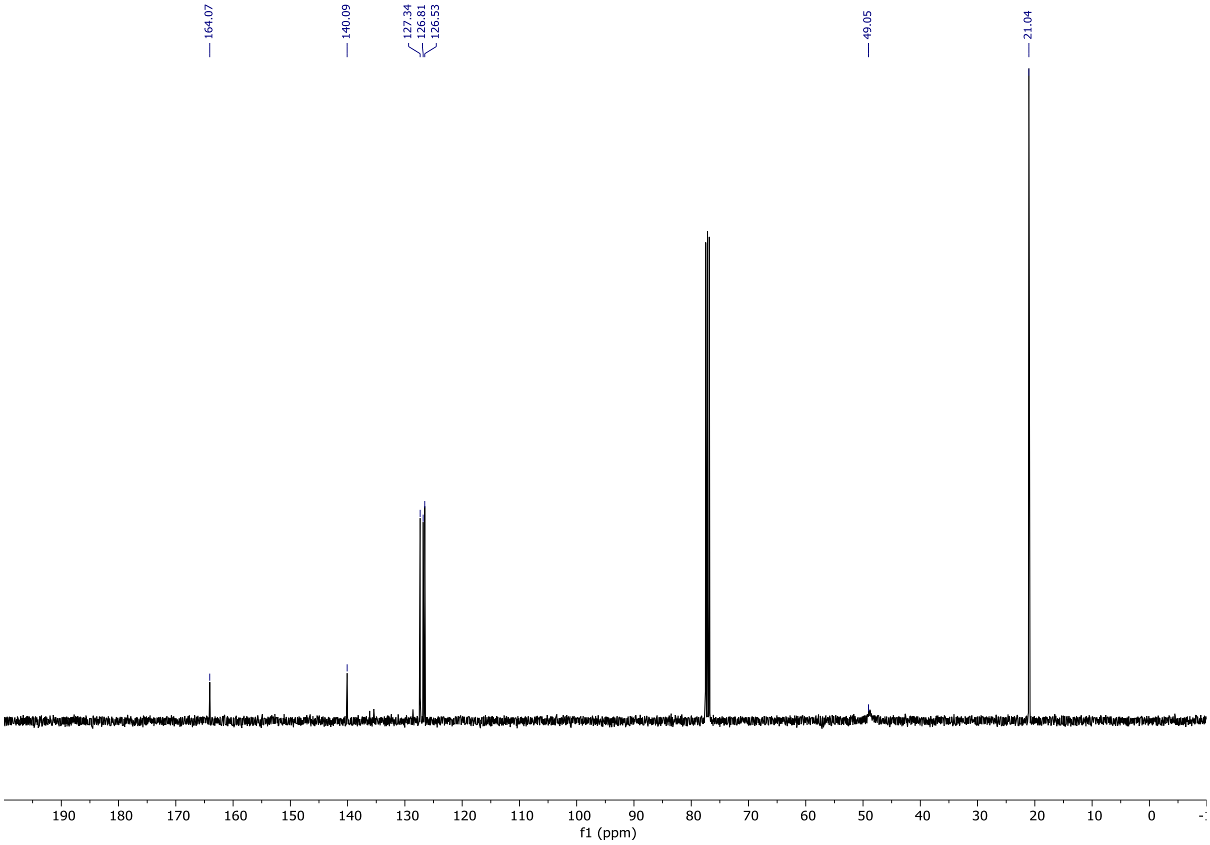
164.07
140.09
127.34
126.81
126.53
49.05
21.04
f1 (ppm)

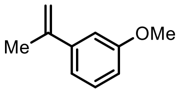
Me
OMe

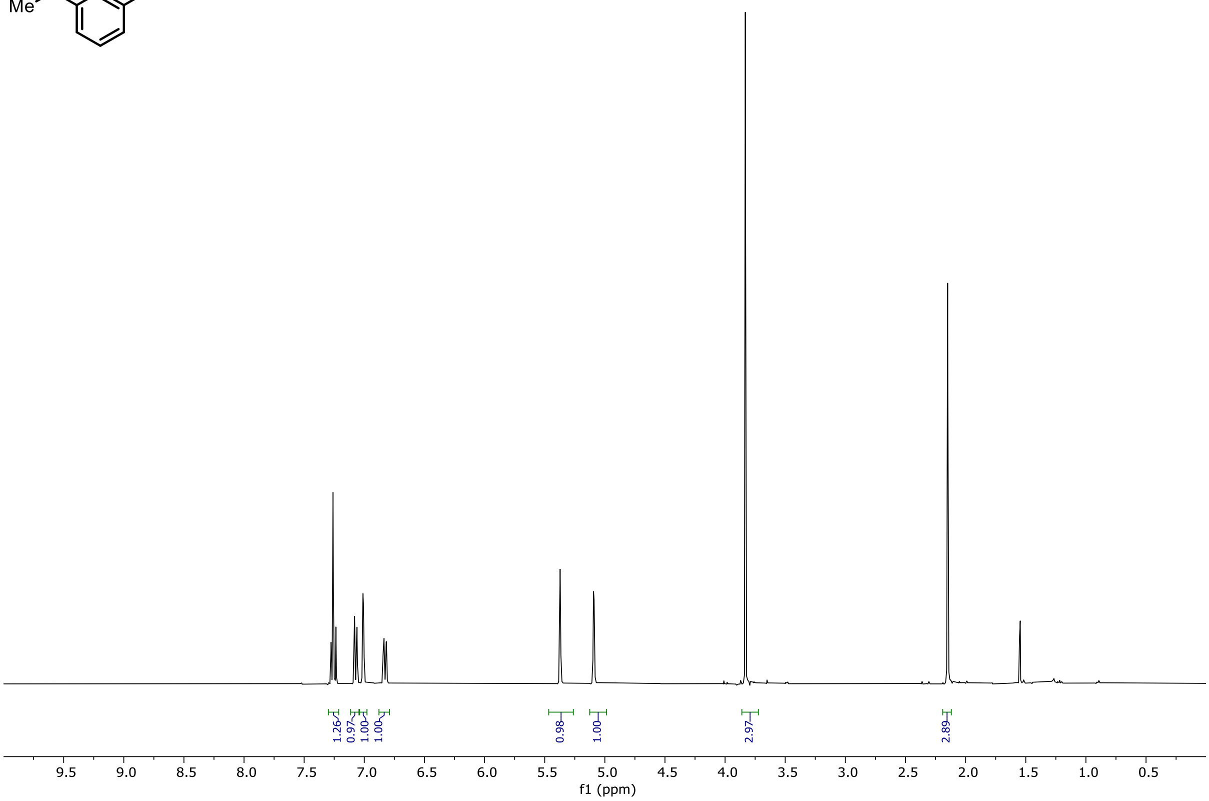
1.26
0.97
1.00
1.00
0.98
1.00
2.97
2.89
9.5 9.0 8.5 8.0 7.5 7.0 6.5 6.0 5.5 5.0 4.5 4.0 3.5 3.0 2.5 2.0 1.5 1.0 0.5
f1 (ppm)

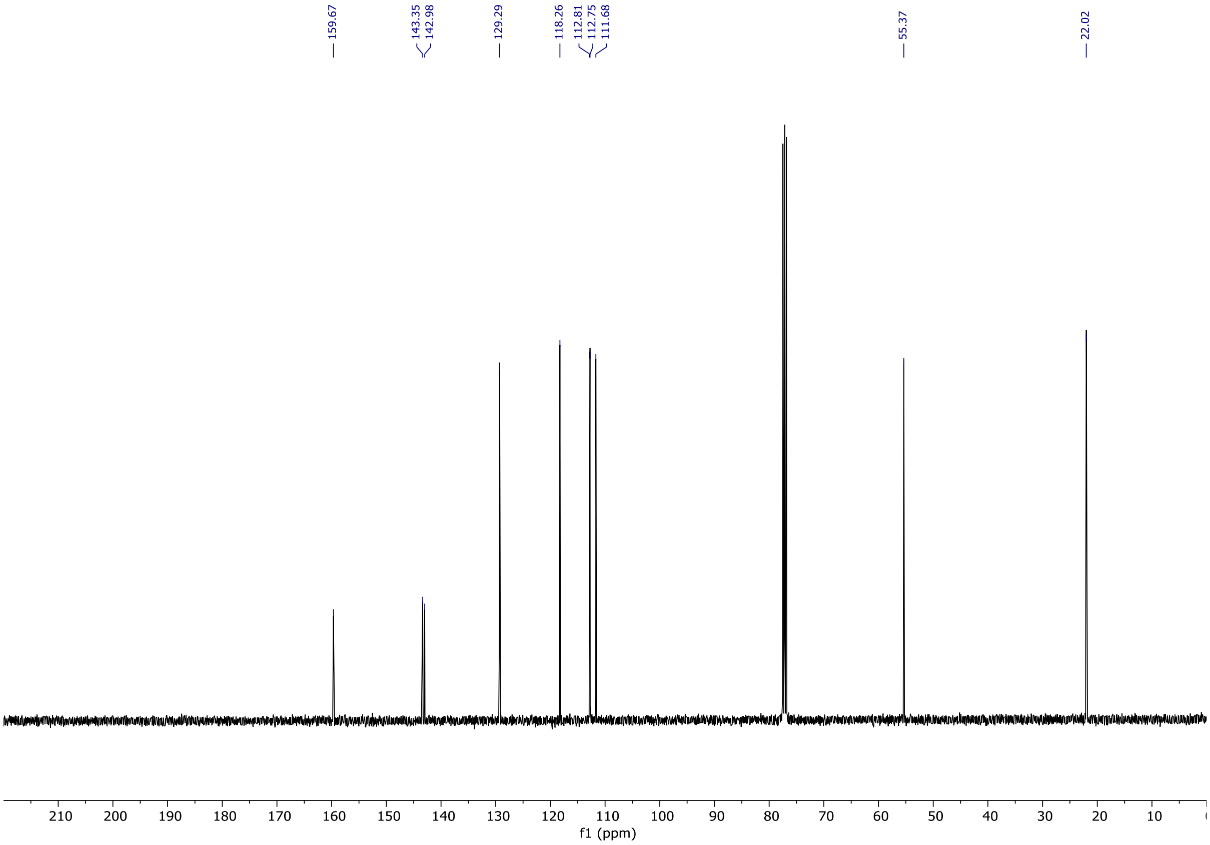
159.67
143.35
142.98
129.29
118.26
112.81
112.75
111.68
55.37
22.02
210 200 190 180 170 160 150 140 130 120 110 100 90 80 70 60 50 40 30 20 10
f1 (ppm)

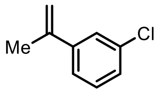
Me
Cl

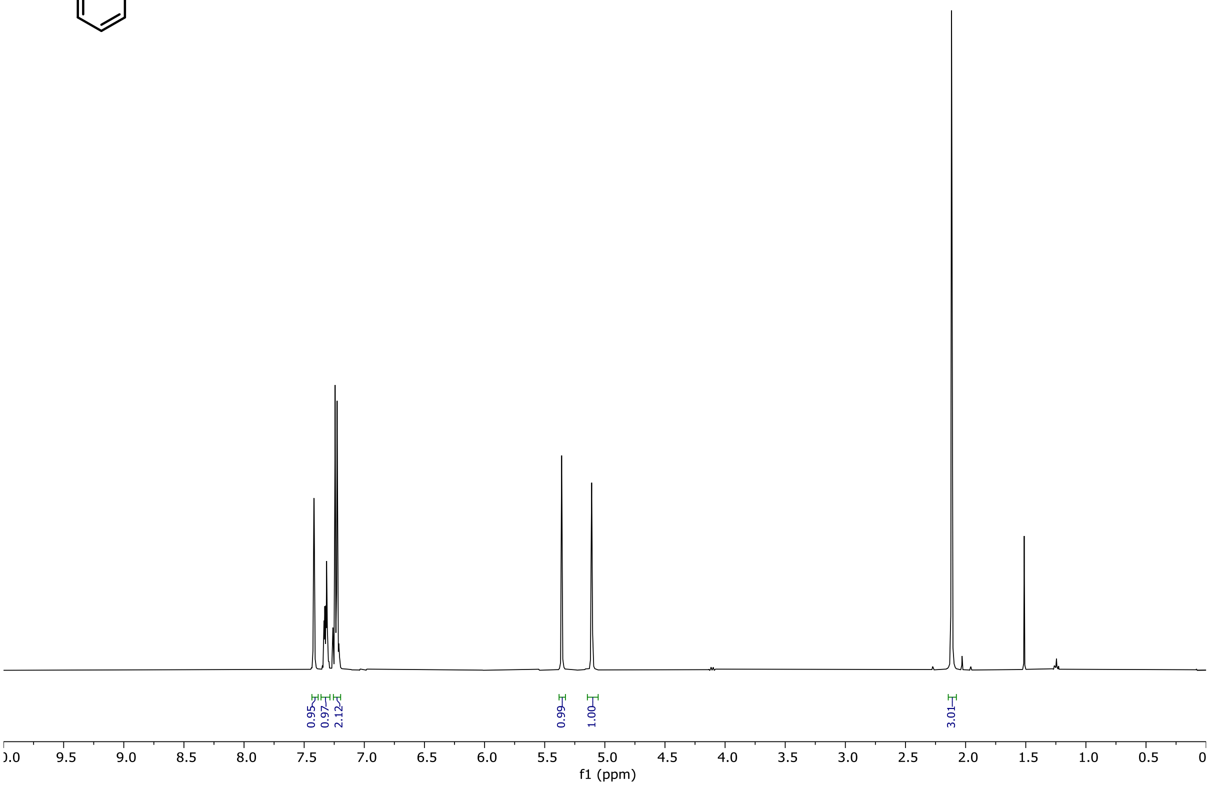
0.95
0.97
2.12
0.99
1.00
3.01
f1 (ppm)

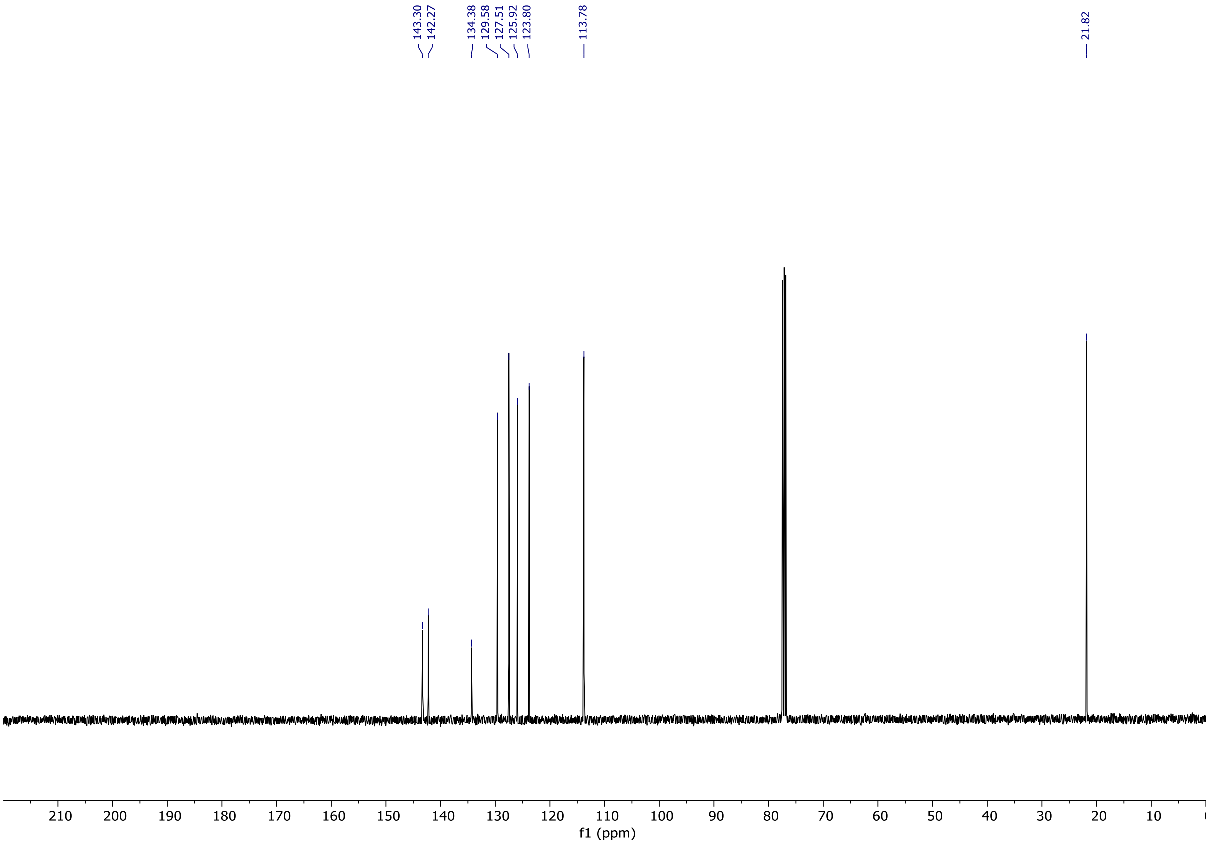
143.30
142.27
134.38
129.58
127.51
125.92
123.80
113.78
21.82
f1 (ppm)

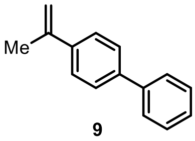
Me

9

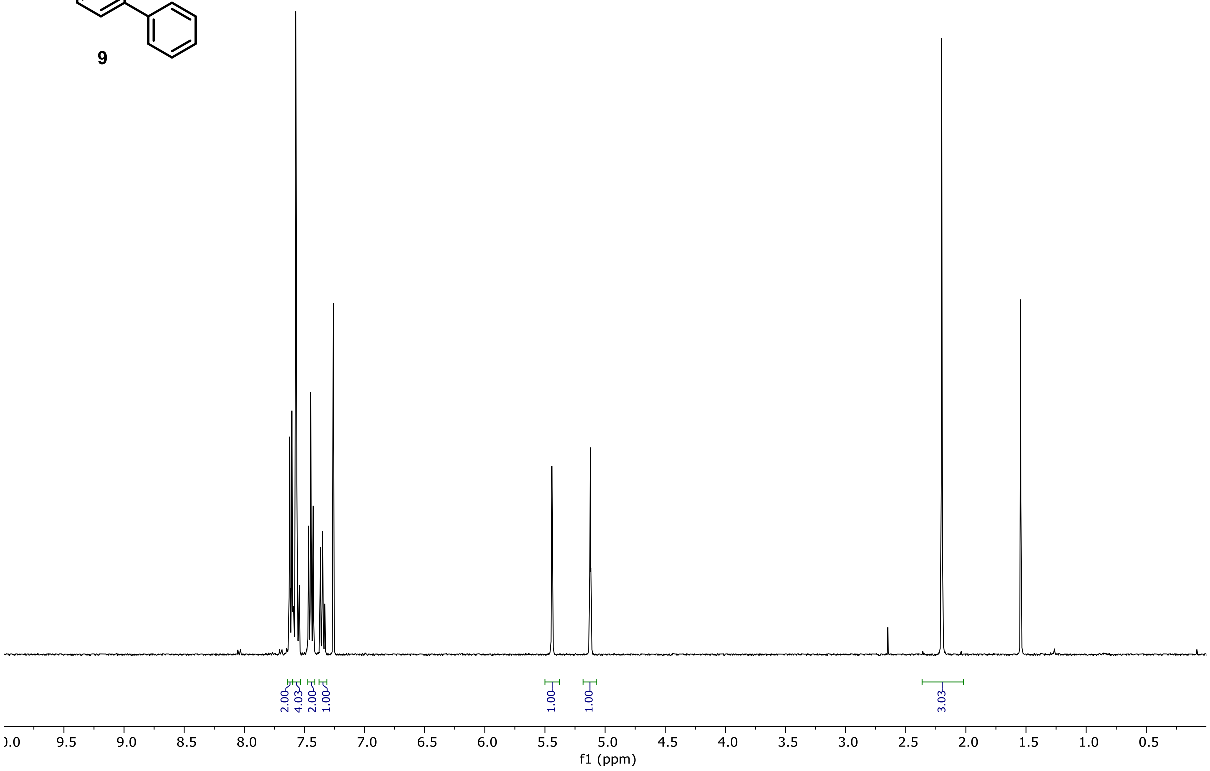
2.00
4.03
2.00
1.00
1.00
1.00
3.03
f1 (ppm)

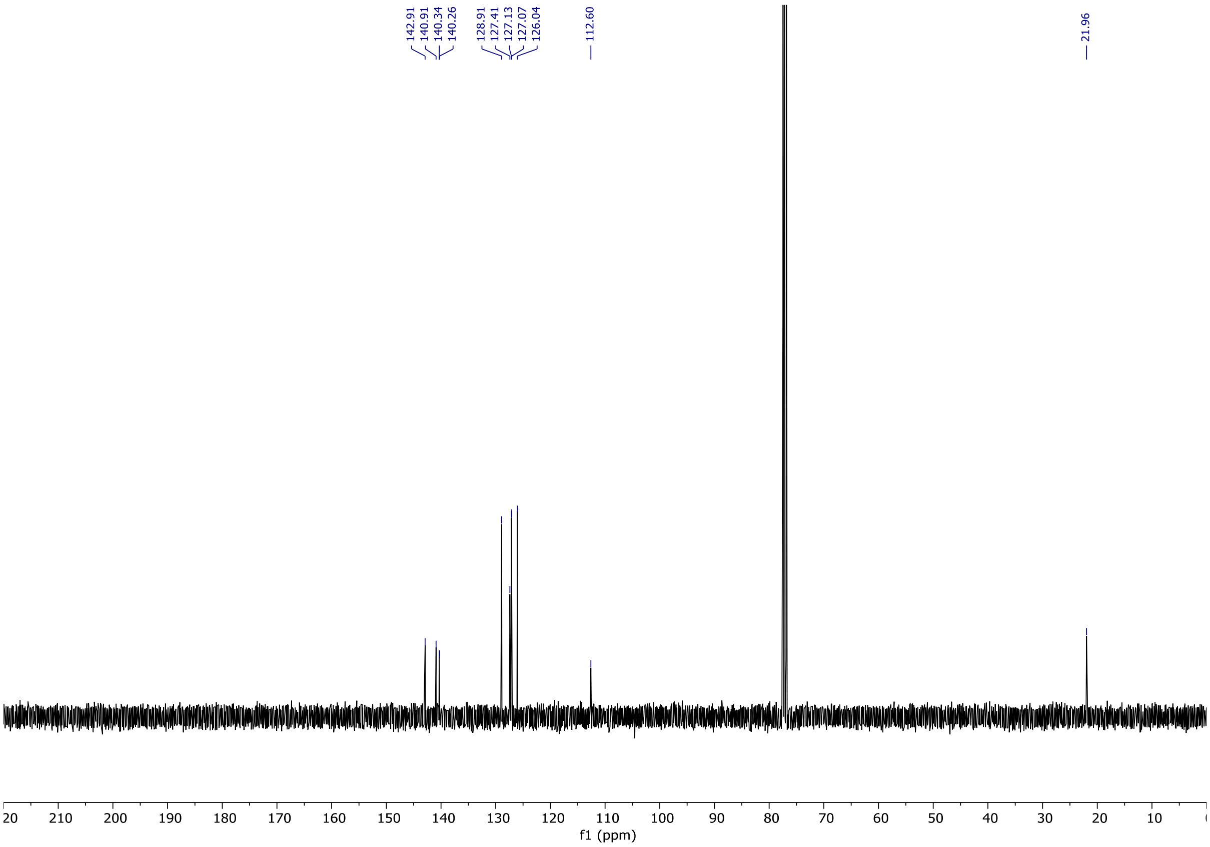
142.91
140.91
140.34
140.26
128.91
127.41
127.13
127.07
126.04
112.60
21.96
f1 (ppm)

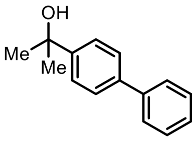
OH
Me
Me

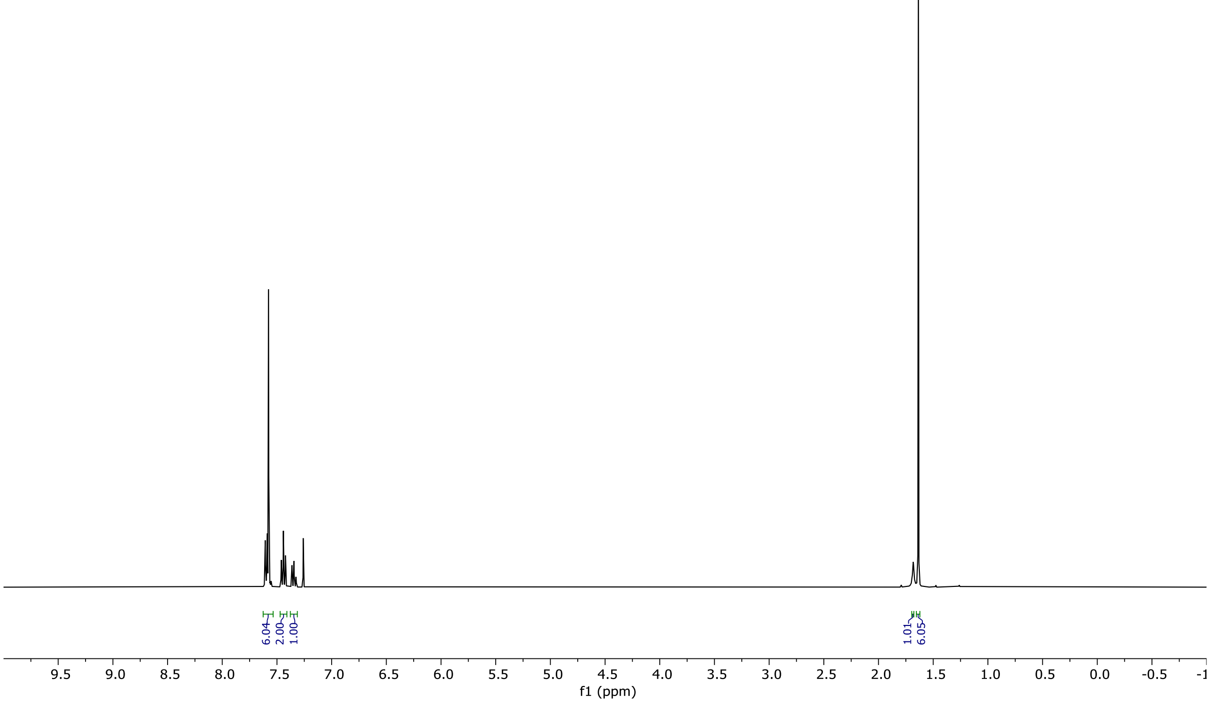
6.04
2.00
1.00
1.01
6.05
9.5 9.0 8.5 8.0 7.5 7.0 6.5 6.0 5.5 5.0 4.5 4.0 3.5 3.0 2.5 2.0 1.5 1.0 0.5 0.0 -0.5
f1 (ppm)

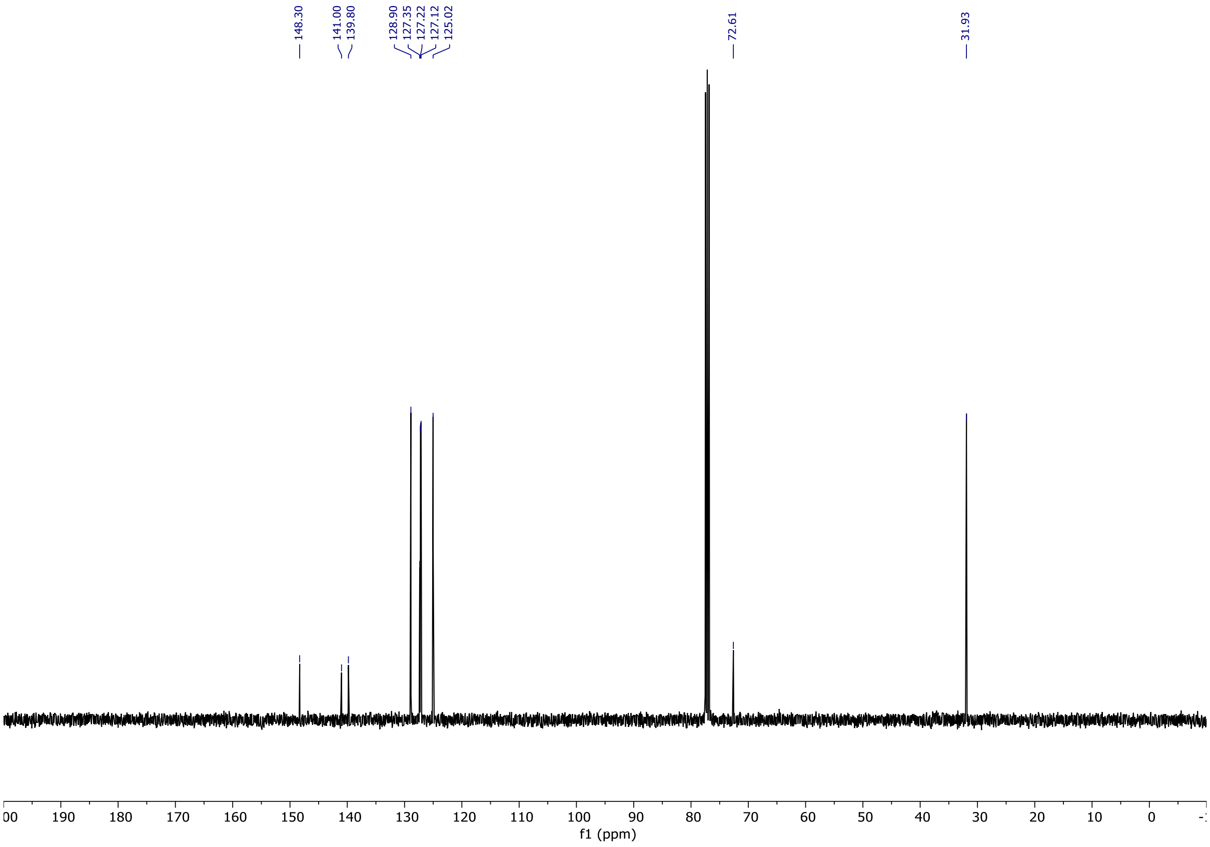
148.30
141.00
139.80
128.90
127.35
127.22
127.12
125.02
72.61
31.93
190 180 170 160 150 140 130 120 110 100 90 80 70 60 50 40 30 20 10 0
f1 (ppm)

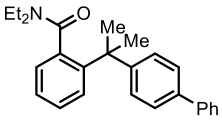

Et2N
O
Me
Me
Ph

2a

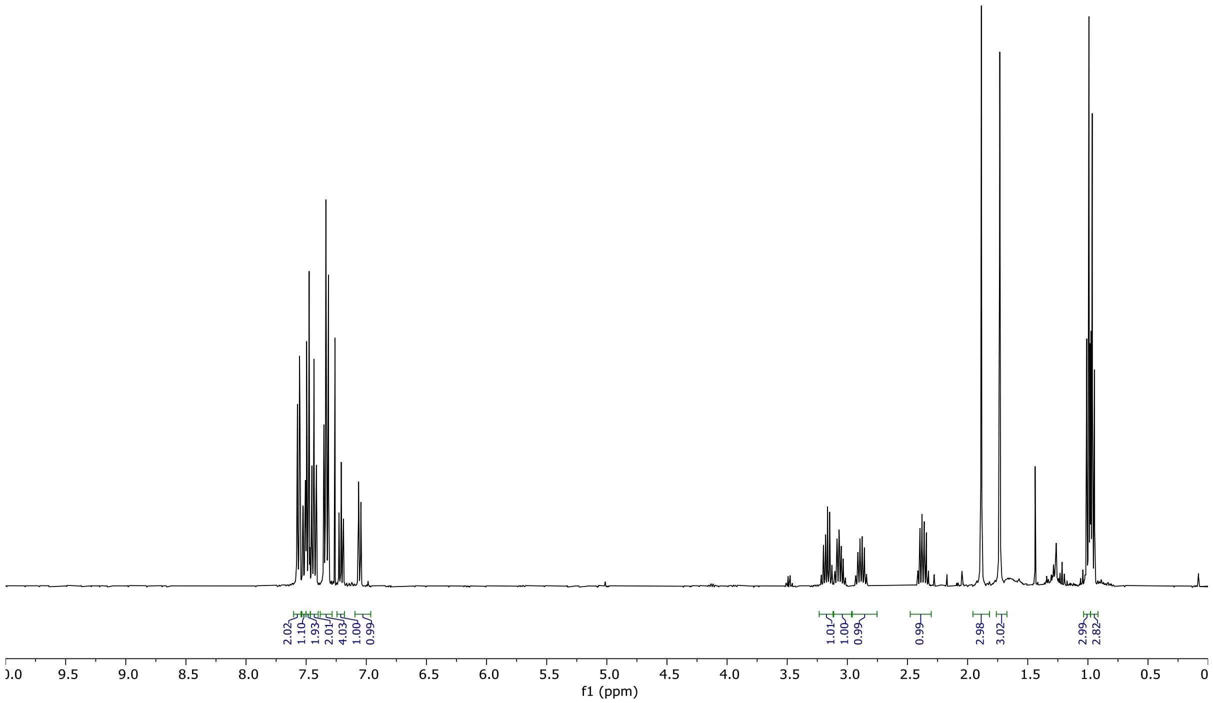

2.02
1.10
1.93
2.01
4.03
1.00
0.99
1.01
1.00
0.99
0.99
2.98
3.02
2.99
2.82
f1 (ppm)

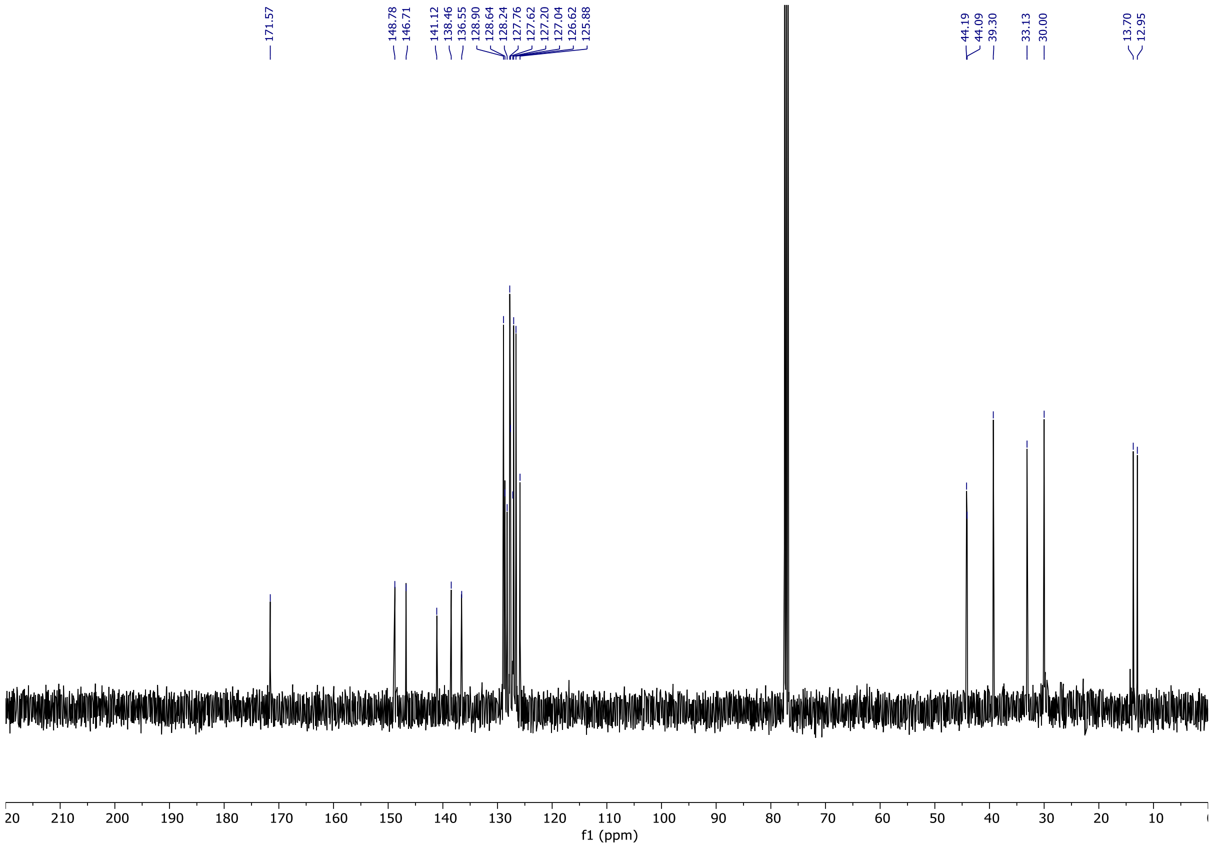

171.57
148.78
146.71
141.12
138.46
136.55
128.90
128.64
128.24
127.76
127.62
127.20
127.04
126.62
125.88
44.19
44.09
39.30
33.13
30.00
13.70
12.95
f1 (ppm)

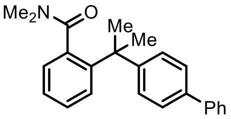
Me2N
O
Me
Me
Ph

2b

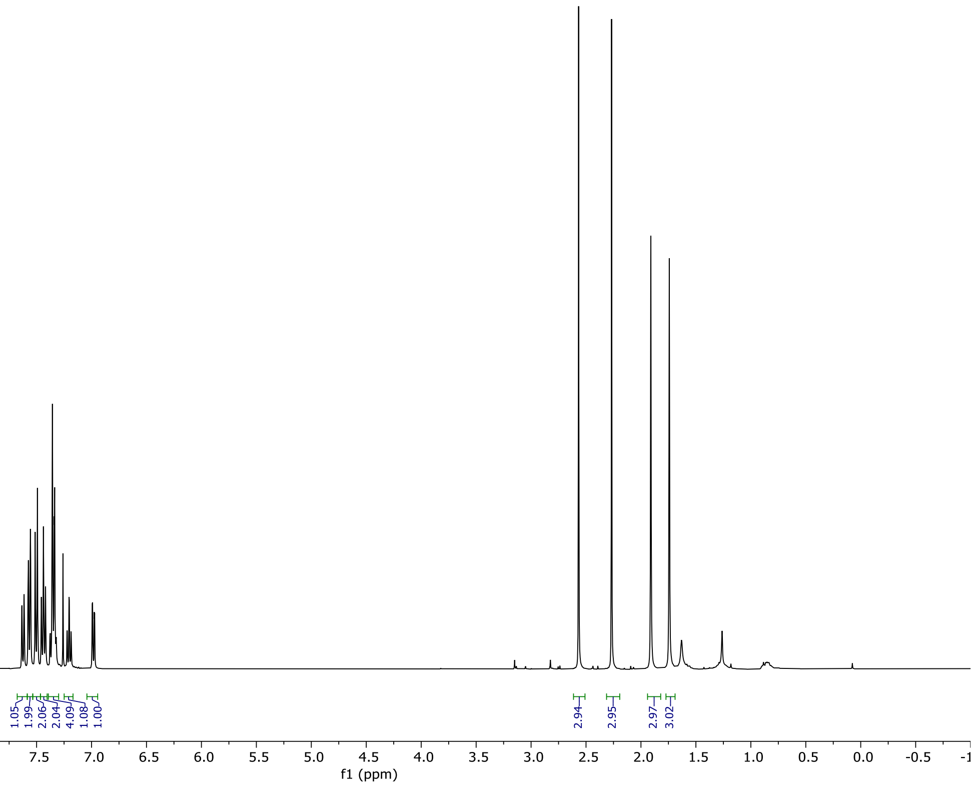
1.05
1.99
2.06
2.04
4.09
1.08
1.00
2.94
2.95
2.97
3.02
9.5 9.0 8.5 8.0 7.5 7.0 6.5 6.0 5.5 5.0 4.5 4.0 3.5 3.0 2.5 2.0 1.5 1.0 0.5 0.0 -0.5 -1
f1 (ppm)

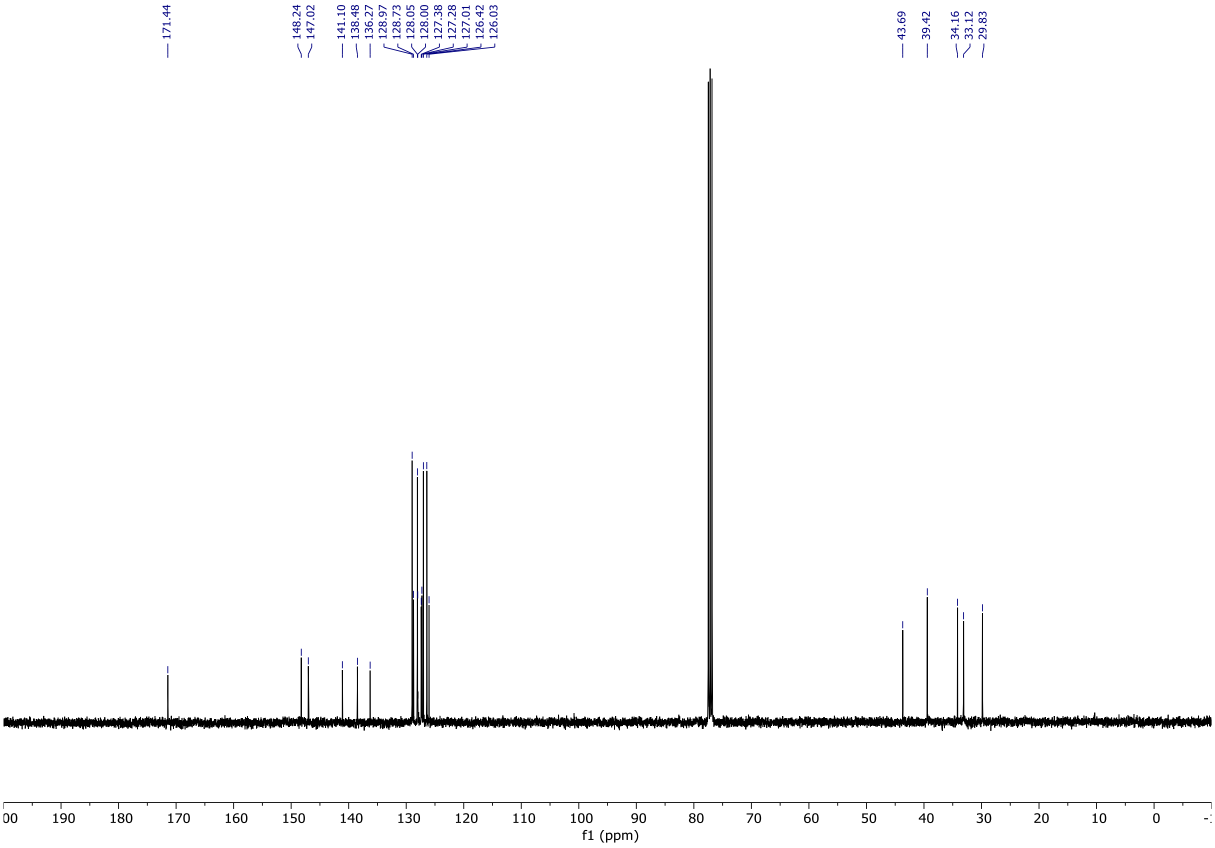
171.44
148.24
147.02
141.10
138.48
136.27
128.97
128.73
128.05
128.00
127.38
127.28
127.01
126.42
126.03
43.69
39.42
34.16
33.12
29.83
190 180 170 160 150 140 130 120 110 100 90 80 70 60 50 40 30 20 10 0
f1 (ppm)

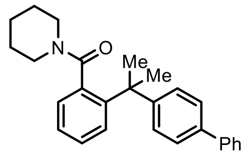

Me
Me
N
O
Ph

2d

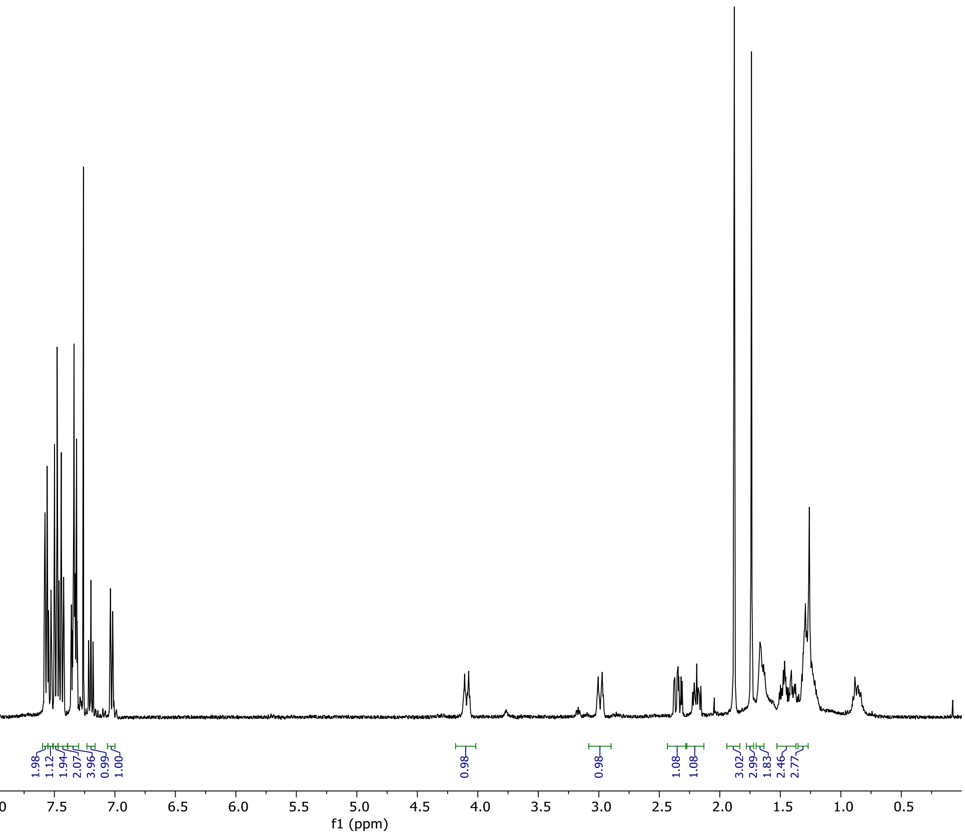

1.98
1.12
1.94
2.07
3.96
0.99
1.00
0.98
0.98
1.08
1.08
3.02
2.99
1.83
2.46
2.77
f1 (ppm)

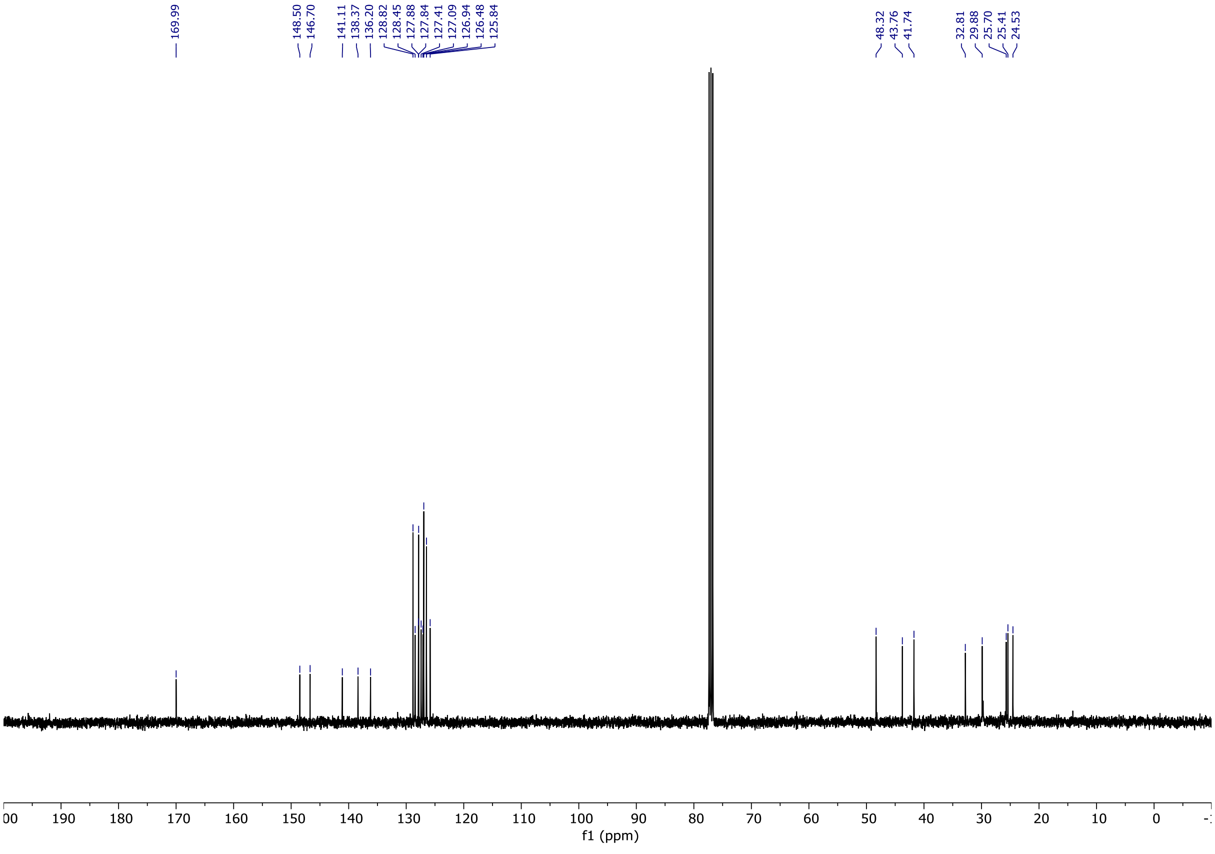

169.99
148.50
146.70
141.11
138.37
136.20
128.82
128.45
127.88
127.84
127.41
127.09
126.94
126.48
125.84
48.32
43.76
41.74
32.81
29.88
25.70
25.41
24.53
f1 (ppm)

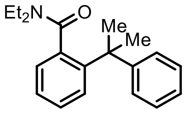
Et2N
O
Me
Me

2f

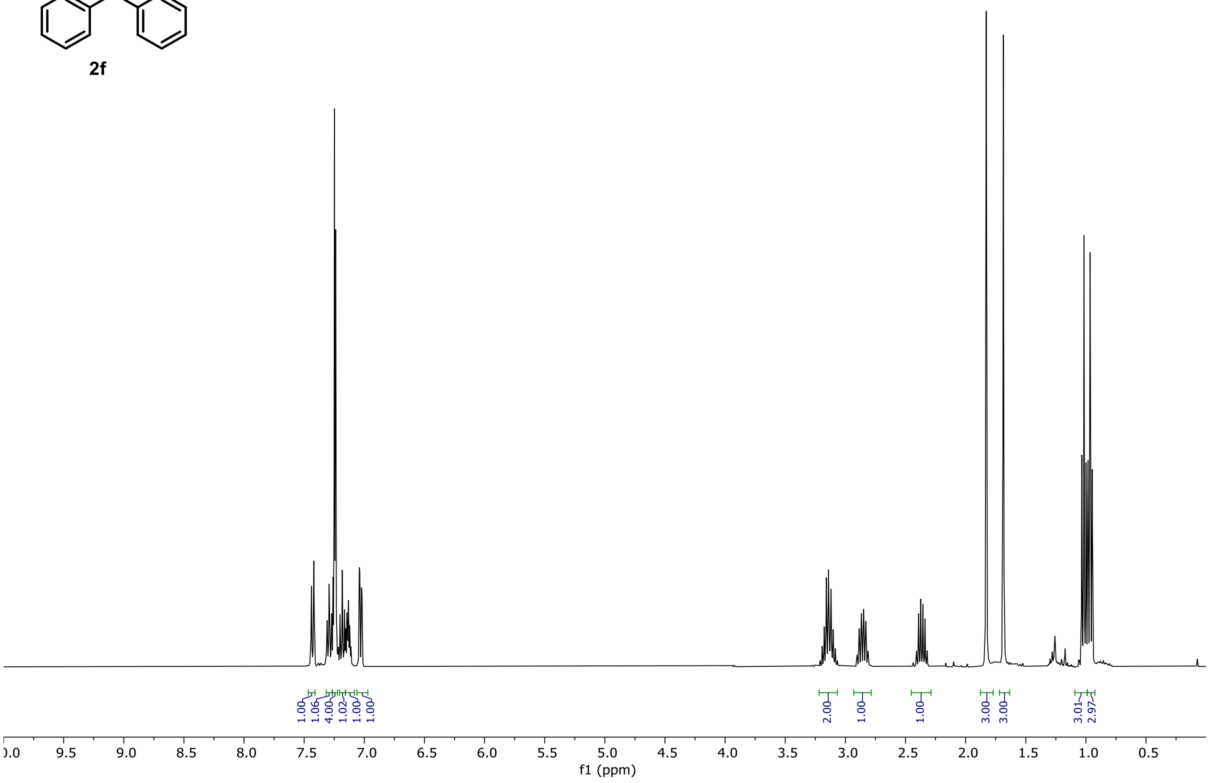
1.00
1.06
4.00
1.02
1.00
1.00
2.00
1.00
1.00
3.00
3.00
3.01
2.97
f1 (ppm)

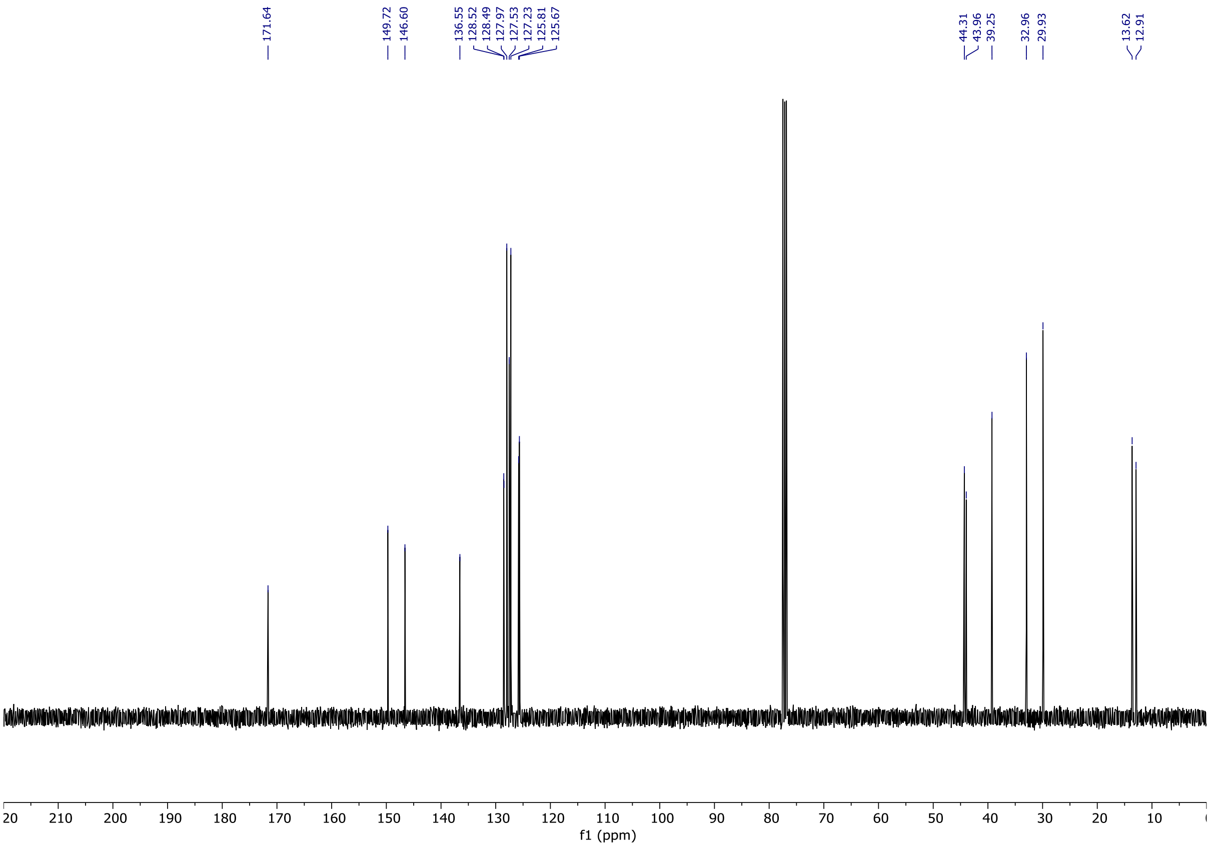
171.64
149.72
146.60
136.55
128.52
128.49
127.97
127.53
127.23
125.81
125.67
44.31
43.96
39.25
32.96
29.93
13.62
12.91
f1 (ppm)

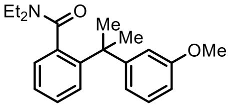
Et2N
O
Me
Me
OMe

2g

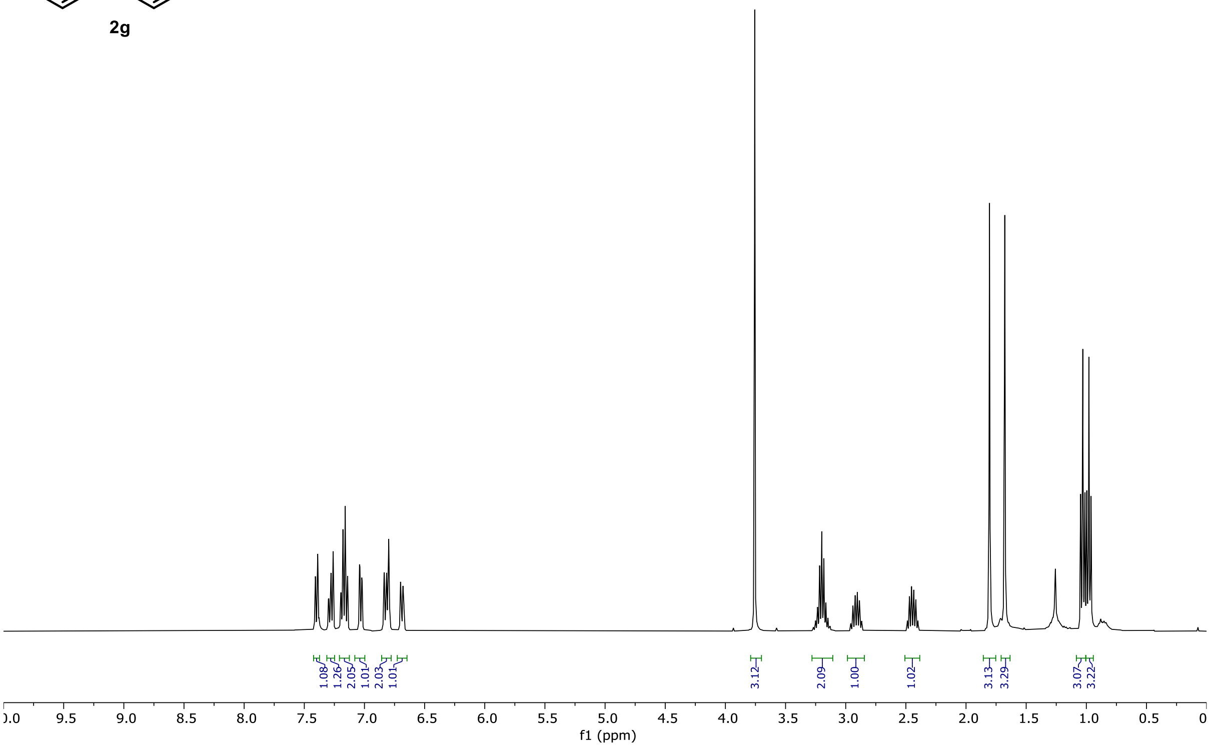
1.08
1.26
2.05
1.01
2.03
1.01
3.12
2.09
1.00
1.02
3.13
3.29
3.07
3.22
9.5 9.0 8.5 8.0 7.5 7.0 6.5 6.0 5.5 5.0 4.5 4.0 3.5 3.0 2.5 2.0 1.5 1.0 0.5 0
f1 (ppm)

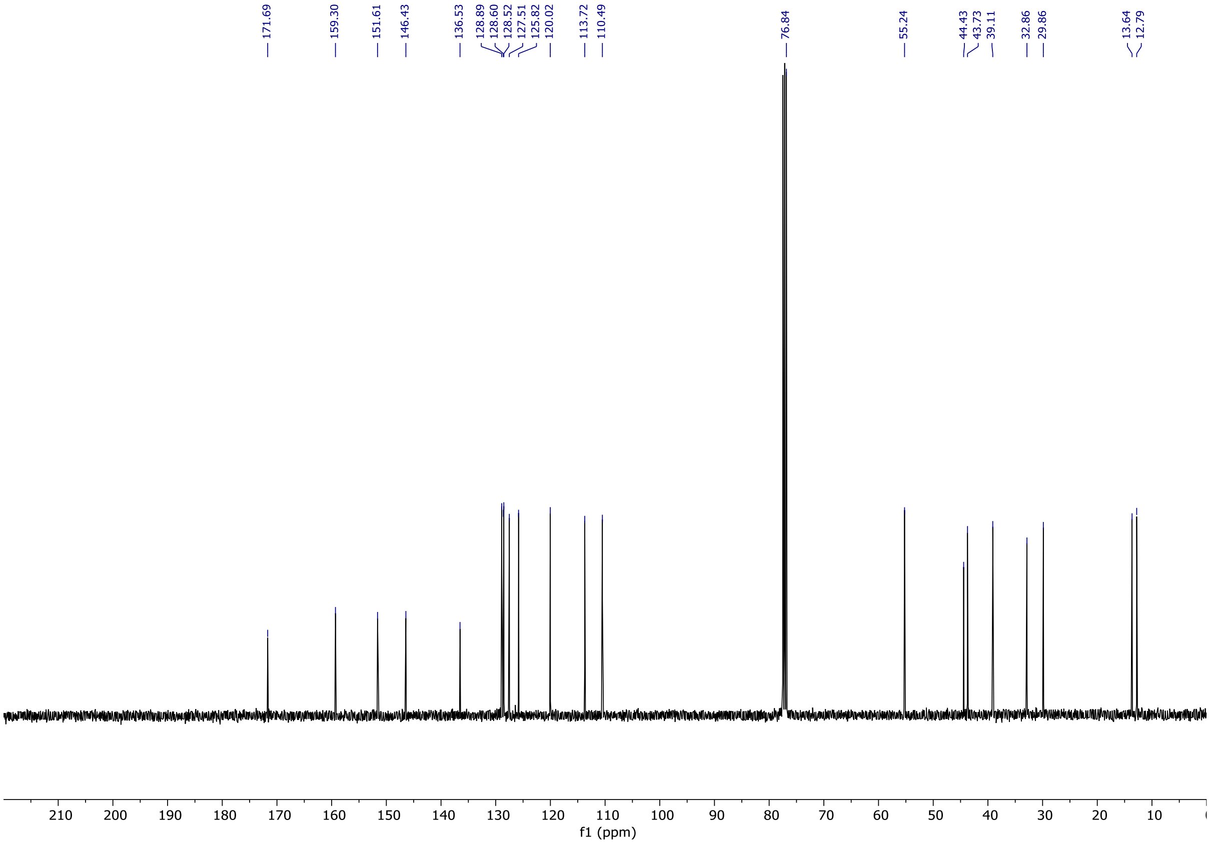
171.69
159.30
151.61
146.43
136.53
128.89
128.60
128.52
127.51
125.82
120.02
113.72
110.49
76.84
55.24
44.43
43.73
39.11
32.86
29.86
13.64
12.79
210 200 190 180 170 160 150 140 130 120 110 100 90 80 70 60 50 40 30 20 10
f1 (ppm)

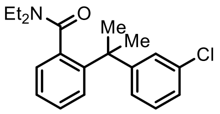
Et2N
O
Me
Me
Cl

2h

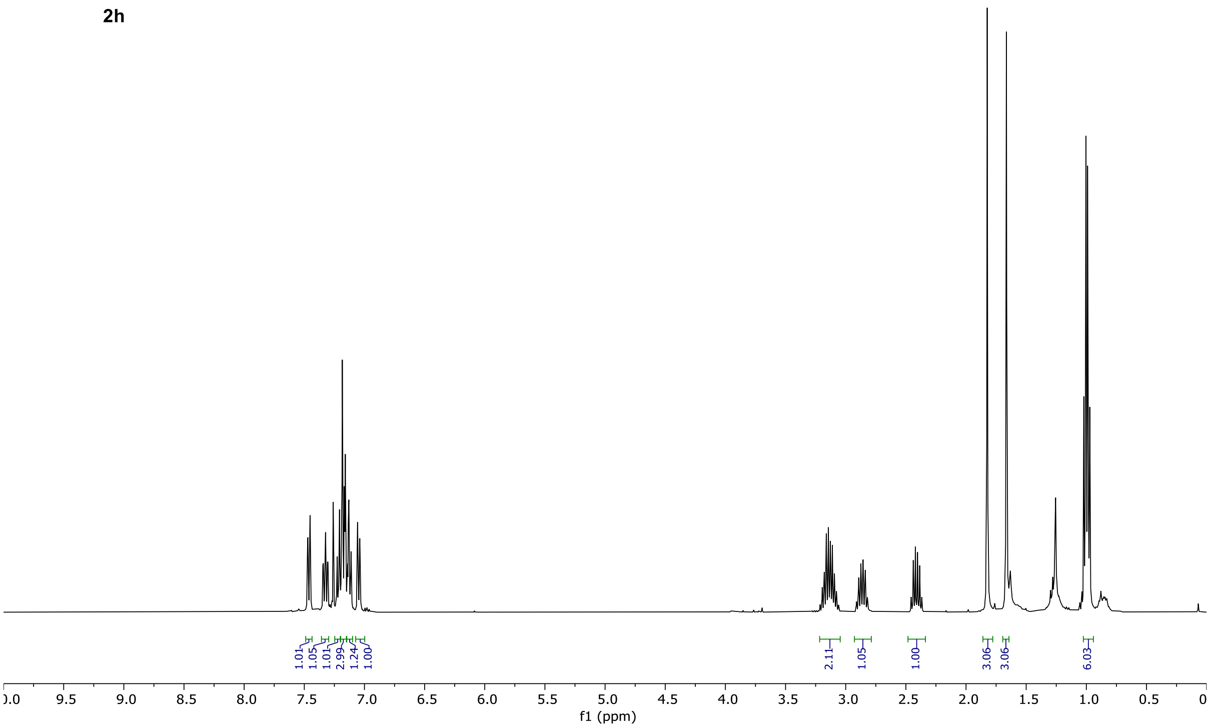
1.01
1.05
1.01
2.99
1.24
1.00
2.11
1.05
1.00
3.06
3.06
6.03
f1 (ppm)

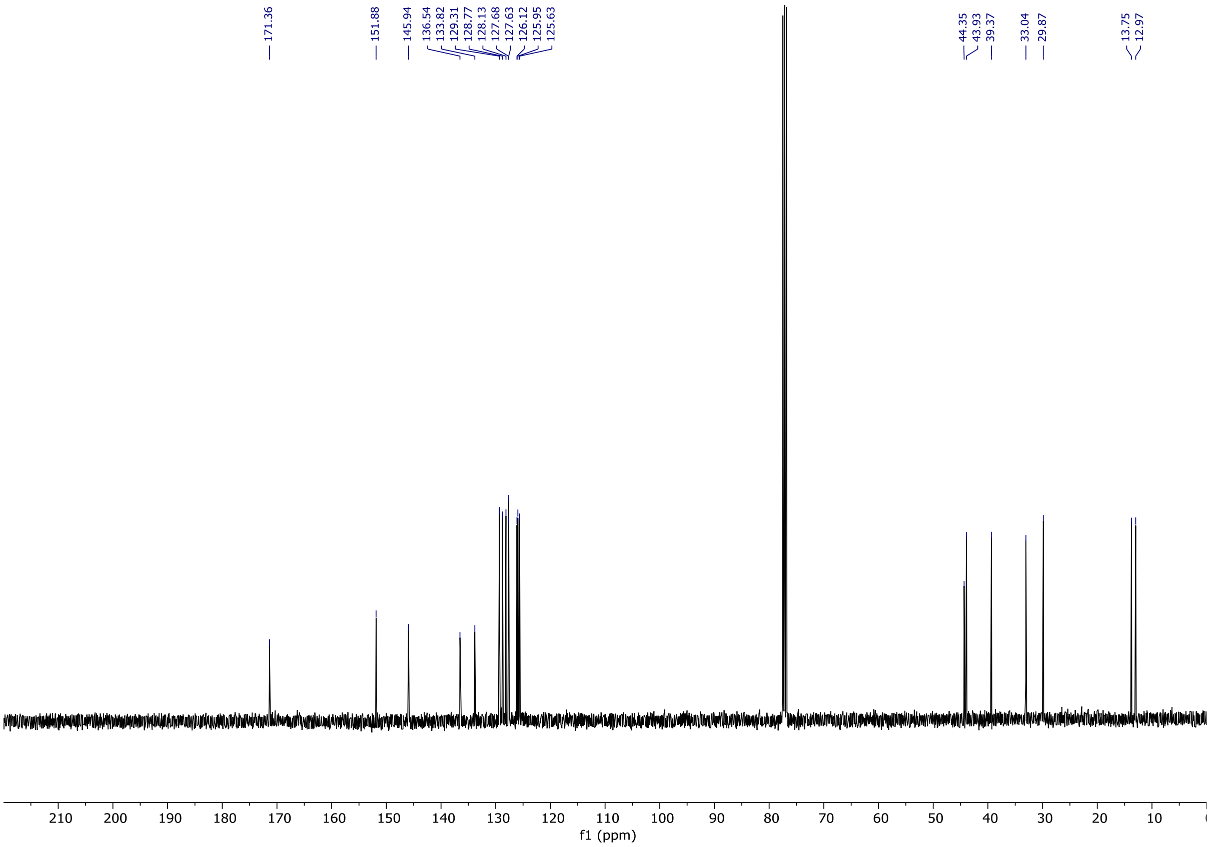
171.36
151.88
145.94
136.54
133.82
129.31
128.77
128.13
127.68
127.63
126.12
125.95
125.63
44.35
43.93
39.37
33.04
29.87
13.75
12.97
f1 (ppm)

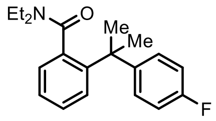
Et2N
O
Me
Me
F

2i

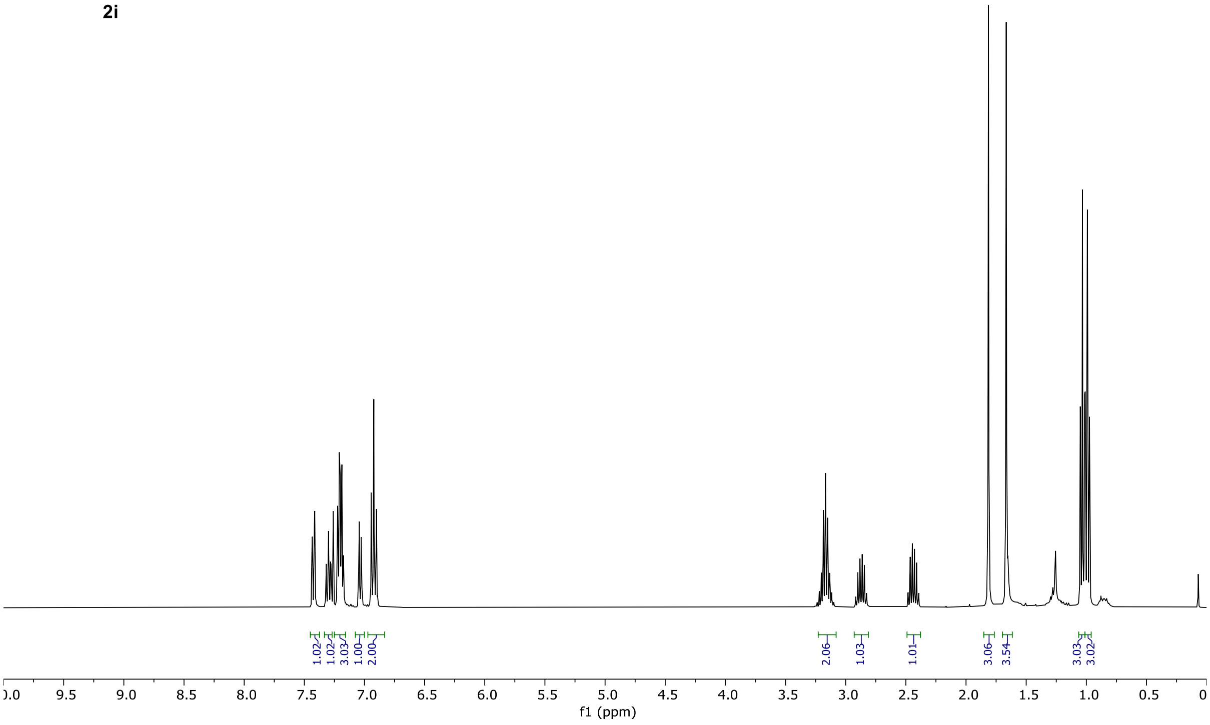
1.02
1.02
3.03
1.00
2.00
2.06
1.03
1.01
3.06
3.54
3.03
3.02
f1 (ppm)

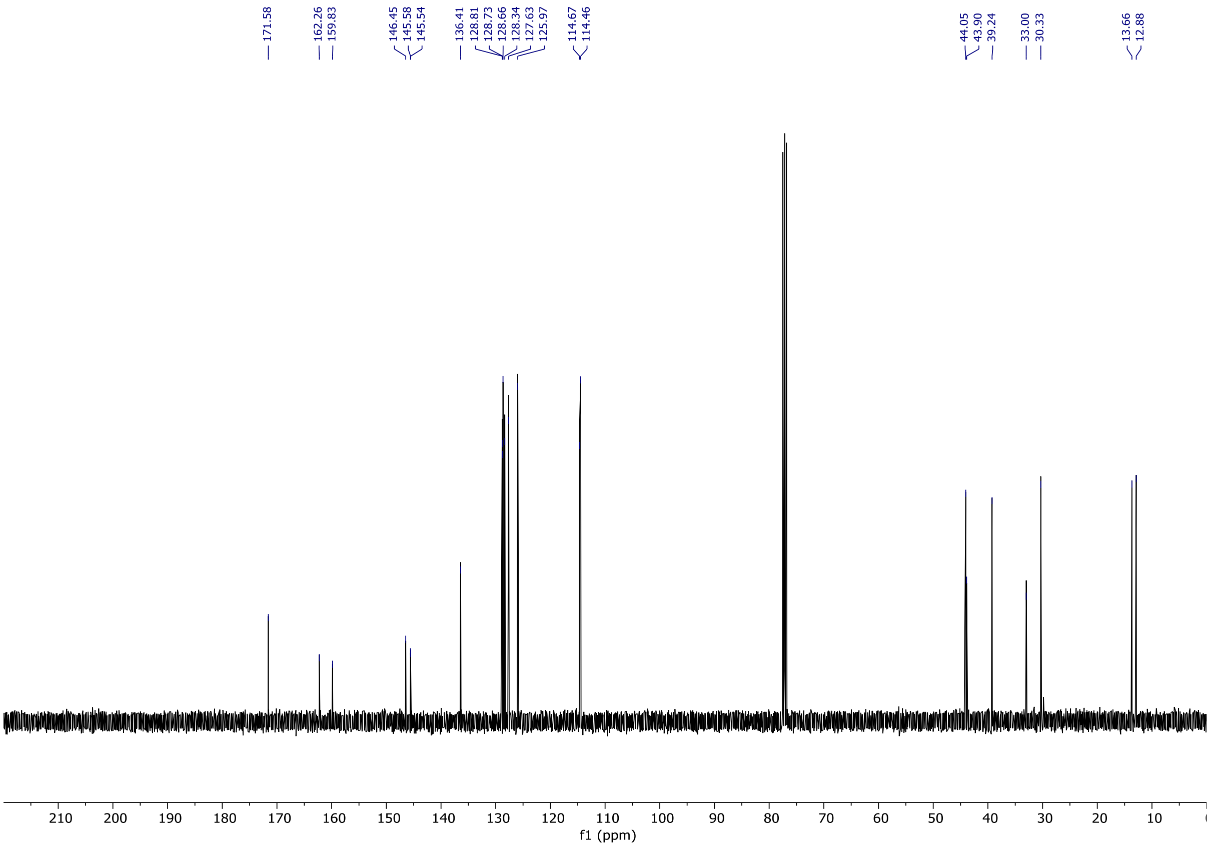
171.58
162.26
159.83
146.45
145.58
145.54
136.41
128.81
128.73
128.66
128.34
127.63
125.97
114.67
114.46
44.05
43.90
39.24
33.00
30.33
13.66
12.88
f1 (ppm)

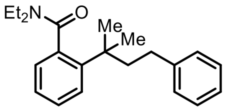
Et2N
O
Me
Me

2j

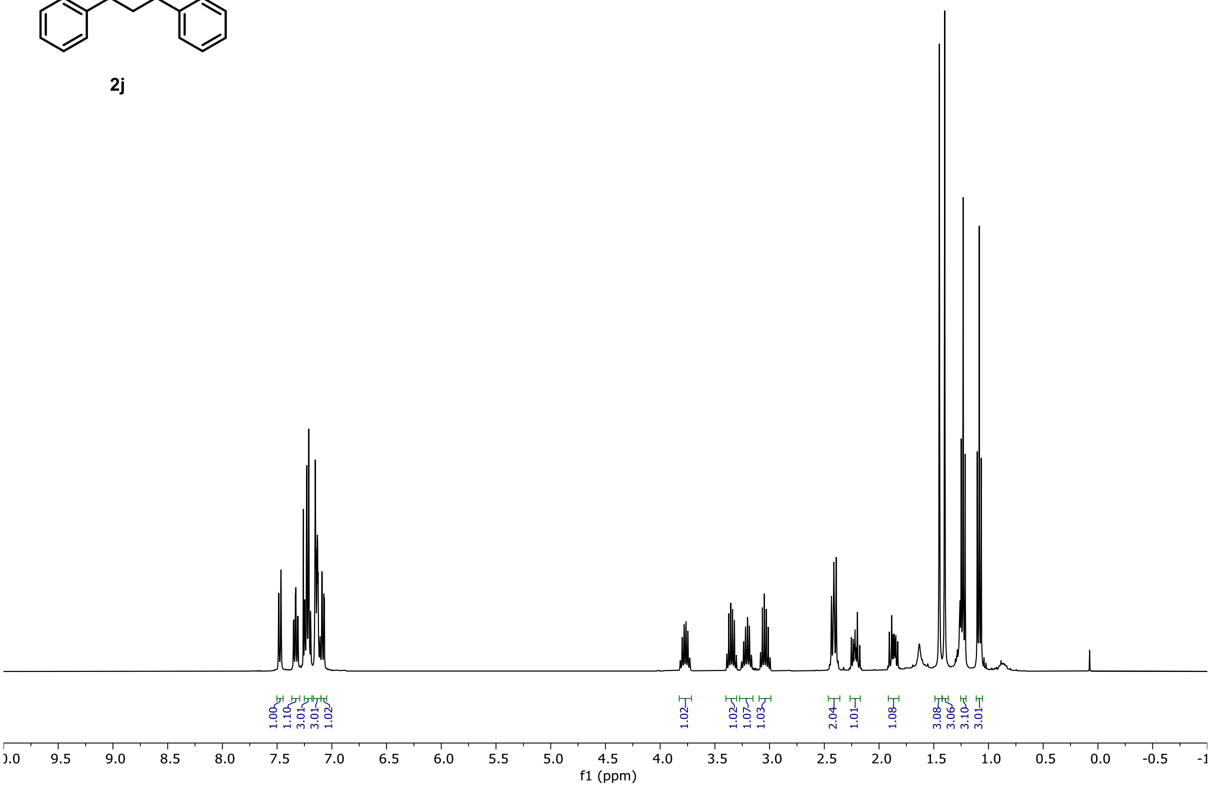
1.00
1.10
3.01
3.01
1.02
1.02
1.02
1.07
1.03
2.04
1.01
1.08
3.08
3.06
3.10
3.01
f1 (ppm)

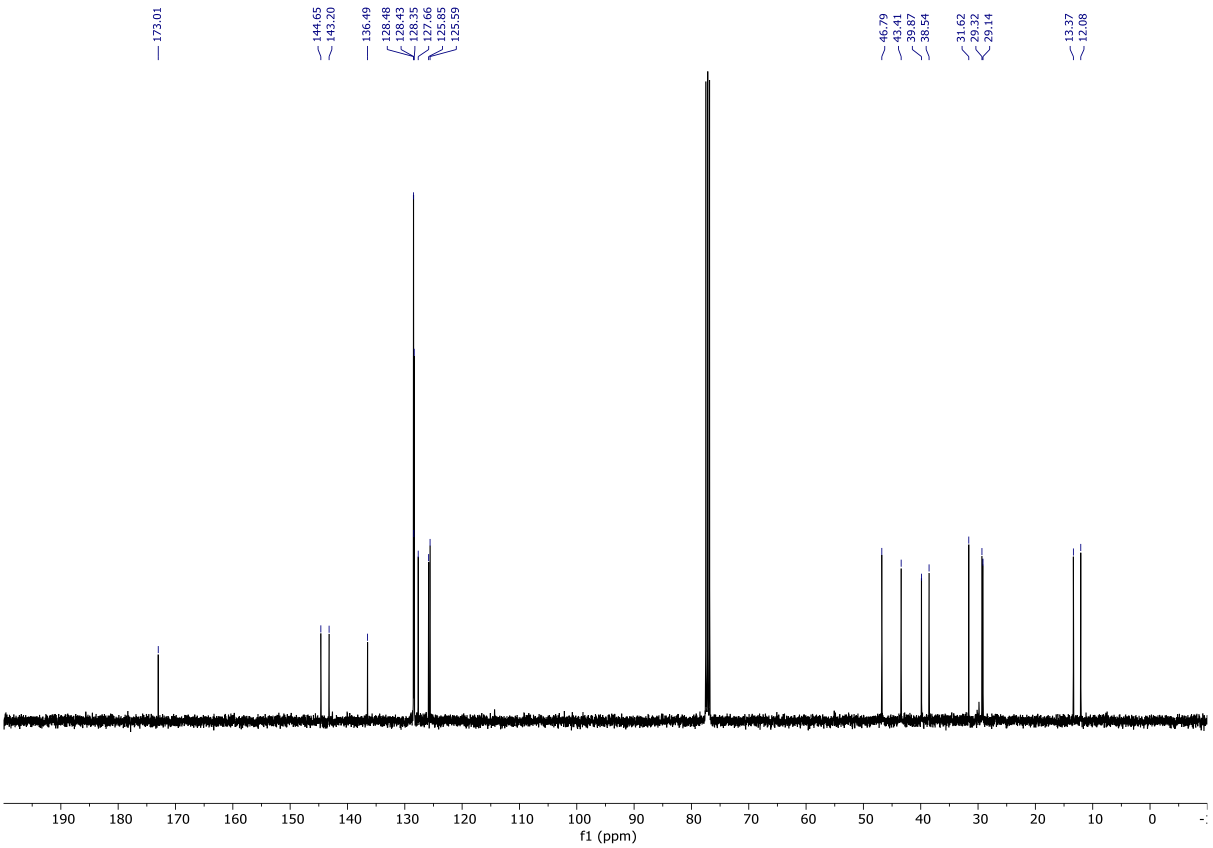
173.01
144.65
143.20
136.49
128.48
128.43
128.35
127.66
125.85
125.59
46.79
43.41
39.87
38.54
31.62
29.32
29.14
13.37
12.08
f1 (ppm)

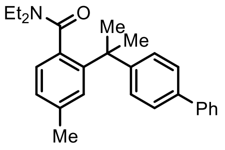
Et2N
O
Me
Me
Me
Ph

2k

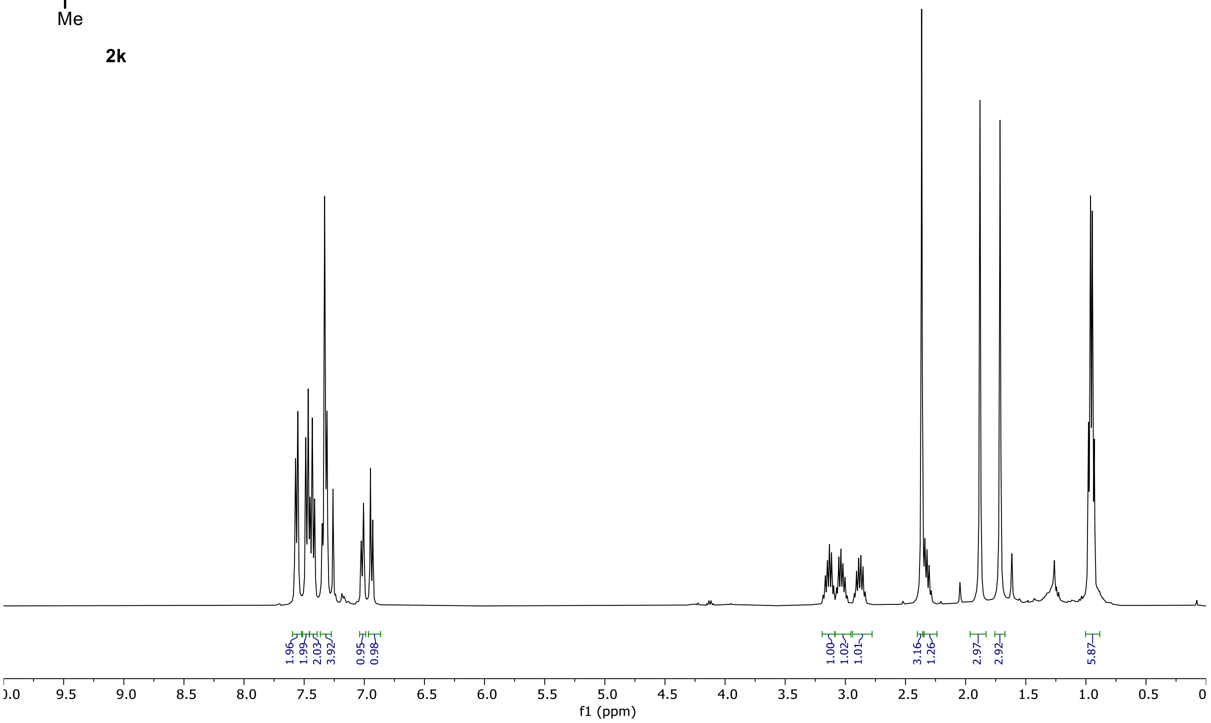
1.96
1.99
2.03
3.92
0.95
0.98
1.00
1.02
1.01
3.16
1.26
2.97
2.92
5.87
f1 (ppm)

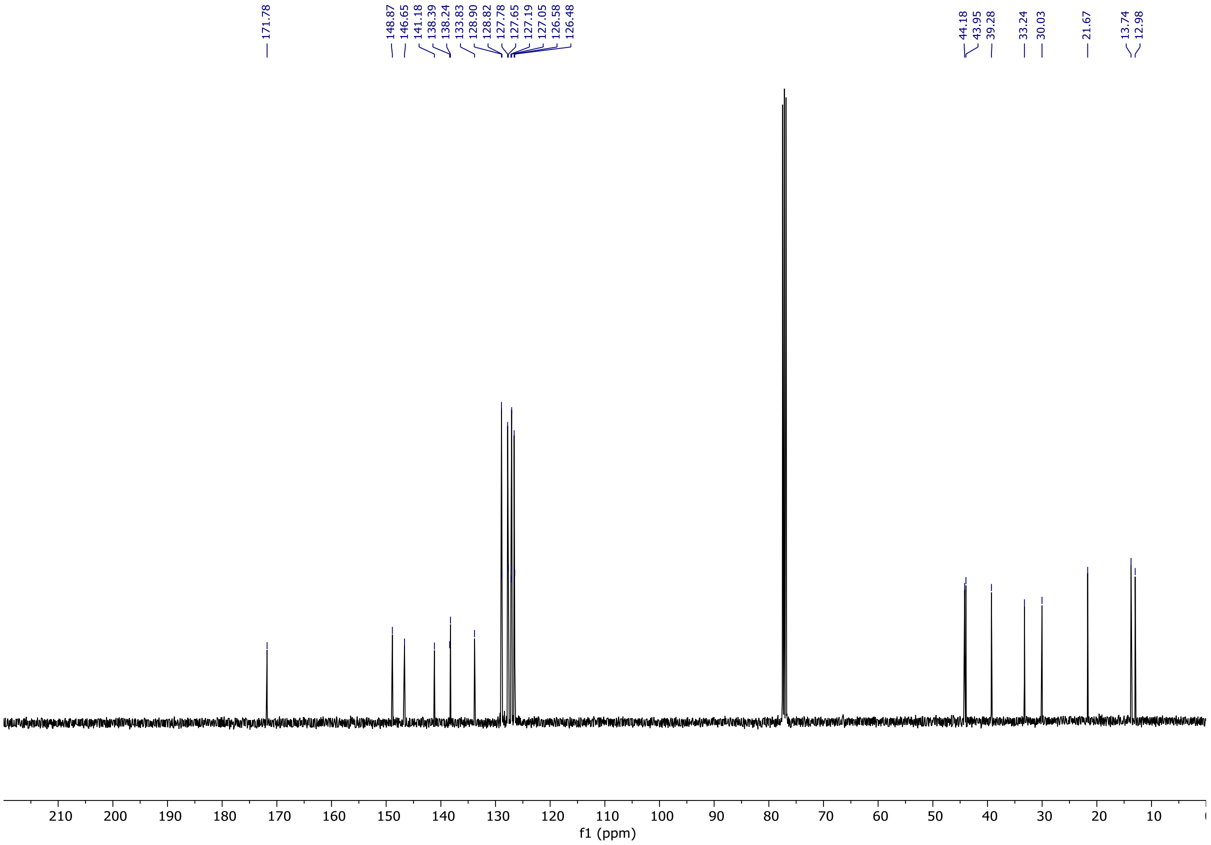
171.78
148.87
146.65
141.18
138.39
138.24
133.83
128.90
128.82
127.78
127.65
127.19
127.05
126.58
126.48
44.18
43.95
39.28
33.24
30.03
21.67
13.74
12.98
f1 (ppm)

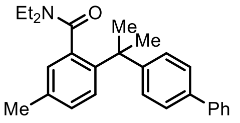
Et2N
O
Me
Me
Me
Ph

2l

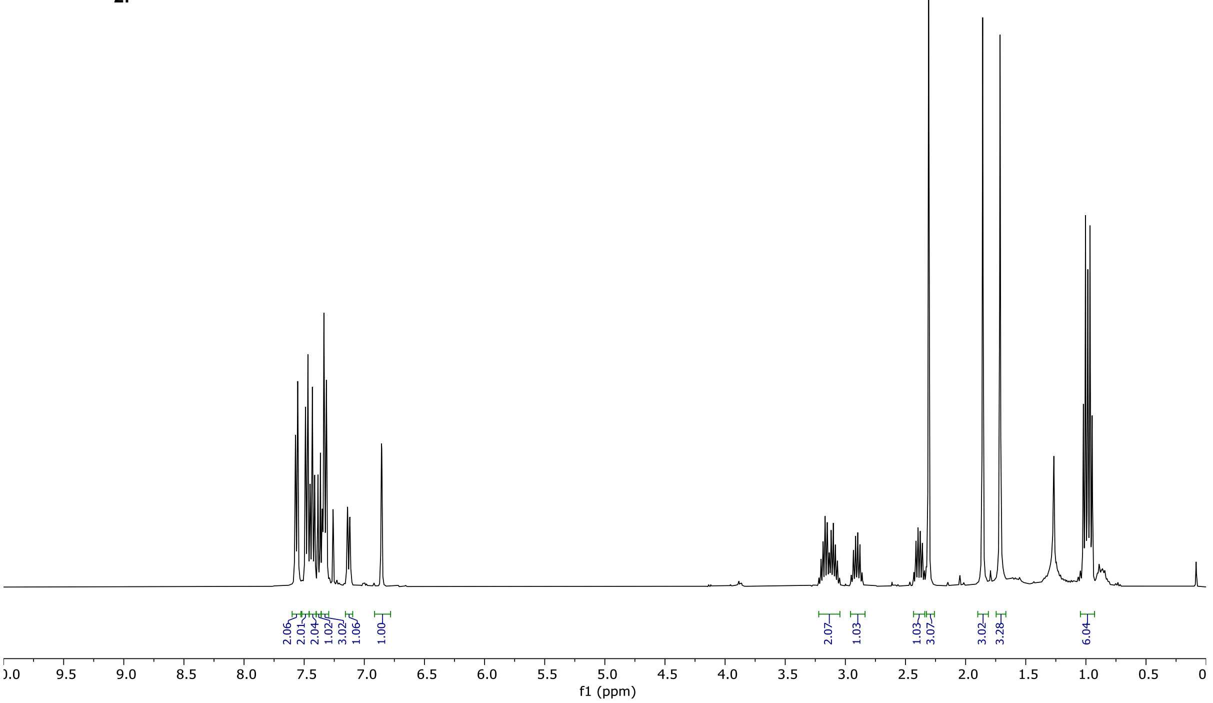
2.06
2.01
2.04
1.02
3.02
1.06
1.00
2.07
1.03
1.03
3.07
3.02
3.28
6.04
f1 (ppm)

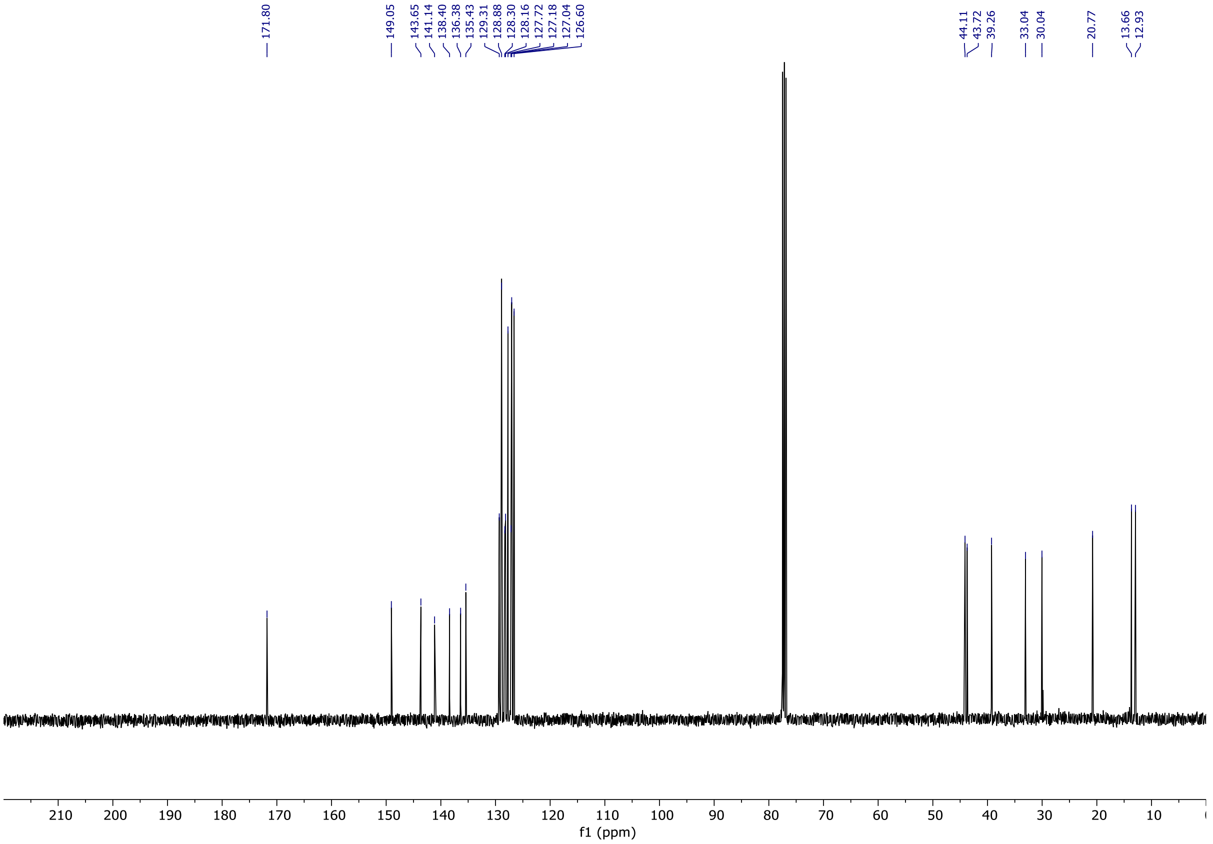
171.80
149.05
143.65
141.14
138.40
136.38
135.43
129.31
128.88
128.30
128.16
127.72
127.18
127.04
126.60
44.11
43.72
39.26
33.04
30.04
20.77
13.66
12.93
f1 (ppm)

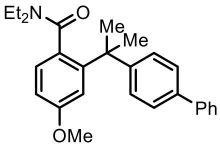
Et2N
O
Me
Me
Ph
OMe

2m

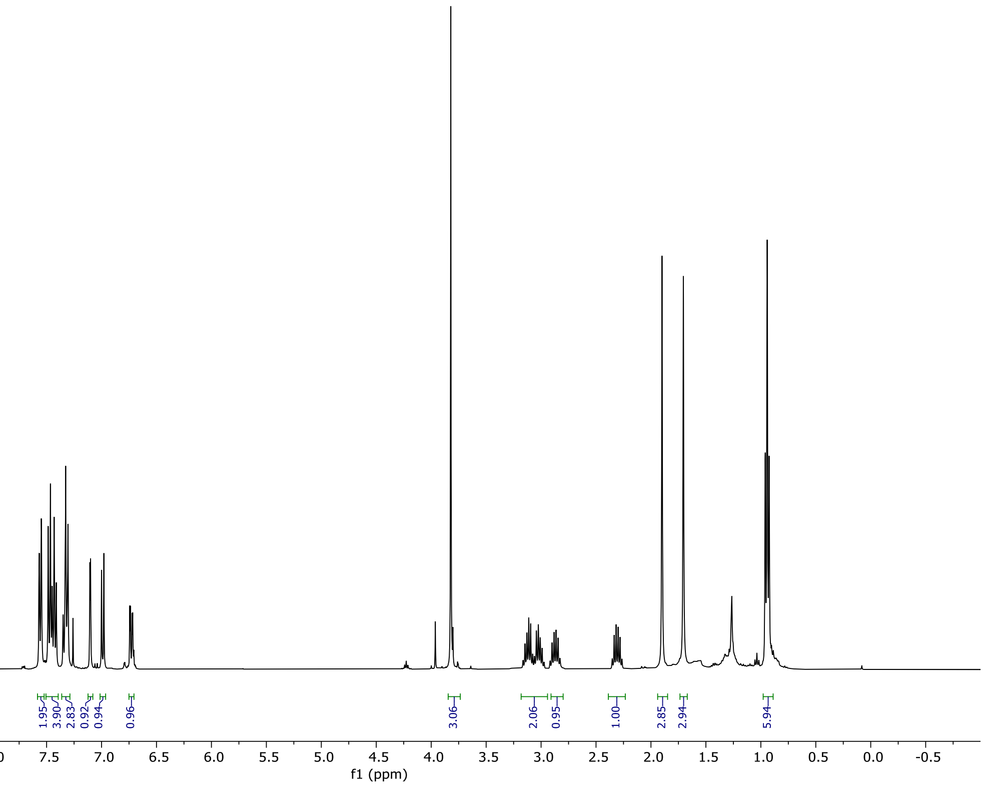
1.95
3.90
2.83
0.92
0.94
0.96
3.06
2.06
0.95
1.00
2.85
2.94
5.94
f1 (ppm)

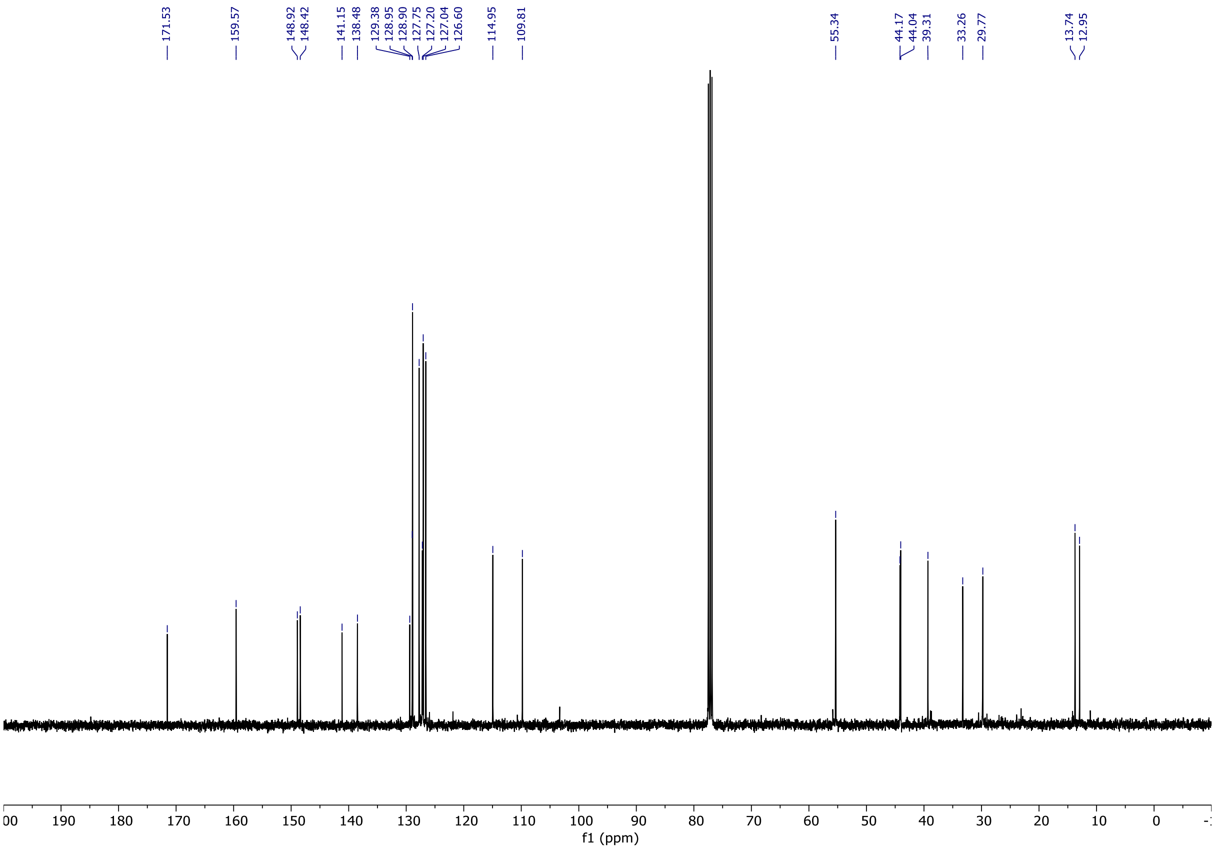
171.53
159.57
148.92
148.42
141.15
138.48
129.38
128.95
128.90
127.75
127.20
127.04
126.60
114.95
109.81
55.34
44.17
44.04
39.31
33.26
29.77
13.74
12.95
f1 (ppm)

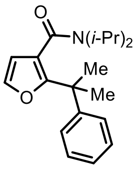
O
N(i-Pr)2
Me
Me
O

4a

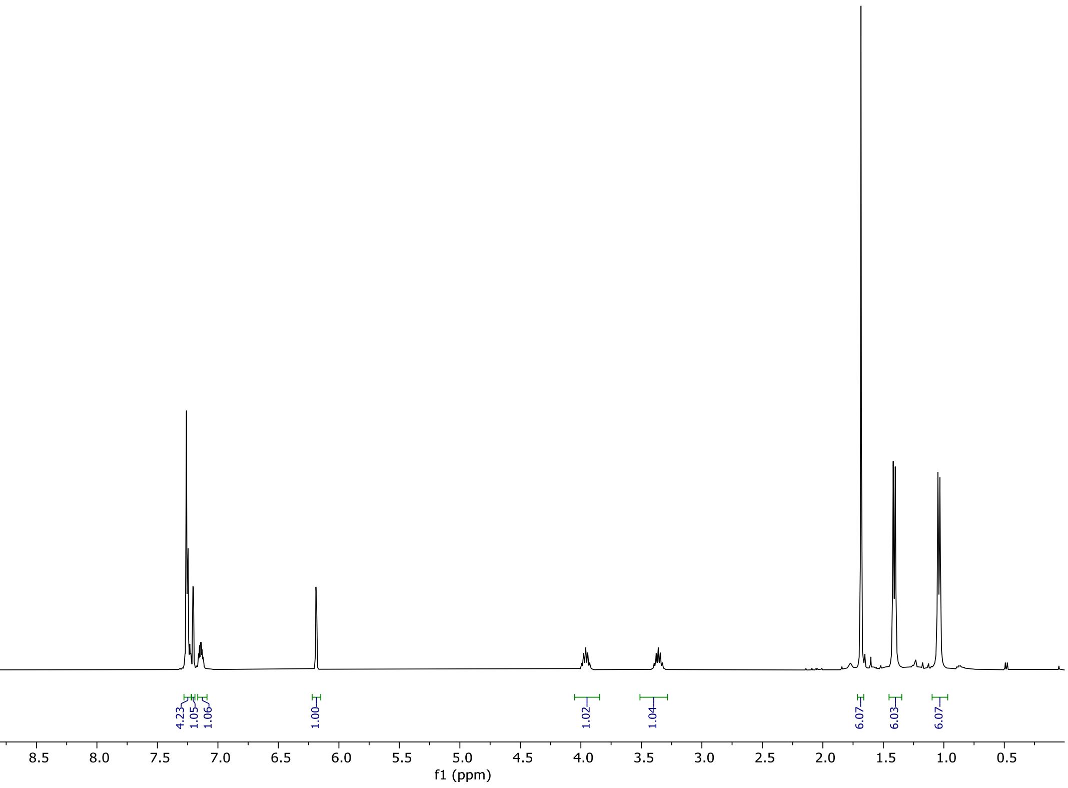
4.23
1.05
1.06
1.00
1.02
1.04
6.07
6.03
6.07
9.5 9.0 8.5 8.0 7.5 7.0 6.5 6.0 5.5 5.0 4.5 4.0 3.5 3.0 2.5 2.0 1.5 1.0 0.5
f1 (ppm)

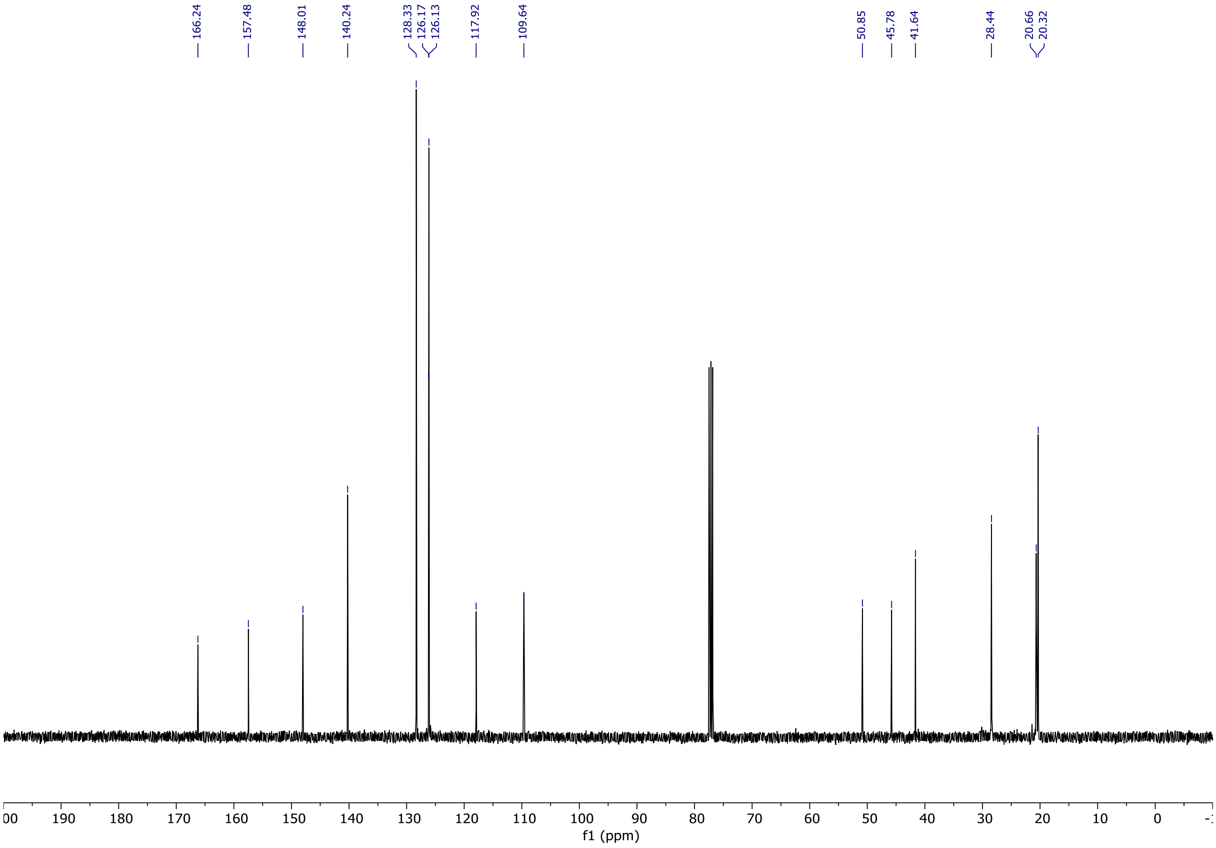
166.24
157.48
148.01
140.24
128.33
126.17
126.13
117.92
109.64
50.85
45.78
41.64
28.44
20.66
20.32
190 180 170 160 150 140 130 120 110 100 90 80 70 60 50 40 30 20 10 0
f1 (ppm)

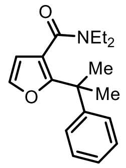
O
NEt2
Me
Me
O

4b

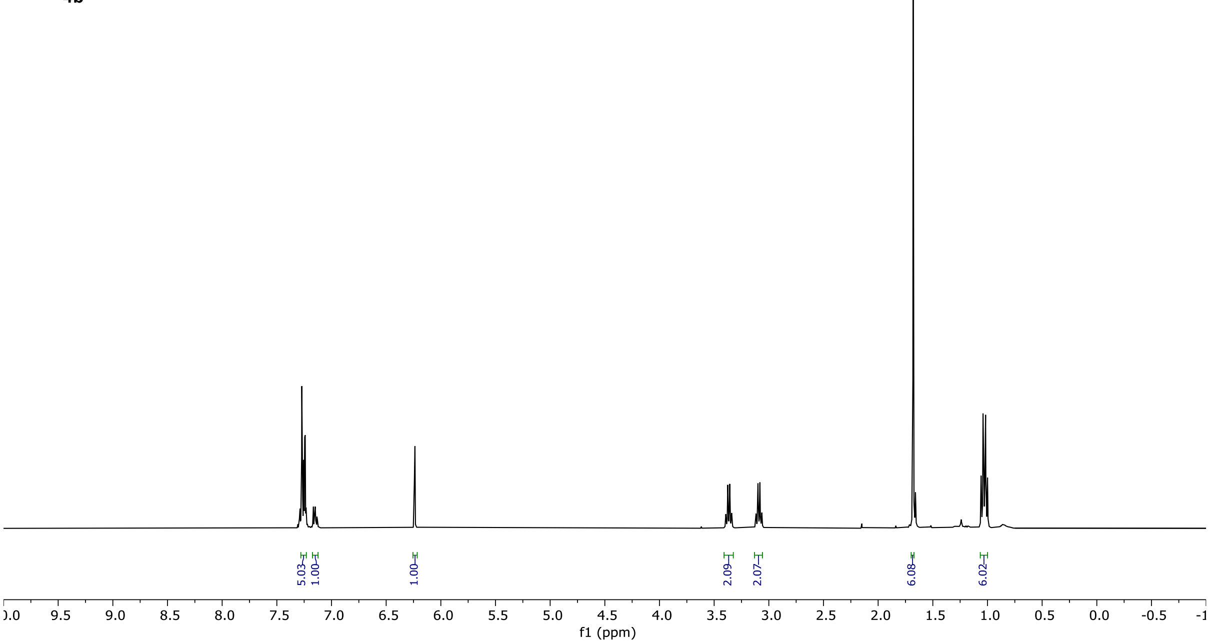
5.03
1.00
1.00
2.09
2.07
6.08
6.02
9.5 9.0 8.5 8.0 7.5 7.0 6.5 6.0 5.5 5.0 4.5 4.0 3.5 3.0 2.5 2.0 1.5 1.0 0.5 0.0 -0.5
f1 (ppm)

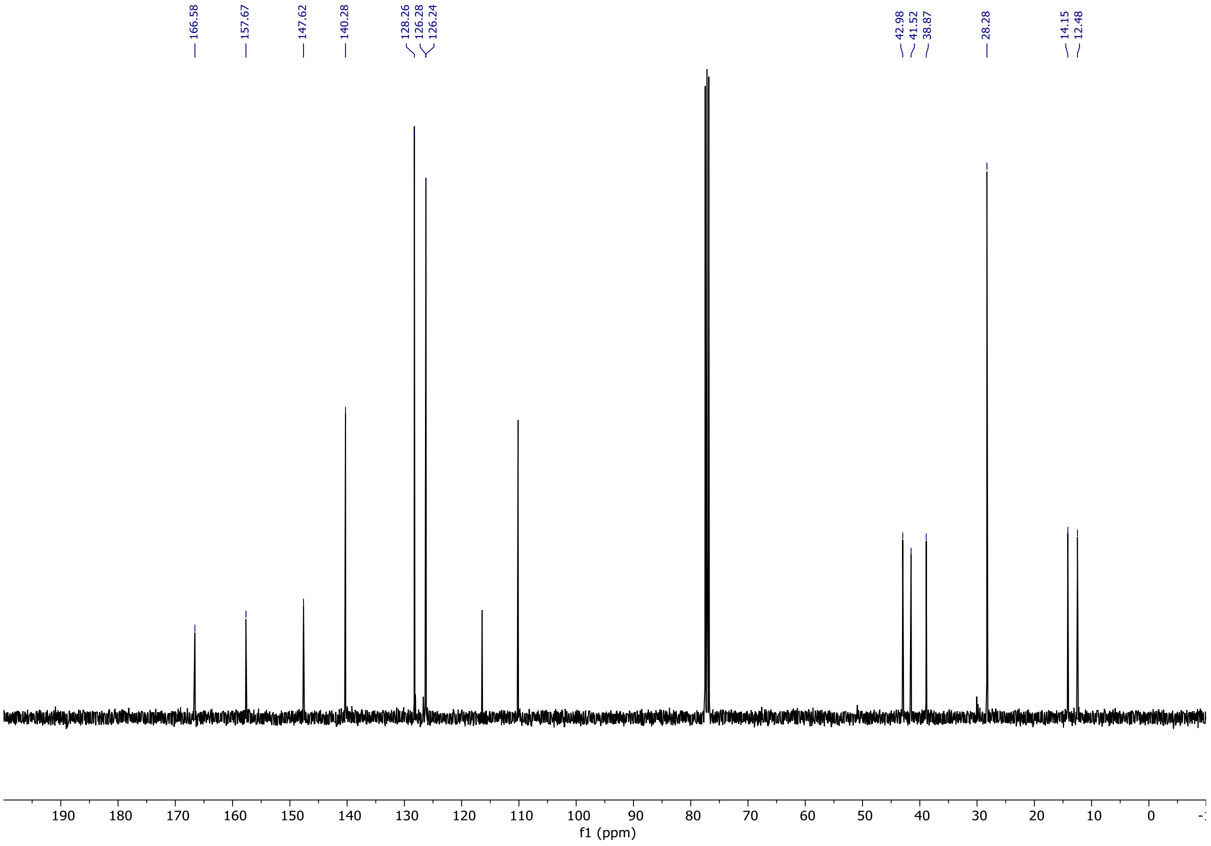
166.58
157.67
147.62
140.28
128.26
126.28
126.24
42.98
41.52
38.87
28.28
14.15
12.48
190 180 170 160 150 140 130 120 110 100 90 80 70 60 50 40 30 20 10 0
f1 (ppm)

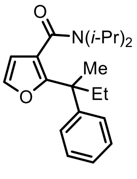
O
N(i-Pr)2
Me
Et
O

4c

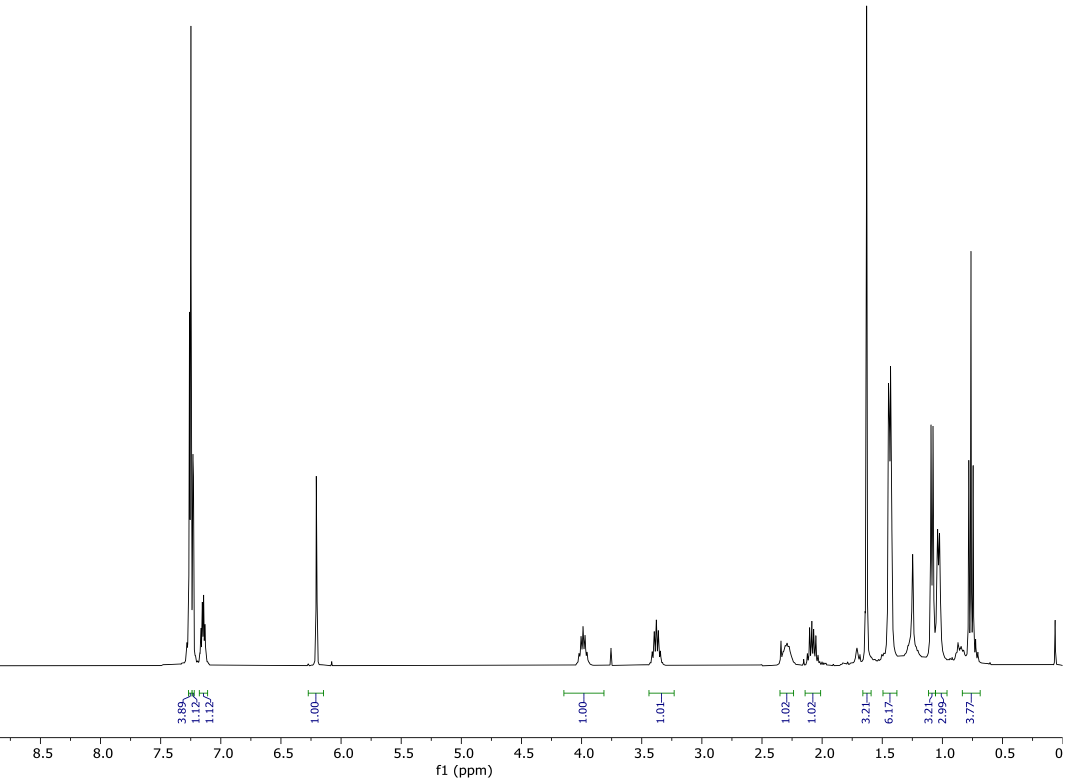
3.89
1.12
1.12
1.00
1.00
1.01
1.02
1.02
3.21
6.17
3.21
2.99
3.77
9.5 9.0 8.5 8.0 7.5 7.0 6.5 6.0 5.5 5.0 4.5 4.0 3.5 3.0 2.5 2.0 1.5 1.0 0.5 0
f1 (ppm)

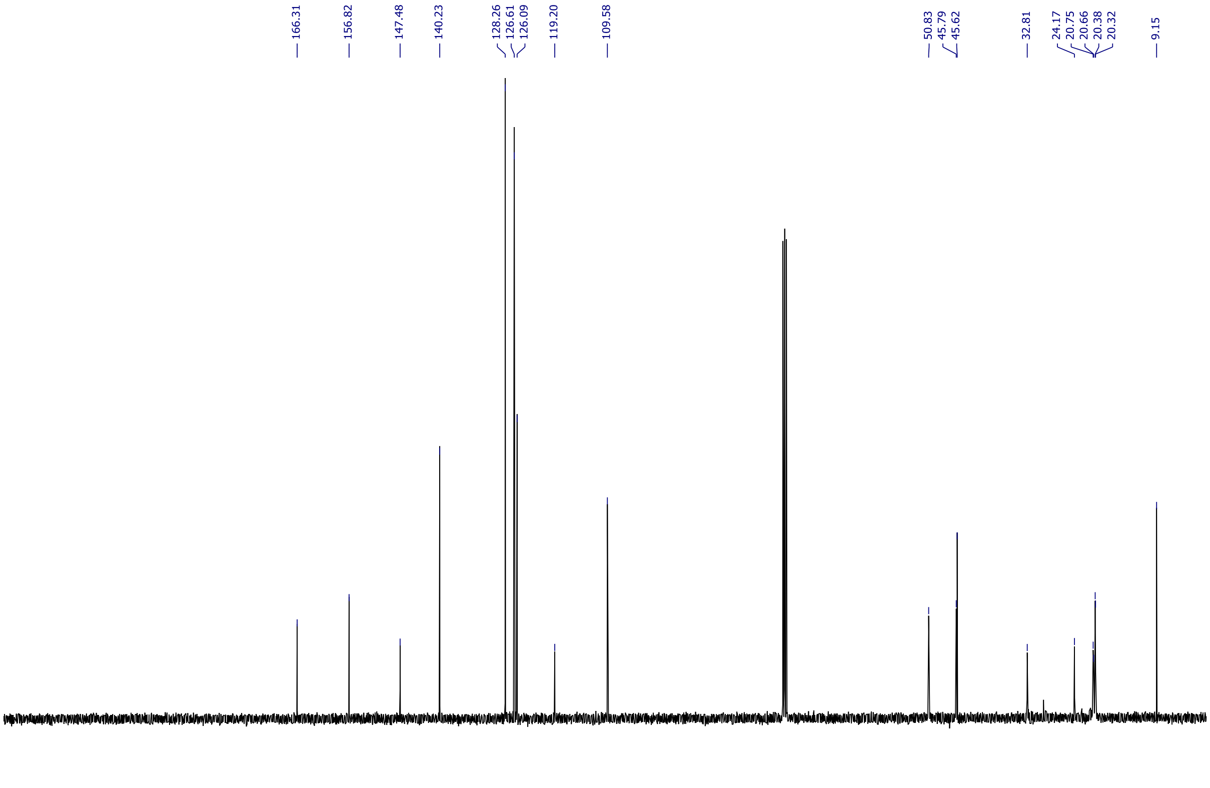
166.31
156.82
147.48
140.23
128.26
126.61
126.09
119.20
109.58
50.83
45.79
45.62
32.81
24.17
20.75
20.66
20.38
20.32
9.15
210 200 190 180 170 160 150 140 130 120 110 100 90 80 70 60 50 40 30 20 10
f1 (ppm)

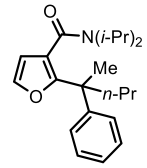
O
N(i-Pr)2
Me
n-Pr
O

4d

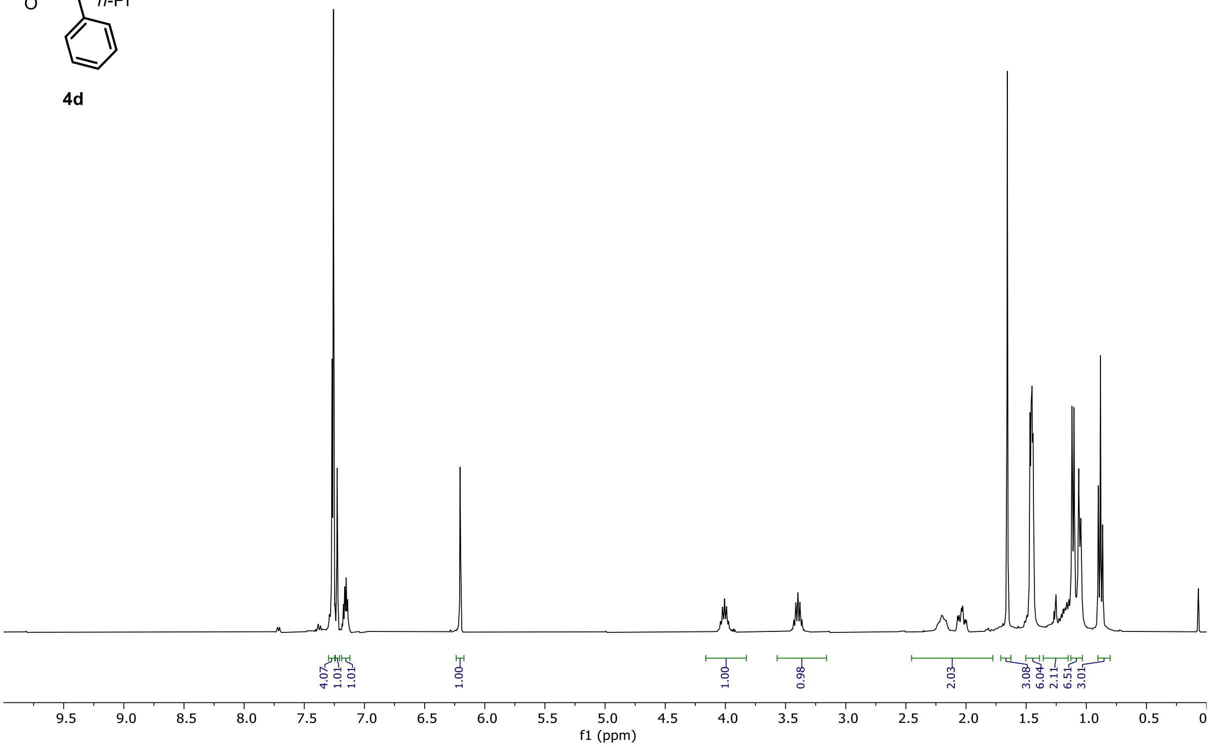
4.07
1.01
1.01
1.00
1.00
0.98
2.03
3.08
6.04
2.11
6.51
3.01
9.5 9.0 8.5 8.0 7.5 7.0 6.5 6.0 5.5 5.0 4.5 4.0 3.5 3.0 2.5 2.0 1.5 1.0 0.5 0
f1 (ppm)

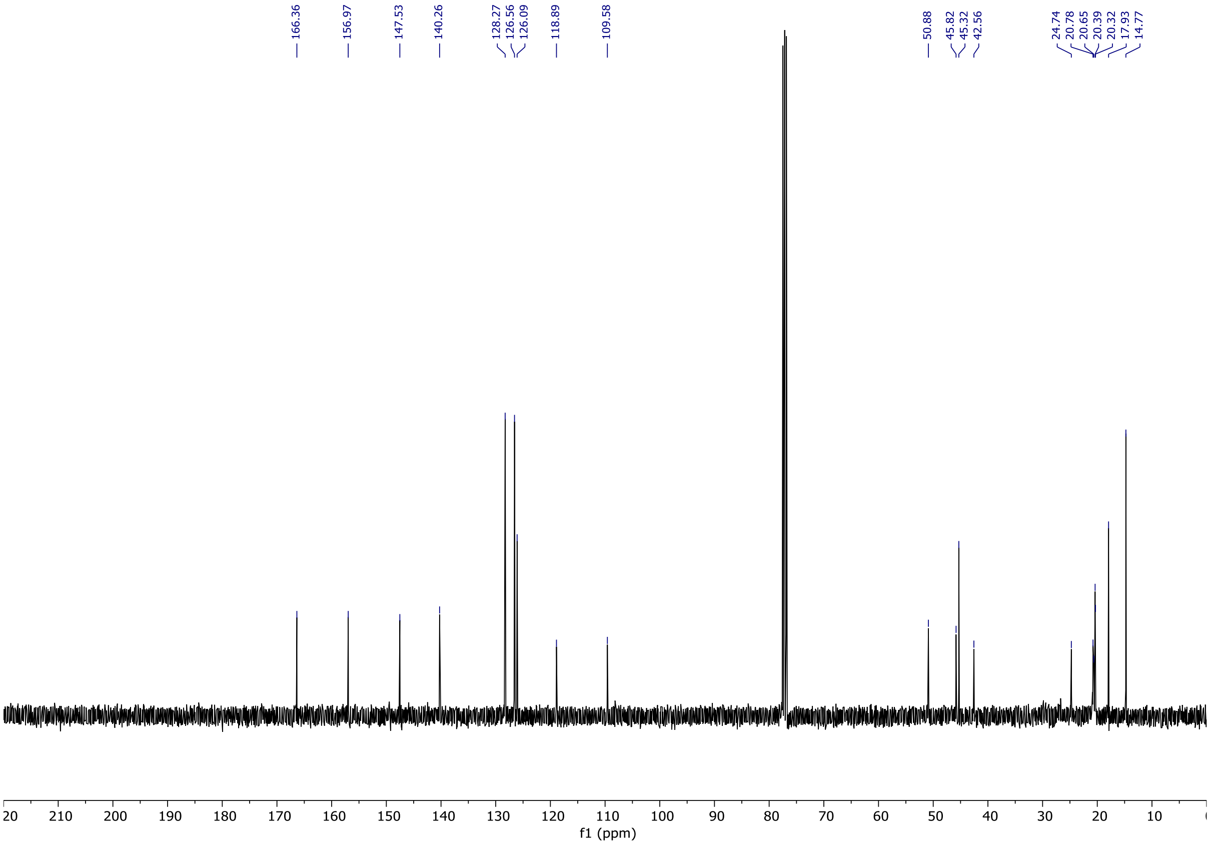
166.36
156.97
147.53
140.26
128.27
126.56
126.09
118.89
109.58
50.88
45.82
45.32
42.56
24.74
20.78
20.65
20.39
20.32
17.93
14.77
20 210 200 190 180 170 160 150 140 130 120 110 100 90 80 70 60 50 40 30 20 10
f1 (ppm)

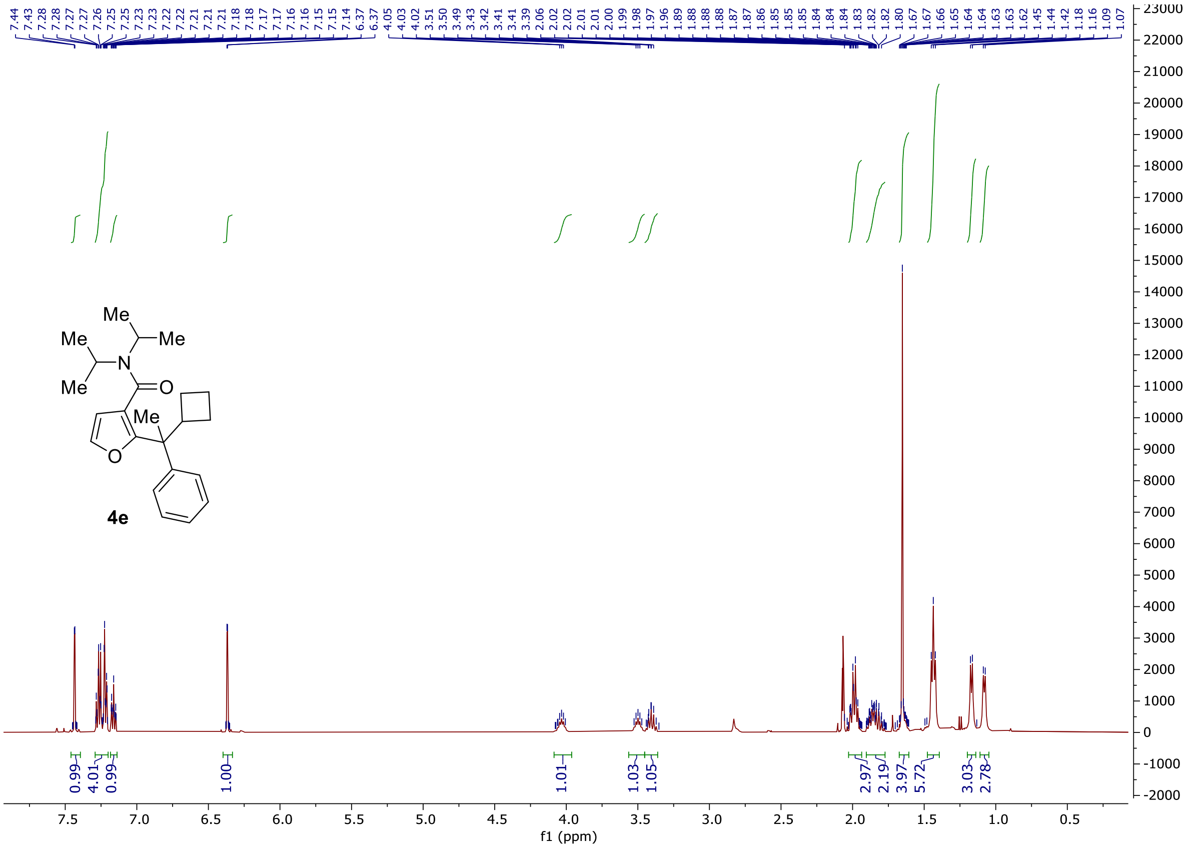

7.44 7.43 7.28 7.28 7.27 7.27 7.26 7.25 7.25 7.23 7.23 7.22 7.22 7.21 7.21 7.21 7.18 7.18 7.17 7.17 7.16 7.16 7.15 7.15 7.14 6.37
4.05 4.03 4.02 3.51 3.50 3.49 3.43 3.42 3.41 3.41 3.39 2.06 2.02 2.02 2.01 2.01 2.00 1.99 1.98 1.97 1.96 1.89 1.88 1.88 1.88 1.87 1.86 1.85 1.85 1.85 1.84 1.84 1.84 1.83 1.82 1.82 1.80 1.67 1.67 1.66 1.65 1.64 1.64 1.63 1.63 1.62 1.45 1.44 1.42 1.18 1.16 1.09 1.07
Me
Me
N
Me
Me
O
Me
O
4e
0.99
4.01
0.99
1.00
1.01
1.03
1.05
2.97
2.19
3.97
5.72
3.03
2.78
f1 (ppm)

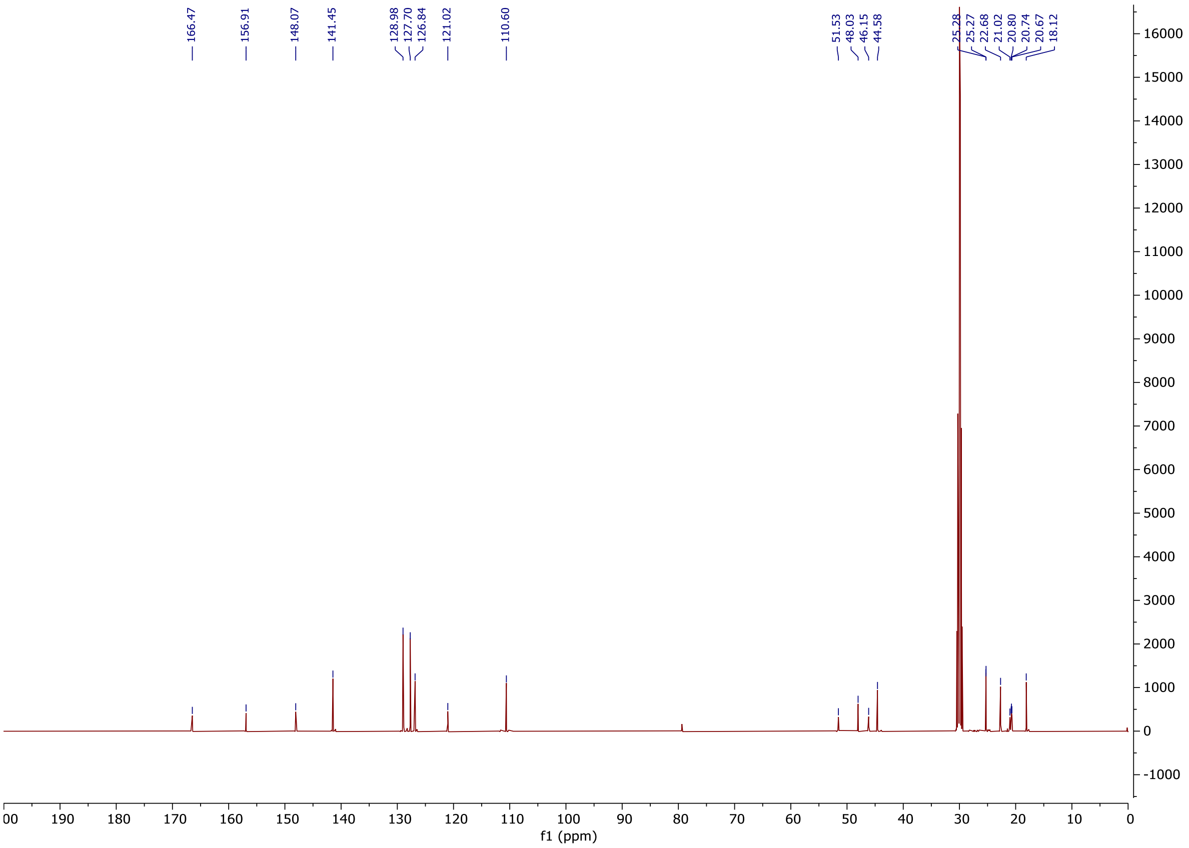

166.47
156.91
148.07
141.45
128.98
127.70
126.84
121.02
110.60
51.53
48.03
46.15
44.58
25.28
25.27
22.68
21.02
20.80
20.74
20.67
18.12
f1 (ppm)

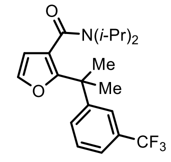

4f

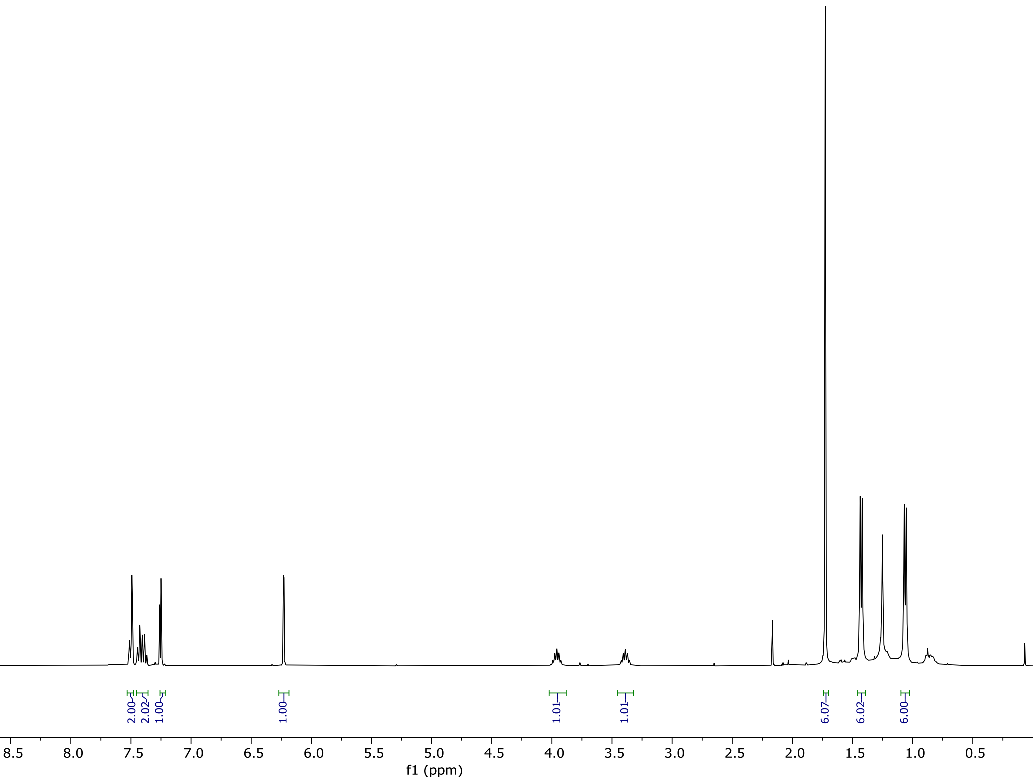
2.00
2.02
1.00
1.00
1.01
1.01
6.07
6.02
6.00
9.5 9.0 8.5 8.0 7.5 7.0 6.5 6.0 5.5 5.0 4.5 4.0 3.5 3.0 2.5 2.0 1.5 1.0 0.5
f1 (ppm)

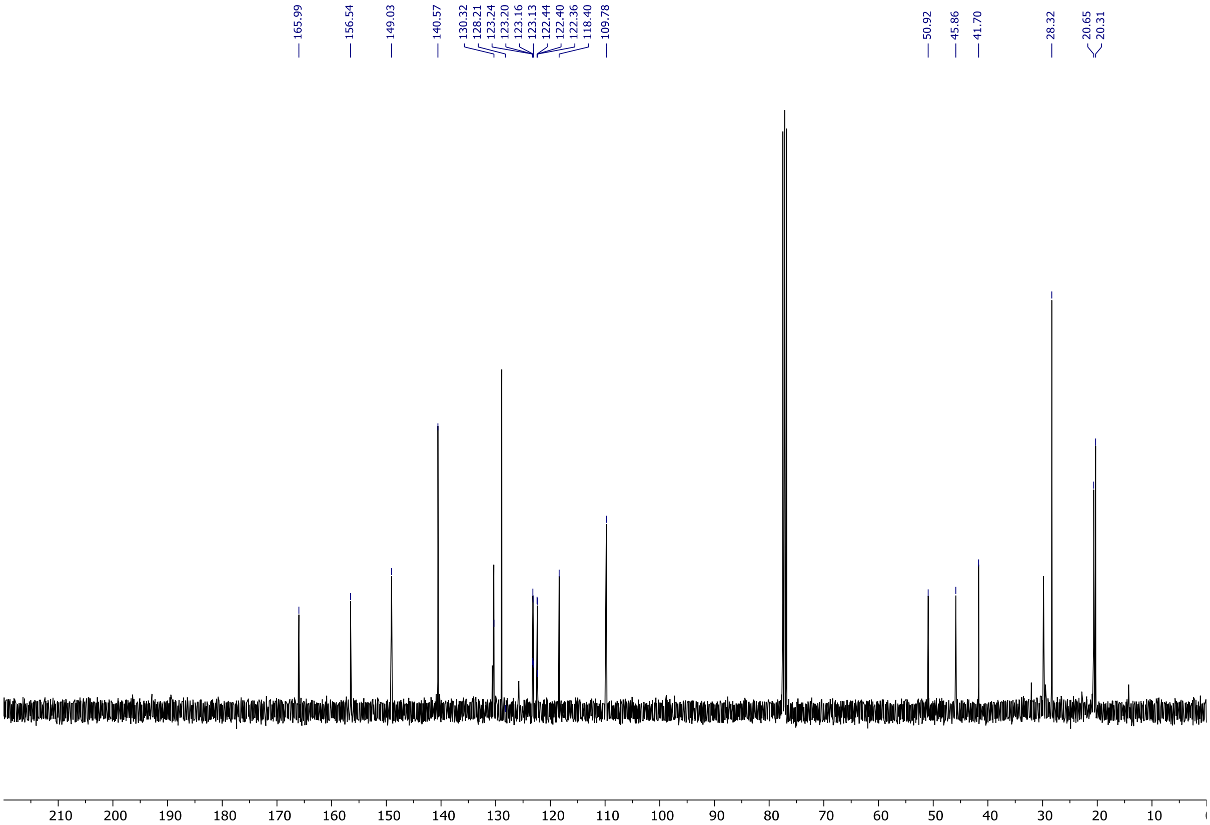
165.99
156.54
149.03
140.57
130.32
128.21
123.24
123.20
123.16
123.13
122.44
122.40
122.36
118.40
109.78
50.92
45.86
41.70
28.32
20.65
20.31
210 200 190 180 170 160 150 140 130 120 110 100 90 80 70 60 50 40 30 20 10
f1 (ppm)

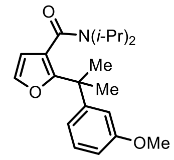
O
N(i-Pr)2
Me
Me
O
OMe

4g

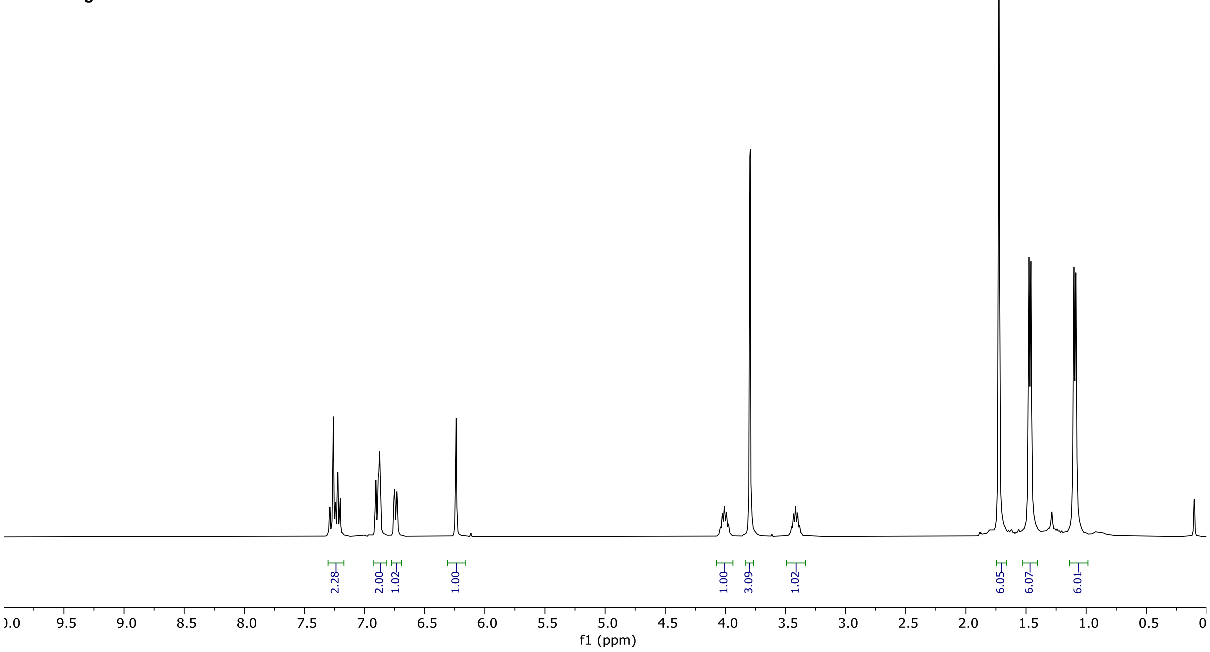
2.28
2.00
1.02
1.00
1.00
3.09
1.02
6.05
6.07
6.01
f1 (ppm)

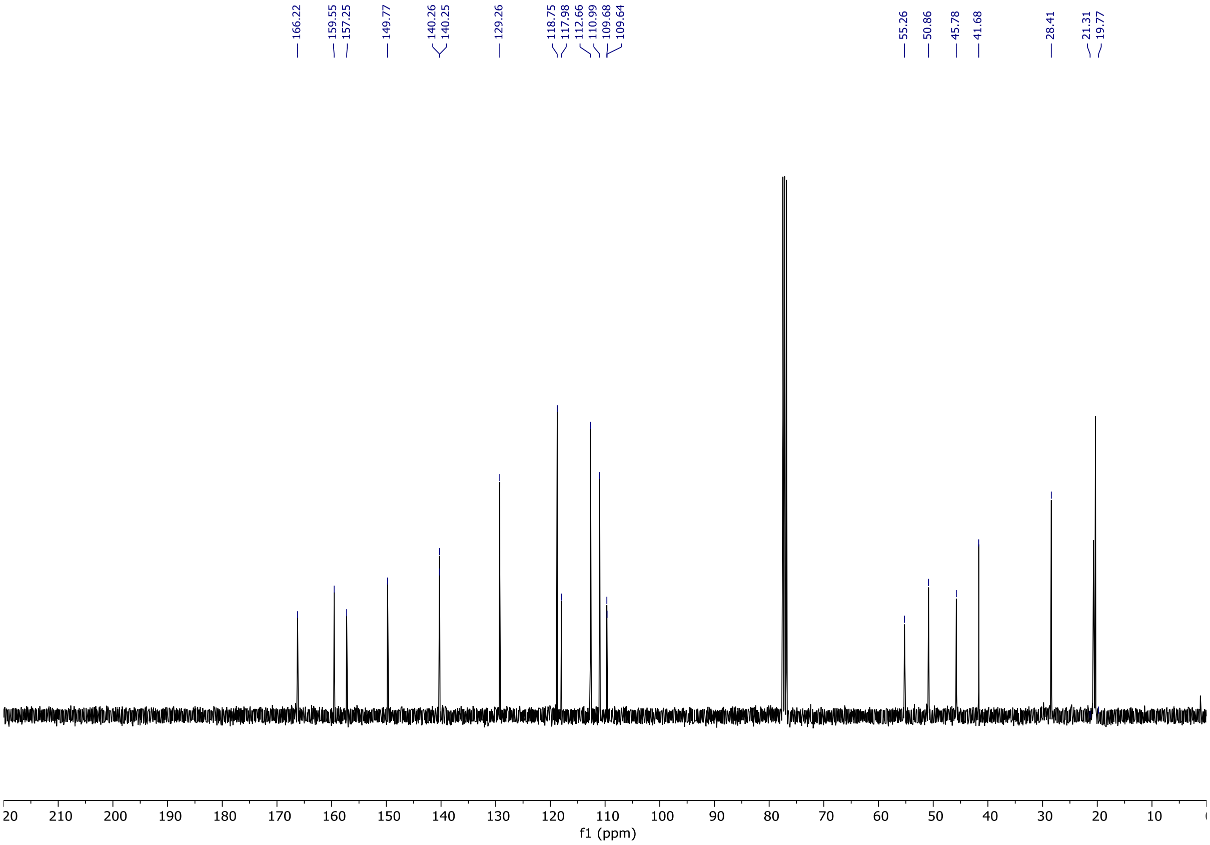
166.22
159.55
157.25
149.77
140.26
140.25
129.26
118.75
117.98
112.66
110.99
109.68
109.64
55.26
50.86
45.78
41.68
28.41
21.31
19.77
f1 (ppm)

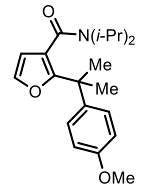

O
N(i-Pr)2
Me
Me
O
OMe

4h

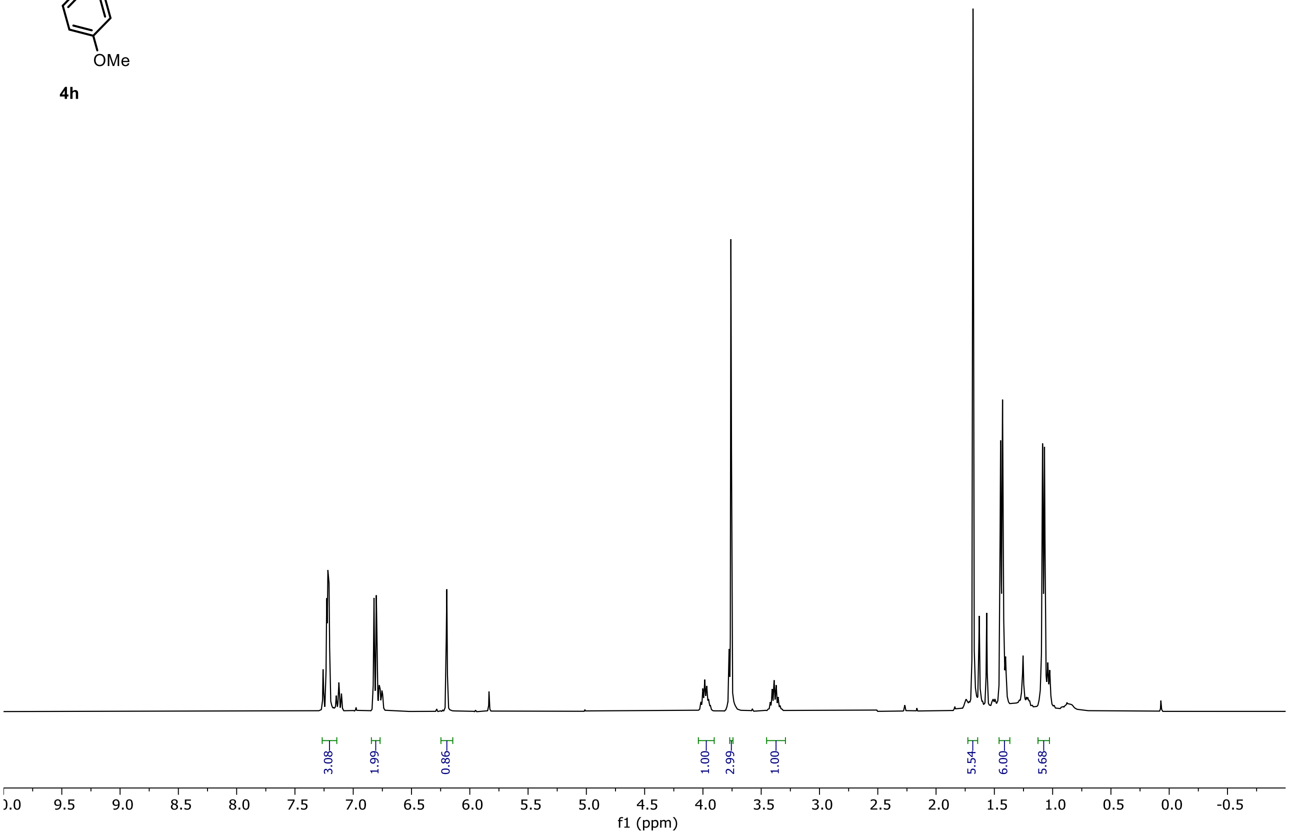

3.08
1.99
0.86
1.00
2.99
1.00
5.54
6.00
5.68
f1 (ppm)

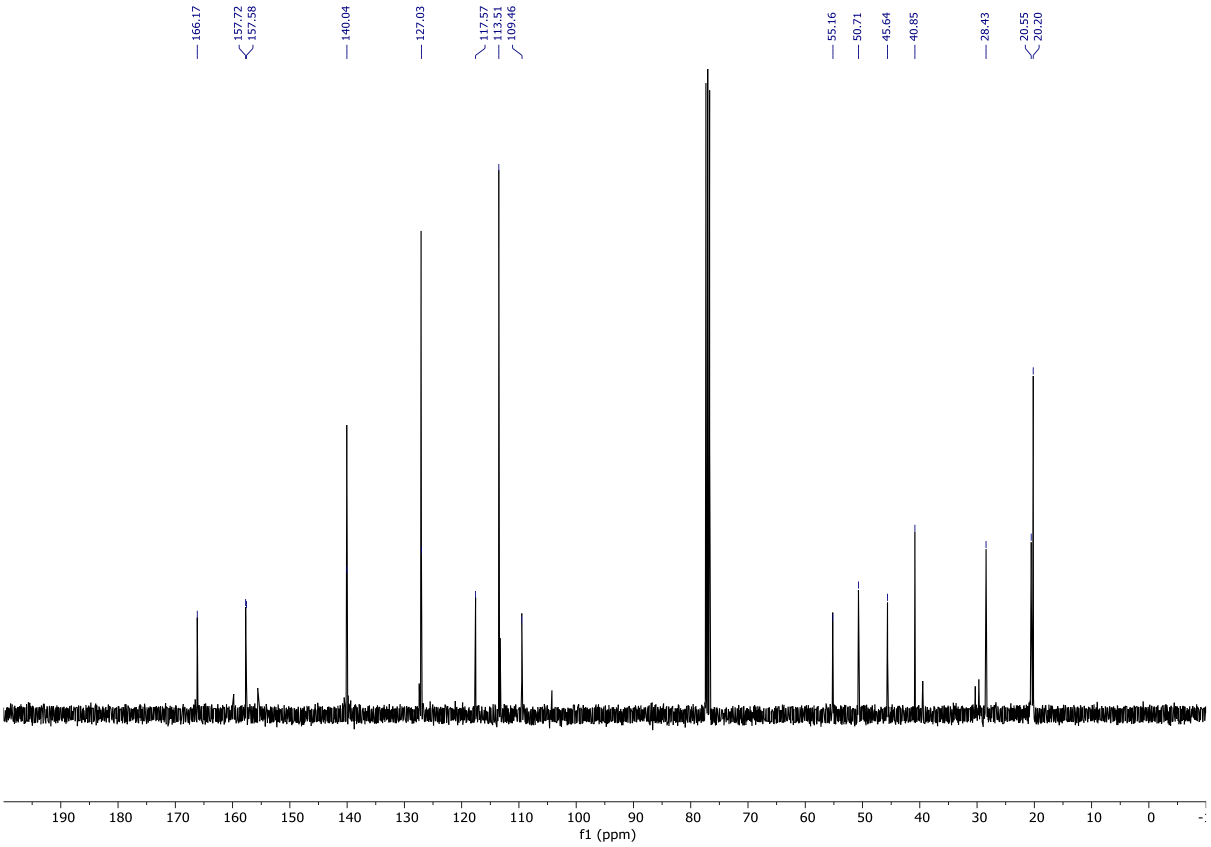

166.17
157.72
157.58
140.04
127.03
117.57
113.51
109.46
55.16
50.71
45.64
40.85
28.43
20.55
20.20
f1 (ppm)

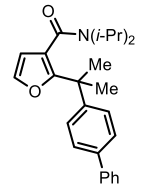
O
N(i-Pr)2
Me
Me
O
Ph

4i

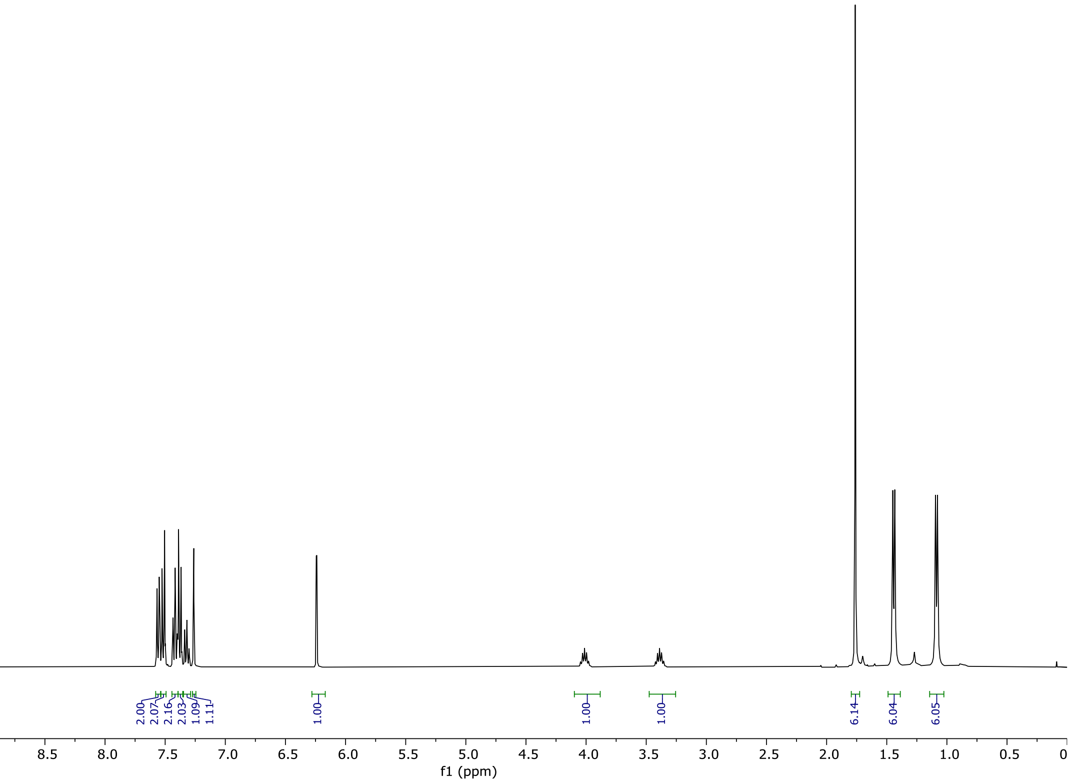
2.00
2.07
2.16
2.03
1.09
1.11
1.00
1.00
1.00
6.14
6.04
6.05
f1 (ppm)

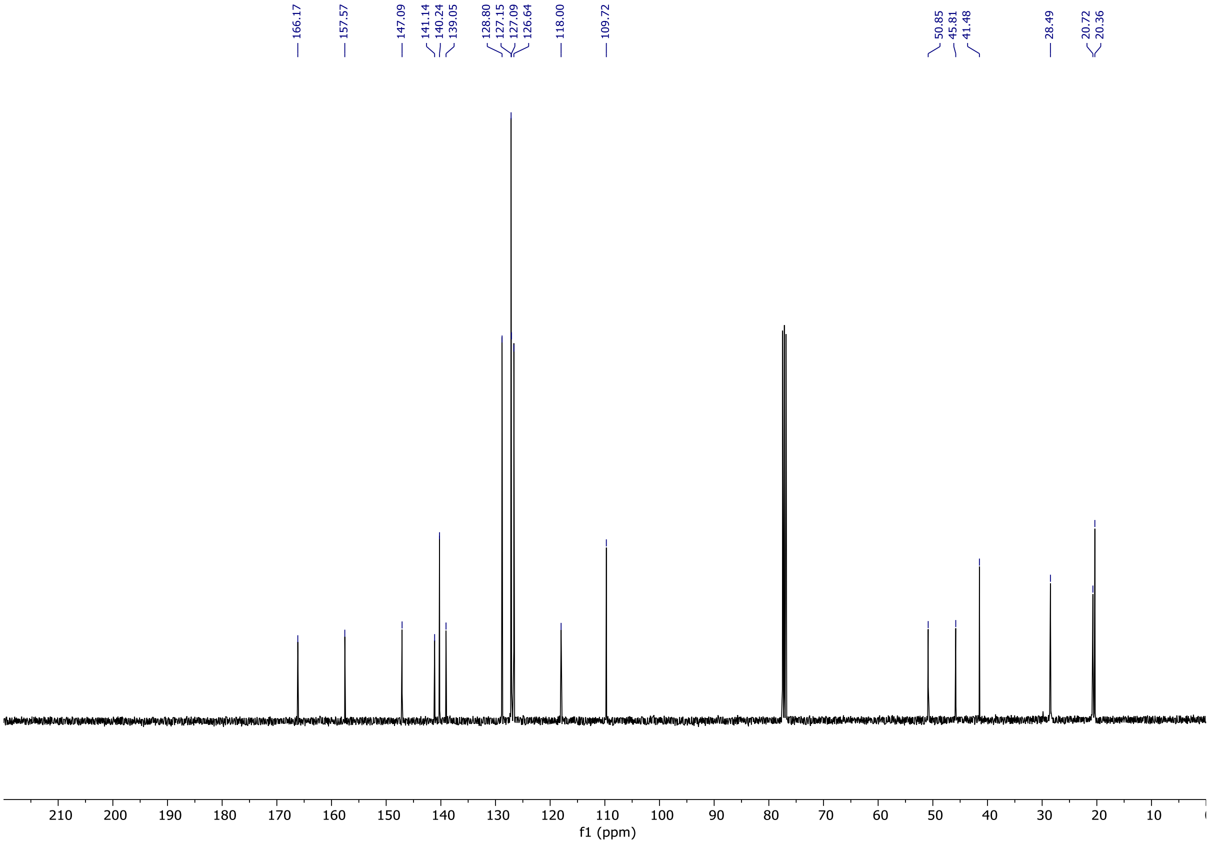
166.17
157.57
147.09
141.14
140.24
139.05
128.80
127.15
127.09
126.64
118.00
109.72
50.85
45.81
41.48
28.49
20.72
20.36
f1 (ppm)

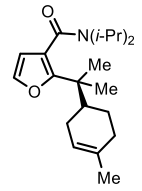
O
N(i-Pr)2
Me
Me
O
Me

4j

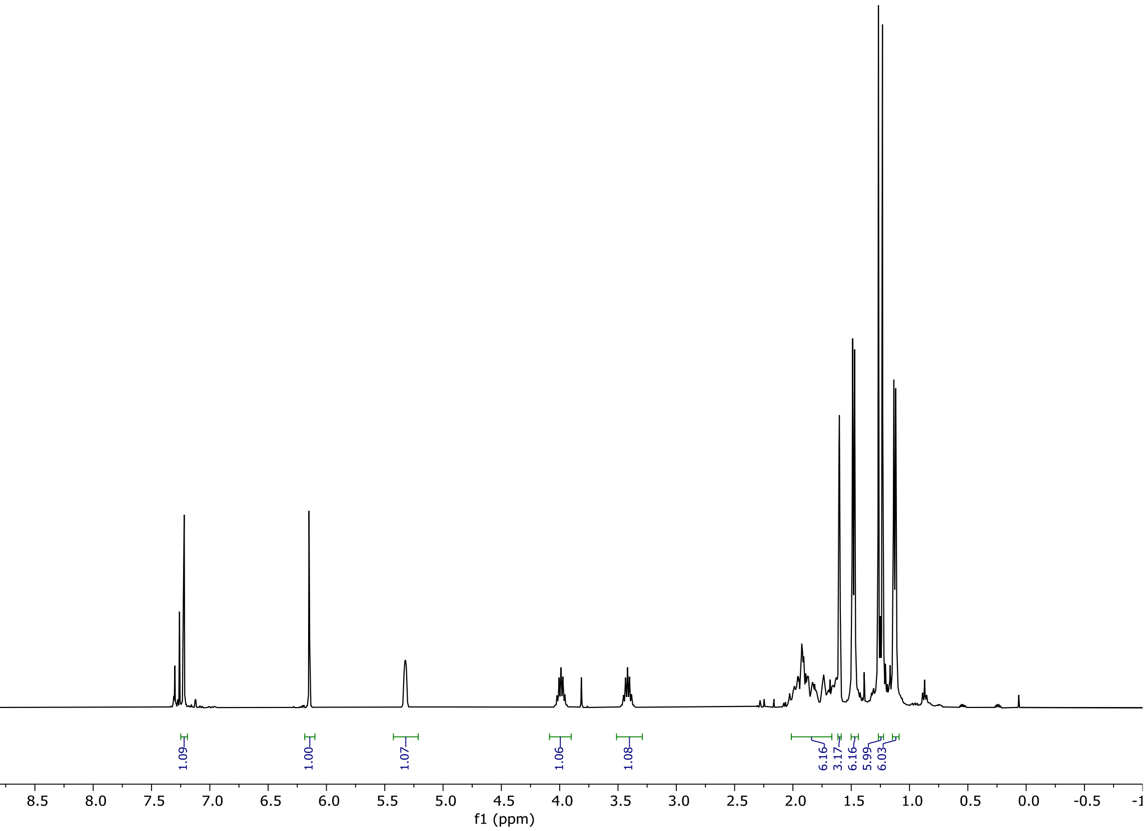
1.09
1.00
1.07
1.06
1.08
6.16
3.17
6.16
5.99
6.03
9.5 9.0 8.5 8.0 7.5 7.0 6.5 6.0 5.5 5.0 4.5 4.0 3.5 3.0 2.5 2.0 1.5 1.0 0.5 0.0 -0.5
f1 (ppm)

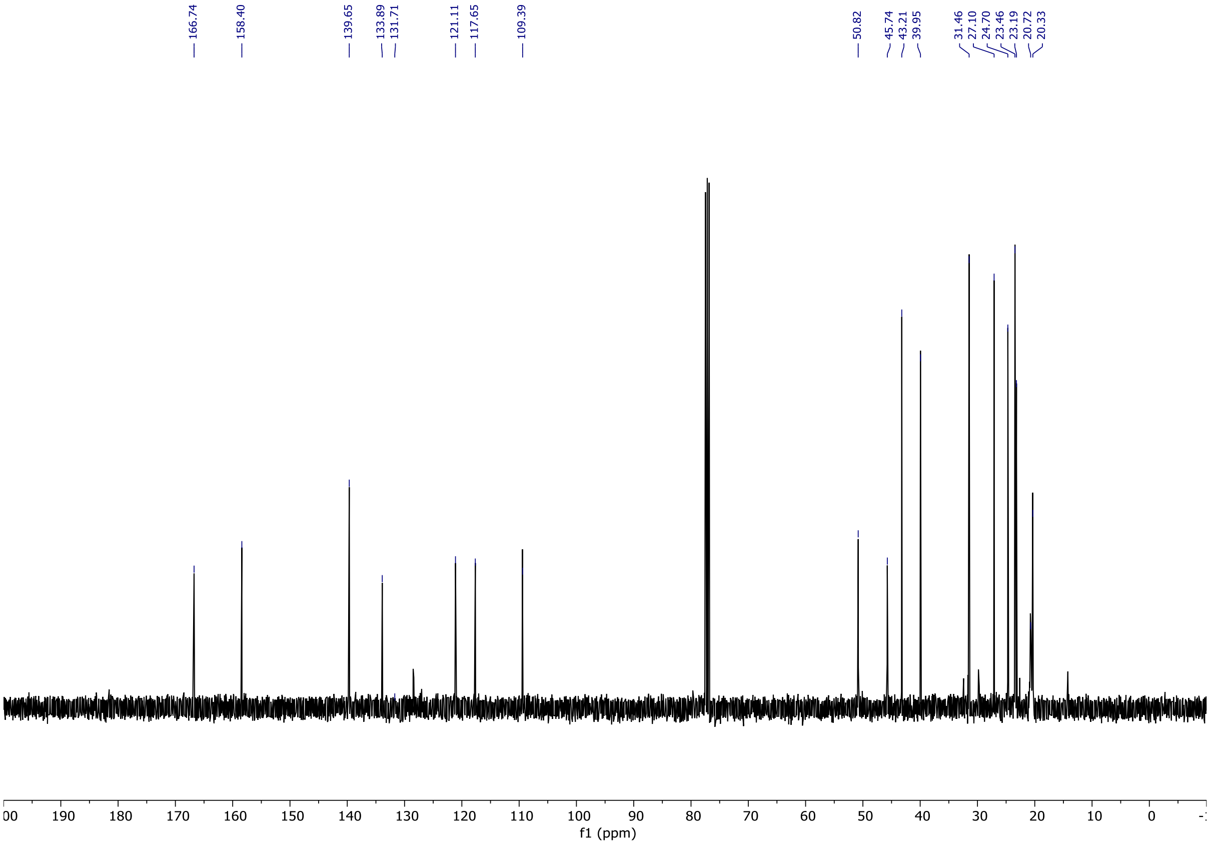
166.74
158.40
139.65
133.89
131.71
121.11
117.65
109.39
50.82
45.74
43.21
39.95
31.46
27.10
24.70
23.46
23.19
20.72
20.33
190 180 170 160 150 140 130 120 110 100 90 80 70 60 50 40 30 20 10 0
f1 (ppm)

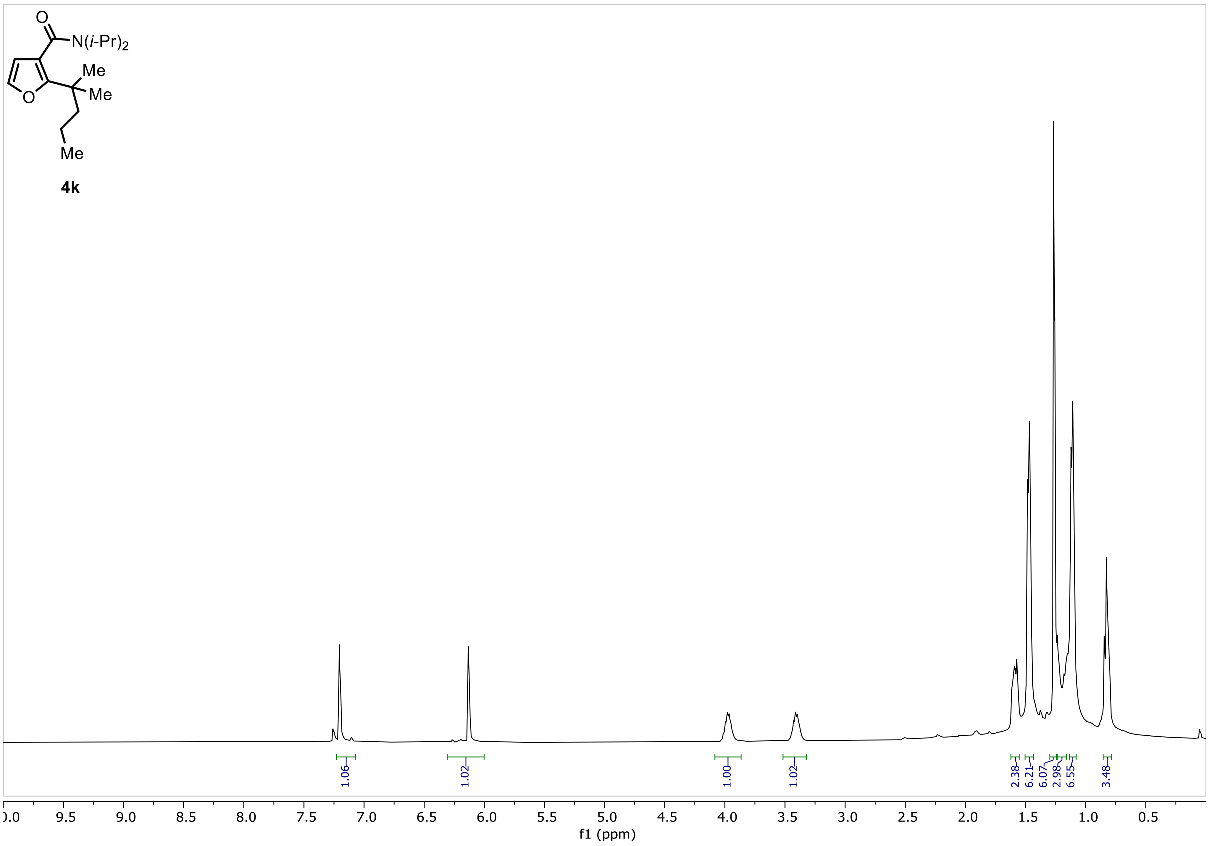
O
N(i-Pr)2
Me
Me
O
Me
4k
1.06
1.02
1.00
1.02
2.38
6.21
6.07
2.98
6.55
3.48
10.0 9.5 9.0 8.5 8.0 7.5 7.0 6.5 6.0 5.5 5.0 4.5 4.0 3.5 3.0 2.5 2.0 1.5 1.0 0.5
f1 (ppm)

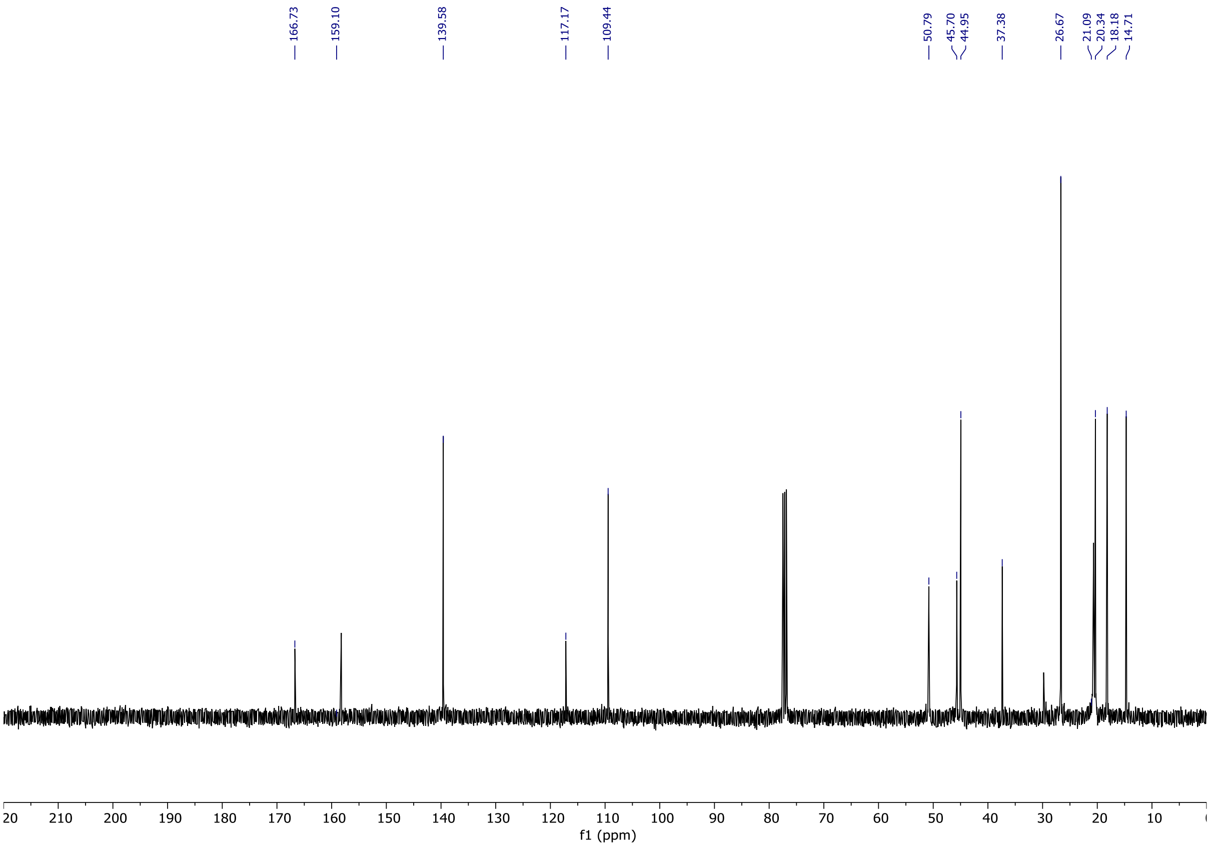
166.73
159.10
139.58
117.17
109.44
50.79
45.70
44.95
37.38
26.67
21.09
20.34
18.18
14.71
220 210 200 190 180 170 160 150 140 130 120 110 100 90 80 70 60 50 40 30 20 10
f1 (ppm)

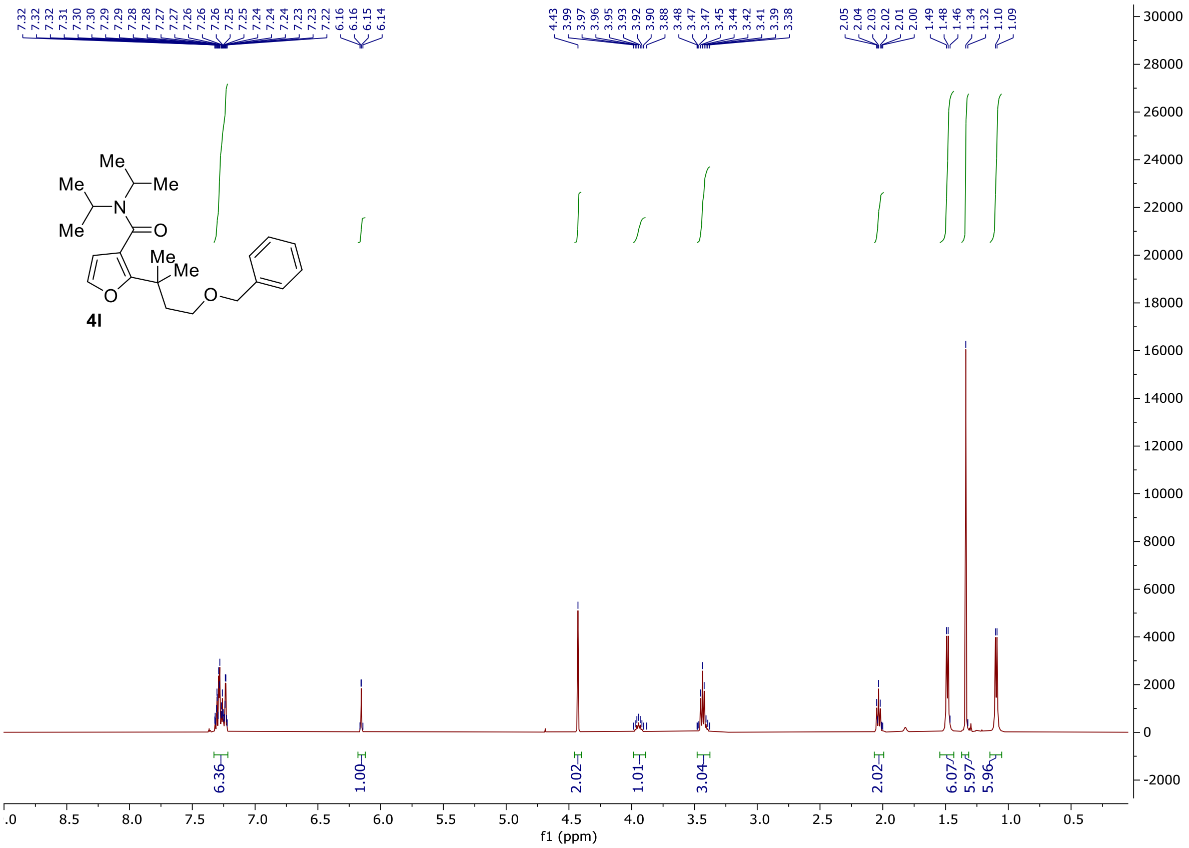
4l

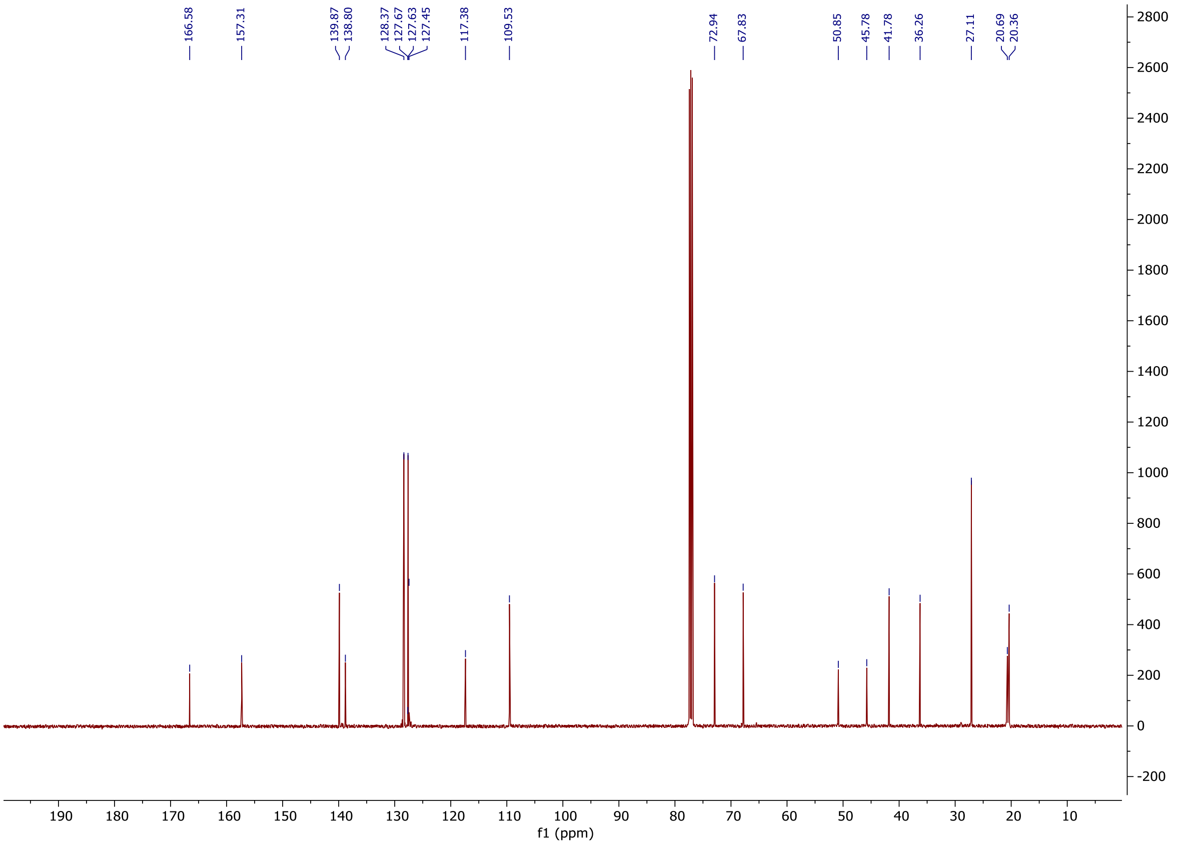

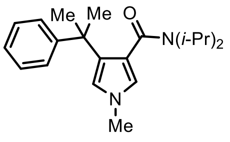
Me
Me
O
N(i-Pr)2
N
Me

6a

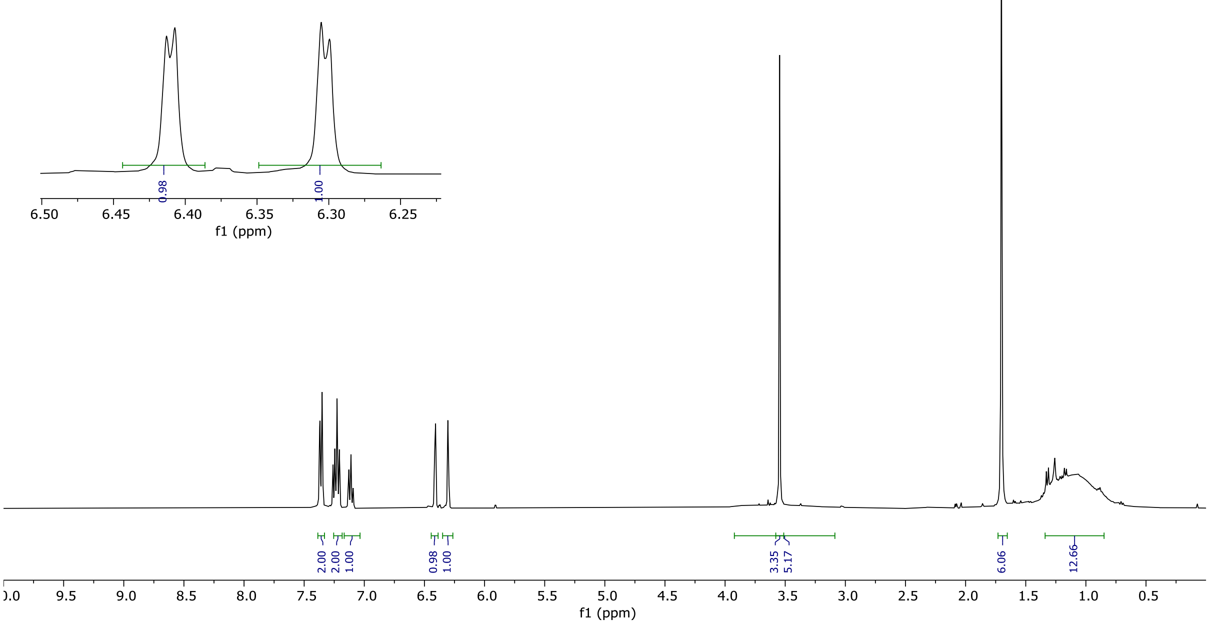
0.98
1.00
6.50
6.45
6.40
6.35
6.30
6.25
f1 (ppm)
2.00
2.00
1.00
0.98
1.00
3.35
5.17
6.06
12.66
f1 (ppm)

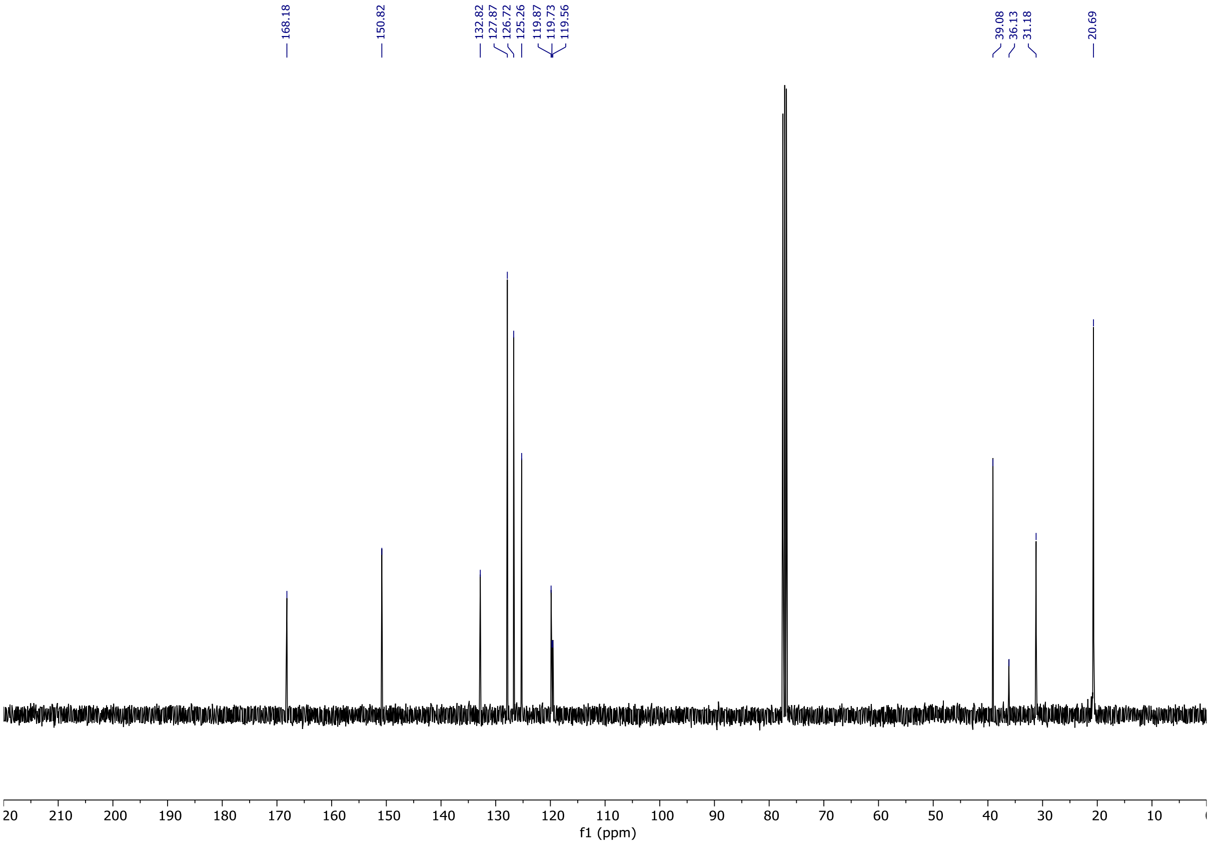
168.18
150.82
132.82
127.87
126.72
125.26
119.87
119.73
119.56
39.08
36.13
31.18
20.69
f1 (ppm)

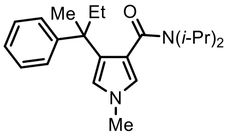
Me
Et
O
N(i-Pr)2
N
Me

6b

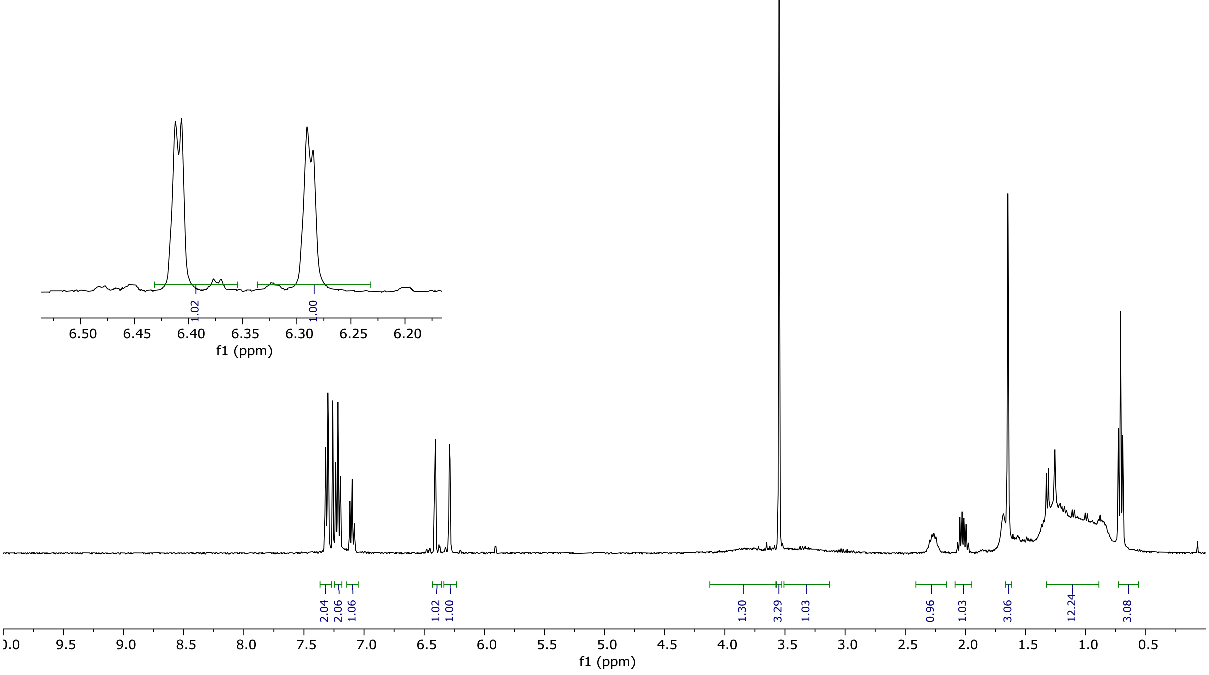
1.02
1.00
f1 (ppm)
2.04
2.06
1.06
1.02
1.00
1.30
3.29
1.03
0.96
1.03
3.06
12.24
3.08
f1 (ppm)

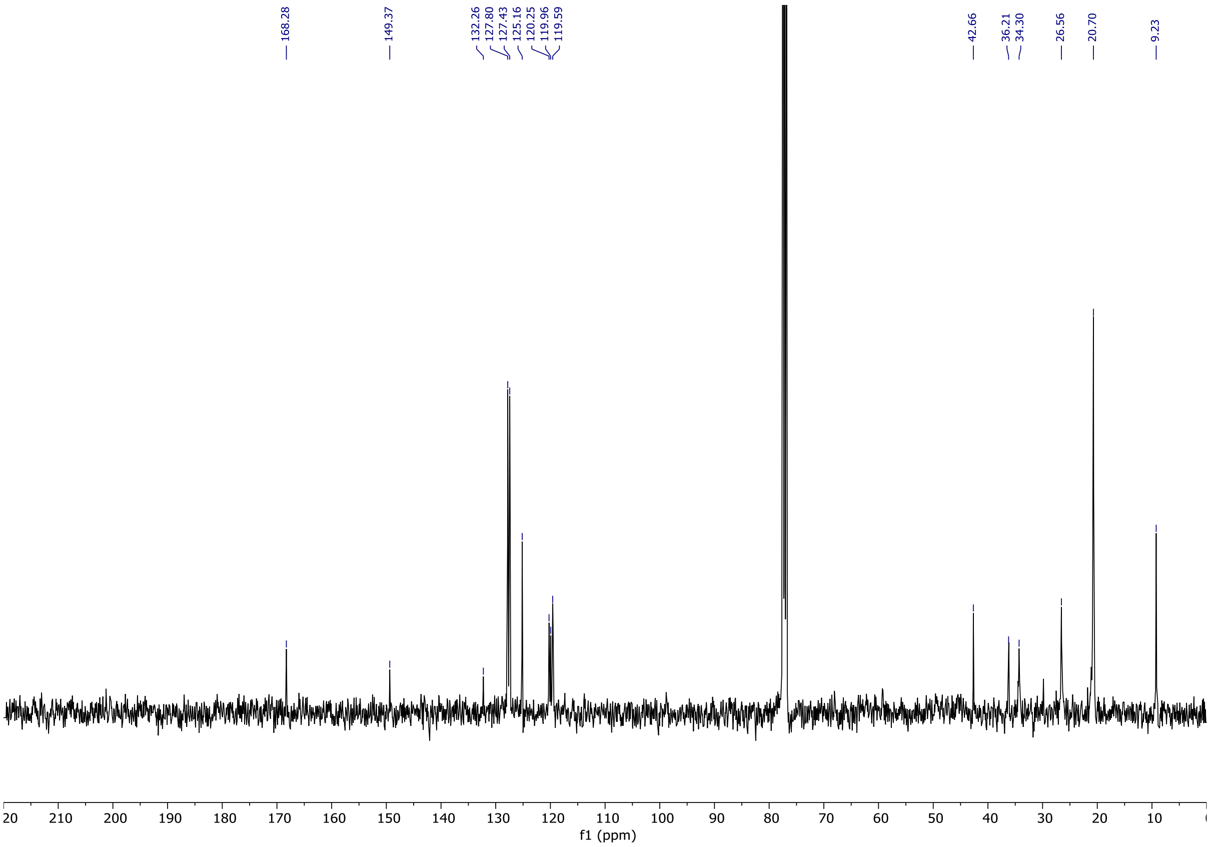
168.28
149.37
132.26
127.80
127.43
125.16
120.25
119.96
119.59
42.66
36.21
34.30
26.56
20.70
9.23
f1 (ppm)

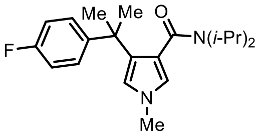
Me
Me
O
N(i-Pr)2
F
N
Me

6c

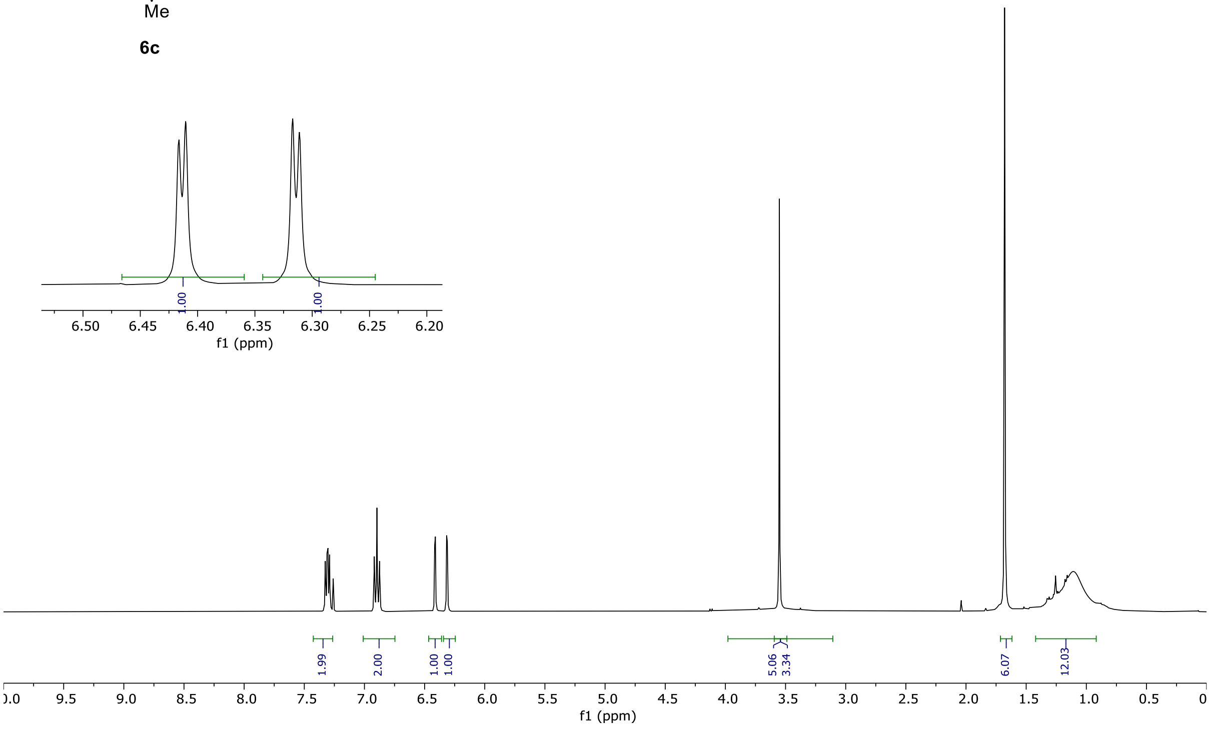
6.50 6.45 6.40 6.35 6.30 6.25 6.20
f1 (ppm)
1.00
1.00
1.99
2.00
1.00
1.00
5.06
3.34
6.07
12.03
10.0 9.5 9.0 8.5 8.0 7.5 7.0 6.5 6.0 5.5 5.0 4.5 4.0 3.5 3.0 2.5 2.0 1.5 1.0 0.5 0
f1 (ppm)

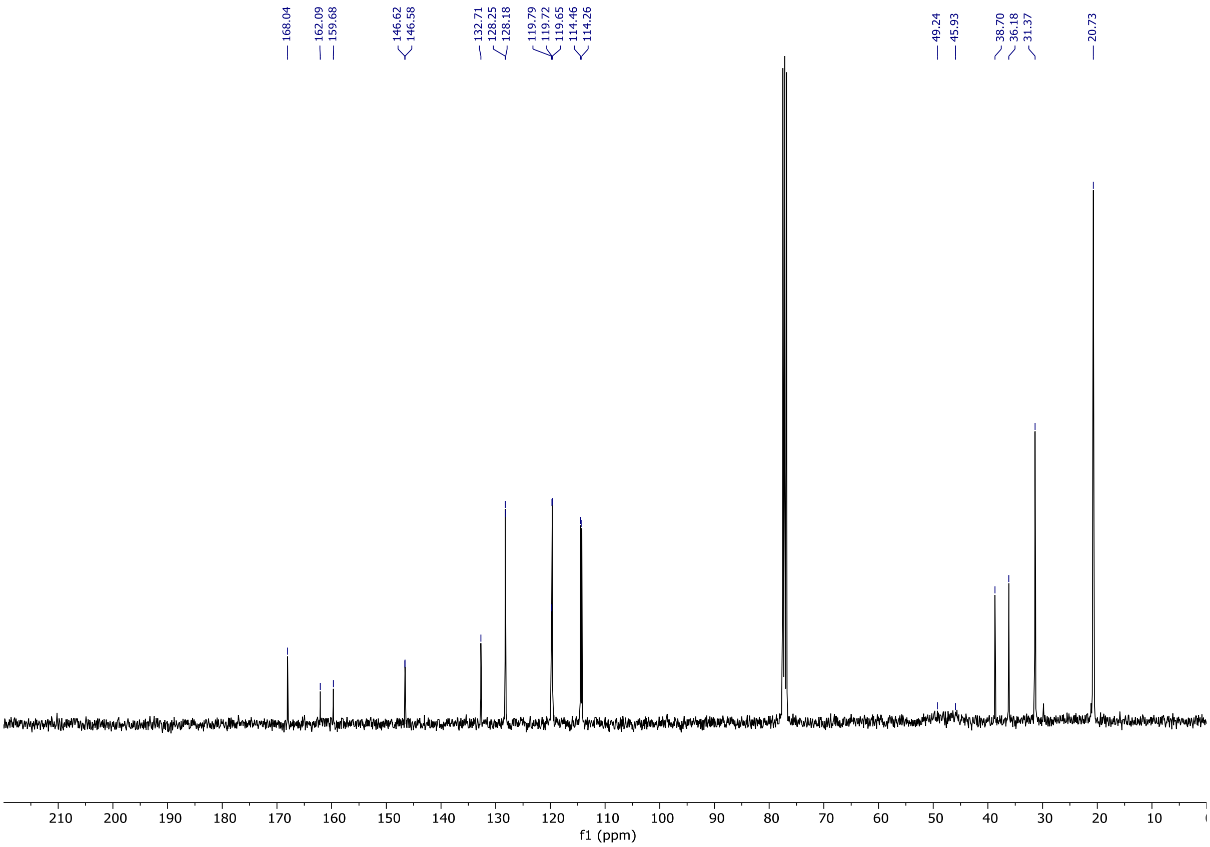
168.04
162.09
159.68
146.62
146.58
132.71
128.25
128.18
119.79
119.72
119.65
114.46
114.26
49.24
45.93
38.70
36.18
31.37
20.73
210 200 190 180 170 160 150 140 130 120 110 100 90 80 70 60 50 40 30 20 10 0
f1 (ppm)

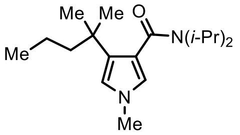
Me
Me
O
N(i-Pr)2
Me
N
Me

6d

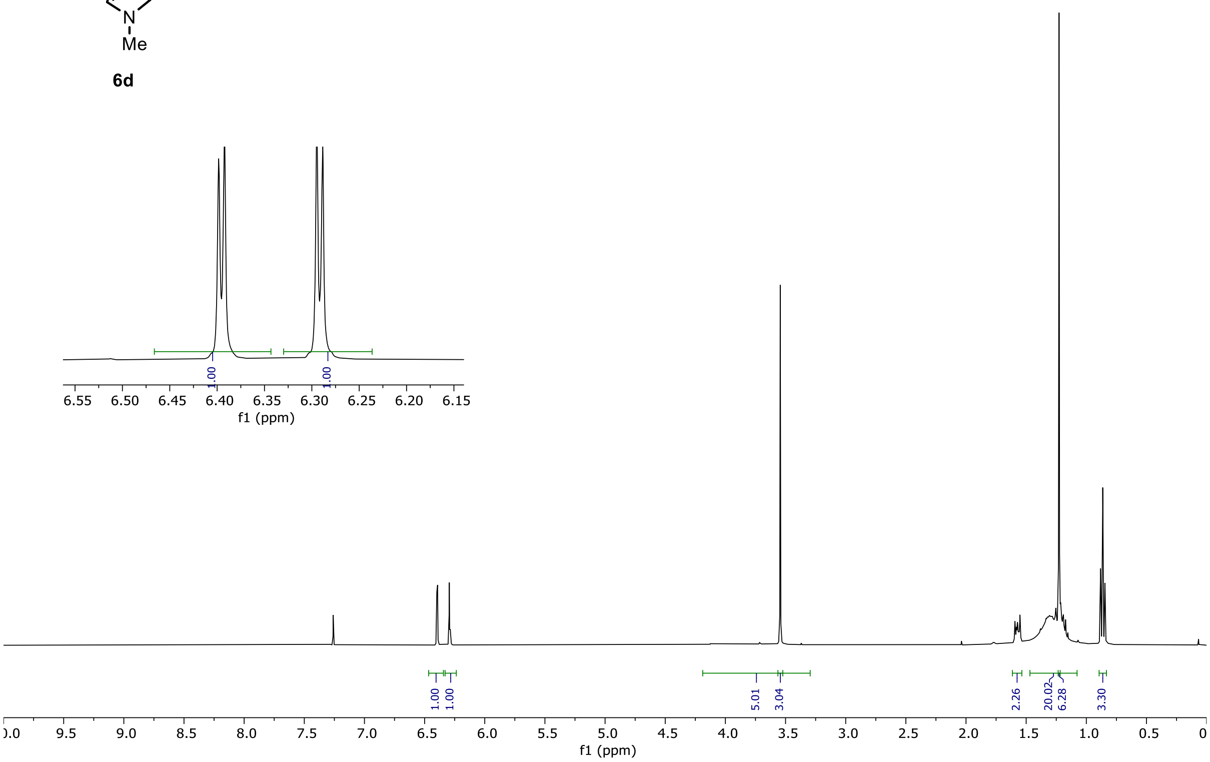
1.00
1.00
6.55 6.50 6.45 6.40 6.35 6.30 6.25 6.20 6.15
f1 (ppm)
1.00
1.00
5.01
3.04
2.26
20.02
6.28
3.30
9.5 9.0 8.5 8.0 7.5 7.0 6.5 6.0 5.5 5.0 4.5 4.0 3.5 3.0 2.5 2.0 1.5 1.0 0.5 0
f1 (ppm)

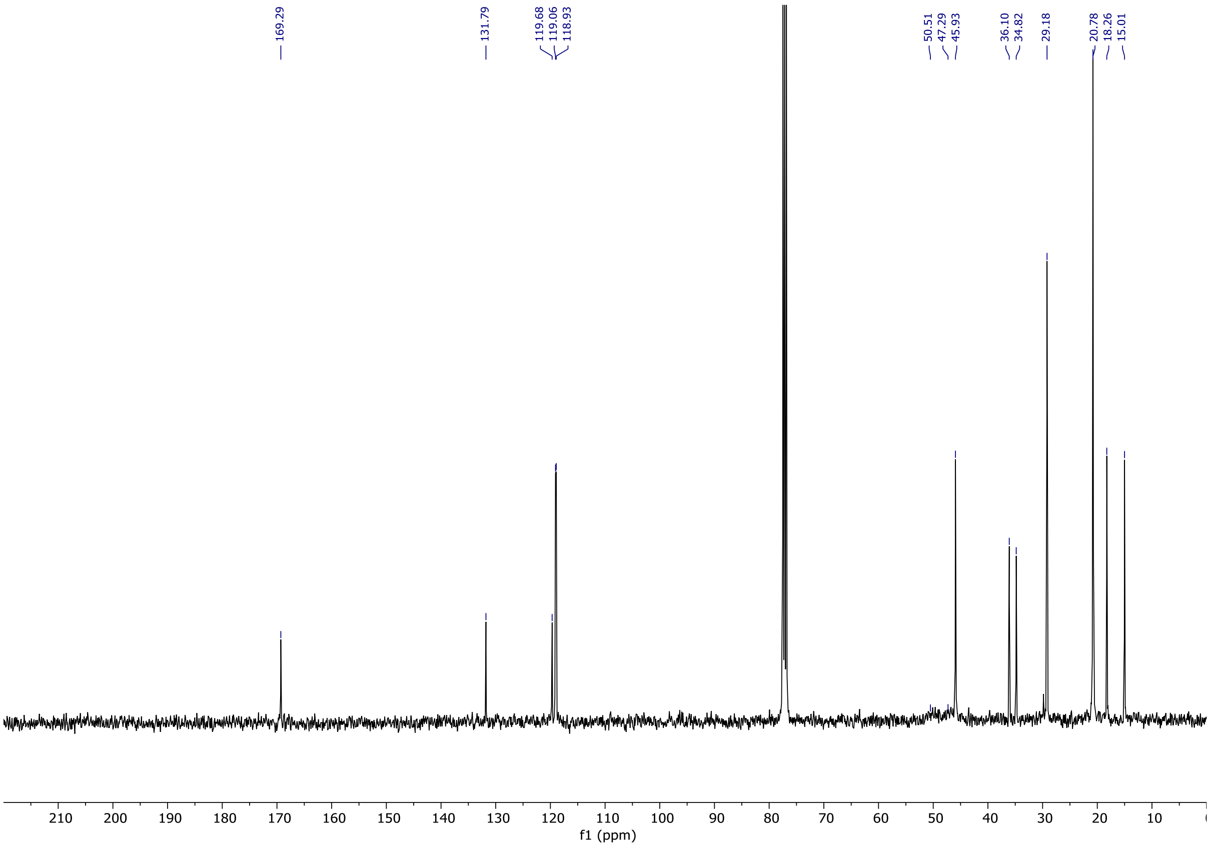
169.29
131.79
119.68
119.06
118.93
50.51
47.29
45.93
36.10
34.82
29.18
20.78
18.26
15.01
210 200 190 180 170 160 150 140 130 120 110 100 90 80 70 60 50 40 30 20 10
f1 (ppm)

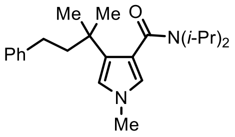
Me
Me
O
N(i-Pr)2
Ph
N
Me

6e

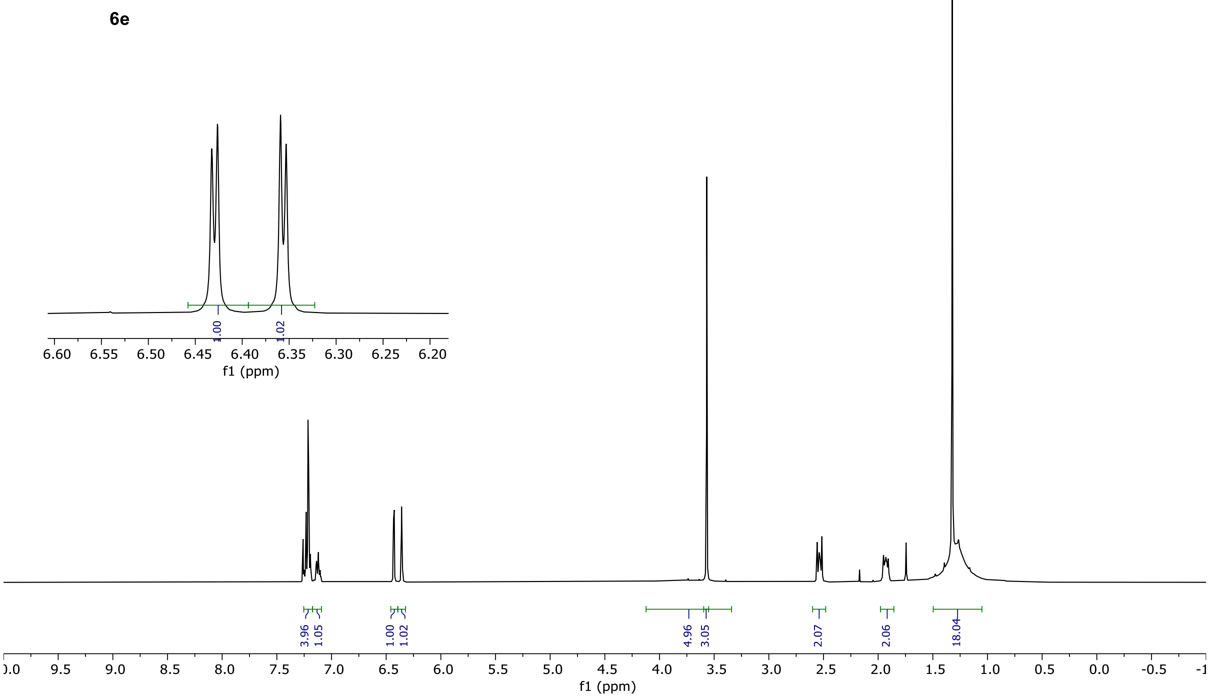
1.00
1.02
6.60 6.55 6.50 6.45 6.40 6.35 6.30 6.25 6.20
f1 (ppm)
3.96
1.05
1.00
1.02
4.96
3.05
2.07
2.06
18.04
9.5 9.0 8.5 8.0 7.5 7.0 6.5 6.0 5.5 5.0 4.5 4.0 3.5 3.0 2.5 2.0 1.5 1.0 0.5 0.0 -0.5
f1 (ppm)

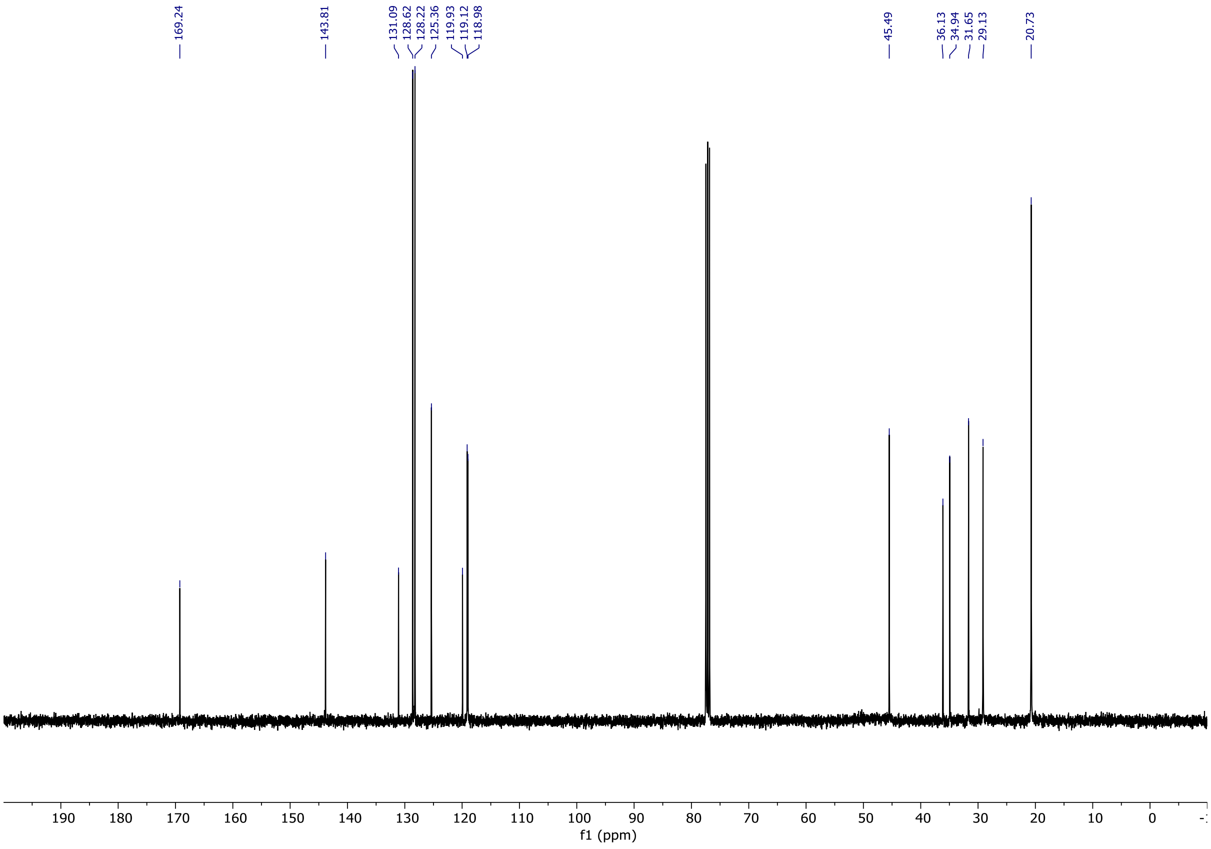
169.24
143.81
131.09
128.62
128.22
125.36
119.93
119.12
118.98
45.49
36.13
34.94
31.65
29.13
20.73
190 180 170 160 150 140 130 120 110 100 90 80 70 60 50 40 30 20 10 0
f1 (ppm)

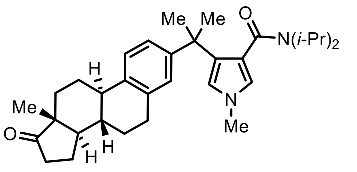
Me
Me
O
N(i-Pr)2
H
Me
N
Me
O
H
H

6f

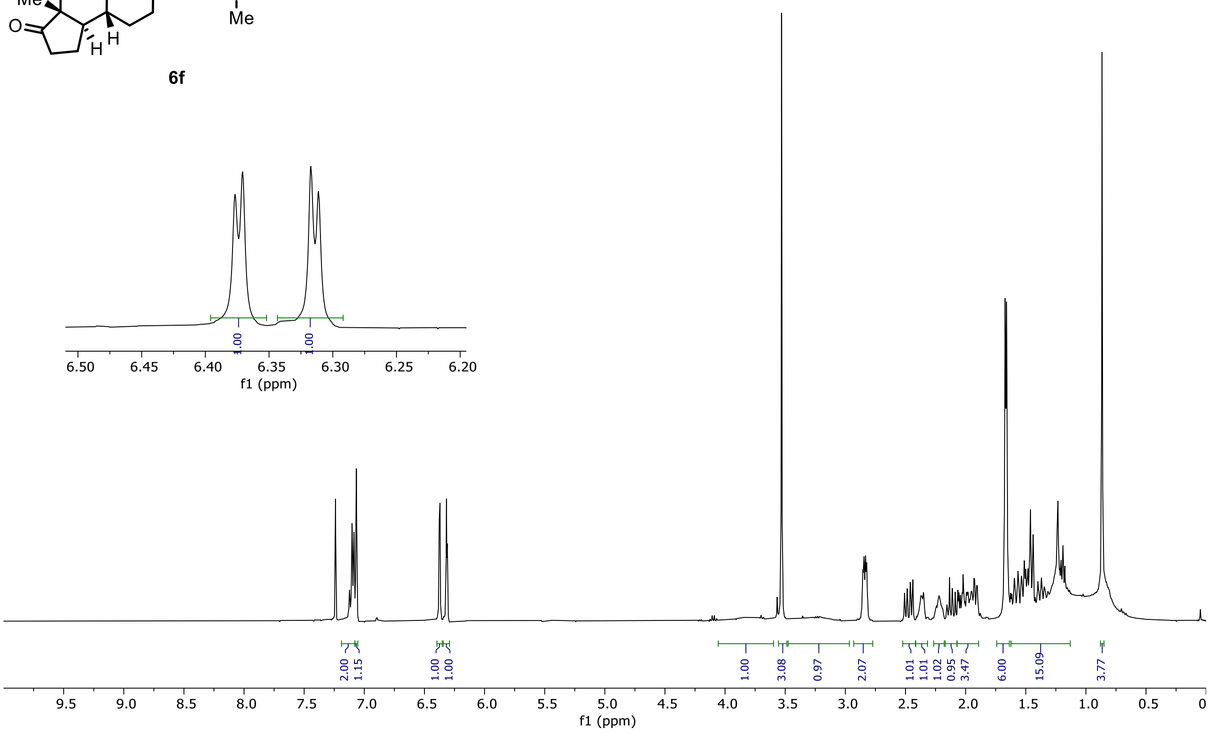
1.00
1.00
6.50 6.45 6.40 6.35 6.30 6.25 6.20
f1 (ppm)
2.00
1.15
1.00
1.00
1.00
3.08
0.97
2.07
1.01
1.01
1.02
0.95
3.47
6.00
15.09
3.77
9.5 9.0 8.5 8.0 7.5 7.0 6.5 6.0 5.5 5.0 4.5 4.0 3.5 3.0 2.5 2.0 1.5 1.0 0.5 0
f1 (ppm)

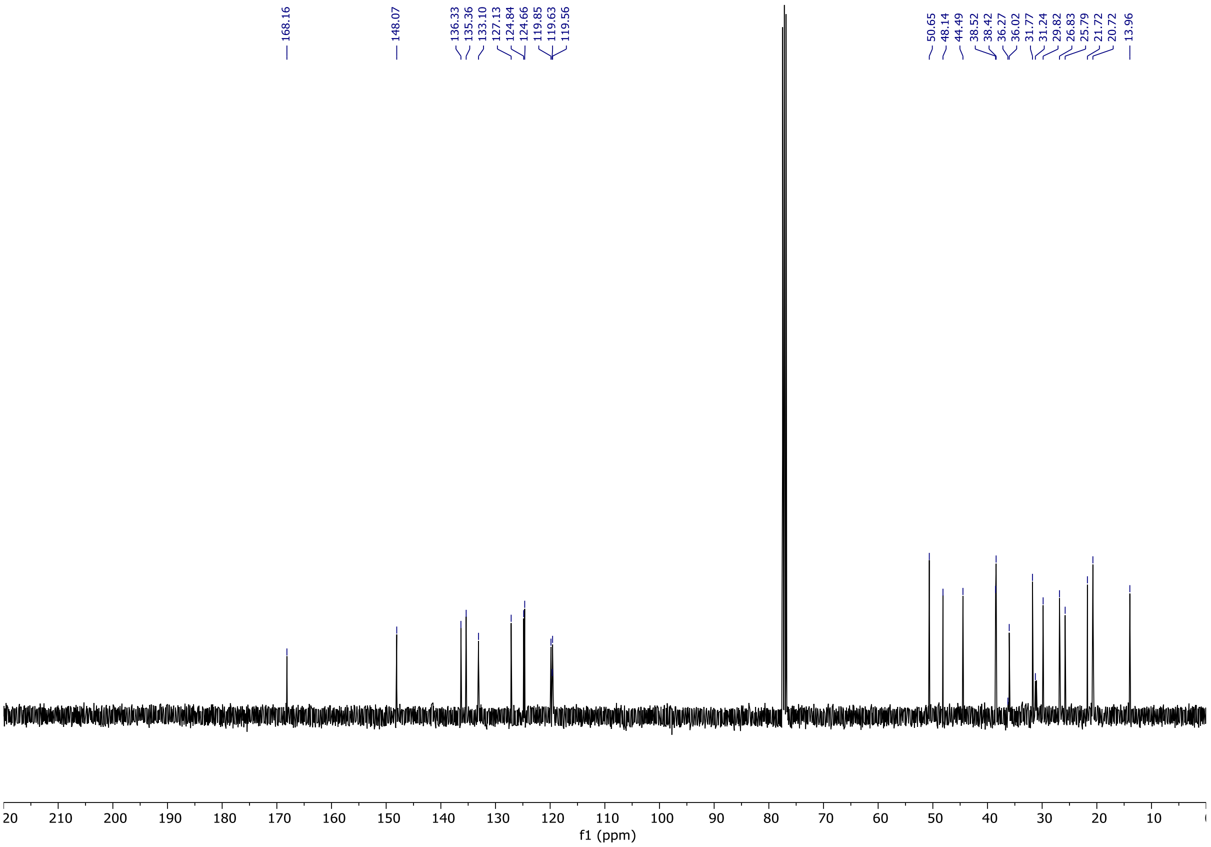
168.16
148.07
136.33
135.36
133.10
127.13
124.84
124.66
119.85
119.63
119.56
50.65
48.14
44.49
38.52
38.42
36.27
36.02
31.77
31.24
29.82
26.83
25.79
21.72
20.72
13.96
20 210 200 190 180 170 160 150 140 130 120 110 100 90 80 70 60 50 40 30 20 10
f1 (ppm)

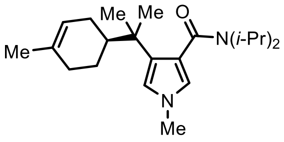
Me
Me
O
N(i-Pr)2
Me
N
Me

6g

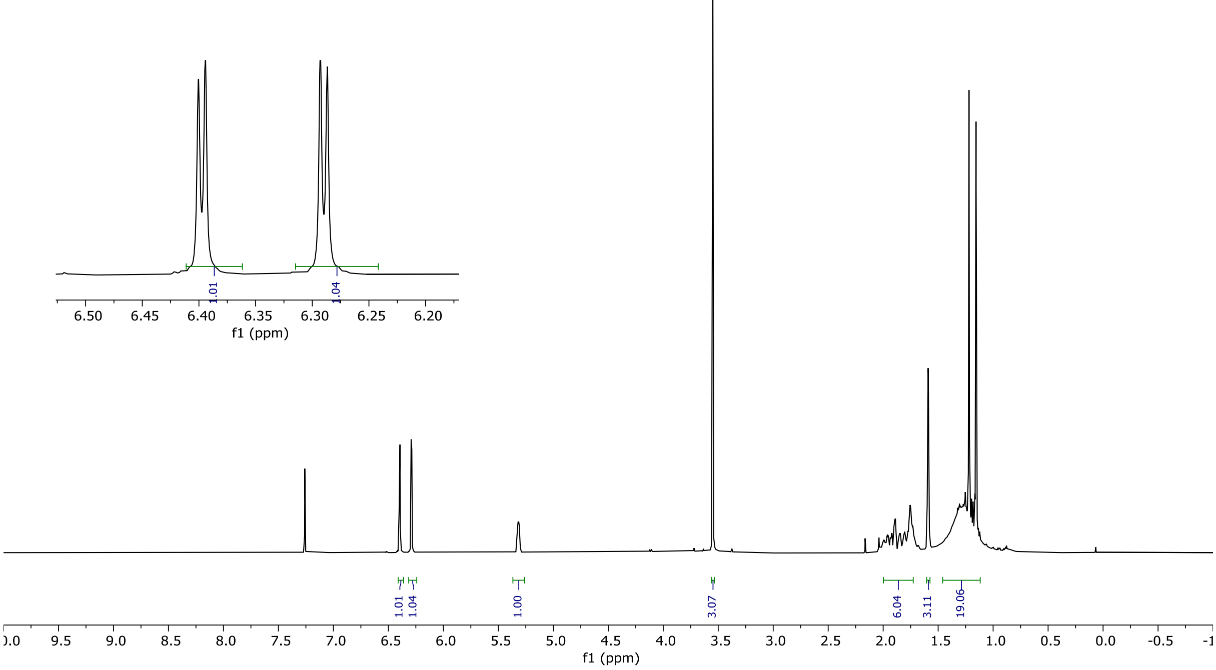
1.01
1.04
6.50 6.45 6.40 6.35 6.30 6.25 6.20
f1 (ppm)
1.01
1.04
1.00
3.07
6.04
3.11
19.06
10.0 9.5 9.0 8.5 8.0 7.5 7.0 6.5 6.0 5.5 5.0 4.5 4.0 3.5 3.0 2.5 2.0 1.5 1.0 0.5 0.0 -0.5 -1
f1 (ppm)

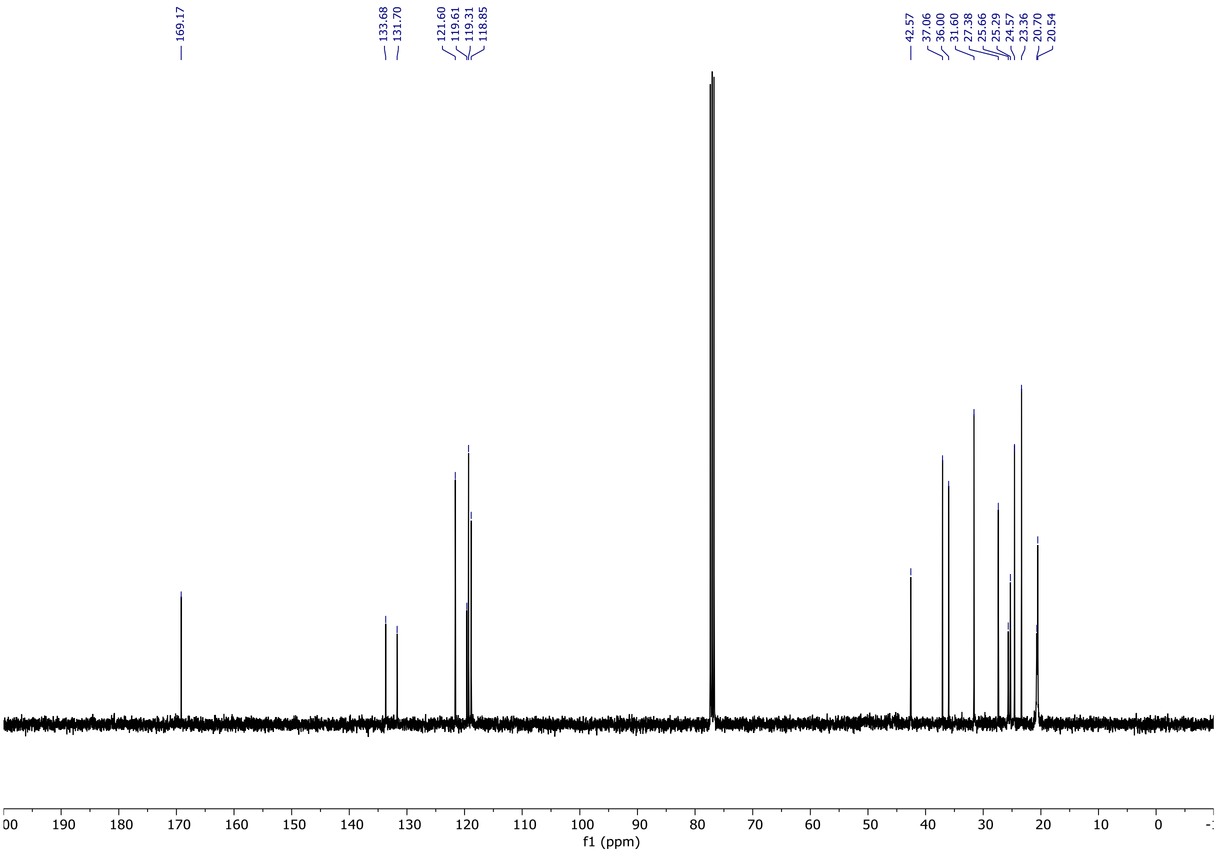
169.17
133.68
131.70
121.60
119.61
119.31
118.85
42.57
37.06
36.00
31.60
27.38
25.66
25.29
24.57
23.36
20.70
20.54
200 190 180 170 160 150 140 130 120 110 100 90 80 70 60 50 40 30 20 10 0
f1 (ppm)

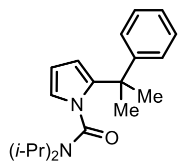

Me
Me
N
O
(i-Pr)2N

6h

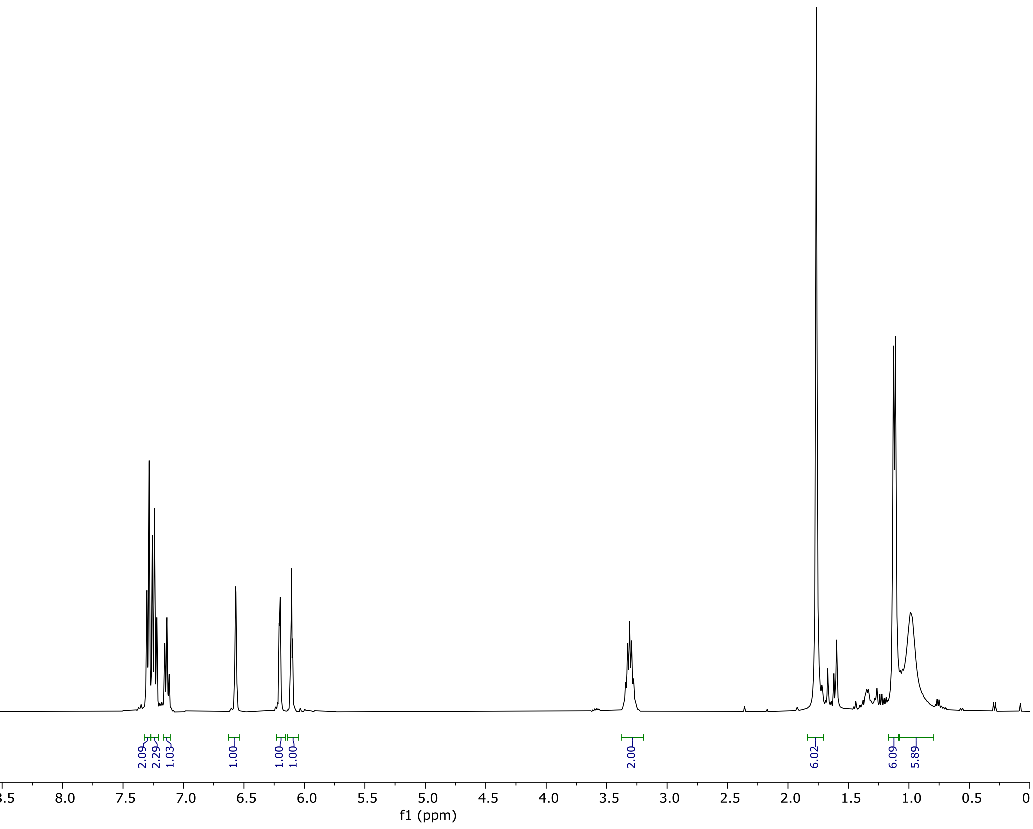

2.09
2.29
1.03
1.00
1.00
1.00
2.00
6.02
6.09
5.89
9.5 9.0 8.5 8.0 7.5 7.0 6.5 6.0 5.5 5.0 4.5 4.0 3.5 3.0 2.5 2.0 1.5 1.0 0.5 0
f1 (ppm)

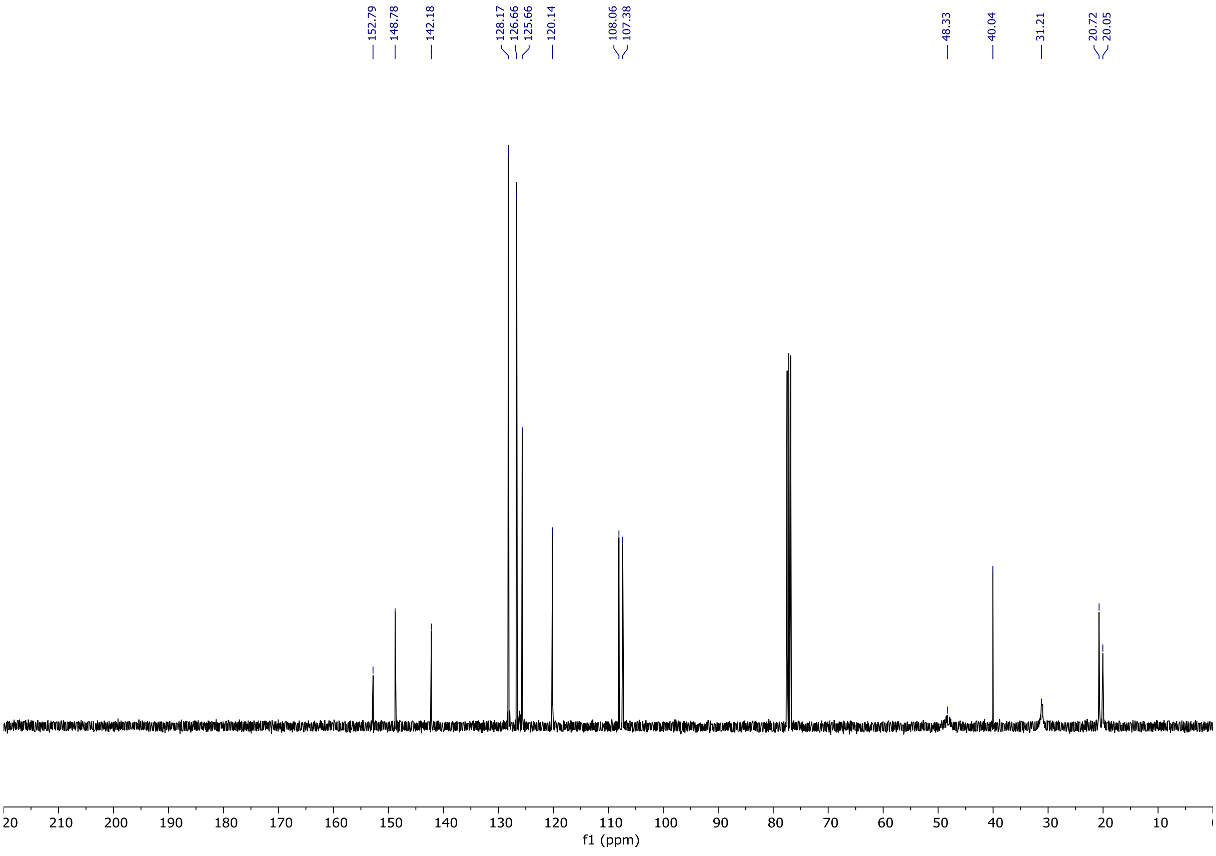

152.79
148.78
142.18
128.17
126.66
125.66
120.14
108.06
107.38
48.33
40.04
31.21
20.72
20.05
20 210 200 190 180 170 160 150 140 130 120 110 100 90 80 70 60 50 40 30 20 10
f1 (ppm)

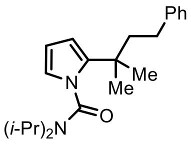
Ph
Me
Me
N
O
(i-Pr)2N

6i

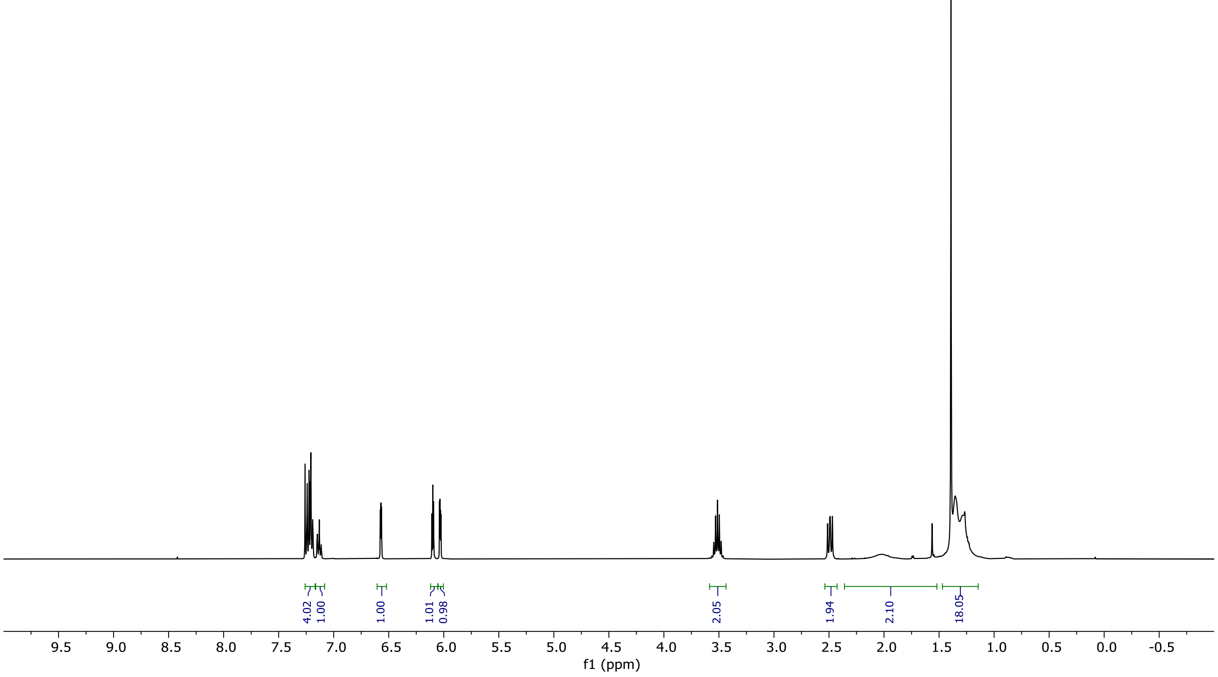
4.02
1.00
1.00
1.01
0.98
2.05
1.94
2.10
18.05
9.5 9.0 8.5 8.0 7.5 7.0 6.5 6.0 5.5 5.0 4.5 4.0 3.5 3.0 2.5 2.0 1.5 1.0 0.5 0.0 -0.5
f1 (ppm)

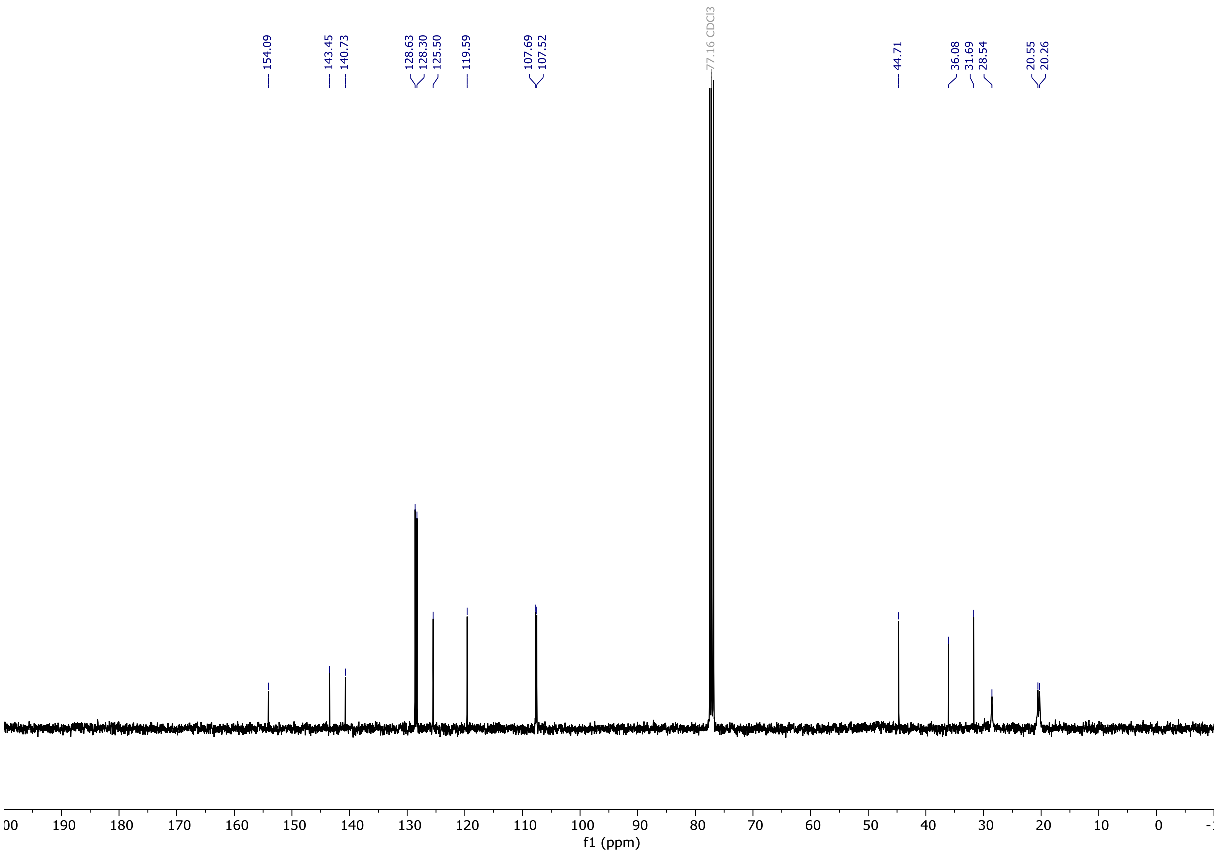
154.09
143.45
140.73
128.63
128.30
125.50
119.59
107.69
107.52
77.16 CDCl3
44.71
36.08
31.69
28.54
20.55
20.26
190 180 170 160 150 140 130 120 110 100 90 80 70 60 50 40 30 20 10 0
f1 (ppm)

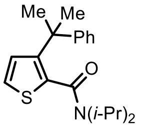
Me
Me
Ph
O
S
N(i-Pr)2

8a

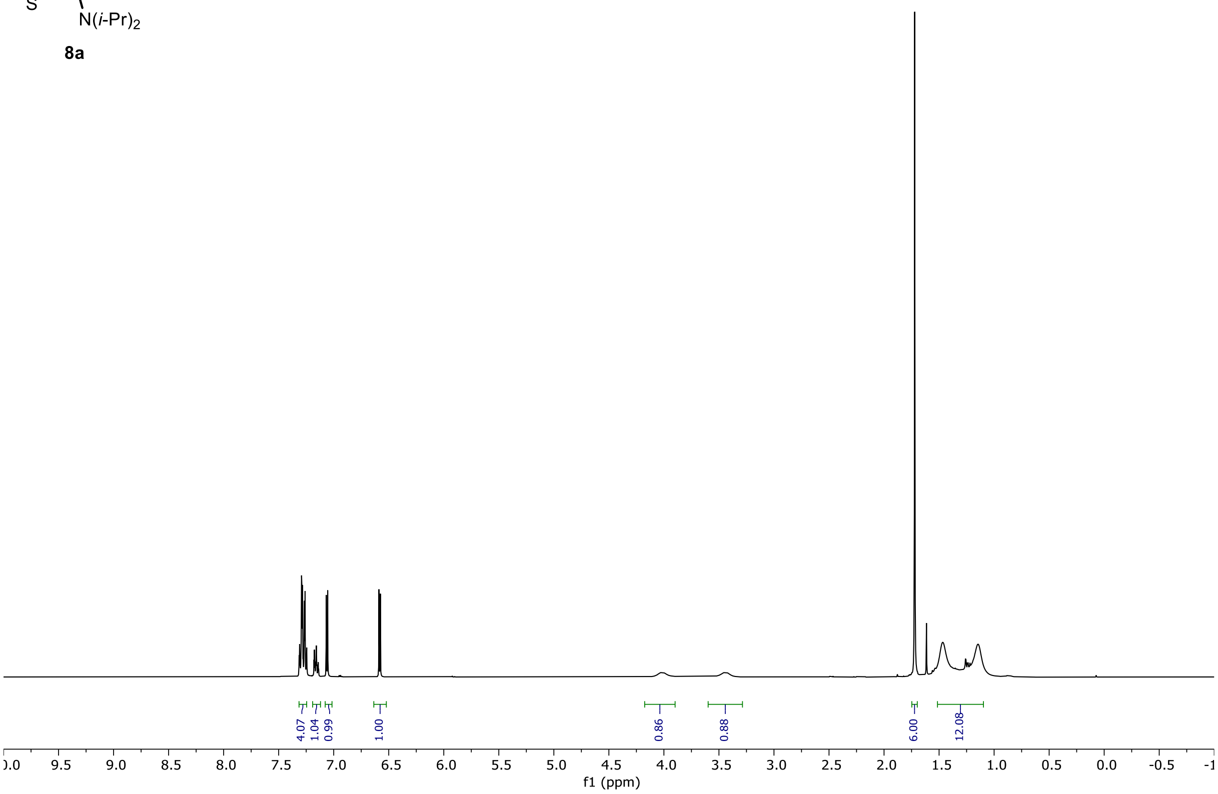
4.07
1.04
0.99
1.00
0.86
0.88
6.00
12.08
f1 (ppm)

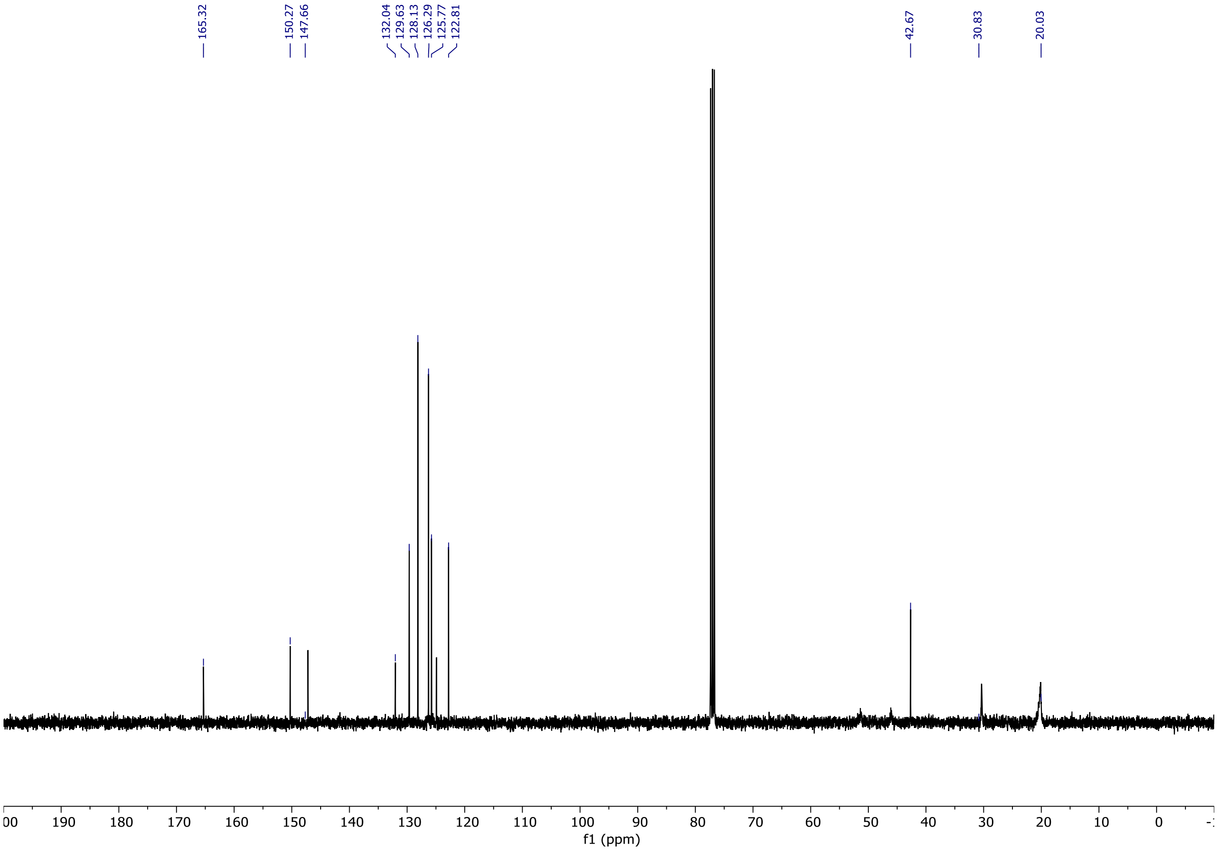
165.32
150.27
147.66
132.04
129.63
128.13
126.29
125.77
122.81
42.67
30.83
20.03
f1 (ppm)

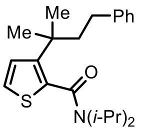
Me
Me
Ph
O
S
N(i-Pr)2

8b

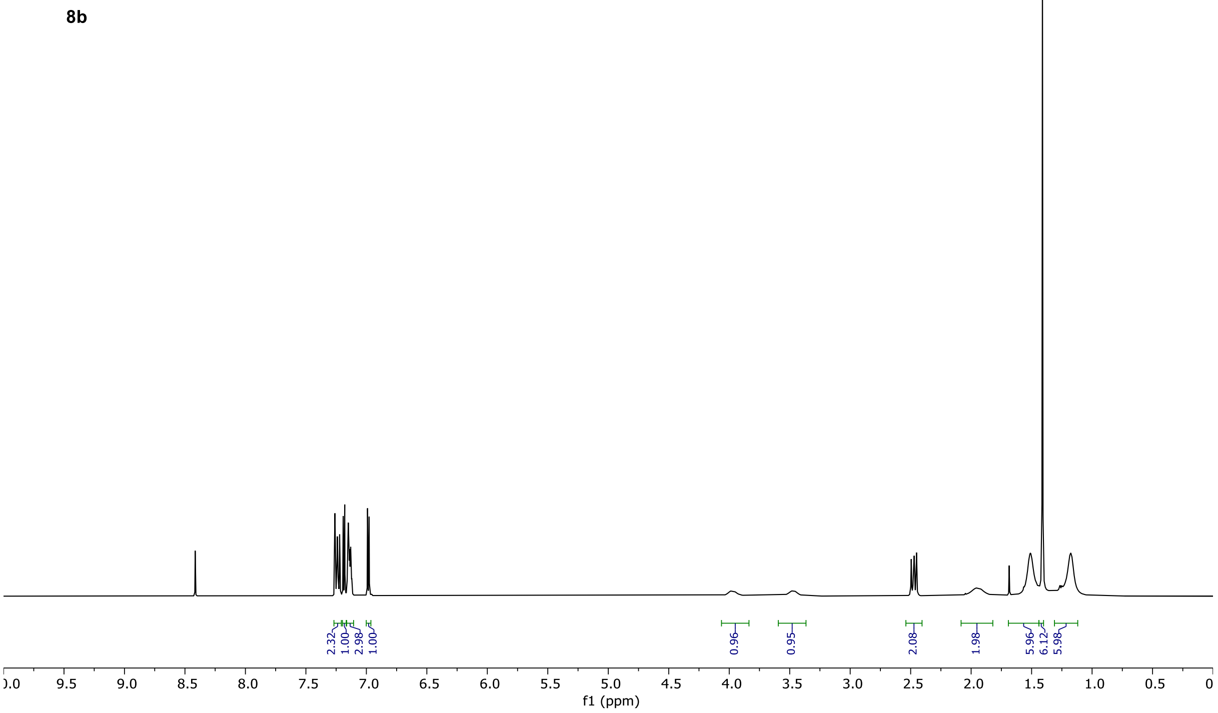
2.32
1.00
2.98
1.00
0.96
0.95
2.08
1.98
5.96
6.12
5.98
9.5
9.0
8.5
8.0
7.5
7.0
6.5
6.0
5.5
5.0
4.5
4.0
3.5
3.0
2.5
2.0
1.5
1.0
0.5
0
f1 (ppm)

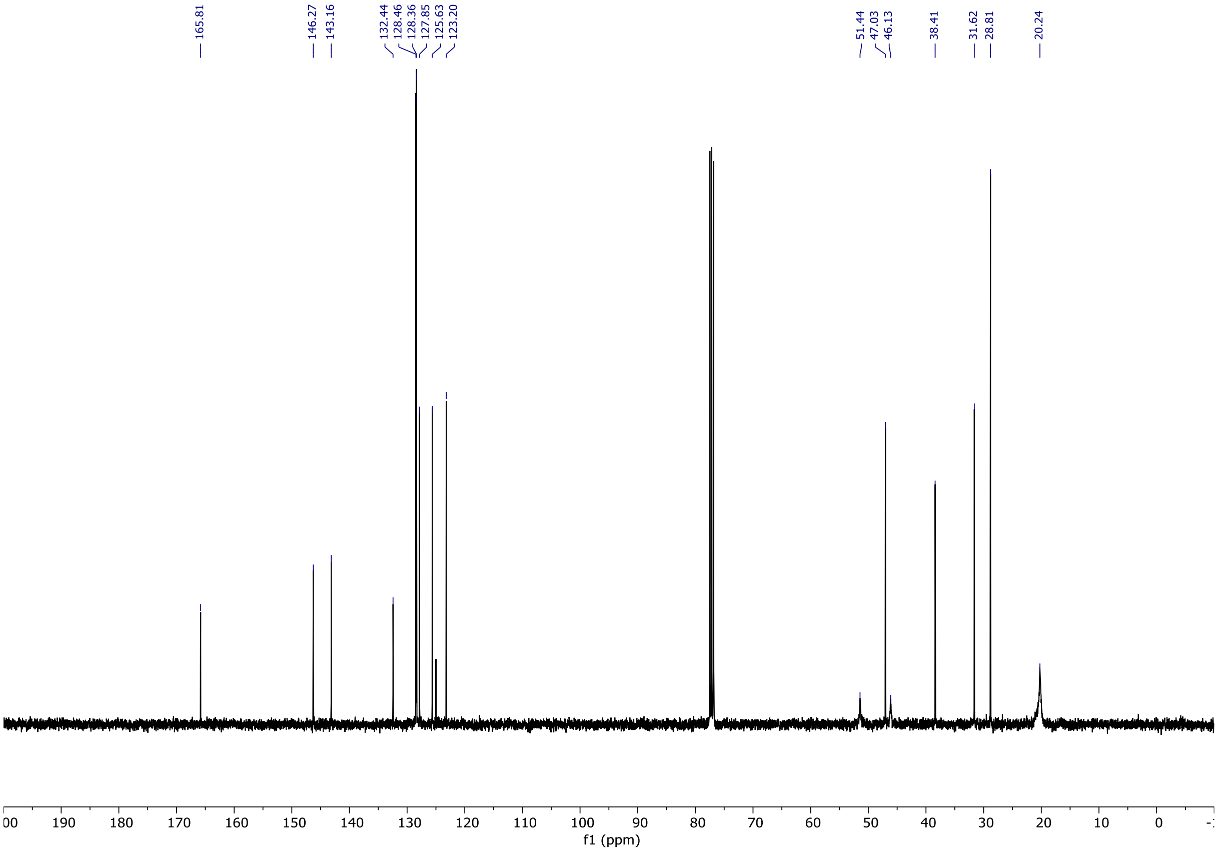
165.81
146.27
143.16
132.44
128.46
128.36
127.85
125.63
123.20
51.44
47.03
46.13
38.41
31.62
28.81
20.24
190
180
170
160
150
140
130
120
110
100
90
80
70
60
50
40
30
20
10
0
f1 (ppm)

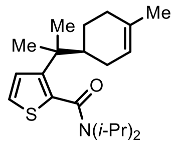
Me
Me
Me
O
S
N(i-Pr)2

8c

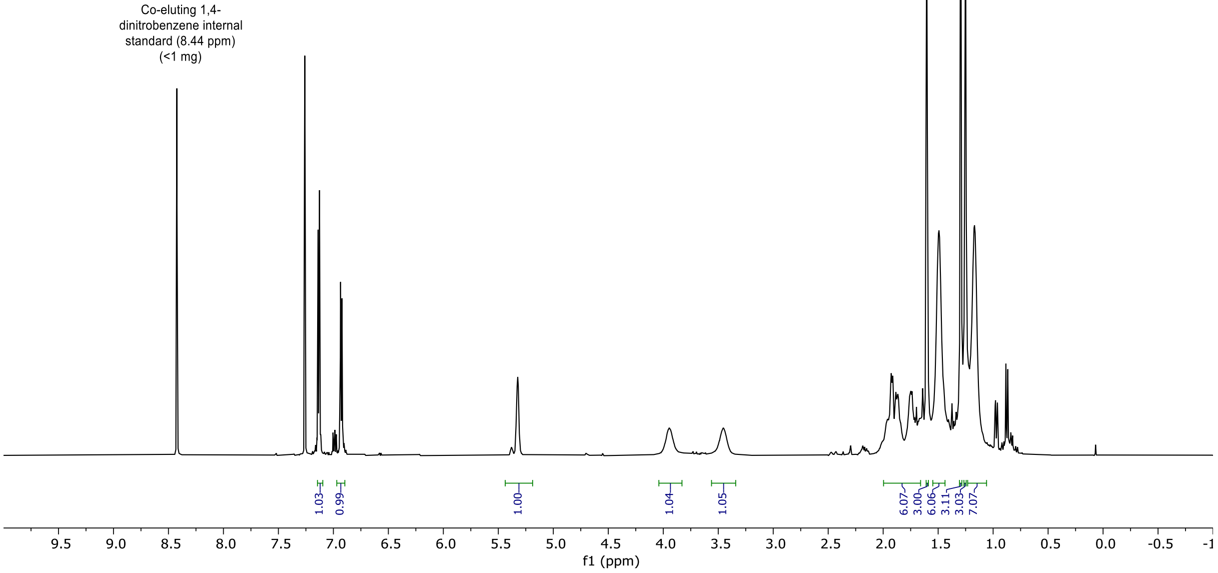
Co-eluting 1,4-dinitrobenzene internal standard (8.44 ppm) (<1 mg)
1.03
0.99
1.00
1.04
1.05
6.07
3.00
6.06
3.11
3.03
7.07
f1 (ppm)

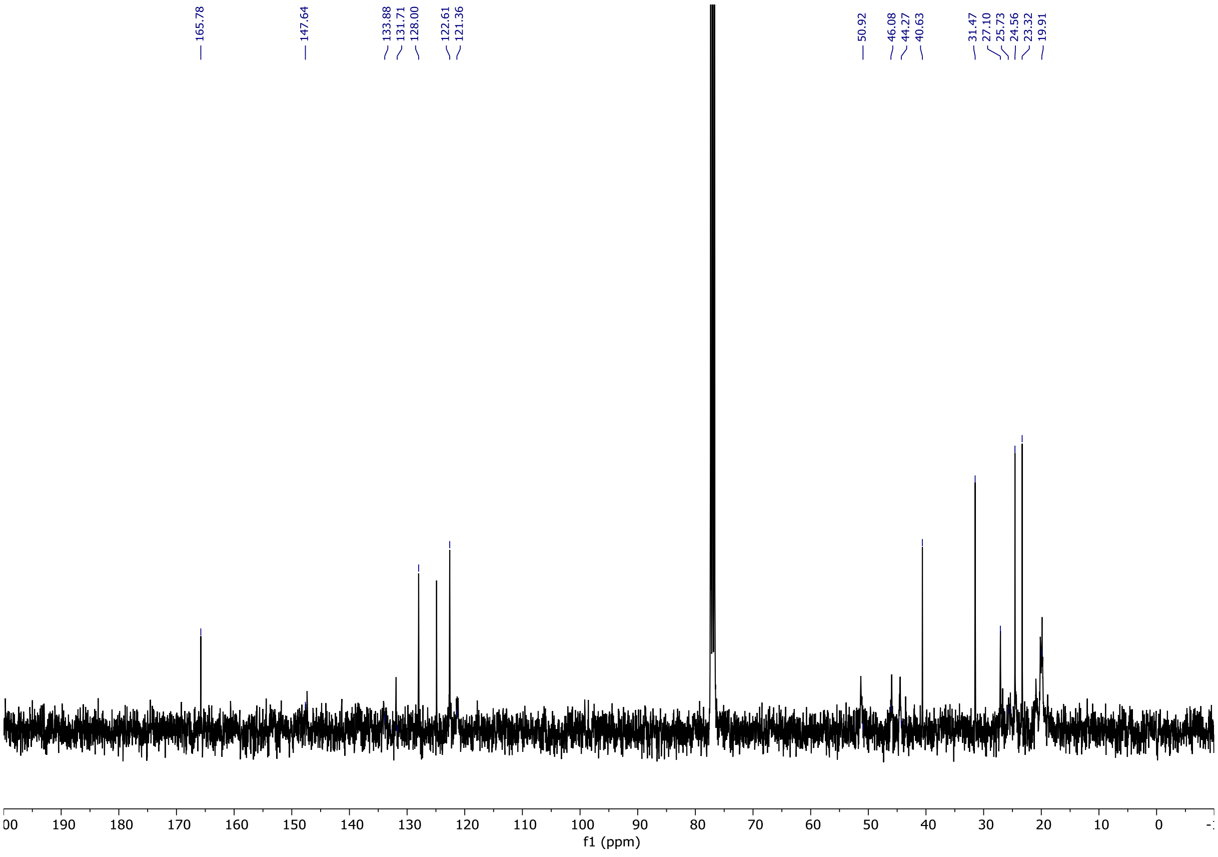
165.78
147.64
133.88
131.71
128.00
122.61
121.36
50.92
46.08
44.27
40.63
31.47
27.10
25.73
24.56
23.32
19.91
f1 (ppm)

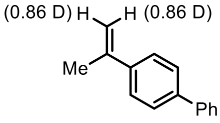

deuterio-**9**

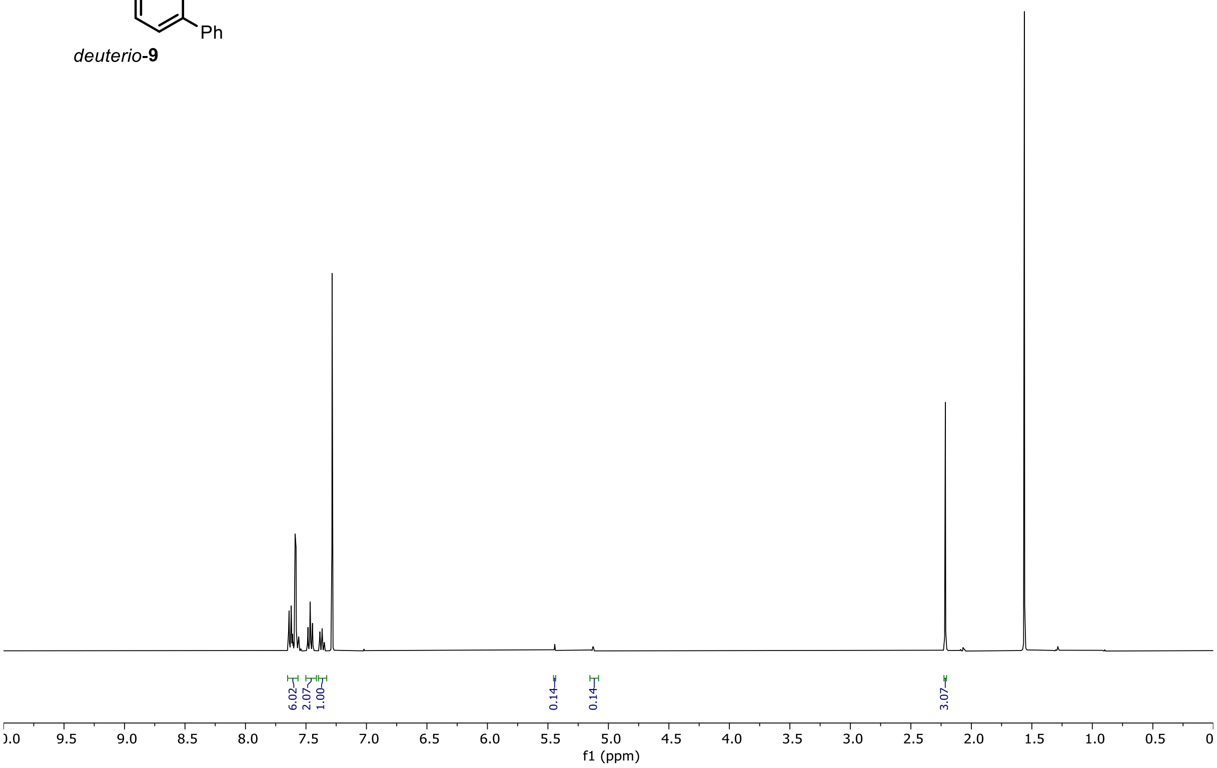

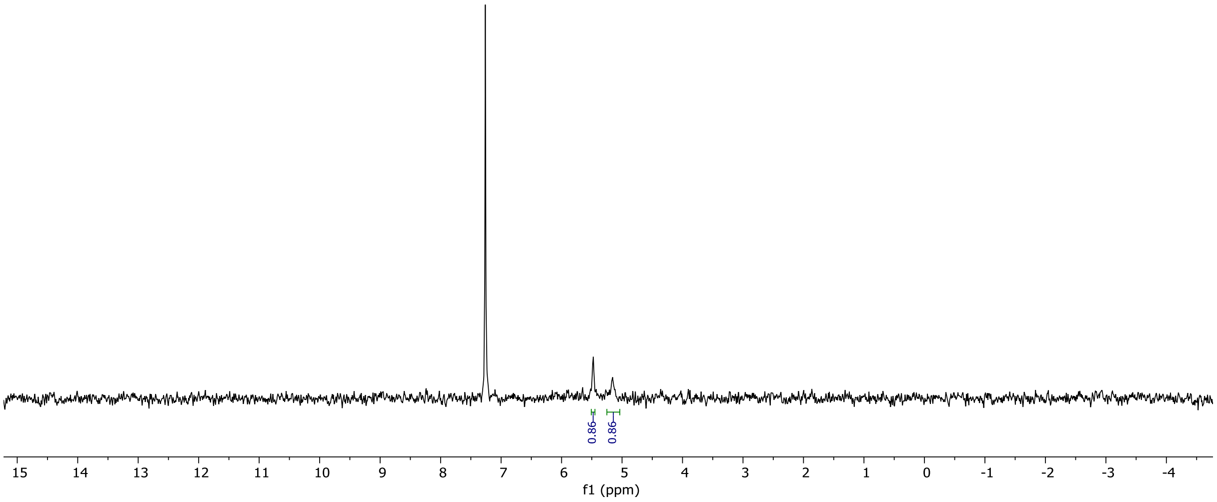

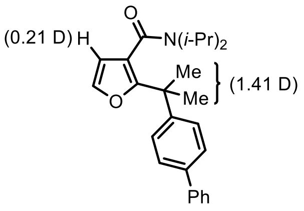
(0.21 D) H
O
N(i-Pr)2
Me
Me
(1.41 D)
O
Ph

deuterio-**4i**

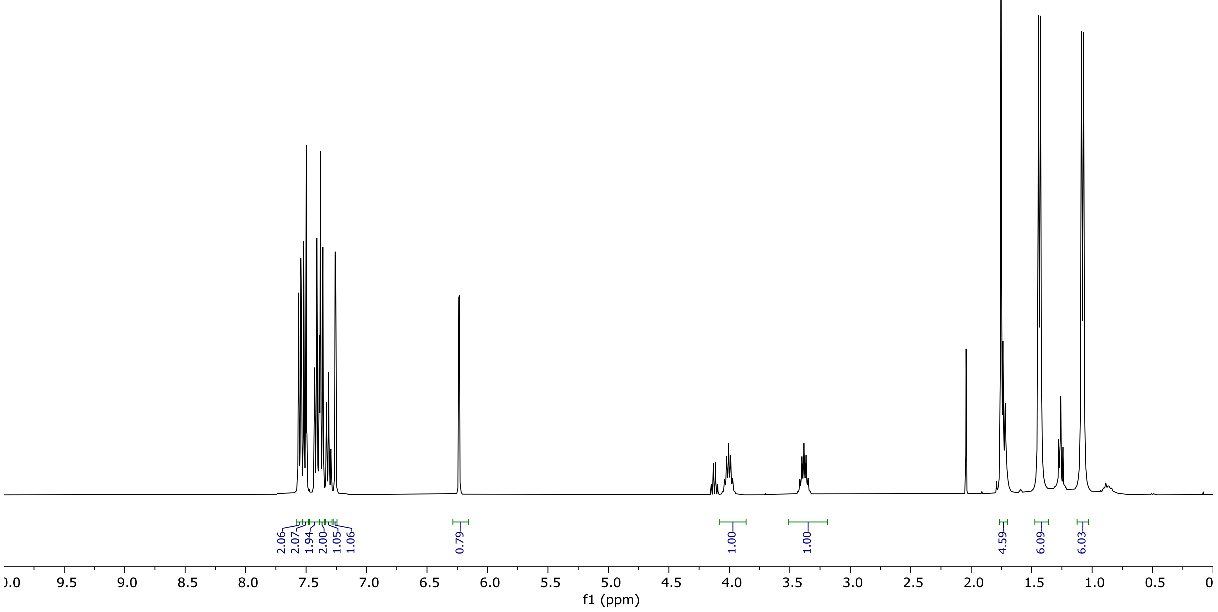
2.06
2.07
1.94
2.00
1.05
1.06
0.79
1.00
1.00
4.59
6.09
6.03
10.0 9.5 9.0 8.5 8.0 7.5 7.0 6.5 6.0 5.5 5.0 4.5 4.0 3.5 3.0 2.5 2.0 1.5 1.0 0.5 0
f1 (ppm)

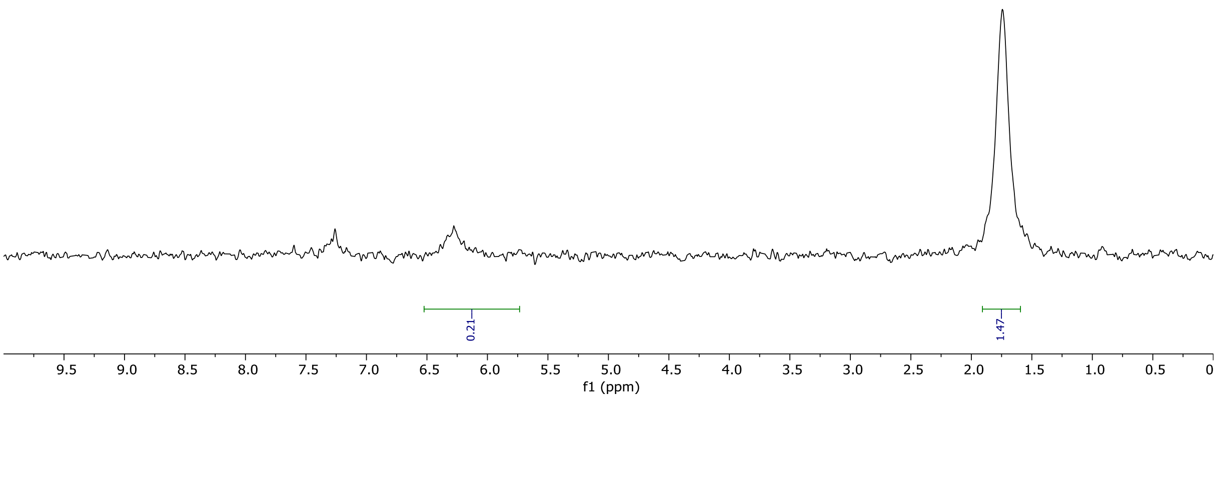
0.21
1.47
9.5 9.0 8.5 8.0 7.5 7.0 6.5 6.0 5.5 5.0 4.5 4.0 3.5 3.0 2.5 2.0 1.5 1.0 0.5 0
f1 (ppm)

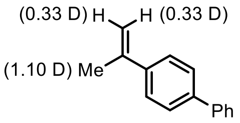
(0.33 D) H
H (0.33 D)
(1.10 D) Me
Ph

deuterio-**9'**

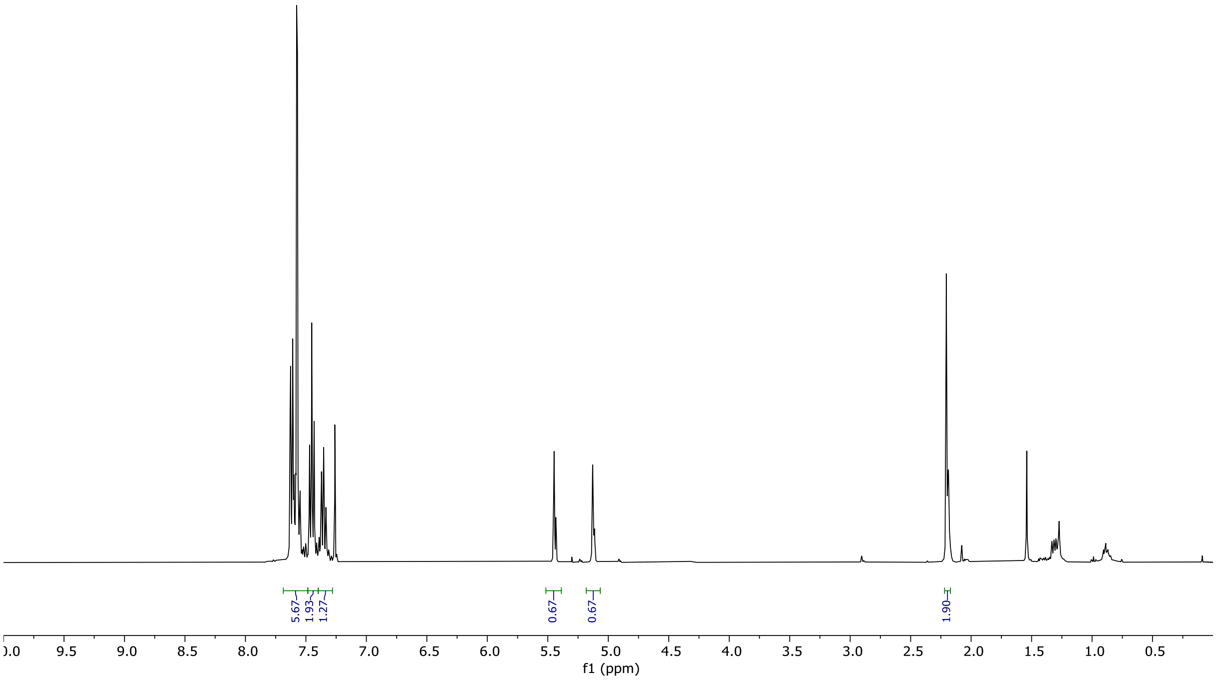
5.67
1.93
1.27
0.67
0.67
1.90
f1 (ppm)

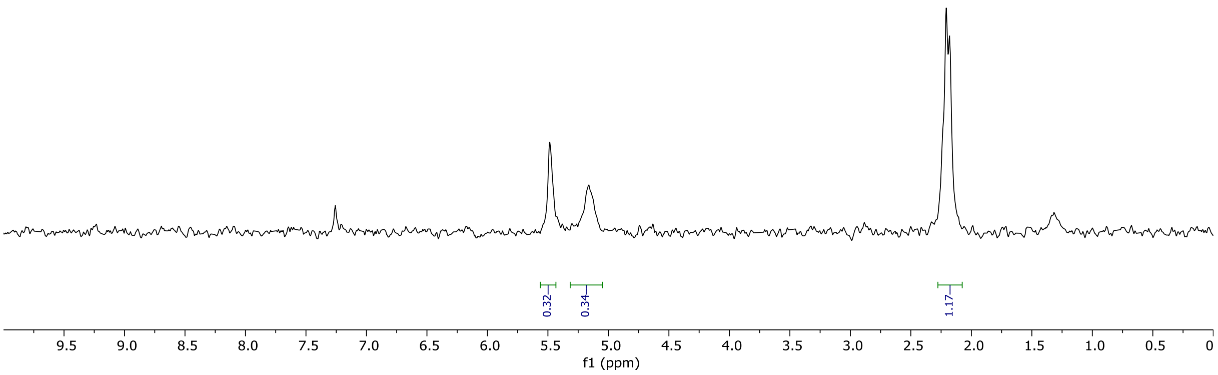
0.32
0.34
1.17
f1 (ppm)

References

1. [a] Crisenza, G. E. M.; McCreanor, N. G.; Bower, J. F. *J. Am. Chem. Soc.* **2014**, *136*, 10258; [b] Tsuchikama, K.; Kasagawa, M.; Endo, K.; Shibata, T. *Org. Lett.* **2009**, *11*, 1821.
2. Busireddy, M. R.; Mantena, V. N. R.; Chereddy, N. R.; Shanigaram, B.; Kotamarthi, B.; Biswas, S.; Sharma, G. D.; Vaidya, J. R. *Org. Electron.* **2016**, *37*, 312.
3. Grélaud, S.; Cooper, P.; Feron, L. J.; Bower, J. F. *J. Am. Chem. Soc.* **2018**, *140*, 9351.
4. Hua, Z.; Vassar, V. C.; Ojima, I. *Org. Lett.* **2003**, *5*, 3831.
5. Ohshima, T., Iwasaki, T., Maegawa, Y., Yoshiyama, A., Mashima, K. *J. Am. Chem. Soc.*, **2008**, *130*, 2944.
6. Kumar, P. S.; Kumar, G. S.; Kumar, R. A.; Reddy, N. V.; Reddy, K. R. *Eur. J. Org. Chem.* **2013**, 1218.
7. Zoller, B; Zapp, J; Huy, P. H. *Eur. J. Org. Chem*. **2020**, 26, 9632.
8. Smedley, C. J.; Barrow, A. S.; Spiteri, C.; Giel, M.-C.; Sharma, P.; Moses, J. E. *Chem. Eur. J.* **2017**, 23, 9990.
9. Kinoshita, T.; Icbinari, D.; Sinya, J. *J. Heterocycl. Chem.* **1996**, *33*, 1313.
10. Montaña, Á. M.; Grima, P. M.; Castellví, M.; Batalla, C.; Font-Bardia, M. *Tetrahedron* **2012**, *68*, 9982.
11. Wang, H.; Shröder, N.; Glorius, F. *Angew. Chem. Int. Ed.* **2013**, 52, 5386.
12. Movahhed, S.; Westphal, J.; Dindaroğlu, M.; Falk, A.; Schmalz, H.-G. *Chem. Eur. J.* **2016**, *22*, 7381.
13. Molloy, J. J.; Metternich, J. B.; Daniliuc, C. G.; Watson, A. J. B.; Gilmour, R. *Angew. Chem. Int. Ed.* **2018**, *57*, 3168.
14. Phan, D. H. T.; Kou, K. G. M.; Dong, V. M. *J. Am. Chem. Soc.* **2010**, *132*, 16354.
15. Purohit, V. C.; Allwein, S. P.; Bakale, R. P. *Org. Lett.* **2013**, *15*, 1650.
16. Siu, J. C.; Parry, J. B.; Lin, S. *J. Am. Chem. Soc.* **2019**, *141*, 2825.
17. Walker, J. C. L.; Oestreich, M. *Org. Lett.* **2018**, *20*, 6411.
18. Cleary, P. A.; Woerpel, K. A. *Org. Lett.* **2005**, *7*, 5531.
19. Xiong, B.; Zeng. X.; Geng, S.; Chen, S.; He, Y.; Feng, Z. *Green Chem*. **2018**, 20, 4521.
20. Hirano, M.; Ueda, T.; Komine, N.; Komiya, S.; Nakamura, S.; Deguchi, H.; Kawauchi, S. *J. Organomet. Chem.* **2015**, *797*, 174.
21. Saunders, W. H.; Melander, L., In *Reaction Rates of Isotopic Molecules*, Wiley, New York, 1981.
22. Singleton, D. A.; Thomas, A. A. *J. Am. Chem. Soc.* **1995**, *117*, 9357.
23. Huang, G.; Liu, P. *ACS Catal.* **2016**, *6*, 809-920.
24. Frisch, M. J.; Trucks, G. W.; Schlegel, H. B.; Scuseria, G. E.; Robb, M. A.; Cheeseman, J. R.; Scalmani, G.; Barone, V.; Mennucci, B.; Petersson, G. A.; Nakatsuji, H.; Caricato, M.; Li, X.; Hratchian, H. P.; Izmaylov, A. F.; Bloino, J.; Zheng, J.; Sonnenberg, J. L.; Hada, M.; Ehara, M.; Toyota, K.; Fukuda, R.; Hasegawa, J.; Ishida, M.; Nakajima, T.; Honda, Y.; Kitao, O.; Nakai, H.; Vreven, T.; Montgomery, J. A.; Peralta, J. E.; Ogliaro, F.; Bearpark, M.; Heyd, J. J.; Brothers, E.; Kudin, K. N.; Staroverov, V. N.; Kobayashi, R.; Normand, J.; Raghavachari, K.; A. Rendell, J. C.; Burant, S.; Iyengar, S.; Tomasi, J.; Cossi, M.; Rega, N.; Millam, J. M.; Klene, M.; Knox, J. E.; Cross, J. B.; Bakken, V.; Adamo, C.; Jaramillo, J.; Gomperts, R.; Stratmann, R. E.; Yazyev, O.; Austin, A. J.; Cammi, R.; Pomelli, C.; Ochterski, J. W.; Martin, R. L.; Morokuma, K.; Zakrzewski, V. G.; Voth, G. A.; Salvador, P.; Dannenberg, J. J.; Dapprich, S.; Daniels, A. D.; Farkas, O.; Foresman, J. B.; Ortiz, J. V.; Cioslowski, J.; Fox, D. J. Gaussian 16, Revision A.03. Gaussian, Inc.: Wallingford CT 2016.
25. Becke, A. D. *J. Chem. Phys.* **1993**, *98*, 5648.
26. Stephens, P. J.; Devlin, F. J.; Chabalowski, C. F.; Frisch, M. J. *J. Phys. Chem.* **1994**, *98*, 11623.

27. Andrae, D.; Häußermann, U.; Dolg, M.; Stoll, H.; Preuß, H. *Theor. Chim. Acta* **1990**, *77*, 123.
28. Hehre, W. J.; Ditchfield, R.; Pople, J. A. *J. Chem. Phys.* **1972**, *56*, 2257.
29. Simón, L.; Goodman, J. M. *Org. Biomol. Chem.* **2011**, *9*, 689.
30. Weigend, F.; Ahlrichs, R. *Phys. Chem. Chem. Phys.* **2005**, *7*, 3297.
31. Mennucci, B.; Cammi, R.; Tomasi, J. *J. Chem. Phys.* **1998**, *109*, 2798.
32. Grimme, S. *Chem. Eur. J.* **2012**, *18*, 9955.
33. Funes-Ardoiz, I.; Paton, R. S. Goodvibes: Version 2.0.3. 2018.
34. Lam, Y. -H.; Grayson, M. N.; Holland, M. C.; Simon, A.; Houk, K. N. *Acc. Chem. Res.* **2016**, *49*, 750.
35. Grayson, M. N.; Krische, M. J.; Houk, K. N. *J. Am. Chem. Soc.* **2015**, *137*, 8838.
36. Pedregal, J. R.-G.; Gómez-Orellana, P.; Maréchal, J.-D. *J. Chem. Inf. Model.* **2018**, *58*, 561.
